# Self‐Determination Theory and Workplace Outcomes: A Meta‐Analysis

**DOI:** 10.1002/smi.70151

**Published:** 2026-02-10

**Authors:** Martin S. Hagger, Kaylyn McAnally Star

**Affiliations:** ^1^ Department of Psychological Sciences University of California Merced California USA; ^2^ Health Sciences Research Institute University of California Merced California USA; ^3^ Faculty of Sport and Health Sciences University of Jyväskylä Jyväskylä Finland; ^4^ School of Applied Psychology Griffith University Brisbane Australia; ^5^ School of Rehabilitative and Health Sciences Regis University Denver Colorado USA

**Keywords:** health behaviour, job stress, meta‐analysis, motivation, psychological well‐being, theoretical models of stress and coping

## Abstract

Applications of self‐determination theory (SDT) in workplace contexts have demonstrable utility in identifying motivational determinants of adaptive workplace outcomes (e.g., job satisfaction, work engagement) implicated in employee well‐being and work productivity and effective stress management. Studies have found consistent SDT‐stipulated associations between theory constructs (e.g., psychological need satisfaction, autonomous motivation) and adaptive workplace outcomes, although with substantive between‐study variability. The current meta‐analysis synthesised research reporting relations among SDT constructs and workplace outcomes and tested a novel model specifying SDT‐stipulated predictions. In the proposed model, associations between employees' perceived need support from workplace leaders and adaptive and maladaptive workplace outcomes were mediated by autonomy, competence, and relatedness need satisfaction and autonomous and controlled motivation forms. Multilevel meta‐analysis of data from studies identified in a systematic database search (*k* = 192) confirmed SDT‐consistent patterns of averaged correlations among theory constructs and outcomes. Meta‐analytic structural equation modelling analysis of the proposed model indicated positive direct perceived need support–need satisfaction, need satisfaction–autonomous motivation forms, and autonomous motivation forms–adaptive outcome effects consistent with theory. Most salient, indirect effects of need support on adaptive workplace outcomes, and negative indirect effects on maladaptive outcomes, mediated by need satisfaction and autonomous motivation forms were observed. Moderation analyses indicated that model varied effects by work type, employee type, and country cultural orientation and GDP ranking. The analysis updates and advances prior research syntheses of SDT in workplace contexts providing robust support for the model including mechanism‐related effects and identification of key moderators on which model effects are conditional.

## 1 Introduction

Employee motivation, perceived workplace success, and perceived employer support are positively associated with adaptive work‐, health‐, and stress‐related employee outcomes (e.g., well‐being, effective stress management, job satisfaction, work engagement), and negatively associated with maladaptive outcomes (e.g., job stress, boredom, fatigue, burnout, work disengagement, job dissatisfaction; Jackson and Frame, 2018; Gagné et al. 2022). Job engagement and satisfaction are also linked to enhanced productivity and reduced absenteeism and presenteeism in the workplace (e.g., Neuber et al. 2021). Workplace authority figures (e.g., managers, supervisors) therefore place high value in instigating workplace interventions to enhance employee work engagement, job satisfaction, and well‐being. Further, intervention studies utilising strategies that enhance forms of motivation that promote reflect interest and self‐endorsement in work tasks (e.g., intrinsic and autonomous motivation) have been shown to promote greater employee responsibility, ownership, choice, and engagement (Galletta et al. 2019; Moon et al. 2019; Slemp et al. 2021). This research has largely been informed by motivational theories that provide a conceptual basis for techniques and processes that promote intrinsic and autonomous motivation.

Pre‐eminent among these theories is self‐determination theory (SDT; Deci and Ryan 1985; Ryan and Deci 2017; Ryan 2023), which describes how forms of motivation relate to individual functioning, behaviour, and outcomes (e.g., psychological well‐being, mental health). The theory posits that the quality of motivation, rather than its quantity, is the primary determinant of intention, behavioural uptake, persistence, and adaptive outcomes such as positive affect, perceived interest and agency, and psychological health (Ryan and Deci 2000). The theory makes the global distinction between autonomous and controlled forms of motivation. Autonomous motivation reflects acting for self‐determined reasons, including for the inherent interest or enjoyment derived from the task, or because the behaviour aligns with personally valued goals. By contrast, controlled motivation reflects acting out of externally referenced contingencies (e.g., rewards, praise), or out of perceived obligation or pressure from external agents (e.g., authority figures). Research has subsequently distinguished between multiple forms of autonomous and controlled motivation, known as regulation ‘styles’, which delineate a continuum of autonomy or ‘internalisation’—the extent to which actions and tasks are performed for autonomous or controlled reasons (Ryan and Connell 1989)—referred to as the perceived locus of causality continuum. The continuum comprises interim constructs proposed to be situated at graduated intervals including autonomous (intrinsic motivation, and integrated and identified regulation) and controlled (introjected and external regulation) forms. Importantly, the theory predicts that satisfaction of basic psychological needs, namely, autonomy (i.e., the desire for volition), competence (i.e., the desire for mastery), and relatedness (i.e., the desire for personal connection), determines the extent to which individuals are likely to experience behaviours as autonomous or controlled motivated. The theory has been widely applied to predict intention, behaviour, and adaptive outcomes (e.g., well‐being, optimal functioning, satisfaction) in multiple contexts and for a range of behaviours (Bureau et al. 2021; Ng et al. 2012).

Consistent with research applying SDT in broader contexts (for reviews see Ryan and Deci 2017; Ryan 2023), research applying the theory in workplace settings has indicated that autonomous forms of motivation are typically associated with adaptive outcomes in occupational contexts, including enhanced engagement in work‐related tasks and job satisfaction, and reduced stress and burnout (Coxen et al. 2021; Messmann et al. 2022; Wörtler et al. 2020). By contrast, controlled forms of motivation are associated with maladaptive outcomes, including absenteeism and burnout, and with lower work engagement and higher job turnover. In addition, consistent with proposed generalised models based on the theory (see Ryan et al. 2008; Williams et al. 2004), studies have shown that relations between autonomous forms of motivation and adaptive workplace outcomes, and between controlled forms of motivation and maladaptive outcomes, in employees is mediated by the extent to which their basic needs are satisfied in the workplace (Graves and Luciano 2013; van Hooff and van Hooft 2017). Furthermore, research informed by the theory has outlined how adoption of autonomy‐supportive strategies in workplace interventions, such as training authority figures to adopt autonomy‐supportive practices and behaviours (e.g., prompting choice, facilitating autonomous goals, providing feedback) and avoid controlling behaviours (e.g., controlling language), promote adaptive workplace outcomes and does so by enhancing autonomous forms of motivation (Cheon and Reeve 2013; Slemp et al. 2018, 2024; Su et al. 2011). Analogously, employees' beliefs in the degree to which their employers or authority figures in the workplace support their autonomy has been shown to be associated with adaptive workplace outcomes mediated by autonomous and controlled forms of motivation and need satisfaction, which is consistent with the proposed generalised models (Dahling and Lauricella 2017; Gillet et al. 2013; van Hooff and van Hooft 2017). Taken together, the extant research applying SDT in workplace contexts seems to converge on autonomous forms of motivation as important correlates of adaptive workplace outcomes, and controlled forms of motivation as correlates of maladaptive outcomes, and indicated that need satisfaction and autonomous motivation forms are salient process‐related constructs that serve to mediate effects of workplace leader support for psychological needs and adaptive workplace outcomes in employees (Graves and Luciano 2013; van Hooff and van Hooft 2017). Furthermore, interventions in which workplace leaders apply autonomy‐supportive techniques and behaviours are associated with more adaptive workplace outcomes through the mediation of need satisfaction and autonomous forms of motivation (Graves and Luciano 2013; Hardré and Reeve 2009).

Meta‐analytic reviews of research applying SDT in workplace contexts have also supported these associations (Van den Broeck et al. 2021; Slemp et al. 2018). Of these, Slemp et al. (2018) analysis has provided the most wide‐reaching and comprehensive evidence, with a focus on leader autonomy support. A key component of their analysis was the application of structural equation modelling to test a model consistent with Ryan et al.’s (2008) generalised model using their meta‐analytically synthesised data. Corroborating primary research findings, the analysis indicated that leader autonomy support in the workplace (e.g., supervisors, managers toward employees or teams) was related with greater internalized motivation toward work and more positive work behaviours in employees. Alongside this, Slemp et al. also tested the effects of key moderator variables on the averaged bivariate correlations between SDT constructs and workplace outcomes in their meta‐analysis such as the source of autonomy support as coded by the closeness or proximity of workplace leaders to their employees (i.e., direct supervisors compared with distant managers) and country cultural orientation (individualist and collectivist orientations). Their findings indicated that there were no moderation effects of autonomy support source proximity or country cultural orientation in the averaged correlation between leader autonomy support and workplace outcomes (Slemp et al. 2018).

Despite widespread support for SDT predictions in the workplace, a number of limitations of the extant research have been identified and represent salient gaps in the current evidence that warrant future research. Specifically, considerable variability has been observed in studies examining associations between autonomous and controlled forms of motivation and workplace outcomes across studies. Analogously, prior meta‐analyses applying SDT in the workplace have also observed non‐trivial residual heterogeneity in the correlations among SDT constructs and outcomes, even after examination of candidate moderators (Slemp et al. 2018; Van den Broeck et al. 2021). A further limitation is that studies applying SDT in the workplace context have seldom tested some of the key mechanisms outlined in Ryan et al. ’s (2008) model. In particular, the mediating role of autonomous and controlled forms of motivation on the effects of basic psychological need support and need satisfaction on workplace outcomes is not routinely tested. This dearth of research hinders the drawing of definitive conclusions with respect to the proposed mechanistic effects based on the available evidence in this context.

Alongside these observations, there are some notable methodological limitations of the meta‐analysis conducted by Slemp et al. (2018). Specifically, the authors did not include research items that estimated key effects among SDT constructs (e.g., correlations among need satisfaction constructs) and between constructs and outcomes (e.g., correlations between need satisfaction and well‐being and work engagement outcomes) required for estimating the model. Instead, these effects were imputed using data from a prior meta‐analysis. This practice likely introduced bias to the model parameter estimates (for a discussion see Hagger and Hamilton 2024a) such that moderator tests had to be confined to univariate correlations among theory constructs and outcomes rather than model effects. In addition, Slemp et al. suggested that their model findings confirmed their hypothesised mediation effects, but they did not report the indirect effects necessary to provide definitive support for mediation and permit evaluation of their effect size and variability. Further, their data were analysed using meta‐analytic path analysis, which treats the meta‐analytic data as correlations with a rule‐of‐thumb estimate for the sample size used because the studies contributing data for each correlation varied (Cheung 2015; Hagger and Hamilton 2024a). This approach likely leads to inaccurate variance and confidence interval estimates (Jak and Cheung 2024). Finally, Slemp et al. confined their analysis to research examining the effects of leader autonomy support, and recommended the need to examine effects of broader aspects of the work organization such as support for other basic psychological needs (i.e., competence and relatedness), or support from additional sources (e.g., work colleagues), on workplace outcomes.

### 1.1 The Present Study

In the present study, we aimed to conduct a meta‐analytic synthesis of research applying SDT in workplace contexts to (a) provide precise estimates of correlations among key theory constructs (i.e., basic psychological need support, autonomous and controlled motivational forms, and basic psychological need satisfaction) and adaptive (work engagement, well‐being, job performance, job satisfaction) and maladaptive (turnover, burnout) outcomes; (b) estimate the unique effects of SDT constructs on workplace outcomes as proposed in Ryan et al.’s (2008) model including estimation of key mediation effects that relate to process, particularly the indirect effects of psychological need support on adaptive and maladaptive outcomes mediated by need satisfaction and forms of motivation from SDT; (c) resolve some shortcomings of previous meta‐analyses of SDT workplace research by using a matrix of effect sizes that includes all available data and using fit‐for‐purpose meta‐analytic structural equation modelling techniques to test our proposed model; and (d) examine the effects of key moderator variables on the parameter estimates of our model test.

We expected our analysis to yield highly precise average point and variability estimates of the associations between SDT constructs and workplace outcomes in accordance with Ryan et al.’s (2008) model based on current research. Most importantly, we anticipated that our research would add to current knowledge by building on and extending prior meta‐analyses of theory applications in the workplace and tests of Ryan et al.’s. Specifically, the analysis was expected to update and broaden the studies and constructs (e.g., satisfaction of all basic needs including autonomy, competence, and relatedness and support from all influential leadership figures responsible for employee supervision, management, and welfare) included in the analysis; provide estimates of the unique effects of all SDT constructs on workplace outcomes consistent with the proposed model; test the effects of unique moderator variables (e.g., work and employee type, country GDP) on model effects; and address the limitations of the analytic methods used in prior meta‐analyses (e.g., use of meta‐analytic structural equation modelling analytic techniques, refraining from effect size imputation). The findings are expected to be of value to researchers by providing highly accurate estimates of model effects that researchers conducting studies testing the model in new contexts might expect to find and may also be used as highly precise, informative priors for analyses of data arising from new studies (e.g., analyses adopting Bayesian statistical methods). We also expected our research to provide initial upstream evidence of value to those interested in promoting better workplace outcomes in employees by identifying possible targets for subsequent intervention research testing the effects of techniques or strategies that workplace authority figures may adopt to promote adaptive employee motivation and workplace outcomes (Hagger et al. 2020).

#### 1.1.1 Meta‐Analysis of Correlations

Our first aim was to provide meta‐analytic estimates of the average size and variability of the zero‐order correlations between SDT constructs and workplace outcomes. Hypothesised correlations (HC), consistent with theory and observations reported in previous meta‐analyses of theory applications in work and health contexts (e.g., Slemp et al. 2018; Ng et al. 2012), are summarised (Table A1 Supporting Information S1: A). Specifically, we expected positive, non‐zero averaged correlations between autonomous forms of motivation (i.e., autonomous and intrinsic motivation, integrated regulation, identified regulation), basic psychological need satisfaction (i.e., autonomy, competence, relatedness), and need support (HC1a–l), and between autonomous forms of motivation and adaptive workplace outcomes (work engagement, well‐being, job performance, job satisfaction; HC2a–l) across studies. We also expected negative, non‐zero averaged correlations between controlled forms of motivation (e.g., controlled motivation and external regulation, introjected regulation), basic psychological need satisfaction, and need support (HC3a–l), and between controlled forms of motivation and adaptive workplace outcomes (H4a–l) across studies. Analogously, we also predicted negative correlations between autonomous forms of motivation and maladaptive workplace outcomes (burnout, turnover; HC5a–f) and positive correlations between controlled forms of motivation and these outcomes (H6a–f).

#### 1.1.2 Model Test

Our second aim was to use the meta‐analytically derived matrix of correlations among the SDT constructs and workplace outcomes as input for a comprehensive, fit‐for‐purpose test of Ryan et al. ’s (2008) model in which effects of basic psychological need support on adaptive and maladaptive workplace outcomes were hypothesised to be mediated by need satisfaction (autonomy, competence, and relatedness) and autonomous and controlled forms of motivation. We expected this analysis to build on and extend the model previously estimated in a prior meta‐analysis in this context (Slemp et al. 2018). A key advantage the model test is that it enables estimation of the unique or independent effects of each construct on other constructs and outcomes as proposed in the model while taking the simultaneous effects of all other construct effects into account. This is the meta‐analytic equivalent to the multivariate analyses (e.g., path analysis, structural equation modelling) considered de rigueur to test these predictions in primary studies (Cheung 2015; Hagger and Hamilton 2024a).

**FIGURE 1:**
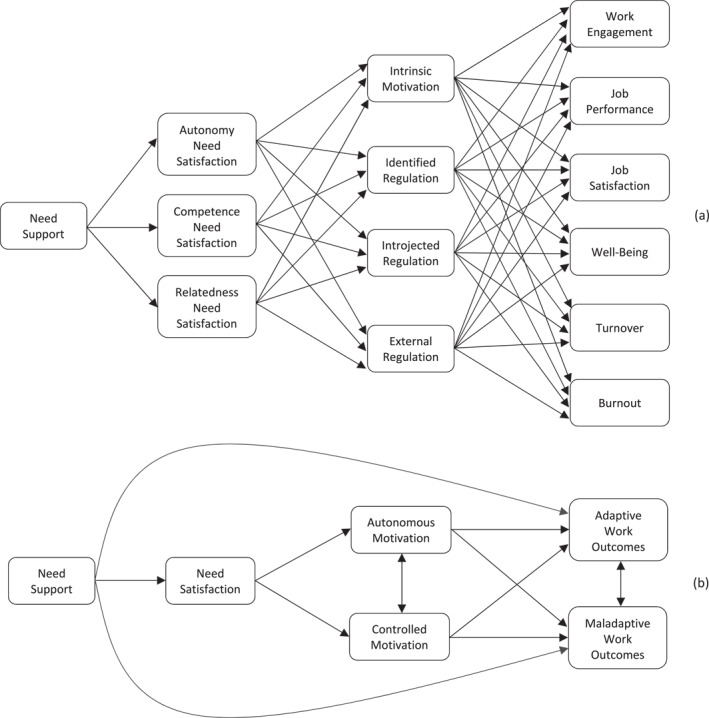
Proposed model indicating hypothesised relations between need support, basic psychological need satisfaction, motivation regulation, and work outcomes for the full (panel a) and truncated (panel b) Models. Intercorrelations among work‐related outcomes for the full model (panel a) omitted for clarity.

A diagram summarising the specific predictions of our proposed full model is presented in Figure 1 with a full itemised breakdown of each hypothesised model effect (HF) provided in an accompanying table (Table A2, Supporting Information S1: A). Specifically, we hypothesised non‐zero positive direct effects of psychological need support on autonomy, competence, and relatedness need satisfaction (HF1a‐c); non‐zero effects of autonomy (HF2a‐d), competence (HF3a‐d), and relatedness (HF4a–d) need satisfaction constructs on each motivational regulation (intrinsic motivation, identified, introjected, and external regulation)—effects were expected to vary in sign and size consistent with simplex‐like patterns observed in previous studies (e.g., Chatzisarantis et al. 2003); non‐zero effects of the intrinsic motivation (HF5a–f), and the identified (HF6a–f), introjected (HF7a–f), and external (HF8a–f) regulation forms on each adaptive (work engagement, well‐being, job performance, job satisfaction) and maladaptive (turnover, burnout) outcome, again with signs consistent with prior research (e.g., positive and negative effects of autonomous motivation forms on adaptive and maladaptive workplace outcomes, respectively).

In addition, the model test using the meta‐analysed data afforded estimation of proposed mediation effects that represent key SDT mechanisms according to the model. Specifically, we predicted non‐zero positive effects of need support on adaptive outcomes mediated by need satisfaction and autonomous forms of motivation (H9a–d) and negative indirect effects of need support on maladaptive outcomes, through need satisfaction and autonomous motivation forms (H9e–f). We also predicted non‐zero negative effects of need support on adaptive outcomes through need satisfaction and controlled forms of motivation (H10a–d), and non‐zero positive effects of need support on maladaptive outcomes through need satisfaction and controlled forms of motivation (H10e–f). Finally, we expected these indirect effects to translate to non‐zero positive net or sums of indirect effects of need support on adaptive and maladaptive outcomes through all motivation forms (H11a–f).

With respect to our third aim, we adopted fit‐for‐purpose analytic methods to test our proposed model to address some of the noted limitations of the model test reported in a previous meta‐analysis (Slemp et al. 2018). The advances afforded by our analysis in this regard included extracting data to compute meta‐analytic estimates of all requisite correlations for the matrix required for the model test obviating the need for imputation, reporting indirect effects from mediation analyses derived from the analysis permitting interpretation of their effect size and variability, and adopting the meta‐analytic structural equation modelling approach, which allows for the input matrix to be analysed as a covariance matrix and, in doing so, affords optimal precision in the model parameter and variability estimates (Hagger and Hamilton 2024a; Jak and Cheung 2024).

#### 1.1.3 Analysis of Moderators

Assuming substantive residual heterogeneity in the effects in our model test, our fourth aim was to test the effects of key conceptual study‐level moderators on model effects including work type (public service or for‐profit), employee type (corporate employees, healthcare workers, or teachers), proximity of leader autonomy support (proximal or distal), GDP (top 10 country by GDP or country outside the top 10 countries by GDP), and cultural orientation (collectivist or individualist) of study country of origin. Specifically, we estimated our meta‐analytic structural equation models separately in groups of studies at each level of the moderator with formal comparisons of model effects. As we expected small numbers of studies contributing data to some cells of the input correlation matrix in some moderator groups, we conducted our moderator analyses using a truncated model in which effect size data for key model constructs were aggregated into superordinate categories along conceptual lines.

Specifically, based on prior empirical precedent, we aggregated autonomy, competence, and relatedness need satisfaction constructs into a global need satisfaction construct (see Hagger et al. 2006), and autonomous (e.g., intrinsic motivation, integrated regulation, and identified regulation) and controlled (e.g., introjected and external regulation) forms of motivation into global autonomous and controlled motivation constructs, respectively (see Vallerand and Ratelle 2002). We also collapsed our outcome variables into adaptive and maladaptive workplace outcome categories (see Collie and Martin 2017). The truncated model is presented in Figure 1b. Accordingly, we proposed a set of parallel hypotheses for model effects (HT) for the truncated model, also summarised in our Supporting Information S1 (Table A2, Supporting Information S1: A). Specifically, we hypothesised non‐zero positive direct effects of psychological need support on need satisfaction (HT1); non‐zero positive and negative effects of need satisfaction on autonomous (HT2a) and controlled (HT2b) forms of motivation, respectively; non‐zero positive and negative effects of autonomous and controlled motivation forms on adaptive (HT3a, HT4a) and maladaptive (HT3b, HT4b) outcomes, respectively; and non‐zero residual positive and negative effects need support on adaptive (HT5a) and maladaptive (HT5b) outcomes, respectively. In addition, we hypothesised non‐zero positive and negative indirect effects of need support on adaptive (HT6a) and maladaptive (HT7a) outcomes, respectively, mediated by autonomous motivation forms, with concomitant negative and positive indirect need support effects on adaptive (HT6b) and maladaptive (HT7b) outcomes, respectively, mediated by controlled motivation forms. Analogous with the full model, we also expected these direct effects to translate to net positive and negative sums of indirect effects of need support on adaptive (HT8a) and maladaptive (HT8b) outcomes, respectively, mediated by need support and all forms of motivation.^1^

Hypothesised effects of the moderator variables for our truncated model (HM) are summarised in our Supporting Information S1 (Table A3, Supporting Information S1: A). Focusing on our employee and work type moderator variables, we expected larger effects of need support on need satisfaction, of need satisfaction on autonomous forms of motivation, and of autonomous forms of motivation on adaptive workplace outcomes in studies targeting employees in public service roles (e.g., teachers, healthcare workers; HM1a–c), and employees working in organizations with public service remit (HM2a–c). Our rationale for these predictions was based on the premise that these types of employees may be more autonomously driven by their work than corporate employees and those working for organizations engaged in for‐profit work (Rebitzer and Taylor 2011). We also expected larger effects of autonomous motivation (HM3a), and smaller effects of controlled forms of motivation (HM3b), on adaptive and maladaptive workplace outcomes, respectively, among employees of studies on samples from countries ranked in the global top 10 by GDP. We reasoned that employees living in countries outside the top 10 GDP may feel greater pressure to provide for themselves and their dependants due to greater economic uncertainty, comparatively lower living standards, and less support or ‘safety net’ from their government (Ryan et al. 1999). With respect to cultural orientation, we expected that effect sizes would be similar across studies regardless of cultural group (HM4a–d). Although research has demonstrated some differences in the average levels of autonomous and controlled forms of motivation among employees of nations that tend to endorse individualist and collectivist cultural orientations, these forms of motivation have been shown to contribute equally to adaptive outcomes independent of cultural orientation (Chirkov et al. 2003; Sheldon et al. 2004). Finally, we also expected larger effects of psychological need support on need satisfaction (HM5a), and of need satisfaction on autonomous forms of motivation (HM5b), in studies where leaders were more proximal to their employees (see also Slemp et al. 2018). This is because employees who experience need support firsthand and more frequently are more likely to experience the benefits of need support relative to employees whose leaders are more distal (Cole et al. 2009; Humphreys 2002).

In addition, we also tested effects of our moderator variables on the proposed indirect effects in our model. Based on our hypotheses for the individual model effects, we predicted that the indirect effects of psychological need support on adaptive outcomes mediated by need support and autonomous forms of motivation would be larger in studies targeting those employed in public service roles such as teachers and healthcare workers (HM6), in studies on employees in working in public service organizations (HM7), in studies on samples from countries ranked in the global top 10 by GDP (HM8), and in studies on organizations in which leaders provided proximal autonomy support for their employees (HM10). We hypothesised no differences in the model effects according to country cultural orientation (HM9), as noted in previous research (Sheldon et al. 2004).

## 2 Method

### 2.1 Search Strategy and Study Selection

To identify research items for inclusion in the current analysis, we conducted a systematic search of four electronic databases (‘Web of Science’, ‘Scopus’, ‘PsychINFO’, ‘PubMed’) for articles reporting associations among constructs from SDT (i.e., intrinsic motivation, autonomy) and workplace outcomes (i.e., job performance). Search strings were customised for each database and are reported in Supporting Information S1: B. Our searches were not limited by publication year, language, or publication status. In addition to the database searches, we also conducted manual searches of the reference lists of pertinent theoretical and review articles in the field. Prominent authors were also contacted and asked to supply any unpublished data and requests for data were circulated on the listservs of key scholarly organizations (e.g., Society of Behavioural Medicine, Society for Personality and Social Psychology).

The articles obtained through the initial searches were screened for duplicates by three researchers. After removal of duplicates (*k* = 3059), the researchers screened the remaining (*k* = 8677) articles according to pre‐determined title and abstract screening criteria. The remaining research items (*k* = 536) were then subjected to article full‐text screening against inclusion criteria. To evaluate the inter‐rater reliability of the researchers applying the screening procedures, each researcher screened a sub‐set of the articles against screening criteria. Agreement in screening decisions across raters was high (average Cohen's *κ* = 0.87). Identified disagreements were discussed among the research team for consensus and the screening procedure adjusted or clarified where necessary. Where articles met inclusion criteria but did not report sufficient data for effect size computation, we contacted study authors by email to request the unavailable data. Flow of articles through the screening procedure is presented in a PRISMA‐compliant diagram with an accompanying PRISMA checklist (see Supporting Information S1: C and D). The study protocol and methods were pre‐registered online: https://osf.io/gj37p/.

### 2.2 Inclusion Criteria and Included Studies

Articles were eligible for inclusion in the current analysis if they reported an effect size between at least one measure of a construct from SDT (e.g., psychological need satisfaction, autonomous or controlled forms of motivation, perceived need support) and one measure of a workplace outcome (e.g., job satisfaction, work engagement, burnout) in employees in a workplace context. We defined the workplace context as any occupational, organizational, or employment setting in which individuals provide a service for compensation. For the purposes of the current study, we defined workplace outcomes as any cognitive, affective, or behavioural response or state relevant to employee performance or function in the workplace. Studies were excluded if they adopted qualitative methods or if they were a non‐empirical article (e.g., review, study protocol, commentary). Our screening procedure identified 214 studies eligible for inclusion in the meta‐analysis. Twenty‐two studies were excluded due to insufficient data reported to compute an effect size and could not been sourced from contacting the study author(s), leaving 192 articles eligible for analysis with a total sample size of 93,552. A list of included studies is presented in our Supporting Information S1: E.

### 2.3 Effect Size Data Extraction and Classification of Constructs

#### 2.3.1 Data Extraction

Effect size data for relations between measures of SDT constructs and workplace outcomes were extracted from studies selected for inclusion. The zero‐order correlation coefficient (*r*) was adopted as the effect size metric as a majority of the included studies were correlational in design. Where effect sizes were not expressed as a correlation, an effect size estimate was derived from available data including computed effect sizes (Cohen's *d* or *f*, η^2^), and tests of difference (e.g., *t* and *F*‐ratios, χ^2^ values), and converted to *r* using standard formulae (Borenstein et al. 2009).

#### 2.3.2 Construct Classification

A common issue when pooling effect size estimates among measures of psychological constructs is the extent to which measures used in the studies from which the effect sizes are drawn are equivalent. It is, therefore, important to specify clear definitions of the key constructs involved in the effect size estimates a priori and apply a systematic procedure to ensure that measures are appropriately classified according to their match with theory‐specified construct definitions and rejected as a candidate measure of the construct in cases where the measure deviates substantially from the formal definition. Furthermore, it is important that this procedure is based on the content of the measures used rather than the terms or labels adopted by study authors to refer to the constructs, given noted tendencies for researchers to label constructs with similar content, particularly in terms of their measures, differently, or to label constructs with similar content differently (Hagger 2014; Shaffer et al. 2016). To this end, we ensured that measures of key SDT constructs and the outcomes of interest in the current analysis conformed to our a priori definitions. We developed a ‘construct classification’ procedure consistent with previous meta‐analyses (e.g., Hagger and Hamilton 2021, 2024b) and systematically applied it to the measures of each construct and outcome identified in the studies included in the current analysis. Specifically, the procedure involved (a) developing a set of operational definitions of the key constructs and outcomes including typical measures used from the extant conceptual literature defining these constructs and outcomes together with a review of prior empirical studies measuring them; (b) developing a written procedure and coding spreadsheet outlining the process in which item content from the study measures was extracted, logged on the spreadsheet, compared to the appropriate operational definition; and (c) application of the procedure including documenting the decisions made. The procedure was conducted by a single member of the research team familiar with SDT and trained in systematic review methods. Our validation of the procedure involved a second researcher with identical training applying the procedure to 10% of the coded items, which yielded identical results (Cohen's *κ* = 1.00). The written coding procedure, construct and outcome definitions, and spreadsheet comprising sample items from the measures used in the included studies and their assigned classifications are available online: https://osf.io/gj37p.

##### 2.3.2.1 SDT Constructs

Constructs from SDT included autonomous forms of motivation (intrinsic motivation, integrated regulation, and identified regulation), controlled forms of motivation (introjected regulation, external regulation), satisfaction of the basic needs for autonomy, competence, and relatedness, and basic psychological need or autonomy support. Definitions of study constructs were derived from prior conceptual summaries of SDT and prior studies that have classified each construct (e.g., Deci and Ryan 2000; Ryan and Deci 2000, 2017; Teixeira et al. 2020). These were used as a basis for developing our operational definition and prototype measures of each construct and fed forward into our classification procedure. This also formed criteria for excluding effect size estimates from our analysis in cases where the measures did not conform to the operational definition. Operational definitions and prototype measures for each SDT construct, the measures used to tap these constructs extracted from the included studies, and their final classification based on our procedure are available online: https://osf.io/gj37p.

##### 2.3.2.2 Workplace Outcomes

Included studies reported a substantive and highly variable set of workplace outcomes as dependent variables. For the purposes of the current study, we defined workplace outcomes broadly as any outcome likely to be relevant to employee performance in their job or role in the workplace, or relevant to employee well‐ or ill‐being in the workplace. The most frequently reported outcomes were job performance, job satisfaction, burnout, employee turnover, work engagement, well‐being, absenteeism, work stress, and creativity. Given the breadth of outcomes and the expected pattern of effects that the constructs relating to need satisfaction and motivation quality were expected to exhibit in relation to each based on prior research and our proposed model, we focused our analysis on broad adaptive and maladaptive outcome categories. Adaptive workplace outcomes were defined as outcomes encompassing factors likely to confer a work or well‐being related benefit to the employee, including behavioural work persistence (i.e., work engagement), work quality, and work attitudes, while maladaptive workplace outcomes were defined as outcomes associated with poor employee performance or ill‐being. This was based on prior studies that have made such distinctions and provided a conceptual basis for doing so (e.g., Deci et al. 2017; Slemp et al. 2018). Accordingly, outcomes reported in the included studies were classified into four adaptive outcome categories: job satisfaction, work engagement, job performance, and well‐being.

Analogously, we classified outcomes into two maladaptive outcome categories: burnout and turnover. Our classification procedure in this case, therefore, not only needed to classify the workplace outcome measures into these broad categories, but also to ensure the measures used assessed workplace outcomes with broad relevance to work performance or employee well‐being or ill‐being. Accordingly, we first assessed whether the outcome measure was relevant to performance of well‐being or ill‐being, and, if so, identified the adaptive or maladaptive outcomes category into which it should be classified. Where the measure was not considered relevant, effect size data involving the measure were excluded from our analysis. The procedure was accompanied by a recording scheme to log the definition and sample items of each measure and decisions on their classification, similar to that developed for the SDT construct measures. Of the workplace outcome measures used in the current sample of studies, almost all adopted employee self‐report methods, including single‐ and multiple‐item scales. Each workplace outcome with prototypes of typical measures used to assess them, example measures and items from the measures used in the current study, and their final classification based on our protocol is reported in a spreadsheet available online: https://osf.io/gj37p.

### 2.4 Moderator and Covariate Coding

We coded five candidate moderator variables expected to affect relations among constructs in our proposed model. Two moderators were related to the organizational context in which participant employees in the included studies worked. The first, employee type, referred to the specific job of the participants (e.g., corporate employee, teacher, healthcare worker), while the second, work type, referred to the type of work in which participant employees typically engaged in their organization, either public service or for‐profit. Two moderators related to the country of origin from which the study sample was drawn. Country GDP was used as a proxy measure of national fiscal resources, and cultural orientation referred to whether or not citizens of the sample country of origin generally tended to endorse collectivist or individualist cultural values. The last moderator was proximity of leader autonomy support (see Slemp et al. 2018), in which studies were classified according to whether the targeted workplace leader provided autonomy support to their employees that was proximal (i.e., direct supervisors) or distal (i.e., managers higher in the chain of command). We also coded studies according to key demographic (age and sex) and sampling (study quality and design) characteristics to include as covariates in the analysis. A full summary table of study characteristics including moderator and covariate coding for the included studies is available in our Supporting Information S1: F.

#### 2.4.1 Moderator Variables

##### 2.4.1.1 Organizational Context

The organizations targeted in the included studies were highly diverse including public service organizations (e.g., emergency services—police, ambulance, fire; large companies in various industries—finance, banking, pharmaceutical, insurance, engineering, consulting, manufacturing, newspapers; small independent businesses—beauty shops, restaurants, hotels; government services departments—public hospitals, psychiatric treatment centres, nursing homes, schools, public and private universities; military and armed forces organizations; and non‐profit organizations). This diversity precluded a fine‐grained analysis of specific work context as a candidate moderator due to small numbers of studies in each category. We classified studies along broader organizational lines. Specifically, we coded two organizational context moderator variables: employee type and work type. For the employee type moderator, we coded studies inductively according to the most frequently reported job descriptions for the samples in the included articles. Three major categories emerged: ‘corporate employees’ (*k* = 70), ‘healthcare workers’ (*k* = 35), and ‘teachers’ (*k =* 29). The remaining highly diverse set of vocations, or studies comprising employees from multiple job types, were coded into an indeterminate ‘other’ category (*k* = 58). For the work type moderator, we coded studies according to the primary function of the targeted organization, that is, whether it prioritised public service or owner or shareholder profit. Accordingly, the organizations targeted in the study were classified as ‘public serving’ (*k* = 105; i.e., healthcare, schools, nonprofits) or ‘for‐profit’ (*k* = 65; i.e., banking, private corporations, hospitality) organizations. A small number of studies (*k* = 22) reported samples from multiple organizations or where work type was highly diverse.

##### 2.4.1.2 Country GDP

Another candidate moderator coded was the GDP of the sample country of origin. Study samples were classified as originating from a high GDP country if the country was ranked in the global top 10 countries by GDP as of September 2023 according to US News (2024; *k* = 99) rankings. The remaining study samples were classified as from countries outside the top 10 countries by GDP (*k* = 91), or study samples that comprised participants from countries in both categories (*k* = 2).

##### 2.4.1.3 Cultural Orientation

We coded studies according to whether citizens from the sample country of origin tended to endorse ‘individualist’ (*k* = 139) or ‘collectivist’ (*k* = 48) cultural values. Those from countries typically endorsing both value types were classified as ‘mixed’ (*k* = 5). Coding was based on Hofstede's (2001) cultural dimensions with countries scoring above 50 on the individualism‐collectivism cultural scale ranked as individualist (Slemp et al. 2018, 2024).

##### 2.4.1.4 Proximity of Leader Autonomy Support

We also classified studies according to the proximity of the workplace leader that provided autonomy support to their employees, consistent with Slemp and colleagues' (2018) classification. Studies in which leaders served as direct supervisors to their employees were classified as ‘proximal’ (*k* = 47), while those in which leaders offered less direct support such as those that provided little or no direct supervision of employees were classified as ‘distal’ (*k* = 13). Some articles reported measures of leader autonomy support, but did not specify the source of the support, or did not provide sufficient information on the leader's role. These studies were classified as indeterminate with respect to leader proximity (*k* = 16).

#### 2.4.2 Covariates

##### 2.4.2.1 Demographic Characteristics

Included studies were coded according to sample age and sex distribution. Samples were classified as ‘younger’ aged if the average sample age was less than 40 years with a standard deviation less than 15 (*k* = 105), ‘older’ aged if the average sample age was equal to or greater than 40 years with a standard deviation less than 15 (*k* = 54), and ‘balanced’ in age distribution if the sample age range fell outside these limits (*k* = 33). We also coded studies according to the sex of the sample participants. Following guidelines outlined in similar prior meta‐analyses (e.g., Hamilton et al. 2020), studies were coded as either ‘majority female’ (≥ 75% female; *k* = 55) or ‘majority male’ (≤ 25% female; *k* = 14), with the remaining samples classified as having a ‘balanced’ sex profile (> 25% female and < 75% female; *k* = 123). These variables were included as categorical covariates in our final analyses.

##### 2.4.2.2 Study Quality

We assessed the quality of included studies using the 20‐item Quality Assessment Checklist for Survey Studies in Psychology (Q‐SSP; Protogerou and Hagger 2020). The Q‐SSP provides an assessment of study quality in four domains: introduction, participants, data, and ethics, and yields a quality score out of 20 for each study. The Q‐SSP items and scoring system are presented in out Supporting Information S1: G. Scores for the Q‐SSP ranged from 21% to 89.4%, with a mean score of 60.25% (10.94%). A full summary of quality scores for the included studies is presented in Table H1 in our Supporting Information S1: H. Quality scores were included in our analysis as a continuous covariate.

##### 2.4.2.3 Study Design

All included studies reported correlational data. Studies in which study measures were administered to participants on a single occasion, the majority (*k* = 157), were classified as ‘cross‐sectional’ in design. A small portion of studies administered study measures on multiple occasions and were classified as ‘longitudinal’ in design (*k* = 35). This variable was included as a categorical covariate in subsequent analyses.

### 2.5 Data Analysis

#### 2.5.1 Multilevel Meta‐Analysis of Correlations

We estimated averaged bias‐corrected correlations between constructs from SDT and workplace outcomes using multilevel random‐effects meta‐analysis. Many of the included studies reported multiple effect sizes for the same relationship of interest (e.g., multiple measures of SDT constructs and workplace outcomes, or measures collected on multiple occasions) in the same study. Inclusion of multiple within‐study effect sizes in the same meta‐analysis violates the assumption of effect size independence. The three‐level meta‐analytic model offers an elegant analytic solution to this problem because it explicitly compartmentalises variance in effect sizes between studies and variance between effect sizes within studies and circumvents the problem of data loss when within‐study effect sizes are aggregated (for details see Assink and Wibbelink 2016; Hagger 2022). The analysis yielded averaged point estimates of each correlation across studies corrected for sampling error with variability estimates in the form of 95% confidence intervals and variability estimates (σ^2^) attributed to each level of the analysis, equivalent to the τ^2^ estimate of true variability in the averaged correlation (Cheung 2015). We formally assessed the heterogeneity associated with each correlation using Cochran's *Q*, with an accompanying *I* ^2^ estimate used to represent the specific level of variance in the correlation for each sample.

#### 2.5.2 Meta‐Analytic Structural Equation Models

We tested our proposed model specifying theory‐specified associations between SDT constructs and workplace outcomes using the meta‐analytically synthesised matrix of correlations among theory constructs and outcomes extracted from the included studies (Cheung 2015). Specifically, we first pooled the matrices of correlations among model constructs and the associated variance‐covariance matrix using multilevel multivariate meta‐analysis using Wilson et al. ’s (2016) procedure. The analysis provides a solution to synthesising correlation matrices when each cell of the matrix comprises correlations from different numbers of studies and multiple correlations within each study. It also allows for the adjustment of each correlation for study‐level covariates, in our case, sample age and sex, and study design and quality. We then fit the resulting unadjusted and covariate‐adjusted matrices to the proposed model using meta‐analytic structural equation modelling using Cheung's (2015) MASEM approach, which allows researchers to capitalize on the large sample size of the meta‐analytically pooled data to test existing and unique models (see Hagger and Hamilton 2024a). We utilised multiple model fit indices to assess the goodness of fit of the model with the data: the comparative fit index (CFI) and Tucker‐Lewis index (TLI), the standardized root mean squared residual (SRMR), and the root mean square error of approximation (RMSEA). Values approaching or exceeding 0.950, less than 0.050, and less than 0.080 for the CFI and TLI, SRMSR, and RMSEA, respectively, were considered indicative of acceptable model fit. The non‐nested unadjusted and covariate‐adjusted versions of the models were compared using Akaike's information criterion (AIC) and its Bayesian implementation (BAIC), with the model exhibiting the lowest values in each case taken as the optimal model.

We also assessed the effects of moderator variables on the relations in the proposed model by estimating the proposed model separately in groups of studies at each level of our moderator variables: organizational context (work type and employee type), country GDP, country cultural orientation, and leader autonomy support proximity.^2^ We tested differences in the standardized parameter estimates across the models in each moderator group using the 95% confidence interval about the parameter mean difference (Schenker and Gentleman 2001).

### 2.6 Assessment of Bias

We utilised a panel of bias‐correction methods to evaluate potential for selective reporting bias in the correlations among the constructs and outcomes in the included studies, widely considered indicative of publication bias (Carter et al. 2019). Two sets of methods were used, those based on ‘funnel’ plot of the effect size of interest on an estimate of its precision (e.g., the reciprocal of the study sample size) and those based on selection methods, which model the effect size under different hypothetical conditions of bias.

Methods based on funnel plots aim to detect degree of asymmetry in the funnel plot, a condition suggestive of small‐study bias. Methods adopted were the rank correlation test (Begg and Mazumdar 1994), the ‘trim and fill’ method (Duval and Tweedie 2000), and regression methods (Sterne et al. 2001). A statistically significant rank correlation test and a high number of imputed studies for the ‘trim and fill’ method indicates the presence of bias, with a corrected ‘trimmed and filled’ effect size estimate also offered. For the regression tests, a significant effect of the precision estimate indicates bias, with the intercept providing a corrected estimate of the effect size (see Sterne et al. 2001). We used two versions: the precision effect test (PET), which uses the standard error as the precision estimate, and the precision effect estimate with standard error (PEESE), which uses the square of the standard error as the precision estimate. According to Stanley and Doucouliagos (2014), when the PET estimate of the effect size is not statistically significant, the PET estimate should be retained as the revised effect size, but when the PET estimate is statistically significant, the PEESE estimate should be taken. These tests were implemented using the metafor function in R (Viechtbauer 2010).

The analyses based on selection methods estimate the extent of bias in computed effect sizes by comparing the data model with a selection model, which models certain conditions of bias (Vevea and Hedges 1995). We used the three‐parameter model suggested by Carter et al. (2019) based on Iyengar and Greenhouse's (1988) model. The three‐parameter selection model yields a corrected estimate of the effect size and a likelihood ratio (χ^2^) test of whether the selection model differs from the standard meta‐analytic model, which should be non‐significant in the absence of bias. We also used more recently developed versions of this model, the *p*‐curve (Simonsohn et al. 2014) and *p*‐uniform (van Aert and van Assen 2018) methods, which are based on the distribution of *p*‐values of studies. Distributions of these values should follow characteristic pattern under conditions of high statistical power, deviations from which represent presence of possible selection bias. In the absence of bias, *p*‐curves should be significantly right‐skewed and a non‐significant estimate of curve ‘flatness’. The *p*‐uniform provides a corrected effect size estimate, an estimate of true between‐study variance (τ^2^), and a likelihood‐ratio test of publication bias. We used the weightr (Coburn and Vevea 2019), dmetar (Harrer et al. 2019), and puniform (van Aert 2020) functions in R to compute the three‐parameter selection model, *p*‐curve, and *p*‐uniform* analyses, respectively.

## 3 Results

### 3.1 Meta‐Analysis of Correlations

Averaged bias‐corrected zero‐order correlations among SDT constructs and workplace outcomes are presented in our Supporting Information S1 (Table J1, Supporting Information S1: J). The correlations revealed SDT‐consistent trends in the pattern of correlations among constructs and outcomes and were generally in support of our hypotheses. Specifically, we observed non‐zero positive correlations between autonomous forms of motivation and the basic psychological need satisfaction and psychological need support constructs (*r* range = 0.325 to 0.473, *p*s < 0.001) consistent with our hypotheses (HC1a‐l), and between autonomous motivation forms and adaptive workplace outcomes (work engagement, well‐being, job performance, job satisfaction; *r* range = 0.187 to 0.488, *p*s < 0.001), supporting our hypotheses (HC2a‐l). In addition, we found negative correlations between controlled forms of motivation and basic psychological need and need satisfaction constructs (*r* range = −0.093 to −0.034, *p*s < 0.001), although in some cases these effects were non‐zero and trivial in size, but the general pattern was in generally in accordance with our hypotheses (HC3a‐h). There were, exceptions, however—the introjected regulation construct which was positively associated with need support (*r* = 0.155, *p* = 0.036) and relatedness (*r* = 0.026, *p* = 0.612), and extrinsic motivation/external regulation was positively correlated with need support (*r* = 0.034, *p* = 0.539), although the former two correlations were no different from the null. However, we did not observe the expected non‐zero negative correlations between controlled forms of motivation and adaptive workplace outcomes—in the majority of cases correlations were small in size and positive in sign, and none differed from zero (*r* range = −0.076 to 0.138, *p*s > 0.164), leading us to reject our hypotheses (HC4a‐h). Further, we found non‐zero, negative correlations between autonomous forms of motivation and maladaptive workplace outcomes (burnout, turnover), as hypothesised (HC5a‐f; *r* range = −0.255 to 0.320, *p*s < 0.001), and positive correlations between controlled forms of motivation and these outcomes. The correlation between extrinsic motivation and burnout was positive and non‐zero as we hypothesised (HC6b; *r* = 0.124, *p* < 0.001). However, the correlation between introjected regulation and burnout was non‐zero but, in contrast with our predictions, negative in sign (HC6d; *r* = −0.148, *p* < 0.001). Finally, correlations between controlled forms of motivation and turnover were small and no different from the null and were rejected accordingly (HC6a, HC6c).

### 3.2 Tests of the Proposed Model

#### 3.2.1 Model Fit and Covariate Adjustment

Multilevel meta‐analytic structural equation models testing predictions of our proposed full and truncated models in the full sample, and the truncated model in groups of studies at each level of our moderators, exhibited acceptable fit in each case according to adopted criteria, although each model was associated with substantive heterogeneity (see Tables K1 and K2, Supporting Information S1: K).^3^ AIC and BAIC values suggested minimal differences in model fit across the unadjusted and covariate‐adjusted versions of the model, but the covariate‐adjusted models were marginally superior, so we took the adjusted estimates for our hypothesis tests.

#### 3.2.2 Full Model

**FIGURE 2:**
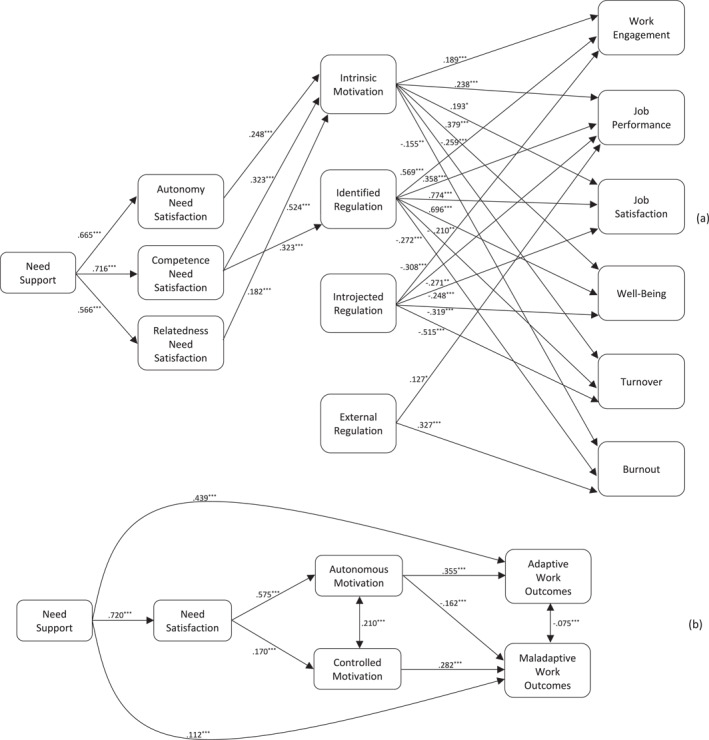
Results of final meta‐analytic structural equation analysis of the proposed model estimated in the full sample indicating non‐zero standardized parameter estimates and tests of significance between need support, basic psychological need satisfaction, motivation regulation, and work outcomes for the full (panel a) and truncated (panel b) models. Only non‐zero parameter estimates and tests of significance reported. The direct effect of need support on maladaptive work outcomes (*β* = −0.234, *p* < 0.001) for the truncated model (panel b) has been omitted for clarity. Covariances among work‐related outcomes for the full model (Panel a) have been omitted for clarity. *p < 0.05 **p < 0.01 ***p < 0.001.

Standardized parameter estimates and 95% confidence intervals for the direct and indirect effects for the full model test adjusted for covariates are presented in Table 1 with direct effects also illustrated in the final model presented in Figure 2 (Panel a). Focusing on model direct effects, we found non‐zero positive effects of psychological need support on autonomy, competence, and relatedness (HF1a‐c; *β* range = 0.566 to 0.716, *p*s < 0.001), and basic psychological need satisfaction constructs on intrinsic motivation (HF2a, HF3a, HF4a; *β* range = 0.182 to 0.323, *p*s < 0.013) consistent with hypotheses, but only found a non‐zero effects of competence need satisfaction on identified regulation (HF3b; *β* = 0.182, *p* < 0.001), with effects of autonomy (HF3a; *β* = 0.168, *p* = 0.054) and relatedness (HF3c; *β* = 0.167, *p* = 0.088) need satisfaction constructs no different from zero. In addition, effects of the need satisfaction constructs on introjected and external regulation constructs were no different from zero (HF2c–d, HF3c, HF4c–d; *β* range = −0.099 to 0.275, *p*s > 0.072), leading us to reject our hypotheses. The effect of competence need satisfaction on external regulation was non‐zero (*β* = 254., *p* = 0.015), but not in the expected direction, leading us to reject our hypothesis (HF3d). In addition, consistent with predictions, we observed non‐zero effects of autonomous motivational regulation constructs (intrinsic motivation, identified regulation) on all workplace outcomes, with positive effects for adaptive outcomes (work engagement, job performance, job satisfaction, well‐being; HF5a–d, HF6a–; *β* range = −0.193 to −0.774, *p*s < 0.023) and negative effects for maladaptive outcomes (turnover, burnout; HF5e‐f, HF6e‐f; *β* range = −0.272 to −0.155, *p*s < 0.032). By contrast, we found non‐zero negative effects of introjected regulation on adaptive outcomes (HF7a–d; *β* range = −0.319 to −0.248, *p*s < 0.035) and non‐zero positive effect of introjected regulation on turnover (H7e; *β* = 0.515, *p* < 0.001), consistent with hypotheses. But the effect of introjected regulation on burnout was no different from the null (H7f; *β* = 0.167, *p* = 0.096). Finally, we observed a positive, non‐zero effect of external regulation on burnout (H8f; *β* = 0.327, *p* < 0.001) and job performance (H8b; *β* = 0.127, *p* = 0.047), although the latter effect was not in the expected direction leading us to reject our hypothesis for this effect. Effects of external regulation on adaptive work outcomes (HF7a–d), and on turnover (HF8e), were no different from the null (*β* range = −0.024 to −0.106, *p*s < 0.068), so we rejected our hypotheses for these effects.

**TABLE 1:** Standardized parameter estimates for direct and indirect effects in the multilevel meta‐analytic structural equation model of the full and truncated models adjusted for covariates.

Effect	*β*	95% CI	
		LL	UL
Full model			
Direct effects			
NSUP→AUT	0.665^***^	0.603	0.726
NSUP→COM	0.716^***^	0.643	0.789
NSUP→REL	0.566^***^	0.491	0.641
AUT→IM	0.248^***^	0.113	0.383
AUT→ID	0.168	−0.003	0.340
AUT→IJ	−0.065	−0.333	0.204
AUT→ER	−0.099	−0.276	0.077
COM→IM	0.323^***^	0.180	0.465
COM→ID	0.524^***^	0.347	0.700
COM→IJ	0.275	−0.024	0.575
COM→ER	0.254^*^	0.050	0.458
REL→IM	0.182^*^	0.039	0.325
REL→ID	0.167	−0.006	0.340
REL→IJ	−0.016	−0.287	0.255
REL→ER	0.076	−0.112	0.264
IM→WE	0.374^***^	0.261	0.487
IM→JP	0.300^***^	0.175	0.418
IM→JS	0.193^*^	0.027	0.359
IM→WB	0.379^***^	0.187	0.571
IM→TU	−0.259^***^	−0.398	−0.119
IM→BU	−0.155^*^	−0.297	−0.014
ID→WE	0.569^***^	0.446	0.692
ID→JP	0.358^***^	0.216	0.499
ID→JS	0.774^***^	0.604	0.944
ID→WB	0.696^***^	0.502	0.891
ID→TU	−0.210^**^	−0.371	−0.050
ID→BU	−0.272^***^	−0.426	−0.118
IJ→WE	−0.308^***^	−0.469	−0.147
IJ→JP	−0.271^**^	−0.444	−0.098
IJ→JS	−0.248^*^	−0.479	−0.016
IJ→WB	−0.319^**^	−0.524	−0.114
IJ→TU	0.515^***^	0.347	0.683
IJ→BU	0.167	−0.030	0.364
ER→WE	0.106	−0.008	0.220
ER→JP	0.127^*^	0.002	0.253
ER→JS	0.002	−0.162	0.166
ER→WB	−0.024	−0.207	0.158
ER→TU	0.068	−0.056	0.193
ER→BU	0.327^***^	0.203	0.450
Indirect effects			
NSUP→NSAT→AM→JP	0.355^***^	0.298	0.413
NSUP→NSAT→AM→JS	0.546^***^	0.486	0.606
NSUP→NSAT→AM→TU	−0.251^***^	−0.309	−0.194
NSUP→NSAT→AM→BU	−0.236^***^	−0.286	−0.185
NSUP→NSAT→AM→WE	0.517^***^	0.463	0.571
NSUP→NSAT→AM→WB	0.594^***^	0.522	0.666
NSUP→NSAT→CM→JP	−0.019	−0.048	0.010
NSUP→NSAT→CM→JS	−0.036^*^	−0.067	−0.004
NSUP→NSAT→CM→TU	0.086^**^	0.034	0.137
NSUP→NSAT→CM→BU	0.076^***^	0.043	0.110
NSUP→NSAT→CM→WE	−0.028	−0.060	0.004
NSUP→NSAT→CM→WB	−0.050^*^	−0.086	−0.014
Sums of indirect effects			
NSUP→NSAT→AM/CM→JP^a^	0.337^***^	0.292	0.381
NSUP→NSAT→AM/CM→JS^b^	0.511^***^	0.467	0.554
NSUP→NSAT→AM/CM→TU^c^	−0.166^***^	−0.205	−0.127
NSUP→NSAT→AM/CM→BU^d^	−0.159^***^	−0.197	−0.121
NSUP→NSAT→AM/CM→WE^e^	0.489^***^	0.451	0.528
NSUP→NSAT→AM/CM→WB^f^	0.544^***^	0.491	0.597
Truncated model			
Direct effects			
NSUP→NSAT	0.720^***^	0.675	0.765
NSUP→AD	0.439^***^	0.396	0.482
NSUP→MAL	−0.161^***^	−0.222	−0.101
NSAT→AM	0.575^***^	0.538	0.612
NSAT→CM	0.170^***^	0.121	0.219
AM→AD	0.355^***^	0.312	0.398
AM→MAL	−0.162^***^	−0.216	−0.108
CM→AD	0.038	−0.002	0.078
CM→MAL	0.282^***^	0.226	0.339
Indirect effects			
NSUP→NSAT→AM→AD	0.147^***^	0.127	0.166
NSUP→NSAT→CM→AD	0.005^*^	0.000	0.009
NSUP→NSAT→AM→MAL	−0.067^***^	−0.090	−0.044
NSUP→NSAT→CM→MAL	0.035^***^	0.021	0.048
Sums of indirect effects			
NSUP→NSAT→CM/AM→AD^a^	0.152^***^	0.133	0.171
NSUP→NSAT→CM/AM→MAL^b^	−0.032^**^	−0.056	−0.009
Total effects			
NSUP→AD^c^	0.591^***^	0.558	0.623
NSUP→MAL^d^	−0.194^***^	−0.239	−0.149

*Note:* Model parameters are adjusted for the following covariates: age, sex, study quality, and study design.

Abbreviations: 95% CI = 95% confidence interval of parameter estimate; *β* = Standardized path coefficient; AD = adaptive workplace outcomes collapsed across job performance, job satisfaction, work engagement, and well‐being outcome variables for the truncated model only; AM = Autonomous forms of motivation collapsed across intrinsic motivation and identified regulation constructs for the truncated model only; AU = autonomy need satisfaction; BU = burnout; CM = controlled forms of motivation collapsed across intrinsic motivation and introjected and external regulation constructs for the truncated model only; COM = competence need satisfaction; ER = External regulation; ID = identified regulation; IM = intrinsic motivation; IJ = introjected regulation; JP = job performance; JS = job satisfaction; LL = Lower limit of 95% CI; MAL = maladaptive workplace outcomes collapsed across turnover and burnout outcomes for the truncated model only; NSAT = all need satisfaction constructs (full model only) or need satisfaction collapsed across autonomy, competence, and relatedness constructs (truncated model only); NSUP = need support; REL = relatedness need satisfaction; WB = well‐being; WE = work engagement; TU = turnover; UL = upper limit of 95% CI.

^a^ Sum of indirect effects of need support on job performance through all variables.

^b^ Sum of indirect effects of need support on job satisfaction through all variables.

^c^ Sum of indirect effects of need support on turnover through all variables.

^d^ Sum of indirect effects of need support on burnout through all variables.

^e^ Sum of indirect effects of need support on work engagement through all variables.

^f^ Sum of indirect effects of need support on well‐being through all variables.

^*^ *p* < 0.05.

^**^ *p* < 0.01.

^***^ *p* < 0.001.

Focusing on indirect effects, consistent with hypotheses, we observed non‐zero positive specific indirect effects for perceived need support on adaptive workplace outcomes through need satisfaction constructs and autonomous forms of motivation (HF9a–d; *β* range = 0.355 to 0.594, *p*s < 0.001), which was also met by non‐zero positive the sums of indirect effects through all motivation forms (HF11a–d; *β* range = 0.337 to 0.554, *p*s < 0.001). Similarly, we found non‐zero, negative specific indirect effects of need support on turnover (HF9e; *β* = −0.251, *p* < 0.001) and burnout (HF9f; *β* = −0.236, *p* < 0.001) through the same mediators, also reflected in non‐zero, negative sums of indirect effects on these outcomes (HF11e, *β* = −0.166, *p* < 0.001; HF11f, *β* = −0.159, *p* < 0.001), as hypothesised. We also observed positive non‐zero specific indirect effects of need support on turnover (HF10e; *β* = 0.086, *p* = 0.001) and burnout (HF10f; *β* = 0.076, *p* < 0.001) through controlled forms of motivation as well as negative non‐zero specific indirect effects on job satisfaction (HF10c; *β* = −0.036, *p* = 0.025) and well‐being (HF10d; *β* = −0.050, *p* = 0.007). Taken together, these equated to negative, non‐zero sums of indirect effects of need support on turnover (HF11e; *β* = −0.166, *p* < 0.001) and burnout (HF11f; *β* = −0.159, *p* < 0.001).

#### 3.2.3 Truncated Model

We estimated a second multilevel meta‐analytic structural equation model for a truncated model in which conceptually related SDT constructs and workplace outcomes were aggregated into higher order constructs. This model was purposed to enable subsequent tests of moderator effects on model effects and ensured we had sufficient data in each cell of the input analysis matrix at each level of the moderator. As a prelude to the moderator analyses, it was necessary to estimate this model in the full sample of studies. Parameter estimates and 95% confidence intervals for this model are presented in Table 1 with direct effects illustrated in Figure 2 (Panel b).^4^ In general, findings mirrored the patterns of effects observed in the full model. In terms of direct effects, consistent with our hypotheses, we found non‐zero positive effects of perceived need support on need satisfaction (HT1; *β* = 0.720, *p* < 0.001), of need satisfaction on autonomous motivation (HT2a; *β* = 0.575, *p* < 0.001), and of autonomous motivation on adaptive workplace outcomes (HT3a; *β* = 0.355, *p* < 0.001). In addition, we found a positive effect of need satisfaction on controlled motivation (HT2b; *β* = 0.170, *p* < 0.001), but not in the predicted direction, so this hypothesis was rejected. Analogously, we observed a non‐zero negative effect of autonomous motivation on maladaptive outcomes (HT3b; *β* = −0.162, *p* < 0.001). In addition, we observed a non‐zero positive effect of controlled motivation on maladaptive outcomes, as predicted (HT4b; *β* = 0.282, *p* < 0.001), but the effect on adaptive outcomes was no different from the null (HT4a; *β* = 0.038, *p* = 0.062). Finally, we observed a positive direct effect of need support on adaptive outcomes (HT5a; *β* = 0.439, *p* < 0.001) and a negative effect on maladaptive outcomes (HT5b; *β* = −0.161, *p* < 0.001), as predicted.

Focusing on the indirect effects, consistent with hypotheses, we observed non‐zero specific indirect effects of perceived need support on adaptive (HT6a; *β* = 0.147, *p* < 0.001) and maladaptive (HT7a; *β* = −0.067, *p* < 0.001) workplace outcomes through need satisfaction and autonomous motivation that were positive and negative in sign, respectively. In addition, we found non‐zero specific indirect effects of need support on adaptive (HT6b; *β* = 0.005, *p* = 0.029) and maladaptive (HT7a; *β* = 0.035, *p* < 0.001) through need satisfaction and controlled motivation forms, although the effect on adaptive outcomes was positive when it was hypothesised to be negative leading us to reject this hypothesis. These indirect effects were corroborated by the predicted non‐zero positive sums of indirect effects on adaptive (HT8a; *β* = 0.152, *p* < 0.001), and a non‐zero negative effect on maladaptive (HT8b; *β* = −0.032, *p* = 0.008) outcomes, mediated by the need satisfaction and autonomous and controlled motivation constructs that were also positive and negative in sign, respectively. Finally, along with the direct effects of need support on outcomes, we observed total effects of need support on adaptive (HT9a; *β* = 0.591, *p* < 0.001) and maladaptive (HT9b; *β* = −0.194, *p* < 0.001) workplace outcomes consistent with this pattern.

#### 3.2.4 Analysis of Moderators

We tested for differences in truncated model parameter estimates according to our moderator variables (work type, employee type, proximity of leader autonomy support, and country GDP and cultural orientation) by estimating the model in sets of studies at each level of the moderator. Model fit statistics for each moderator model are presented in Table K1 (Supporting Information S1: K) and results of the model tests are presented in Tables M1 to M11 of our Supporting Information S1: M. We observed differences in the effects of need support on need satisfaction, and of need satisfaction on autonomous motivation, according to our work type and employee type moderators (HM1a–b, HM2a–b), and for the effect of autonomous motivation on adaptive workplace outcomes in employees engaged in corporate roles compared with ‘other’ roles (HM1c), although the sign for these hypothesised effects in each case was inconsistent with our predictions—larger effects in studies on employees working in public service roles, especially when compared with healthcare workers and ‘other’ roles, and in corporate organizations were expected in all cases leading us to reject these hypotheses. We also found marginally larger effects of autonomous motivation on adaptive workplace outcomes in studies on samples from countries ranked in the GDP top 10 compared to studies on countries outside the top 10, as hypothesised (HM3c), but no reduced effect of controlled motivational forms on maladaptive outcomes, leading us to reject our hypothesis (HM3b). Consistent with our hypotheses, we generally observed no differences in the effects of autonomous motivational forms of adaptive and maladaptive outcomes according to the cultural orientation of the sample country of origin (HM4a–b), although it should be noted that there are larger effects of controlled motivation on maladaptive outcomes in studies in countries with an individualist orientation, so this hypothesis was rejected (H4c). Finally, we found no differences in the effects of need support on need satisfaction, and in the effects of need satisfaction on autonomous motivation forms, in samples with leaders with close proximity to their employees relative to those with a distal supervisor proximity, leading us to reject our hypotheses for this moderator (HM5a–b).

Focusing on our hypotheses for the indirect effects, for the employee type moderator, we observed larger positive sums of indirect and total effects of need support on adaptive outcomes in studies on corporate employees relative to studies on teachers, healthcare workers, and ‘other’ employees, findings that were inconsistent with our hypothesis (HM6). These effects were attributable to larger positive direct effects of need satisfaction on autonomous motivation and need satisfaction. We also observed some differences in the sums of indirect and total effects on outcomes across healthcare, teacher, and ‘other’ employees, but these differences were less consistent and much smaller in size relative to those observed for these employee types and corporate employees.

For the work type moderator, we found larger positive sums of indirect and total effects of need support on adaptive workplace outcomes in studies on for‐profit work organizations compared to public service work environments, effects that were again inconsistent with our hypothesis (HM7). Although not hypothesised, we also observed larger negative indirect and total effects of need support on maladaptive workplace outcomes, in studies on for‐profit relative to public service environments. These effects were mainly due to larger positive direct effects of need satisfaction on autonomous forms of motivation and need support on adaptive outcomes in for‐profit environments, and larger negative effects of need satisfaction on controlled motivation and need support on maladaptive outcomes in public service work environments.

Focusing on the country GDP and cultural orientation moderators, as predicted we observed larger positive sums of indirect and total effects of need support on adaptive outcomes in studies on samples from top 10 GDP countries relative to those on countries outside the top 10 (HM8). These effects were mainly due to larger positive direct effects of need satisfaction on autonomous motivation, need support on need satisfaction, and need support on adaptive outcomes. We also found larger positive sums of indirect effects and total effects of need support on adaptive outcomes in countries endorsing individualist cultural orientation relative to those endorsing a collectivist orientation, contrary to our predictions (HM9). These effects tended to be driven by larger direct effects for need satisfaction on autonomous motivation, need support on need satisfaction, and need support on adaptive outcomes.

Finally, we observed few differences in indirect and direct effects for leader proximity, leading us to reject our hypothesis for this moderator (HM10). It should be noted, however, that there was a markedly larger non‐zero negative effect of need support on maladaptive outcomes in studies on employees whose leaders offered proximal autonomy support compared with studies on employees with leaders offering distal or ‘indeterminate’ support, although this effect was not hypothesised.

### 3.3 Assessment of Publication Bias

Our panel of analyses to assess publication bias indicated limited evidence for systematic bias in our averaged correlations. However, it should be noted that many of the bias detection methods available do not necessarily yield precise results under conditions of high heterogeneity as was the case for the correlations in the present study. Full bias analysis results are presented in Table N1 in our Supporting Information S1: N.

## 4 Discussion

In the present study we aimed to synthesise relationships among constructs from SDT (e.g., basic psychological need support, basic psychological need satisfaction, motivational forms) and outcomes (e.g., job performance and satisfaction, well‐being, burnout, job turnover) in workplace contexts across the extant research using meta‐analysis. In addition, we aimed to use the synthesised effect sizes from the meta‐analysis to test a process model based on SDT and prior research (e.g., Ryan et al. 2008; Slemp et al. 2018; Williams et al. 2004) in which effects of perceived psychological need support constructs on workplace outcomes were mediated by psychological need satisfaction and forms of motivation (see Figure 1). Finally, we also aimed to examine effects of salient moderators (i.e., work and employee type, country resources and cultural orientation, leader autonomy support) on the proposed effects in the model. In the next sections we provide a detailed discussion of the conceptual and practical implications of prominent findings arising from our analysis for theorists and researchers with an interest in the motivational determinants of employees' workplace outcomes, and leaders and interventionists in organizations and professional practice interested in improving work function and well‐being among employees. In addition, we offer insight into how our analysis potentially progresses current knowledge on SDT applications in workplace outcomes and identify potential avenues for future research that elucidates the motivational factors and associated mechanisms linked to better workplace outcomes and well‐being among employees.

### 4.1 Correlational Analysis

Our analysis yielded patterns of averaged zero‐order correlations among SDT constructs (i.e., psychological need support, need satisfaction, autonomous and controlled forms of motivation and adaptive and maladaptive outcomes) that were largely consistent with theory predictions and prior research in workplace (Coxen et al. 2021; Messmann et al. 2022; Slemp et al. 2018; Van den Broeck et al. 2016; van Hooff and van Hooft 2017; Wörtler et al. 2020) and other contexts (e.g., health; Ng et al. 2012; education; Niemiec and Ryan 2009). As expected, employees reporting support for basic psychological needs tended to report higher levels of autonomous forms of motivation (HC1d, HC1h, HC1l), express greater need satisfaction (Hc1a–c, HC1e–g, HC1i–k), and report adaptive work outcomes (HC2a–l), and were less likely to experience turnover (HC5a, HC5c, HC55e) and burnout (HC5b, HC5d, HC55f). In addition, the pattern of correlations between controlled forms of motivation and the psychological need support and need satisfaction constructs was negative and generally in accordance with our hypotheses (HC3a–h) and prior research in workplace and other contexts. Individuals citing better support for their needs from their workplace leaders were more likely to report that their needs were being met and to experience self‐determined reasons for their work tasks, findings that align well with prior research in the workplace and other contexts.

The only pattern of effects inconsistent with our hypotheses involved correlations between the controlled motivation construct and adaptive outcomes—correlations between controlled forms of motivation and adaptive workplace outcomes were no different from the null (HC4a–h). These findings are incongruent with prior findings indicating that workers citing controlled reasons for workplace task performance tend to report lower work engagement and job satisfaction (Li et al. 2015; Roche and Haar 2019). Our findings suggest that externally referenced reasons may not be as detrimental to adaptive workplace outcomes motivation for workers when considered more broadly across the available data, particularly for outcomes such as well‐being. Another possibility, given the substantive heterogeneity in the findings, may be that extrinsic motivation effects on these outcomes may be moderated by specific workplace conditions that vary across organizations, such as conditions that may reduce conditions that lead to the thwarting of employees' needs or impinge on their autonomous rationales for work tasks. For instance, the treatment of workplace pay structure, renumeration, or bonus payments by leaders may be a salient moderator. Supervisors and other workplace leaders may, e.g., place less emphasis on employee pay or other external rewards and instead focus on self‐determined rationales for workplace tasks. This lower emphasis may negate the extent to which employees' externally referenced motives are pertinent to workplace outcomes. Measurement artefacts may also be a possible reason for these findings—studies measuring extrinsic motivation as a generalised construct may have referred to forms of regulation that varied in the extent to which they were externally‐referenced (e.g., introjected or identified regulation), which may reflect external continencies that are partially internalized—a potential confound. Further, there is also the suggestion that such measures may have been more generalised in focus, which may have introduced additional method variance to the observed averaged effect sizes.

### 4.2 Test of the Proposed Model

Our model test corroborated patterns of correlations among SDT constructs and workplace outcomes and, importantly, extended them to indicate the unique effects of each construct on other constructs and outcomes when included in parallel with effects of other theory constructs, an important advance given that these constructs are likely to share variance (Chatzisarantis et al. 2003). Most important, however, was the confirmation of the proposed effects specified in the model consistent with Ryan et al. ’s (2008) original proposal based on SDT and prior primary (Galletta et al. 2019) and meta‐analytic tests of the model in the workplace (Slemp et al. 2018) and similar contexts (e.g., Ng et al. 2012). These hypothesis‐ and theory‐consistent effects included expected positive patterns of direct effects: effects of perceived need support on need satisfaction constructs (autonomy, competence, and relatedness; HF1a‐c); of need satisfaction constructs on autonomous motivation forms (intrinsic motivation and identified regulation; HF2–4a, HF 3b); and of autonomous forms of motivation on adaptive workplace and health outcomes (work engagement, job satisfaction, job performance, well‐being; HF5a–d, HF6a–d). In addition, this pattern of findings was replicated in the truncated model using aggregated theory constructs and workplace outcomes, including need support‐need satisfaction (HT1), need satisfaction‐autonomous motivation (HT2a), and autonomous motivation‐adaptive workplace outcome (HT3a) effects.

Most important, however, was the hypothesis‐consistent indirect effects of need support on adaptive workplace outcomes through need satisfaction and motivation forms, with indirect effects direct through need support and autonomous motivation forms particularly prominent (HF9a–d), also reflected in the sums of indirect effects (HF11a–d). We also noted negative specific and sums of indirect effects of need support on these maladaptive outcomes through autonomous motivation forms (HF9e–f, HF10e–f). This pattern was generally mirrored in the truncated model in which motivational and outcomes constructs were aggregated (HT6a, HT7a, HT8a). Taken together, these findings indicate that employees who perceive their workplace leaders support their basic needs are more likely to report adaptive outcomes like job engagement and satisfaction, and less likely to experience maladaptive outcomes like burnout and turnover. The findings implicate need satisfaction and autonomous motivational forms as a key mechanism by which employees' experience of need supportive behaviours and strategies from workplace leaders relate to workplace outcomes, consistent with SDT. That said, model effects were associated with considerable heterogeneity across studies, so while we could conclude that the proposed effects were viable based on currently available data, they are likely subject to moderation by extraneous variables—which provided impetus for our analysis of moderators, an issue to which we return later in the discussion.

Beyond corroborating the theory‐stipulated pattern of effects of the model reported in prior meta‐analytic tests (Slemp et al. 2018), our analysis extends previous findings by explicitly reporting the size and variability of the indirect effects as well as encompassing need support from multiple sources, including managers, colleagues, and work targets (i.e., students), and multiple forms of motivation. Methodologically, the current analysis also advances prior research because sufficient effect size data were extracted from available studies to obviate the need for imputation (see Hagger and Hamilton 2024a). We also used a fit‐for‐purpose analytic method to estimate the model as a covariance matrix, which yielded precise variability estimates (Jak and Cheung, 2024). Overall, these effects provide the most comprehensive and robust tests of the proposed theory‐stipulated pattern of direct and indirect effects in the model in this context to date.

### 4.3 Moderator Tests

Given the substantive residual heterogeneity in model effects between SDT constructs and workplace outcomes, we tested for differences in model effects according to our specified moderator variables. Prominent findings were for the effects of employee and work type moderators, although findings ran contrary to our predictions. Specifically, we found that need support‐need satisfaction and need satisfaction‐autonomous motivation direct effects, and indirect effects of need support on adaptive outcomes mediated by need satisfaction and autonomous motivation, were larger in corporate employees compared to employees in public service roles (e.g., teachers, healthcare workers). These effects were mirrored for the work type moderator: the need support‐need satisfaction and need satisfaction‐autonomous motivation direct effects, and indirect effects of these constructs on adaptive outcomes through the motivational constructs, were larger in studies on employees working in for‐profit work contexts compared to those working in public service work contexts. Accordingly, we rejected our hypotheses for these moderators (HM1a–c, HM2a–c, HM6, HM7), which were based on our rationale that individuals working in public service contexts and roles would be more likely to interpret employers as more supportive of their internalized goals and psychological needs than those with a greater emphasis on externally‐referenced outcomes, namely, making profit.

To speculate, the counter‐expectation pattern of effects may be due to workers in corporate, for‐profit contexts and roles more likely experiencing supervision by workplace leaders that have been trained under well‐funded, highly‐evolved employee development programs that emphasise employee‐focus and use of autonomy‐supportive behaviours, such as autonomy support training programs (Cheon and Reeve 2013; Hagger et al. 2020; Reeve and Cheon 2020). By contrast, public‐facing organizations may have less capacity to invest in such programs, or such programs may be unevenly applied. Alternatively, these indirect effects may be larger in studies on employees in for‐profit roles and contexts because employees in these organizations may be less constrained by rules and regulations that are perceived as obstacles to task progress than those in public‐service roles and contexts. For example, public‐serving schools or hospitals are periodically subject to federal and state audits and other regulatory obligations, which may limit organizational capacity to invest in training initiatives such as those that support psychological needs. In addition, employees in service‐oriented roles like teachers and healthcare workers may begin their jobs more autonomously motivated (Rebitzer and Taylor 2011) and, therefore, attach higher value to need support due to the potential for increased internalisation which may relate to their motivation and, ultimately, outcomes when those needs are potentially thwarted.

We also found larger negative indirect and total effects of need support on maladaptive outcomes in public service contexts, and among teachers and healthcare workers, compared to for‐profit contexts and corporate employees. This finding was not hypothesised so should be considered an auxiliary finding and, therefore, exploratory. However, the effect suggests that leaders in public serving organizations that offer autonomy support afford greater protection to their subordinates from deleterious workplace outcomes than those in for‐profit corporations (Haski‐Leventhal et al. 2019; West et al. 2018). One speculative reason for this may be that employees in public‐serving contexts, and those in professions like teaching and healthcare work, have greater potential to experience maladaptive outcomes like burnout and turnover than corporate employees (e.g., Dyrbye et al. 2014). This may be due to fewer resources and greater workload, and need support offered by management in these contexts may mitigate this. We look to future research that posts a formal, a priori hypothesis based on this finding to offer potential corroboration observed here.

With respect to our country GDP moderator tests, the larger positive direct effect of autonomous motivation on adaptive workplace outcomes in studies originating from top‐10 ranked countries by GDP was consistent with our hypothesis (HM3c), and was mirrored in the sums of indirect effects, and total effects, of need support on these outcomes through need satisfaction and autonomous motivation (HM8). Workers from countries with fewer resources may feel increased pressure to provide for themselves and their families due to limited federal resources or greater economic uncertainty consistent with explanations offered elsewhere (Ryan et al. 1999). We also saw a larger negative total effect of need support on maladaptive outcomes in studies originating in countries outside of the top 10 ranked countries by GDP. This may indicate that autonomy support offered in the workplace may offer a degree of ‘protection’ for workers from experiencing maladaptive outcomes like burnout and higher turnover in countries with lower available resources and governmental support (Martela et al. 2023).

Although it is notable that we found no differences in direct effects of autonomous motivation forms on outcomes in our model according to country cultural orientation, consistent with our hypotheses (HM4a‐b), indirect and total effects of need support on adaptive outcomes mediated by need satisfaction and autonomous motivation, were larger in countries that tend to endorse individualist cultural values compared to those endorsing collectivist values, effects which ran contrary to our prediction (HM9). These findings are also in contrast with those from prior studies that have generally indicated equivalence in these patterns of effect across national groups based on cultural orientation (Chirkov et al. 2003; Sheldon et al. 2004), and with the findings of recent analysis in multiple contexts (Slemp et al. 2024). Again, we speculate that organization‐level workplace support may have greater salience than cultural values and may be confounded by national resource availability—countries that tended to endorse individualist cultural values are also those that tend to be in the top 10 by GDP, and may, therefore, have greater budgets to train their managers and leaders with autonomy‐support programs than those in lower GDP countries.

We also tested the moderating effect of the proximity of leader autonomy support on model effects, consistent with a prior meta‐analysis (Slemp et al. 2018). As there were too few studies in our sample conducted in organizations offering autonomy support from leaders (e.g., managers, supervisors) that were distal to their employees, we compared studies conducted in organizations in which leaders' autonomy support was proximal to their employees to studies in organizations in which such supports were either distal or in which the proximity of the leader could not be determined. Patterns of associations between the two groups were largely consistent, contrary to our expectations (HM10). Although not hypothesized, the larger negative total effect of need support on maladaptive outcomes in studies on organizations in which autonomy support was offered by leaders in close proximity to their employees, suggested that workers employed under leaders offering proximal support (e.g., direct managers and supervisors) report fewer maladaptive outcomes (Cole et al. 2009; Humphreys 2002). Again, this may be because greater exposure to autonomy‐supportive structures and behaviours from leaders increase the likelihood that such practices filter through marginal increases on autonomous reasons for performing work and offer a level of protection from maladaptive outcomes (Hagger et al. 2020; Reeve and Cheon 2020).

### 4.4 Implications for Practice

From a practical perspective, our current findings provide preliminary evidence to signal the potential of initiatives and interventions instigated by workplace leaders (e.g., managers, supervisors) that comprise autonomy‐supportive techniques and target change in perceived need support and autonomous forms of motivation in workplace contexts to promote adaptive workplace outcomes and well‐being and minimise stress‐related outcomes in employees. However, we must emphasise that we are reluctant to make specific recommendations for practice based on current findings given the limitation that the current analysis is confined to studies that were, or were treated as, correlational in design. While these data offer potential means to indicate a necessary condition for causal effects (for a discussion see Dul 2016), they do not offer a strong basis for the inference of causal effects. As a consequence, subsequent studies adopting randomized‐controlled designs (e.g., experimental or intervention designs) that test the efficacy of workplace leaders' application of techniques or strategies targeting change in perceived need support and autonomous forms of motivation (e.g., adopting a collaborative employee‐focused work climate, involving employees in decision making, assisting employees in setting autonomous goals, adopting non‐controlling need‐supportive language when communicating), consistent with current findings and autonomy supportive training programs (see Reeve and Cheon 2020) should be considered a priority. Such studies would be expected to yield evidence that provides a better basis for causal inference as well as the potential efficacy of these techniques in changing salience workplace outcomes.

Alongside this, it would also be important to confirm in studies adopting randomized research designs that interventions featuring the adoption and display of autonomy supportive techniques by workplace leaders have greater efficacy in groups in which effects of need support on motivational and workplace outcomes were observed to be larger according to current findings. For example, are there autonomy‐supportive strategies that workplace leaders could emphasise or double‐down on to enhance autonomous motivation in employees engaged in public service roles or in employees from countries outside the GDP top 10 relative to their counterparts in for‐profit roles or countries in the GDP top 10? Confirmation of such effects may pave the way for the adoption of intervention strategies that may address the deficits among target populations revealed in our moderator analyses.

### 4.5 Contribution, Limitations, and Avenues for Future Research

The present study provided robust estimates of averaged bias‐corrected relations between constructs from SDT, including basic psychological need support and satisfaction, and autonomous and controlled forms of motivation, and workplace outcomes across the extant literature, and associated variability estimates, using meta‐analysis (Deci et al. 2017; Galletta et al. 2019; Gillet et al. 2013; Slemp et al. 2018; van Hooff and van Hooft 2017). It builds on and advances on prior meta‐analyses by affording a comprehensive test of a process model specifying unique effects of SDT constructs on workplace outcomes and a theory‐based mediation mechanism by which the constructs relate to workplace outcomes using meta‐analytically synthesised data. In addition, our analysis also tested the effects of a series of moderators on the model effects. These model tests not only provide support for the proposed mediation of the effect of perceived need support on workplace outcomes by SDT need satisfaction and forms of motivation as proposed in other research on the theory (Ng et al. 2012; Ryan et al. 2008), but provides insight into the moderating effects of variables representing key context‐ and sample‐related conditions that potentially account for observed variability in model effects. Finally, the analysis addresses some of the shortcomings of prior meta‐analyses such as estimating a model based entirely on a synthesis of available data without the need for imputation and adopting fit‐for‐purpose MASEM procedures for our model tests that circumvent the problem of sample size selection.

The advances, novel contributions, and resolution of prior methodological shortcomings offered in the current study notwithstanding, findings should be interpreted in light of several limitations. First, few included studies reported measuring integrated regulation, a specific form of autonomous motivation in SDT, which precluded estimation of separate averaged correlations of this motivational form with other SDT constructs and workplace outcomes in our analysis. Measures of integrated regulation were therefore aggregated together with measures of intrinsic motivation to include these data in our analysis. Researchers should consider adopting measures that encompass the full complement of motivational regulations when conducting research testing SDT predictions in the workplace, such as the Motivation at Work Scale (Gagné et al. 2010) or the Work Extrinsic and Intrinsic Motivation Scale (Tremblay et al. 2009), which include integrated regulation. In addition, few studies were conducted in organizations in which leaders provided distal autonomy support for their employees, that is, leaders not directly involved with employee supervision. More studies on these types of leader may enable a more comprehensive test of effects of the proximity of autonomy support moderator on model effects.

Another notable limitation is that included studies exclusively adopted correlational designs. This limitation precludes causal inferences in the effect size estimates from our proposed model tests. Our model tests, therefore, provide one possible sequence of effects with other well‐fitting models possible, so causation in the proposed effects is inferred from theory alone rather than the data. To advance this line of research, theorists and researchers applying SDT in workplace contexts should consider adopting experimental or interventions study designs across multiple workplace settings (e.g., corporate, school, healthcare, hybrid and remote work settings; McAnally and Hagger 2024). Further, longitudinal study designs, especially recent implementations of cross‐lagged panel designs which allow for the test of reciprocal relations and control for intraindividual stability (Orth et al. 2021), may assist in resolving direction in model effects and may permit tests of reciprocity in effects meta‐analytically consistent with previous research in other fields (e.g., Chan et al. 2020; Hagger and Hamilton 2024b).

Taken together, the current analysis extends past research on SDT in the workplace by providing an extensive and inclusive summary of relations among theory constructs and workplace outcomes and associated variability across the extant research including the model tests. From the perspective of theorists and researchers, our tests of the proposed model provide a possible expected range of effect sizes that they might plausibly expect when conducting studies applying SDT to identify unique motivational predictors of outcomes in workplace contexts. The findings also provide some evidence on factors that may account for the observed variability in those model effects through our moderator tests. From the perspective of intervention and application, the current research may also provide preliminary evidence of potential motivation‐related constructs that could serve as targets in experimental and intervention research aimed at promoting forms of motivation that may foster adaptive outcomes such as need support and satisfaction. Given the correlational designs and concerns over causal inferences noted earlier, we are loathe to definitively advocate in favour of interventions or organization change based solely on the current fundings (Hagger et al. 2020). Instead, we prefer to view current findings as a potential signal of the proposed effects and as a basis for further research utilising designs that may provide causal verification of these effects, such as experiments or interventions. Such research, for example, may target employee need satisfaction change through workplace leaders' autonomy supportive strategies (Deci et al. 1989; Hardré and Reeve 2009). As research adopting such designs accumulates, researchers would be able to draw more definitive causal conclusions on the effects of SDT constructs on workplace outcomes in subsequent research syntheses.

## Funding

The authors have nothing to report.

## Conflicts of Interest

The authors declare no conflicts of interest.

## Supporting information


Supporting Information S1

## Data Availability

The data that support the findings of this study are openly available in Open Science Framework at https://osf.io/gj37p/.

## Endnotes

^1^
Given we also included direct need support effects on adaptive and maladaptive outcomes, we also hypothesized non‐zero net total positive and negative effects of need satisfaction on adaptive (HT8a) and maladaptive (HT8b) outcomes, respectively, mediated by all forms of motivation with the direct need satisfaction effect.

^2^
For completion, we also conducted a supplementary moderator analysis examining the effects of our moderator variables on each averaged zero‐order correlation among constructs from our multilevel meta‐analysis using meta‐regression. We report this analysis in our Supporting Information S1 (see Supporting Information S1: I, with meta‐regression results presented alongside the zero‐order correlation analysis in Table J1, Supporting Information S1: J), as this analysis is superseded by the analysis of moderators of model effects.

^3^
Model‐level variability and heterogeneity estimates and averaged zero‐order correlations with confidence intervals from the multivariate meta‐analysis of correlations among study constructs, unadjusted and adjusted for covariates, conducted in first stage of our MASEM analysis are presented in Tables K2 and K3, respectively (Supporting Information S1: K). A full breakdown of the parameter estimates and 95% confidence intervals for the full model unadjusted for covariates with accompanying adjusted model values for comparison are presented in Table L1 (Supporting Information S1: L). The table also reports estimates of the covariances among independent variables in the proposed model.

^4^
A full breakdown of the parameter estimates and 95% confidence intervals for the truncated model unadjusted for covariates with accompanying adjusted model values for comparison are presented in Table L2, in our Supporting Information S1: L. The table also reports estimates of the covariances among independent variables in the proposed model.
