# Supplementary material for: Self‐Determination Theory and Workplace Outcomes: A Meta‐Analysis
Source: Stress Health. 2026 Feb 10;42(1):e70151. doi: 10.1002/smi.70151 (PMC12887546; doi:10.1002/smi.70151)
Supplement: Supplementary file 1 — Supporting Information S1 [file SMI-42-e70151-s001.docx]

Supplement A

**Table A1**

*Hypothesized Correlations Among Self-Determination Theory Constructs and Workplace Outcomes*

| Hypothesis | Correlation | Expected sign |
| --- | --- | --- |
| HC1a | IM↔AUT | + |
| HC1b | IM↔COM | + |
| HC1c | IM↔REL | + |
| HC1d | IM↔NSUP | + |
| HC1e | IG↔AUT | + |
| HC1f | IG↔COM | + |
| HC1g | IG↔REL | + |
| HC1h | IG↔NSUP | + |
| HC1i | ID↔AUT | + |
| HC1j | ID↔COM | + |
| HC1k | ID↔REL | + |
| HC1l | ID↔NSUP | + |
| HC2a | IM↔WE | + |
| HC2b | IM↔JP | + |
| HC2c | IM↔JS | + |
| HC2d | IM↔WB | + |
| HC2e | IG↔WE | + |
| HC2f | IG↔JP | + |
| HC2g | IG↔JS | + |
| HC2h | IG↔WB | + |
| HC2i | ID↔WE | + |
| HC2j | ID↔JP | + |
| HC2k | ID↔JS | + |
| HC2l | ID↔WB | + |
| HC3a | EM↔AUT | – |
| HC3b | EM↔COM | – |
| HC3c | EM↔REL | – |
| HC3d | EM↔NSUP | – |
| HC3e | IJ↔AUT | – |
| HC3r | IJ↔COM | – |
| HC3g | IJ↔REL | – |
| HC3h | IJ↔NSUP | – |
| HC4a | EM↔WE | – |
| HC4b | EM↔JP | – |
| HC4c | EM↔JS | – |
| HC4d | EM↔WB | – |
| HC4e | IJ↔WE | – |
| HC4f | IJ↔JP | – |
| HC4g | IJ↔JS | – |
| HC4h | IJ↔WB | – |
| HC5a | IM↔TU | – |
| HC5b | IM↔BU | – |
| HC5c | IG↔TU | – |
| HC5d | IG↔BU | – |
| HC5e | ID↔TU | – |
| HC5f | ID↔BU | – |
| HC6a | EM↔TU | + |
| HC6b | EM↔BU | + |
| HC6c | IJ↔TU | + |
| HC6d | IJ↔BU | + |

*Note*. EM = Global or aggregated autonomous motivation and intrinsic motivation; IG = Integrated regulation; ID = Identified regulation; IJ = Introjected regulation; EM/ER = Global or aggregated extrinsic and controlled motivation and external regulation; AUT = Autonomy need satisfaction; COM = Competence need satisfaction; REL = Relatedness need satisfaction; NSUP = Need support; WE = Work engagement; JP = Job performance; JS = Job satisfaction; WB = Well-Being; TU = Turnover; BU = Burnout.

**Table A2**

*Hypothesized Effects and Accompanying Sign of Direct and Indirect Effects of Self-Determination Theory Constructs on Workplace Outcomes for the Full and Truncated Models*

| Hypothesis | Effect | Expected sign |
| --- | --- | --- |
|  | Full model |  |
|  | Direct effects |  |
| HF1a | NS→AUT | + |
| HF1b | NS→COM | + |
| HF1c | NS→REL | + |
| HF2a | AUT→IM | + |
| HF2b | AUT→ID | + |
| HF2c | AUT→IJ | + |
| HF2d | AUT→ER | ‒ |
| HF3a | COM→IM | + |
| HF3b | COM→ID | + |
| HF3c | COM→IJ | + |
| HF3d | COM→ER | ‒ |
| HF4a | REL→IM | + |
| HF4b | REL→ID | + |
| HF4c | REL→IJ | + |
| HF4d | REL→ER | ‒ |
| HF5a | IM→WE | + |
| HF5b | IM→JP | + |
| HF5c | IM→JS | + |
| HF5d | IM→WB | + |
| HF5e | IM→TU | ‒ |
| HF5f | IM→BU | ‒ |
| HF6a | ID→WE | + |
| HF6b | ID→JP | + |
| HF6c | ID→JS | + |
| HF6d | ID→WB | + |
| HF6e | ID→TU | ‒ |
| HF6f | ID→BU | ‒ |
| HF7a | IJ→WE | ‒ |
| HF7b | IJ→JP | ‒ |
| HF7c | IJ→JS | ‒ |
| HF7d | IJ→WB | ‒ |
| HF7e | IJ→TU | + |
| HF7f | IJ→BU | + |
| HF8a | ER→WE | ‒ |
| HF8b | ER→JP | ‒ |
| HF8c | ER→JS | ‒ |
| HF8d | ER→WB | ‒ |
| HF8e | ER→TU | + |
| HF8f | ER→BU | + |
|  | Indirect effects |  |
| HF9a | NSUP→NSAT→AM→WE | + |
| HF9b | NSUP→NSAT→AM→JP | + |
| HF9c | NSUP→NSAT→AM→JS | + |
| HF9d | NSUP→NSAT→AM→WB | + |
| HF9e | NSUP→NSAT→AM→TU | ‒ |
| HF9f | NSUP→NSAT→AM→BU | ‒ |
| HF10a | NSUP→NSAT→CM→WE | ‒ |
| HF10b | NSUP→NSAT→CM→JP | ‒ |
| HF10c | NSUP→NSAT→CM→JS | ‒ |
| HF10d | NSUP→NSAT→CM→WB | ‒ |
| HF10e | NSUP→NSAT→CM→TU | + |
| HF10f | NSUP→NSAT→CM→BU | + |
|  | Sums of indirect effects |  |
| HF11a | NSUP→NSAT→AM/CM→WE^a^ | + |
| HF11b | NSUP→NSAT→AM/CM→JP^b^ | + |
| HF11c | NSUP→NSAT→AM/CM→JS^c^ | + |
| HF11d | NSUP→NSAT→AM/CM→WB^d^ | + |
| HF11e | NSUP→NSAT→AM/CM→TU^e^ | ‒ |
| HF11f | NSUP→NSAT→AM/CM→BU^f^ | ‒ |
|  |  |  |
|  | Truncated model |  |
|  | Direct effects |  |
| HT1 | NSUP→NS | + |
| HT2a | NSAT→AM | + |
| HT2b | NSAT→CM | ‒ |
| HT3a | AM→AD | + |
| HT3b | AM→MAL | ‒ |
| HT4a | CM→AD | ‒ |
| HT4b | CM→MAL | + |
| HT5a | NSUP→AD | + |
| HT5b | NSUP→MAL | ‒ |
|  | Indirect effects |  |
| HT6a | NSUP→NSAT→AM→AD | + |
| HT6b | NSUP→NSAT→CM→AD | ‒ |
| HT7a | NSUP→NSAT→AM→MAL | + |
| HT7b | NSUP→NSAT→CM→MAL | ‒ |
|  | Sums of indirect effects |  |
| HT8a | NSUP→NSAT→CM/AM→AD^a^ | + |
| HT8b | NSUP→NSAT→CM/AM→MAL^b^ | ‒ |
|  | Total Effects |  |
| HT9a | NSUP→AD^c^ | + |
| HT9b | NSUP→MAL^d^ | ‒ |

*Note*. ^a^Sum of indirect effects of need support on work engagement through all self-determination theory constructs; ^b^Sum of indirect effects of need support on job performance through all self-determination theory constructs; ^c^Sum of indirect effects of need support on job satisfaction through all self-determination theory constructs; ^d^Sum of indirect effects of need support on well-being through all self-determination theory constructs; ^e^Sum of indirect effects of need support on turnover through all self-determination theory constructs; ^f^Sum of indirect effects of need support on burnout through all self-determination theory constructs. IM = Intrinsic motivation; ER = External regulation; AUT = Autonomy need satisfaction; COM = Competence need satisfaction; REL = Relatedness need satisfaction; NSUP = Need support; ID = Identified regulation; IJ = Introjected regulation; JP = Job performance; JS = Job satisfaction; TU = Turnover; BU = Burnout; WE = Work engagement; WB = Well-Being; NSAT = All need satisfaction constructs; AM = Autonomous forms of motivation collapsed across intrinsic motivation and identified regulation constructs for the truncated model only; CM = Controlled forms of motivation collapsed across intrinsic motivation and introjected and external regulation constructs for the truncated model only; AD = Adaptive workplace outcomes collapsed across job performance, job satisfaction, work engagement, and well-being outcome variables for the truncated model only; MAL = Maladaptive workplace outcomes collapsed across turnover and burnout outcomes for the truncated model only.

**Table A3**

*Hypothesized Effects of Study-Level Moderator Variables on Specific Indirect Effects of the Truncated Model*

| Hypothesis | Effect(s) | Moderator^a^ | Direction |
| --- | --- | --- | --- |
| HM1a | NSUPP→NSAT | Employee type | Upwards |
| HM1b | NSAT→AM | Employee type | Upwards |
| HM1c | AM→AD | Employee type | Upwards |
| HM2a | NSUPP→NSAT | Work type | Upwards |
| HM2b | NSAT→AM | Work type | Upwards |
| HM2c | AM→AD | Work type | Upwards |
| HM3a | AM→AD | Country GDP | Upwards |
| HM3b | CM→MAL | Country GDP | Downwards |
| HM4a | AM→AD | Cultural orientation | No difference |
| HM4b | CM→AD | Cultural orientation | No difference |
| HM4c | AM→MAL | Cultural orientation | No difference |
| HM4d | CM→MAL | Cultural orientation | No difference |
| HM5a | NSUPP→NSAT | Proximity of leader autonomy support | Upwards |
| HM5b | NSAT→AM | Proximity of leader autonomy support | Upwards |
| HM6 | NSUP→NSAT→AM→AD | Employee type | Upwards |
| HM7 | NSUP→NSAT→AM→AD | Work type | Upwards |
| HM8 | NSUP→NSAT→AM→AD | Country GDP | Upwards |
| HM9 | NSUP→NSAT→AM→AD | Cultural orientation | No difference |
| HM10 | NSUP→NSAT→AM→AD | Proximity of leader autonomy support | Upwards |

*Note*. All moderator analyses performed using the truncated model. ^a^Moderators were coded as binary contrast variables as follows: Employee type (1 = Teachers, 2 = Healthcare workers, 0 = Corporate employees); Work type (1 = Public service, 0 = For-profit); Country GDP (1 = Country inside top 10 ranked by GDP, 0 = Country ranked outside the global top 10 by GDP); Cultural orientation (1 = Individualist orientation, 0 = Collectivist orientation); Proximity of leader autonomy support (1 = Proximal, 0 = Distal). AM = Autonomous forms of motivation collapsed across intrinsic motivation and identified regulation constructs; CM = Controlled forms of motivation collapsed across intrinsic motivation and introjected and external regulation constructs; AD = Adaptive workplace outcomes collapsed across job performance, job satisfaction, work engagement, and well-being outcome variables; MAL = Maladaptive workplace outcomes collapsed across turnover and burnout outcomes; GDP = Gross domestic product of the country from which the sample was drawn.

Supplement B

*Search Strings Used to Conduct Database Searches*

Psyc Info: ((“Self-determination theory”) OR (“intrinsic motivat*”) OR (“introjected motivat*”) OR (“integrated motivat*”) OR (“identified motiv*”) OR (“identified regulat*”) OR (“introjected regulat”) OR (“integrated regulat*”) OR (“external regulat*”) OR (“external motivat*”) OR (“psychological need*”) OR (“causality orientation*”) OR (“cognitive evaluation theory”)) AND ((“occupation”) OR (“business”) OR (“management”) OR (“burnout”) OR (“work performance”) OR (“industrial”) OR (“job satisfaction”) OR (“absenteeism”) OR (“presenteeism”) OR (“work stress”))

Scopus: (TITLE-ABS(“Self-determination theory”) OR TITLE-ABS(“intrinsic motivat*”) OR TITLE-ABS(“autonom* motivat*”) OR TITLE-ABS(“introjected motivat*”) OR TITLE-ABS(“integrated motivat*”) OR TITLE-ABS(“Introjected regulat*”) OR TITLE-ABS(“integrated regulat*”) OR TITLE-ABS(“identified motivat*”) OR TITLE-ABS(“identified regulat*”) OR TITLE-ABS(“external motivat*”) OR TITLE-ABS(“external regulat*”) OR TITLE-ABS(“psychological need*”) OR TITLE-ABS(“causality orientation*”) OR TITLE-ABS(“cognitive evaluation theory”)) AND (TITLE-ABS(“occupation”) OR TITLE-ABS(“business”) OR TITLE-ABS(“management”) OR TITLE-ABS(‘burnout’) OR TITLE-ABS(“work performance”) OR TITLE-ABS(“industrial”) OR TITLE-ABS(“job satisfaction”) OR TITLE-ABS(“absenteeism”) OR TITLE-ABS(“presenteeism”) OR TITLE-ABS(“work stress”))

Pubmed: (“Self-determination theory” OR “intrinsic motivat*” OR “introjected motivat*” OR “integrated motivat*” OR “identified motiv*” OR “identified regulat*” OR “introjected regulat* OR “integrated regulat*” OR *external regulat*” OR “external motivat*” OR “psychological need*” OR “causality orientation*” OR “autonom* motivat*” OR “cognitive evaluation theory”) AND (“occupation” OR “business” OR “management” OR “burnout” OR “work performance” OR “industrial” OR “job satisfaction” OR “absenteeism” OR “presenteeism” OR “work stress”)

Web of Science: TS=(“Self-determination theory” OR “intrinsic motivat*” OR “introjected motivat*” OR “integrated motivat*” OR “identified motiv*” OR “identified regulat*” OR “introjected regulat* OR “integrated regulat*” OR *external regulat*” OR “external motivat*” OR “psychological need*” OR “causality orientation*” OR “autonom* motivat*” OR “cognitive evaluation theory”) AND TS=(“occupation” OR “business” OR “management” OR “burnout” OR “work performance” OR “industrial” OR “job satisfaction” OR “absenteeism” OR “presenteeism” OR “work stress”)

Supplement C

*PRISMA Flow Diagram for Study Search and Inclusion Strategy*

Full-text articles excluded, with reasons

(*k* = 322):

- Ineligible (e.g., review, book, qualitative study) (*k* = 52)
- Did not meet inclusion criteria (e.g., did not include relevant measure of self-determination theory construct or workplace outcome) (*k* = 183)
- Did not meet inclusion criteria (e.g., not measured in a workplace setting) (*k* = 7)
- Full-text not available from library or authors (*k* = 76)_
- Could not be translated (*k* = 4)

## Identification

## Eligibility

## Screening

## Inclusion

Insufficient data reported and unavailable from authors (*k* = 22)

Final sample after data extraction

*k* = 192

Total sample after full-text screening

*k* = 214

Web of Science

*k* = 3694

PubMed

*k* = 1194

PsycInfo

*k* =3632

SCOPUS

*k* = 3216

Total sample

*k* = 11736

Following duplicate screening

*k* = 8677

Total sample after title screening

*k* = 1151

Total sample after abstract screening

*k* = 536

Supplement D

*PRISMA Checklist*

| **Section/topic** | **#** | **Checklist item** | **Reported on page #** |
| --- | --- | --- | --- |
| **TITLE** | | |  |
| Title | 1 | Identify the report as a systematic review, meta-analysis, or both. | 1 |
| **ABSTRACT** | | |  |
| Structured summary | 2 | Provide a structured summary including, as applicable: background; objectives; data sources; study eligibility criteria, participants, and interventions; study appraisal and synthesis methods; results; limitations; conclusions and implications of key findings; systematic review registration number. | 2 |
| **INTRODUCTION** | | |  |
| Rationale | 3 | Describe the rationale for the review in the context of what is already known. | 3-6 |
| Objectives | 4 | Provide an explicit statement of questions being addressed with reference to participants, interventions, comparisons, outcomes, and study design (PICOS). | 7-10 |
| **METHODS** | | |  |
| Protocol and registration | 5 | Indicate if a review protocol exists, if and where it can be accessed (e.g., Web address), and, if available, provide registration information including registration number. | 10 |
| Eligibility criteria | 6 | Specify study characteristics (e.g., PICOS, length of follow-up) and report characteristics (e.g., years considered, language, publication status) used as criteria for eligibility, giving rationale. | 10-11 |
| Information sources | 7 | Describe all information sources (e.g., databases with dates of coverage, contact with study authors to identify additional studies) in the search and date last searched. | 10 |
| Search | 8 | Present full electronic search strategy for at least one database, including any limits used, such that it could be repeated. | Supplemental Materials |
| Study selection | 9 | State the process for selecting studies (i.e., screening, eligibility, included in systematic review, and, if applicable, included in the meta-analysis). | 10-11 |
| Data collection process | 10 | Describe method of data extraction from reports (e.g., piloted forms, independently, in duplicate) and any processes for obtaining and confirming data from investigators. | 11-17 |
| Data items | 11 | List and define all variables for which data were sought (e.g., PICOS, funding sources) and any assumptions and simplifications made. | 11-17 |
| Risk of bias in individual studies | 12 | Describe methods used for assessing risk of bias of individual studies (including specification of whether this was done at the study or outcome level), and how this information is to be used in any data synthesis. | 18-19 |
| Summary measures | 13 | State the principal summary measures (e.g., risk ratio, difference in means). | 17-18 |
| **Section/topic** | **#** | **Checklist item** | **Reported on page #** |
| Synthesis of results | 14 | Describe the methods of handling data and combining results of studies, if done, including measures of consistency (e.g., I^2^) for each meta-analysis. | 17-18 |
| Risk of bias across studies | 15 | Specify any assessment of risk of bias that may affect the cumulative evidence (e.g., publication bias, selective reporting within studies). | 18, Supplemental Materials |
| Additional analyses | 16 | Describe methods of additional analyses (e.g., sensitivity or subgroup analyses, meta-regression), if done, indicating which were pre-specified. | 17-18, Supplemental Materials |
| **RESULTS** | | |  |
| Study selection | 17 | Give numbers of studies screened, assessed for eligibility, and included in the review, with reasons for exclusions at each stage, ideally with a flow diagram. | 11, Supplemental Materials |
| Study characteristics | 18 | For each study, present characteristics for which data were extracted (e.g., study size, PICOS, follow-up period) and provide the citations. | Supplemental Materials |
| Risk of bias within studies | 19 | Present data on risk of bias of each study and, if available, any outcome level assessment (see item 12). | Supplemental Materials |
| Results of individual studies | 20 | For all outcomes considered (benefits or harms), present, for each study: (a) simple summary data for each intervention group (b) effect estimates and confidence intervals, ideally with a forest plot. | N/A |
| Synthesis of results | 21 | Present results of each meta-analysis done, including confidence intervals and measures of consistency. | Supplemental Materials |
| Risk of bias across studies | 22 | Present results of any assessment of risk of bias across studies (see Item 15). | Supplemental Materials |
| Additional analysis | 23 | Give results of additional analyses, if done (e.g., sensitivity or subgroup analyses, meta-regression [see Item 16]). | 37-45, Supplemental Materials |
| **DISCUSSION** | | |  |
| Summary of evidence | 24 | Summarize the main findings including the strength of evidence for each main outcome; consider their relevance to key groups (e.g., healthcare providers, users, and policy makers). | 23-29 |
| Limitations | 25 | Discuss limitations at study and outcome level (e.g., risk of bias), and at review-level (e.g., incomplete retrieval of identified research, reporting bias). | 29-30 |
| Conclusions | 26 | Provide a general interpretation of the results in the context of other evidence, and implications for future research. | 31 |
| **FUNDING** | | |  |
| Funding | 27 | Describe sources of funding for the systematic review and other support (e.g., supply of data); role of funders for the systematic review. | 1 |

Supplement E

*List of Articles Included in Meta-Analysis*

Chowdhury, M. S. (2007). Enhancing motivation and work performance of the salespeople: The impact of supervisors' behavior. *African Journal of Business Management*, *1*(9).

Chung-Yan, G. A. (2010). The nonlinear effects of job complexity and autonomy on job satisfaction, turnover, and psychological well-being. *Journal of Occupational Health Psychology*, *15*(3), 237. https://doi.org/10.1037/a0019823

Clayton, B. C. (2015). Shared vision and autonomous motivation vs. financial incentives driving success in corporate acquisitions. *Frontiers in Psychology*, *5*, 1466. https://doi.org/10.3389%2Ffpsyg.2014.01466

Collie, R. J., Granziera, H., & Martin, A. J. (2018). Teachers’ perceived autonomy support and adaptability: An investigation employing the job demands-resources model as relevant to workplace exhaustion, disengagement, and commitment. *Teaching and Teacher Education*, *74*, 125-136. https://psycnet.apa.org/doi/10.1016/j.tate.2018.04.015

Collie, R. J., Shapka, J. D., Perry, N. E., & Martin, A. J. (2016). Teachers’ psychological functioning in the workplace: Exploring the roles of contextual beliefs, need satisfaction, and personal characteristics. *Journal of Educational Psychology*, *108*(6), 788. http://dx.doi.org/10.1037/edu0000088

Cross, W., & Wyman, P. A. (2006). Training and motivational factors as predictors of job satisfaction and anticipated job retention among implementers of a school-based prevention program. *Journal of Primary Prevention*, *27*, 195-215. https://doi.org/10.1007/s10935-005-0018-4

Cummings, T. G., & Bigelow, J. (1976). Satisfaction, job involvement, and intrinsic motivation: An extension of Lawler and Hall's factor analysis. *Journal of Applied Psychology*, *61*(4), 523. https://psycnet.apa.org/doi/10.1037/0021-9010.61.4.523

Dagenais-Desmarais, V., Leclerc, J. S., & Londei-Shortall, J. (2018). The relationship between employee motivation and psychological health at work: A chicken-and-egg situation? *Work & Stress*, *32*(2), 147-167. https://doi.org/10.1080/02678373.2017.1317880

Dahling, J. J., & Lauricella, T. K. (2017). Linking job design to subjective career success: A testof Self-Determination Theory. *Journal of Career Assessment*, *25*(3), 371-388. https://doi.org/10.1177/1069072716639689

Dai, B., & Akey-Torku, B. (2020). The influence of managerial psychology on job satisfaction among healthcare employees in Ghana. *Healthcare*, *8*(3), 262. https://doi.org/10.3390/healthcare8030262

Danish, R. Q., Khalid Khan, M., Shahid, A. U., Raza, I., & Humayon, A. A. (2015). Effect of intrinsic rewards on task performance of employees: Mediating role of motivation. *International Journal of Organizational Leadership*, *4*, 33-46. https://doi.org/10.33844/ijol.2015.60415

De Klerk, J. J. (2006). Motivation to work, work commitment and man's will to meaning (Doctoral dissertation, University of Pretoria).

Deci, E. L., Ryan, R. M., Gagné, M., Leone, D. R., Usunov, J., & Kornazheva, B. P. (2001). Need satisfaction, motivation, and well-being in the work organizations of a former eastern bloc country: A cross-cultural study of self-determination. *Personality and Social Psychology Bulletin*, *27*(8), 930-942. https://psycnet.apa.org/doi/10.1177/0146167201278002

Demircioglu, M. A. (2018). Examining the effects of social media use on job satisfaction in the Australian public service: Testing self-determination theory. *Public Performance & Management Review*, *41*(2), 300-327. https://doi.org/10.1080/15309576.2017.1400991

Demircioglu, M. A. (2021). Sources of innovation, autonomy, and employee job satisfaction in public organizations. *Public Performance & Management Review*, *44*(1), 155-186. https://doi.org/10.1080/15309576.2020.1820350

Djordjević, D. B., Petrović, D., Vuković, D., Mihailović, D., & Dimić, A. (2015). Motivation and job satisfaction of health workers in a specialized health institution in Serbia. *Vojnosanitetski Pregled*, *72*(8). https://doi.org/10.2298/vsp131110055d

Dysvik, A., & Kuvaas, B. (2008). The relationship between perceived training opportunities, work motivation and employee outcomes. *International Journal of Training and Development*, *12*(3), 138-157. https://doi.org/10.1111/j.1468-2419.2008.00301.x

Dysvik, A., & Kuvaas, B. (2010). Exploring the relative and combined influence of mastery‐approach goals and work intrinsic motivation on employee turnover intention. *Personnel Review*, *39*(5), 622-638. https://psycnet.apa.org/doi/10.1108/00483481011064172

Dysvik, A., & Kuvaas, B. (2011). Intrinsic motivation as a moderator on the relationship between perceived job autonomy and work performance. *European Journal of Work and Organizational Psychology*, *20*(3), 367-387. https://doi.org/10.1080/13594321003590630

Dysvik, A., & Kuvaas, B. (2013). Intrinsic and extrinsic motivation as predictors of work effort: The moderating role of achievement goals. *British Journal of Social Psychology*, *52*(3), 412-430. https://psycnet.apa.org/doi/10.1111/j.2044-8309.2011.02090.x

Dysvik, A., Kuvaas, B., & Buch, R. (2010). Trainee programme reactions and work performance: The moderating role of intrinsic motivation. *Human Resource Development International*, *13*(4), 409-423. https://psycnet.apa.org/doi/10.1080/13678868.2010.501962

Elias, S. M., Smith, W. L., & Barney, C. E. (2012). Age as a moderator of attitude towards technology in the workplace: Work motivation and overall job satisfaction. *Behaviour & Information Technology*, *31*(5), 453-467. https://psycnet.apa.org/doi/10.1080/0144929X.2010.513419

Eyal, O., & Roth, G. (2011). Principals' leadership and teachers' motivation: Self‐determination theory analysis. *Journal of Educational Administration*, *49*(3), 256-275. https://doi.org/10.1108/09578231111129055

Falvo, R., Capozza, D., Di Bernardo, G. A., & Manganelli, A. (2016). Attributions of competence and warmth to the leader and employees’ organizational commitment: The mediation role of the satisfaction of basic needs. *Testing, Psychometrics, Methodology in Applied Psychology*, *23*(2).

Fernet, C., Austin, S., Trépanier, S. G., & Dussault, M. (2013). How do job characteristics contribute to burnout? Exploring the distinct mediating roles of perceived autonomy, competence, and relatedness. *European Journal of Work and Organizational Psychology*, *22*(2), 123-137. https://psycnet.apa.org/doi/10.1080/1359432X.2011.632161

Fernet, C., Austin, S., & Vallerand, R. J. (2012). The effects of work motivation on employee exhaustion and commitment: An extension of the JD-R model. *Work & Stress*, *26*(3), 213-229. http://dx.doi.org/10.1080/02678373.2012.713202

Fernet, C., Chanal, J., & Guay, F. (2017). What fuels the fire: Job-or task-specific motivation (or both)? On the hierarchical and multidimensional nature of teacher motivation in relation to job burnout. *Work & Stress*, *31*(2), 145-163. https://psycnet.apa.org/doi/10.1080/02678373.2017.1303758

Fernet, C., Gagné, M., & Austin, S. (2010). When does quality of relationships with coworkers predict burnout over time? The moderating role of work motivation. *Journal of Organizational Behavior*, *31*(8), 1163-1180. https://doi.org/10.1002/job.673

Fernet, C., Guay, F., Senécal, C., & Austin, S. (2012). Predicting intraindividual changes in teacher burnout: The role of perceived school environment and motivational factors. *Teaching and Teacher Education*, *28*(4), 514-525. https://doi.org/10.1016/j.tate.2011.11.013

Fernet, C., Litalien, D., Morin, A. J., Austin, S., Gagné, M., Lavoie-Tremblay, M., & Forest, J. (2020). On the temporal stability of self-determined work motivation profiles: A latent transition analysis. *European Journal of Work and Organizational Psychology*, *29*(1), 49-63. https://doi.org/10.1080/1359432X.2019.1688301

Fernet, C., Morin, A. J., Austin, S., Gagné, M., Litalien, D., Lavoie-Tremblay, M., & Forest, J. (2020). Self-determination trajectories at work: A growth mixture analysis. *Journal of Vocational Behavior*, *121*, 103473. https://doi.org/10.1016/j.jvb.2020.103473

Fernet, C., Trépanier, S. G., Austin, S., & Levesque-Côté, J. (2016). Committed, inspiring, andhealthy teachers: How do school environment and motivational factors facilitate optimal functioning at career start? *Teaching and Teacher Education*, *59*, 481-491. https://doi.org/10.1016/j.tate.2016.07.019

Fernet, C., Trépanier, S. G., Demers, M., & Austin, S. (2017). Motivational pathways of occupational and organizational turnover intention among newly registered nurses in Canada. *Nursing Outlook*, *65*(4), 444-454. https://doi.org/10.1016/j.outlook.2017.05.008

Foulk, T. A., Lanaj, K., & Krishnan, S. (2019). The virtuous cycle of daily motivation: Effects of daily strivings on work behaviors, need satisfaction, and next-day strivings. *Journal of Applied Psychology*, *104*(6), 755. https://doi.org/10.1037/apl0000385

Galletta, M., & Portoghese, I. (2012). Organizational citizenship behavior in healthcare: The roles of autonomous motivation, affective commitment and learning orientation. *Revue Internationale de Psychologie Sociale*, *25*(3), 121-145.

Galletta, M., Portoghese, I., Pili, S., Piazza, M. F., & Campagna, M. (2016). The effect of work motivation on a sample of nurses in an Italian healthcare setting. *Work*, *54*(2), 451-460. https://doi.org/10.3233/wor-162327

Galletta, M., Vandenberghe, C., Portoghese, I., Allegrini, E., Saiani, L., & Battistelli, A. (2019). A cross‐lagged analysis of the relationships among workgroup commitment, motivation and proactive work behaviour in nurses. *Journal of Nursing Management*, *27*(6), 1148-1158. https://doi.org/10.1111/jonm.12786

García-Chas, R., Neira-Fontela, E., & Varela-Neira, C. (2016). High-performance work systems and job satisfaction: a multilevel model. *Journal of Managerial Psychology*, *31*(2), 451-466. https://psycnet.apa.org/doi/10.1108/JMP-04-2013-0127

Gatt, G., & Jiang, L. (2021). Can different types of non-territorial working satisfy employees’ needs for autonomy and belongingness? Insights from self-determination theory. *Environment and Behavior*, *53*(9), 953-986. https://doi.org/10.1177/0013916520942603

Gillet, N., Colombat, P., Michinov, E., Pronost, A. M., & Fouquereau, E. (2013). Procedural justice, supervisor autonomy support, work satisfaction, organizational identification and job performance: The mediating role of need satisfaction and perceived organizational support. *Journal of Advanced Nursing*, *69*(11), 2560-2571. https://doi.org/10.1111/jan.12144

Gillet, N., Forest, J., Benabou, C., & Bentein, K. (2015). The effects of organizational factors, psychological need satisfaction and thwarting, and affective commitment on workers’ well-being and turnover intentions. *Travail Humain*, 119-140. https://psycnet.apa.org/doi/10.3917/th.782.0119

Gillet, N., Fouquereau, E., Huyghebaert, T., & Colombat, P. (2015). The effects of job demands and organizational resources through psychological need satisfaction and thwarting. *The Spanish Journal of Psychology*, *18*, E28. https://doi.org/10.1017/sjp.2015.30

Gillet, N., Fouquereau, E., Vallerand, R. J., Abraham, J., & Colombat, P. (2018). The role of workers’ motivational profiles in affective and organizational factors. *Journal of Happiness Studies*, *19*, 1151-1174. https://psycnet.apa.org/doi/10.1007/s10902-017-9867-9

Gillet, N., Gagné, M., Sauvagère, S., & Fouquereau, E. (2013). The role of supervisor autonomy support, organizational support, and autonomous and controlled motivation in predicting employees' satisfaction and turnover intentions. *European Journal of Work and Organizational Psychology*, *22*(4), 450-460. https://psycnet.apa.org/doi/10.1080/1359432X.2012.665228

Gillet, N., Le Gouge, A., Pierre, R., Bongro, J., Méplaux, V., Brunault, P., Guyetant, S., Fremont, C., Camus, V., Colombat, P. and Fouquereau, E., & Cheyroux, P. (2019). Managerial style and well‐being among psychiatric nurses: A prospective study. *Journal of Psychiatric and Mental Health Nursing*, *26*(7-8), 265-273. https://doi.org/10.1111/jpm.12544

Gillet, N., Morin, A. J., Ndiaye, A., Colombat, P., & Fouquereau, E. (2020). A test of work motivation profile similarity across four distinct samples of employees. *Journal of Occupational and Organizational Psychology*, *93*(4), 988-1030. https://doi.org/10.1111/joop.12322

Gkorezis, P., & Kastritsi, A. (2017). Employee expectations and intrinsic motivation: Work-related boredom as a mediator. *Employee Relations*, *39*(1), 100-111. https://psycnet.apa.org/doi/10.1108/ER-02-2016-0025

Godinho-Bitencourt, R., Pauli, J., & Costenaro-Maciel, A. (2019). Influence of the organizational support on generation Y’s work motivation. *Estudios Gerenciales, Universidad Icesi, 35*(153), 388-398.

Gomez‐Baya, D., & Lucia‐Casademunt, A. M. (2018). A self‐determination theory approach to health and well‐being in the workplace: Results from the sixth European working conditions survey in Spain. *Journal of Applied Social Psychology*, *48*(5), 269-283. https://doi.org/10.1111/jasp.12511

Gómez-Baya, D., Lucia-Casademunt, A. M., & Salinas-Pérez, J. A. (2018). Gender differences in psychological well-being and health problems among European health professionals: Analysis of psychological basic needs and job satisfaction. *International Journal of Environmental Research and Public Health*, *15*(7), 1474. https://doi.org/10.3390%2Fijerph15071474

Goodboy, A. K., Martin, M. M., & Bolkan, S. (2020). Workplace bullying and work engagement: A self-determination model. *Journal of Interpersonal Violence*, *35*(21-22), 4686-4708. https://doi.org/10.1177/0886260517717492

Goštautaitė, B., Bučiūnienė, I., & Milašauskienė, Ž. (2022). HRM and work outcomes: The role of basic need satisfaction and age. *The International Journal of Human Resource Management*, *33*(2), 169-202. https://doi.org/10.1080/09585192.2019.1683049

Grant, K., Cravens, D. W., Low, G. S., & Moncrief, W. C. (2001). The role of satisfaction with territory design on the motivation, attitudes, and work outcomes of salespeople. *Journal of the Academy of Marketing Science*, *29*(2), 165-178. https://psycnet.apa.org/doi/10.1177/03079459994533

Graves, L. M., & Luciano, M. M. (2013). Self-determination at work: Understanding the role of leader-member exchange. *Motivation and Emotion*, *37*, 518-536. https://psycnet.apa.org/doi/10.1007/s11031-012-9336-z

Greguras, G. J., & Diefendorff, J. M. (2009). Different fits satisfy different needs: Linking person-environment fit to employee commitment and performance using self-determination theory. *Journal of Applied Psychology*, *94*(2), 465. https://doi.org/10.1037/a0014068

Hadi, R., & Adil, A. (2010). Job characteristics as predictors of work motivation and job satisfaction of bank employees. *Journal of the Indian Academy of Applied Psychology*. *36*(2), 294–299.

Haldorai, K., Kim, W. G., Phetvaroon, K., & Li, J. (2020). Left out of the office “tribe”: The influence of workplace ostracism on employee work engagement. *International Journal of Contemporary Hospitality Management*, *32*(8), 2717-2735. https://psycnet.apa.org/doi/10.1108/IJCHM-04-2020-0285

Haski-Leventhal, D., Kach, A., & Pournader, M. (2019). Employee need satisfaction and positive workplace outcomes: The role of corporate volunteering. *Nonprofit and Voluntary Sector Quarterly*, *48*(3), 593-615. https://doi.org/10.1177/0899764019829829

Hayati, K., & Caniago, I. (2012). Islamic work ethic: The role of intrinsic motivation, job satisfaction, organizational commitment and job performance. *Procedia-Social and Behavioral Sciences*, *65*, 1102-1106. https://doi.org/10.1016/j.sbspro.2014.05.148

Heyns, M., & Rothmann, S. (2018). Volitional trust, autonomy satisfaction, and engagement at work. *Psychological Reports*, *121*(1), 112-134. https://doi.org/10.1177/0033294117718555

Klassen, R. M., Perry, N. E., & Frenzel, A. C. (2012). Teachers' relatedness with students: An underemphasized component of teachers' basic psychological needs. *Journal of Educational Psychology*, *104*(1), 150. https://doi.org/10.1037/a0026253

Kong, D. T., & Ho, V. T. (2016). A self-determination perspective of strengths use at work: Examining its determinant and performance implications. *The Journal of Positive Psychology*, *11*(1), 15-25. https://doi.org/10.1080/17439760.2015.1004555

Kosmala-Anderson, J. P., Wallace, L. M., & Turner, A. (2010). Confidence matters: A Self-Determination Theory study of factors determining engagement in self-management support practices of UK clinicians. *Psychology, Health & Medicine*, *15*(4), 478-491. https://doi.org/10.1080/13548506.2010.487104

Kovjanic, S., Schuh, S. C., & Jonas, K. (2013). Transformational leadership and performance: An experimental investigation of the mediating effects of basic needs satisfaction and work engagement. *Journal of Occupational and Organizational Psychology*, *86*(4), 543-555. https://doi.org/10.1111/joop.12022

Kovjanic, S., Schuh, S. C., Jonas, K., Quaquebeke, N. V., & Van Dick, R. (2012). How do transformational leaders foster positive employee outcomes? A self‐determination‐based analysis of employees' needs as mediating links. *Journal of Organizational Behavior*, *33*(8), 1031-1052. https://doi.org/10.1002/job.1771

Kuvaas, B. (2006). Work performance, affective commitment, and work motivation: The roles of pay administration and pay level. *The International Journal of Industrial, Occupational and Organizational Psychology and Behavior*, *27*(3), 365-385. https://doi.org/10.1002/job.377

Kuvaas, B. (2006). Performance appraisal satisfaction and employee outcomes: Mediating and moderating roles of work motivation. *The International Journal of Human Resource Management*, *17*(3), 504-522. https://doi.org/10.1080/09585190500521581

Kuvaas, B. (2009). A test of hypotheses derived from self‐determination theory among public sector employees. *Employee Relations*, *31*(1), 39-56. https://doi.org/10.1108/01425450910916814

Kuvaas, B., Buch, R., Weibel, A., Dysvik, A., & Nerstad, C. G. (2017). Do intrinsic and extrinsic motivation relate differently to employee outcomes? *Journal of Economic Psychology*, *61*, 244-258. https://doi.org/10.1016/j.joep.2017.05.004

Kuvaas, B., & Dysvik, A. (2009). Perceived investment in employee development, intrinsic motivation and work performance. *Human Resource Management Journal*, *19*(3), 217-236. https://doi.org/10.1111/j.1748-8583.2009.00103.x

Kuvaas, B., & Dysvik, A. (2010). Does best practice HRM only work for intrinsically motivated employees? *The International Journal of Human Resource Management*, *21*(13), 2339-2357. https://psycnet.apa.org/doi/10.1080/09585192.2010.516589

Lai, L. (2011). Employees' perceptions of the opportunities to utilize their competences: exploring the role of perceived competence mobilization. *International Journal of Training and Development*, *15*(2), 140-157. https://doi.org/10.1111/j.1468-2419.2011.00376.x

Landry, A., Gagné, M., Forest, J., Guerrero, S., Séguin, M., & Papachristopoulos, K. (2017). An integrative SDT-based investigation of the relation between financial incentives, motivation, and performance. *Journal of Personnel Psychology, 16*(2), 61-76. https://psycnet.apa.org/doi/10.1027/1866-5888/a000182

Levesque, M., Blais, M. R., & Hess, U. (2004). Dynamique motivationnelle de l'epuisement et du bien-etre chez des enseignants Africains. *Canadian Journal of Behavioural Science/Revue Canadienne des Sciences du Comportement*, *36*(3), 190. https://psycnet.apa.org/doi/10.1037/h0087229

Lewig, K. A., & Dollard, M. F. (2003). Emotional dissonance, emotional exhaustion and job satisfaction in call centre workers. *European Journal of Work and Organizational Psychology*, *12*(4), 366-392. https://psycnet.apa.org/doi/10.1080/13594320344000200

Li, J., Han, X., Qi, J., & He, X. (2021). Managing one’s career: The joint effects of job autonomy, supervisor support, and calling. *Journal of Career Development*, *48*(6), 973-986. https://doi.org/10.1177/0894845320906464

Li, M., Wang, Z., You, X., & Gao, J. (2015). Value congruence and teachers’ work engagement: The mediating role of autonomous and controlled motivation. *Personality and Individual Differences*, *80*, 113-118. https://doi.org/10.1016/j.paid.2015.02.021

Liu, D., Zhang, S., Wang, L., & Lee, T. W. (2011). The effects of autonomy and empowerment on employee turnover: Test of a multilevel model in teams. *Journal of Applied Psychology*, *96*(6), 1305. https://doi.org/10.1037/a0024518

Liu, H., Fan, J., Fu, Y., & Liu, F. (2018). Intrinsic motivation as a mediator of the relationship between organizational support and quantitative workload and work‐related fatigue. *Human Factors and Ergonomics in Manufacturing & Service Industries*, *28*(3), 154-162. https://doi.org/10.1002/hfm.20731

Longo, Y., Gunz, A., Curtis, G. J., & Farsides, T. (2016). Measuring need satisfaction and frustration in educational and work contexts: The Need Satisfaction and Frustration Scale (NSFS). *Journal of Happiness Studies*, *17*, 295-317. https://psycnet.apa.org/doi/10.1007/s10902-014-9595-3

Lopes, S., & Chambel, M. J. (2014). Motives for being temporary agency worker: Validity study of one measure according to the self-determination theory. *Social Indicators Research*, *116*, 137-152. https://psycnet.apa.org/doi/10.1007/s11205-013-0273-3

Lopes, S., & Chambel, M. J. (2017). Temporary agency workers’ motivations and well-being at work: A two-wave study. *International Journal of Stress Management*, *24*(4), 321. https://psycnet.apa.org/doi/10.1037/str0000041

Lopes, S., Chambel, M. J., & Cesário, F. (2019). Linking perceptions of organizational support to temporary agency workers’ well-being: The mediation of motivations. *International Journal of Organizational Analysis*, *27*(5), 1376-1391. http://dx.doi.org/10.1108/IJOA-08-2018-1502

Louka, A. (2011). The role of perceived relatedness in intrinsic need satisfaction: A gender differences study in the workplace.

Lu, L., Lin, H. Y., Lu, C. Q., & Siu, O. L. (2015). The moderating role of intrinsic work value orientation on the dual-process of job demands and resources among Chinese employees. *International Journal of Workplace Health Management*, *8*(2), 78-91. http://dx.doi.org/10.1108/IJWHM-11-2013-0045

Lynch Jr., M. F., Plant, R. W., & Ryan, R. M. (2005). Psychological needs and threat to safety: Implications for staff and patients in a psychiatric hospital for youth. *Professional Psychology: Research and Practice*, *36*(4), 415. https://psycnet.apa.org/doi/10.1037/0735-7028.36.4.415

Lyu, Y., & Zhu, H. (2019). The predictive effects of workplace ostracism on employee attitudes: A job embeddedness perspective. *Journal of Business Ethics*, *158*, 1083-1095. https://psycnet.apa.org/doi/10.1007/s10551-017-3741-x

Malhotra, N., Sahadev, S., & Sharom, N. Q. (2022). Organisational justice, organisational identification and job involvement: The mediating role of psychological need satisfaction and the moderating role of person-organisation fit. *The International Journal of Human Resource Management*, *33*(8), 1526-1561. https://doi.org/10.1080/09585192.2020.1757737

Malinowska, D., & Tokarz, A. (2020). The moderating role of Self Determination Theory's general causality orientations in the relationship between the job resources and work engagement of outsourcing sector employees. *Personality and Individual Differences*, *153*, 109638. https://doi.org/10.1016/j.paid.2019.109638

Malinowska, D., Tokarz, A., & Staszczyk, S. (2019). How to enhance work engagement among outsourcing sector employees: The role of job resources, intrinsic, and identified Motivation. *Journal of Occupational and Environmental Medicine*, *61*(9), e360-e366. https://doi.org/10.1097/jom.0000000000001649

Malinowska, D., Tokarz, A., & Wardzichowska, A. (2018). Job autonomy in relation to work engagement and workaholism: Mediation of autonomous and controlled work motivation. *International Journal of Occupational Medicine and Environmental Health*, *31*(4), 445-458. https://doi.org/10.13075/ijomeh.1896.01197

Menges, J. I., Tussing, D. V., Wihler, A., & Grant, A. M. (2017). When job performance is all relative: How family motivation energizes effort and compensates for intrinsic motivation. *Academy of Management Journal*, *60*(2), 695-719. https://psycnet.apa.org/doi/10.5465/amj.2014.0898

Merriman, K. K. (2017). Extrinsic work values and feedback: Contrary effects for performance and well-being. *Human Relations*, *70*(3), 339-361. https://doi.org/10.1177/0018726716655391

Mishra, M., & Ghosh, K. (2020). Supervisor monitoring and subordinate work attitudes: A need satisfaction and supervisory support perspective. *Leadership & Organization Development Journal*, *41*(8), 1089-1105. http://dx.doi.org/10.1108/LODJ-05-2019-0204

Mohammad, J., Quoquab, F., Halimah, S., & Thurasamy, R. (2019). Workplace internet leisure and employees’ productivity: The mediating role of employee satisfaction. *Internet Research*, *29*(4), 725-748. http://dx.doi.org/10.1108/IntR-05-2017-0191

Moon, T. W., Hur, W. M., & Hyun, S. S. (2019). How service employees’ work motivations lead to job performance: The role of service employees’ job creativity and customer orientation. *Current Psychology*, *38*, 517-532. https://psycnet.apa.org/doi/10.1007/s12144-017-9630-8

Moon, T. W., Youn, N., Hur, W. M., & Kim, K. M. (2020). Does employees’ spirituality enhance job performance? The mediating roles of intrinsic motivation and job crafting. *Current Psychology*, *39*, 1618-1634. https://psycnet.apa.org/doi/10.1007/s12144-018-9864-0

Moran, C. M., Diefendorff, J. M., Kim, T. Y., & Liu, Z. Q. (2012). A profile approach to self-determination theory motivations at work. *Journal of Vocational Behavior*, *81*(3), 354-363. https://doi.org/10.1016/j.jvb.2012.09.002

Mostafa, A. M. S. (2017). The mediating role of positive affect on the relationship between psychological empowerment and employee outcomes: A longitudinal study. *Evidence-based HRM: a Global Forum for Empirical Scholarship*, *5*(3). 266-282. http://dx.doi.org/10.1108/EBHRM-07-2016-0015

Mustafa, G., & Ali, N. (2019). Rewards, autonomous motivation and turnover intention: Results from a non-Western cultural context. *Cogent Business & Management*, *6*(1), 1676090. https://doi.org/10.1080/23311975.2019.1676090

Nerstad, C. G., Dysvik, A., Kuvaas, B., & Buch, R. (2018). Negative and positive synergies: On employee development practices, motivational climate, and employee outcomes. *Human Resource Management*, *57*(5), 1285-1302. https://doi.org/10.1002/hrm.21904

Nie, Y., Chua, B. L., Yeung, A. S., Ryan, R. M., & Chan, W. Y. (2015). The importance of autonomy support and the mediating role of work motivation for well‐being: Testing self‐determination theory in a Chinese work organisation. *International Journal of Psychology*, *50*(4), 245-255. https://psycnet.apa.org/doi/10.1002/ijop.12110

Nijhuis, N., van Beek, I., Taris, T., & Schaufeli, W. (2012). The motivation and performance of workaholic, engaged, and burned-out workers. *Gedrag en Organisatie*, *25*(4), 324-346. https://doi.org/10.5117/2012.025.004.325

Olafsen, A. H. (2017). The implications of need-satisfying work climates on state mindfulness in a longitudinal analysis of work outcomes. *Motivation and Emotion*, *41*(1), 22-37. https://link.springer.com/article/10.1007/s11031-016-9592-4

Olafsen, A. H., & Halvari, H. (2017). Motivational mechanisms in the relation between job characteristics and employee functioning. *The Spanish Journal of Psychology*, *20*, E38. https://doi.org/10.1017/sjp.2017.34

Olafsen, A. H., Niemiec, C. P., Halvari, H., Deci, E. L., & Williams, G. C. (2017). On the dark side of work: A longitudinal analysis using self-determination theory. *European Journal of Work and Organizational Psychology*, *26*(2), 275-285. https://psycnet.apa.org/doi/10.1080/1359432X.2016.1257611

Omansky, R., Eatough, E. M., & Fila, M. J. (2016). Illegitimate tasks as an impediment to job satisfaction and intrinsic motivation: Moderated mediation effects of gender and effort-reward imbalance. *Frontiers in Psychology*, *7*, 1818. https://doi.org/10.3389/fpsyg.2016.01818

Onyishi, I. E., Enwereuzor, I. K., Ogbonna, M. N., Ugwu, F. O., & Amazue, L. O. (2019). Role of career satisfaction in basic psychological needs satisfaction and career commitment of nurses in Nigeria: A self‐determination theory perspective. *Journal of Nursing Scholarship*, *51*(4), 470-479. https://doi.org/10.1111/jnu.12474

Park, R. (2018). The roles of OCB and automation in the relationship between job autonomy and organizational performance: A moderated mediation model. *The International Journal of Human Resource Management*, *29*(6), 1139-1156. https://doi.org/10.1080/09585192.2016.1180315

Park, S. M., & Rainey, H. G. (2012). Work motivation and social communication among public managers. *The International Journal of Human Resource Management*, *23*(13), 2630-2660. https://doi.org/10.1080/09585192.2011.637060

Parker, S. L., Jimmieson, N. L., & Amiot, C. E. (2010). Self-determination as a moderator of demands and control: Implications for employee strain and engagement. *Journal of Vocational Behavior*, *76*(1), 52-67. https://doi.org/10.1016/j.jvb.2009.06.010

Pearce, J. L. (1983). Job attitude and motivation differences between volunteers and employees from comparable organizations. *Journal of Applied Psychology*, *68*(4), 646. https://doi.org/10.1037/0021-9010.68.4.646

Potipiroon, W., & Ford, M. T. (2017). Does public service motivation always lead to organizational commitment? Examining the moderating roles of intrinsic motivation and ethical leadership. *Public Personnel Management*, *46*(3), 211-238. https://doi.org/10.1177/0091026017717241

Prokesova, L., Vaculik, M., Kasparkova, L., & Prochazka, J. (2019). An integrated model of work engagement: How the satisfaction of basic psychological needs explains the relationship between personality and work engagement. *Psihologija*, *52*(3), 265-284. http://dx.doi.org/10.2298/PSI181204004P

Putra, E. D., Cho, S., & Liu, J. (2017). Extrinsic and intrinsic motivation on work engagement in the hospitality industry: Test of motivation crowding theory. *Tourism and Hospitality Research*, *17*(2), 228-241. https://doi.org/10.1177/1467358415613393

Rahmadani, V. G., Schaufeli, W. B., Ivanova, T. Y., & Osin, E. N. (2019). Basic psychological need satisfaction mediates the relationship between engaging leadership and work engagement: A cross‐national study. *Human Resource Development Quarterly*, *30*(4), 453-471. https://doi.org/10.1002/hrdq.21366

Rahmadani, V. G., Schaufeli, W. B., & Stouten, J. (2020). How engaging leaders foster employees' work engagement. *Leadership & Organization Development Journal*, *41*(8), 1155-1169. http://dx.doi.org/10.1108/LODJ-01-2020-0014

Ramalu, S. S., & Subramaniam, C. (2019). Cultural intelligence and work engagement of expatriate academics: The role of psychological needs satisfaction. *International Journal of Cross Cultural Management*, *19*(1), 7-26. https://doi.org/10.1177/1470595819827992

Ranđelović, K., Stojiljković, S., & Milojević, M. (2013). Personal factors of burnout syndrome in teachers in the framework of self-determination theory. *Zbornik Instituta za Pedagoska Istrazivanja*, *45*(2), 260-281. http://dx.doi.org/10.2298/ZIPI1302260R

Raven, H., & Kleinert, J. (2019). Run away from burnout? Relations between job related need satisfaction, physical activity and burnout in teachers. *Psychologie in Erziehung Und Unterricht*, *66*(2), 101-117. https://doi.org/10.2378/peu2019.art10d

Rawolle, M., Wallis, M. S., Badham, R., & Kehr, H. M. (2016). No fit, no fun: The effect of motive incongruence on job burnout and the mediating role of intrinsic motivation. *Personality and Individual Differences*, *89*, 65-68. https://doi.org/10.1016/j.paid.2015.09.030

Reinardy, S. (2014). Autonomy and perceptions of work quality: Drive the job satisfaction of TV news workers. *Journalism Practice*, *8*(6), 855-870. http://hdl.handle.net/1808/19364

Reizer, A., Brender-Ilan, Y., & Sheaffer, Z. (2019). Employee motivation, emotions, and performance: A longitudinal diary study. *Journal of Managerial Psychology*, *34*(6), 415-428. https://doi.org/10.1108/JMP-07-2018-0299

Ren, T., Fang, R., & Yang, Z. (2017). The impact of pay-for-performance perception and pay level satisfaction on employee work attitudes and extra-role behaviors: An investigation of moderating effects. *Journal of Chinese Human Resource Management*, *8*(2), 94-113. http://dx.doi.org/10.1108/JCHRM-06-2015-0012

Renard, M., & Snelgar, R. J. (2017). Positive consequences of intrinsically rewarding work: A model to motivate, engage and retain non-profit employees. *Southern African Business Review*, *21*(1), 177-197. https://hdl.handle.net/10520/EJC-8e0defabe

Robijn, W., Euwema, M. C., Schaufeli, W. B., & Deprez, J. (2020). Leaders, teams and work engagement: A basic needs perspective. *Career Development International*, *25*(4), 373-388. https://doi.org/10.1108/CDI-06-2019-0150

Roche, M., & Haar, J. (2020). Motivations, work–family enrichment and job satisfaction: An indirect effects model. *Personnel Review*, *49*(3), 903-920. https://doi.org/10.1108/PR-06-2019-0289

Rubino, C., Luksyte, A., Perry, S. J., & Volpone, S. D. (2009). How do stressors lead to burnout? The mediating role of motivation. *Journal of Occupational Health Psychology*, *14*(3), 289. https://doi.org/10.1037/a0015284

Sagnak, M. (2016). Participative leadership and change-oriented organizational citizenship: The mediating effect of intrinsic motivation. *Eurasian Journal of Educational Research*, *16*(62). https://doi.org/10.14689/ejer.2016.62.11

Sandrin, É., Gillet, N., Fernet, C., Leloup, M., & Depin‐Rouault, C. (2019). Effects of motivation and workload on firefighters' perceived health, stress, and performance. *Stress and Health*, *35*(4), 447-456. https://doi.org/10.1002/smi.2873

Sawang, S., O’Connor, P. J., Kivits, R. A., & Jones, P. (2020). Business owner-managers’ job autonomy and job satisfaction: Up, down or no change? *Frontiers in Psychology*, *11*, 1506. https://doi.org/10.3389/fpsyg.2020.01506

Schopman, L. M., Kalshoven, K., & Boon, C. (2017). When health care workers perceive high-commitment HRM will they be motivated to continue working in health care? It may depend on their supervisor and intrinsic motivation. *The International Journal of Human Resource Management*, *28*(4), 657-677. https://doi.org/10.1080/09585192.2015.1109534

Schreurs, B., Van Emmerik, I. J., Van den Broeck, A., & Guenter, H. (2014). Work values and work engagement within teams: The mediating role of need satisfaction. *Group Dynamics: Theory, Research, and Practice*, *18*(4), 267. http://dx.doi.org/10.1037/gdn0000009

Schultz, P. P., Ryan, R. M., Niemiec, C. P., Legate, N., & Williams, G. C. (2015). Mindfulness, work climate, and psychological need satisfaction in employee well-being. *Mindfulness*, *6*, 971-985. https://doi.org/10.1007/s12671-014-0338-7

Shih, C. T., Chen, S. L., & Chao, M. (2022). How autonomy-supportive leaders’ influence employee service performance: A multilevel study. *The Service Industries Journal*, *42*(7-8), 630-651. https://doi.org/10.1080/02642069.2019.1691168

Shkoler, O., & Kimura, T. (2020). How does work motivation impact employees’ investment at work and their job engagement? A moderated-moderation perspective through an international lens. *Frontiers in Psychology*, *11*, 38. https://doi.org/10.3389%2Ffpsyg.2020.00038

Shuck, B., Peyton Roberts, T., & Zigarmi, D. (2018). Employee perceptions of the work environment, motivational outlooks, and employee work intentions: An HR practitioner’s dream or nightmare? *Advances in Developing Human Resources*, *20*(2), 197-213. https://doi.org/10.1177/1523422318757209

Skaalvik, E. M., & Skaalvik, S. (2014). Teacher self-efficacy and perceived autonomy: Relations with teacher engagement, job satisfaction, and emotional exhaustion. *Psychological Reports*, *114*(1), 68-77. https://doi.org/10.2466/14.02.PR0.114k14w0

Skiba, T., & Wildman, J. L. (2019). Uncertainty reducer, exchange deepener, or self-determination enhancer? Feeling trust versus feeling trusted in supervisor-subordinate relationships. *Journal of Business and Psychology*, *34*, 219-235. https://doi.org/10.1007/s10869-018-9537-x

Skipworth, C. M. (2016). *Motivation that impacts job satisfaction, involvement and commitment of men in nonprofits* (Doctoral dissertation, Capella University).

Slemp, G. R., & Vella-Brodrick, D. A. (2014). Optimising employee mental health: The relationship between intrinsic need satisfaction, job crafting, and employee well-being. *Journal of Happiness Studies*, *15*, 957-977. http://dx.doi.org/10.1007/s10902-013-9458-3

Slemp, G. R., Zhao, Y., Hou, H., & Vallerand, R. J. (2021). Job crafting, leader autonomy support, and passion for work: Testing a model in Australia and China. *Motivation and Emotion*, *45*(1), 60-74. https://doi.org/10.1007/s11031-020-09850-6

Smith, J. L., Handley, I. M., Rushing, S., Belou, R., Shanahan, E. A., Skewes, M. C., Kambich, L., Honea, J., & Intemann, K. (2018). Added benefits: How supporting women faculty in STEM improves everyone’s job satisfaction. *Journal of Diversity in Higher Education*, *11*(4), 502. http://dx.doi.org/10.1037/dhe0000066

Steinbauer, R., Renn, R. W., Chen, H. S., & Rhew, N. (2018). Workplace ostracism, self-regulation, and job performance: Moderating role of intrinsic work motivation. *The Journal of Social Psychology*, *158*(6), 767-783.

Stokowski, S., Li, B., Goss, B. D., Hutchens, S., & Turk, M. (2018). Work motivation and job satisfaction of sport management faculty members. *Sport Management Education Journal*, *12*(2), 80-89. http://dx.doi.org/10.1123/smej.2017-0011

Strauss, K., Parker, S. K., & O'Shea, D. (2017). When does proactivity have a cost? Motivation at work moderates the effects of proactive work behavior on employee job strain. *Journal of Vocational Behavior*, *100*, 15-26. https://doi.org/10.1016/j.jvb.2017.02.001

Sun, P. C., Pan, F. T., & Ho, C. W. (2016). Does motivating language matter in leader–subordinate communication? *Chinese Journal of Communication*, *9*(3), 264-282. https://doi.org/10.1080/17544750.2016.1206029

Tadić Vujčić, M., Oerlemans, W. G., & Bakker, A. B. (2017). How challenging was your work today? The role of autonomous work motivation. *European Journal of Work and Organizational Psychology*, *26*(1), 81-93. https://doi.org/10.1080/1359432X.2016.1208653

ten Brummelhuis, L. L., Ter Hoeven, C. L., Bakker, A. B., & Peper, B. (2011). Breaking through the loss cycle of burnout: The role of motivation. *Journal of Occupational and Organizational Psychology*, *84*(2), 268-287. http://dx.doi.org/10.1111/j.2044-8325.2011.02019.x

Thatcher, J. B., Liu, Y., Stepina, L. P., Goodman, J. M., & Treadway, D. C. (2006). IT worker turnover: An empirical examination of intrinsic motivation. *Database for Advances in Information Systems*, *37*(2-3), 133-146. http://dx.doi.org/10.1145/1161345.1161361

Thibault-Landry, A., Egan, R., Crevier-Braud, L., Manganelli, L., & Forest, J. (2018). An empirical investigation of the employee work passion appraisal model using self-determination theory. *Advances in Developing Human Resources*, *20*(2), 148-168. https://doi.org/10.1177/1523422318756636

Trépanier, S. G., Fernet, C., & Austin, S. (2013). The moderating role of autonomous motivation in the job demands-strain relation: A two sample study. *Motivation and Emotion*, *37*, 93-105. https://doi.org/10.1007/s11031-012-9290-9

Trépanier, S. G., Fernet, C., & Austin, S. (2013). Workplace bullying and psychological health at work: The mediating role of satisfaction of needs for autonomy, competence and relatedness. *Work & Stress*, *27*(2), 123-140. https://doi.org/10.1080/02678373.2013.782158

Trépanier, S. G., Fernet, C., & Austin, S. (2015). A longitudinal investigation of workplace bullying, basic need satisfaction, and employee functioning. *Journal of Occupational Health Psychology*, *20*(1), 105. https://doi.org/10.1037/a0037726

Trépanier, S. G., Forest, J., Fernet, C., & Austin, S. (2015). On the psychological and motivational processes linking job characteristics to employee functioning: Insights from self-determination theory. *Work & Stress*, *29*(3), 286-305. http://dx.doi.org/10.1080/02678373.2015.1074957

Trépanier, S. G., Vallerand, R. J., Ménard, J., & Peterson, C. (2020). Job resources and burnout: Work motivation as a moderator. *Stress and Health*, *36*(4), 433-441. https://doi.org/10.1002/smi.2939

Tummers, G. E., Van Merode, G. G., & Landeweerd, J. A. (2006). Organizational characteristics as predictors of nurses’ psychological work reactions. *Organization Studies*, *27*(4), 559-584. https://doi.org/10.1177/0170840605059455

Tyagi, P. K. (1985). Relative importance of key job dimensions and leadership behaviors in motivating salesperson work performance. *Journal of Marketing*, *49*(3), 76-86. https://doi.org/10.2307/1251617

Unanue, W., Gómez, M. E., Cortez, D., Oyanedel, J. C., & Mendiburo-Seguel, A. (2017). Revisiting the link between job satisfaction and life satisfaction: The role of basic psychological needs. *Frontiers in Psychology*, *8*, 680. https://doi.org/10.3389/fpsyg.2017.00680

Unanue, W., Rempel, K., Gómez, M. E., & Van den Broeck, A. (2017). When and why does materialism relate to employees’ attitudes and well-being: The mediational role of need satisfaction and need frustration. *Frontiers in Psychology*, *8*, 1755. https://doi.org/10.3389/fpsyg.2017.01755

Van Beek, I., Hu, Q., Schaufeli, W. B., Taris, T. W., & Schreurs, B. H. (2012). For fun, love, or money: What drives workaholic, engaged, and burned‐out employees at work? *Applied Psychology*, *61*(1), 30-55. https://doi.org/10.1111/j.1464-0597.2011.00454.x

Van den Berg, P. T. (2011). Characteristics of the work environment related to older employees' willingness to continue working: Intrinsic motivation as a mediator. *Psychological Reports*, *109*(1), 174-186. https://doi.org/10.2466/01.09.10.PR0.109.4.174-186

Van den Broeck, A., Lens, W., De Witte, H., & Van Coillie, H. (2013). Unraveling the importance of the quantity and the quality of workers’ motivation for well-being: A person-centered perspective. *Journal of Vocational Behavior*, *82*(1), 69-78. https://doi.org/10.1016/j.jvb.2012.11.005

Van den Broeck, A., Schreurs, B., De Witte, H., Vansteenkiste, M., Germeys, F., & Schaufeli, W. (2011). Understanding workaholics' motivations: A self‐determination perspective. *Applied Psychology*, *60*(4), 600-621. https://doi.org/10.1111/j.1464-0597.2011.00449.x

Van den Broeck, A., Schreurs, B., Guenter, H., & van Emmerik, I. H. (2015). Skill utilization and well-being: A cross-level story of day-to-day fluctuations and personal intrinsic values. *Work & Stress*, *29*(3), 306-323. http://dx.doi.org/10.1080/02678373.2015.1074955

Van den Broeck, A., Van Ruysseveldt, J., Smulders, P., & De Witte, H. (2011). Does an intrinsic work value orientation strengthen the impact of job resources? A perspective from the Job Demands–Resources Model. *European Journal of Work and Organizational Psychology*, *20*(5), 581-609. https://doi.org/10.1080/13594321003669053

van der Kolk, B., van Veen-Dirks, P. M., & ter Bogt, H. J. (2019). The impact of management control on employee motivation and performance in the public sector. *European Accounting Review*, *28*(5), 901-928. https://doi.org/10.1080/09638180.2018.1553728

van Den Berg, P. T. (2011). Characteristics of the work environment related to older employees' willingness to continue working: Intrinsic motivation as a mediator. *Psychological Reports*, *109*(1), 174-186. https://doi.org/10.2466/01.09.10.PR0.109.4.174-186

van Dorssen-Boog, P., De Jong, J., Veld, M., & Van Vuuren, T. (2020). Self-leadership among healthcare workers: A mediator for the effects of job autonomy on work engagement and health. *Frontiers in Psychology*, *11*, 1420. https://doi.org/10.3389%2Ffpsyg.2020.01420

Van Tuin, L., Schaufeli, W. B., & Van Rhenen, W. (2020). The satisfaction and frustration of basic psychological needs in engaging leadership. *Journal of Leadership Studies*, *14*(2), 6-23. https://doi.org/10.1002/jls.21695

Van Wingerden, J., Derks, D., & Bakker, A. B. (2018). Facilitating interns’ performance: The role of job resources, basic need satisfaction and work engagement. *Career Development International*, *23*(4), 382-396. https://doi.org/10.1108/CDI-12-2017-0237

Vander Elst, T., Van den Broeck, A., De Witte, H., & De Cuyper, N. (2012). The mediating role of frustration of psychological needs in the relationship between job insecurity and work-related well-being. *Work & Stress*, *26*(3), 252-271. https://doi.org/10.1080/02678373.2012.703900

Vansteenkiste, M., Neyrinck, B., Niemiec, C. P., Soenens, B., De Witte, H., & Van den Broeck, A. (2007). On the relations among work value orientations, psychological need satisfaction and job outcomes: A self‐determination theory approach. *Journal of Occupational and Organizational Psychology*, *80*(2), 251-277. http://dx.doi.org/10.1348/096317906X111024

Victor, J., & Hoole, C. (2017). The influence of organisational rewards on workplace trust and work engagement. *SA Journal of Human Resource Management*, *15*(1), 1-14. http://dx.doi.org/10.4102/sajhrm.v15i0.853

Vîrgă, D., Pascu, D. M., Mioc, M., Draguț, I. E., Țepeș-Onea, A., & Petrucă, E. (2013). The role of personal resources in well-being of the employees: work engagement and burnout. *Psihologia Resurselor Umane*, *11*(1), 51-64. https://www.hrp-journal.com/index.php/pru/article/view/193

Walczak, R. B. (2014). Psychological Predictors of Employee Happiness. *Knowledge for Market Use*, 429-437.

Wallace, J. E., & Brinkerhoff, M. B. (1991). The measurement of burnout revisited. *Journal of Social Service Research*, *14*(1-2), 85-111. https://doi.org/10.1300/J079v14n01_05

Wang, E., Hu, H., Mao, S., & Liu, H. (2019). Intrinsic motivation and turnover intention among geriatric nurses employed in nursing homes: The roles of job burnout and pay satisfaction. *Contemporary Nurse*, *55*(2-3), 195-210. https://doi.org/10.1080/10376178.2019.1641120

Wang, N., Zhu, J., Dormann, C., Song, Z., & Bakker, A. B. (2020). The daily motivators: Positive work events, psychological needs satisfaction, and work engagement. *Applied Psychology*, *69*(2), 508-537. https://doi.org/10.1111/apps.12182

Wang, Z., Chen, L., Duan, Y., & Du, J. (2018). Supervisory mentoring and newcomers' work engagement: The mediating role of basic psychological need satisfaction. *Social Behavior and Personality: An International Journal*, *46*(10), 1745-1760. https://doi.org/10.2224/sbp.7609

Williams, G. C., Halvari, H., Niemiec, C. P., Sørebø, Ø., Olafsen, A. H., & Westbye, C. (2014). Managerial support for basic psychological needs, somatic symptom burden and work-related correlates: A self-determination theory perspective. *Work & Stress*, *28*(4), 404-419. https://doi.org/10.1080/02678373.2014.971920

Wininger, S. R., & Birkholz, P. M. (2013). Sources of instructional feedback, job satisfaction, and basic psychological needs. *Innovative Higher Education*, *38*, 159-170. https://doi.org/10.1007/s10755-012-9229-9

Winter-Collins, A., & McDaniel, A. M. (2000). Sense of belonging and new graduate job satisfaction. *Journal for Nurses in Professional Development*, *16*(3), 103-111. https://doi.org/10.1097/00124645-200005000-00002

Xie, T., Shi, Y. N., & Zhou, J. (2016). The adverse effect of materialism on employee engagement in China. *Journal of Chinese Human Resource Management*, *7*(2), 100-114. https://doi.org/10.1108/JCHRM-07-2016-0013

Yan Lin, & Takashi Horiuchi. (2019). Part-time job satisfaction among international students in Japan: An examination based on self-determination theory. *Psychological Research*, *90*(2), 178-186. https://doi.org/10.4992/jjpsy.90.18203

Yang, J., Yang, F., & Gao, N. (2022). Enhancing career satisfaction: The roles of spiritual leadership, basic need satisfaction, and power distance orientation. *Current Psychology*, *41*(4), 1856-1867. https://doi.org/10.1007/s12144-020-00712-5

Yoon, D. J. (2022). Rude customers and service performance: Roles of motivation and personality. *The Service Industries Journal*, *42*(1-2), 81-106. https://doi.org/10.1080/02642069.2020.1826453

Yoon, H. J., Sung, S. Y., Choi, J. N., Lee, K., & Kim, S. (2015). Tangible and intangible rewards and employee creativity: The mediating role of situational extrinsic motivation. *Creativity Research Journal*, *27*(4), 383-393. https://doi.org/10.1080/10400419.2015.1088283

Young, G. J., Beckman, H., & Baker, E. (2012). Financial incentives, professional values and performance: A study of pay‐for‐performance in a professional organization. *Journal of Organizational Behavior*, *33*(7), 964-983. https://doi.org/10.1002/job.1770

Zeijen, M. E., Petrou, P., Bakker, A. B., & Van Gelderen, B. R. (2020). Dyadic support exchange and work engagement: An episodic test and expansion of self‐determination theory. *Journal of Occupational and Organizational Psychology*, *93*(3), 687-711. https://doi.org/10.1111/joop.12311

Zhang, J., Zhang, Y., Song, Y., & Gong, Z. (2016). The different relations of extrinsic, introjected, identified regulation and intrinsic motivation on employees’ performance: Empirical studies following self-determination theory. *Management Decision*, *54*(10), 2393-2412. http://dx.doi.org/10.1108/MD-01-2016-0007

Zhou, Q., Li, Q., & Gong, S. (2019). How job autonomy promotes employee’s sustainable development? A moderated mediation model. *Sustainability*, *11*(22), 6445. https://doi.org/10.3390/su11226445

Zhao, X. R., Ghiselli, R., Law, R., & Ma, J. (2016). Motivating frontline employees: Role of job characteristics in work and life satisfaction. *Journal of Hospitality and Tourism Management*, *27*, 27-38. https://doi.org/10.1016/j.jhtm.2016.01.010

Supplement F

**Table F1**

*Summary Characteristics and Covariate and Moderator Coding of Studies Included in Meta-Analysis*

| Author | Study | Year | Within measures^a^ | Study  type | Pub. status | *N* | Sample age (*M* SD) | Age code | Sample sex^b^ | Sex code^d^ | Quality | Employee  code^f^ | Sample country | Country  code^g^ | Culture code^h^ | LAS prox. code^i^ |
| --- | --- | --- | --- | --- | --- | --- | --- | --- | --- | --- | --- | --- | --- | --- | --- | --- |
| Chowdhury, M. S. | 1 | 2008 |  | 0 | 0 | 105 | 64.2%<30, 26.9% 31-40 | 0 | 60.6% female | 2 | 5 | 0 | United States | 0 | 0 |  |
| Chung-Yan, G. A. | 2 | 2010 |  | 0 | 0 | 259 | 38.3 (SD=10.9), range=19-65 | 0 | 50.9% female | 2 | 5.8 | 3 | United States | 0 | 0 |  |
| Clayton, B. C. | 3 | 2015 |  | 0 | 0 | 306 | range 18-45+ | 2 | 17% female | 1 | 6.3 | 0 | United States | 0 | 0 |  |
| Collie, R. J.. | 4 | 2015 | WB: General, REL: colleagues | 0 | 0 | 485 | 44 (SD=11) | 1 | 76% female | 0 | 5.5 | 2 | Canada | 0 | 0 | 0 |
| Collie, R. J.. | 4 | 2015 | WB: General, REL: students | 0 | 0 | 485 | 45 (SD=11) | 1 | 76% female | 0 | 5.5 | 2 | Canada | 0 | 0 |  |
| Collie, R. J.. | 4 | 2015 | WB: Teacher, REL: students | 0 | 0 | 485 | 46 (SD=11) | 1 | 76% female | 0 | 5.5 | 2 | Canada | 0 | 0 | 0 |
| Collie, R. J.. | 4 | 2015 | WB: Teacher, REL: colleagues | 0 | 0 | 485 | 47 (SD=11) | 1 | 76% female | 0 | 5.5 | 2 | Australia | 0 | 0 |  |
| Collie, R. J.. | 5 | 2018 |  | 0 | 0 | 164 | 41.4 (SD=10.16), | 1 | 65% female | 2 | 6.5 | 2 | Australia | 1 | 0 | 0 |
| Cross, W. | 6 | 2006 |  | 0 | 0 | 128 | 48 (SD=10), range= 21-74 | 1 | 98% female | 0 | 6.5 | 1 | United States | 0 | 0 |  |
| Cummings, T. G | 7 | 1976 |  | 0 | 0 | 96 | 41.2 (SD=16.4), | 1 | 100% male | 1 | 2.1 | 3 | United States | 0 | 0 |  |
| Dagenais-Desmarais, V. | 8 | 2018 | Time 1 | 1 | 0 | 686 | 41 (SD=12), range= | 1 | 66% female | 2 | 6.8 | 3 | Canada | 0 | 0 |  |
| Dagenais-Desmarais, V. | 8 | 2018 | Time 2 | 1 | 0 | 686 | 42 (SD=12), range= | 1 | 66% female | 2 | 6.8 | 3 | Canada | 0 | 0 |  |
| Dahling, J. J. | 9 | 2017 | NSUP: autonomy support | 1 | 0 | 205 | 34.46 (SD=9.54), range= | 0 | 48.3% female | 2 | 7.5 | 3 | United States | 0 | 0 | 0 |
| Dahling, J. J. | 9 | 2017 | NSUP: competence support | 1 | 0 | 205 | 34.46 (SD=9.54), range= | 0 | 48.3% female | 2 | 7.5 | 3 | United States | 0 | 0 | 0 |
| Dahling, J. J. | 9 | 2017 | NSUP: need satisfaction | 1 | 0 | 205 | 34.46 (SD=9.54), range= | 0 | 48.3% female | 2 | 7.5 | 3 | United States | 0 | 0 | 0 |
| Dai, B. | 10 | 2020 |  | 0 | 0 | 385 | 33% 20-30, 34% 31-40, 24% 41-50 | 0 | 65 % female | 2 | 6.5 | 1 | Ghana | 1 | 1 |  |
| Danish, R. Q. | 11 | 2015 |  | 0 | 0 | 290 | NR | 2 | NR | 2 | 2.6 | 0 | Pakistan | 1 | 1 |  |
| De Klerk, J. J. | 12 | 2001 |  | 0 | 0 | 458 | 42.96 (SD=8.31) | 1 | 9.83% female | 1 | 7.9 | 3 | South Africa | 1 | 1 |  |
| Deci, E. L. | 13 | 2001 | POP: Bulgarian, AUT: autonomy NSUP: supervisor autonomy need support | 0 | 0 | 431 | NR | 2 | NR | 2 | 5 | 3 | United States, Bulgaria | 1 | 0 | 2 |
| Deci, E. L. | 13 | 2001 | POP: Bulgarian, AUT: autonomy NSUP: top management autonomy need support | 0 | 0 | 431 | NR | 2 | NR | 2 | 5 | 3 | United States, Bulgaria | 1 | 0 | 2 |
| Deci, E. L. | 13 | 2001 | POP: Bulgarian, AUT: autonomy NSUP: total need support | 0 | 0 | 431 | NR | 2 | NR | 2 | 5 | 3 | United States, Bulgaria | 1 | 0 | 2 |
| Deci, E. L. | 13 | 2001 | POP: Bulgarian, AUT: total need satisfaction NSUP: supervisor total autonomy support | 0 | 0 | 431 | NR | 2 | NR | 2 | 5 | 3 | United States, Bulgaria | 1 | 0 | 2 |
| Deci, E. L. | 13 | 2001 | POP: Bulgarian, AUT: total need satisfaction NSUP: top management autonomy support | 0 | 0 | 431 | NR | 2 | NR | 2 | 5 | 3 | United States, Bulgaria | 1 | 0 | 2 |
| Deci, E. L. | 13 | 2001 | POP: Bulgarian, AUT: total need satisfaction NSUP: total need support | 0 | 0 | 431 | NR | 2 | NR | 2 | 5 | 3 | United States, Bulgaria | 1 | 0 | 2 |
| Deci, E. L. | 13 | 2001 | POP: US, AUT: autonomy NSUP: supervisor autonomy need support | 0 | 0 | 128 | NR | 2 | NR | 2 | 5 | 3 | United States, Bulgaria | 0 | 0 | 2 |
| Deci, E. L. | 13 | 2001 | POP: US, AUT: autonomy NSUP: top management autonomy need support | 0 | 0 | 128 | NR | 2 | NR | 2 | 5 | 3 | United States, Bulgaria | 0 | 0 | 2 |
| Deci, E. L. | 13 | 2001 | POP: US, AUT: autonomy NSUP: total need support | 0 | 0 | 128 | NR | 2 | NR | 2 | 5 | 3 | United States, Bulgaria | 0 | 0 | 2 |
| Deci, E. L. | 13 | 2001 | POP: US, AUT: total need satisfaction NSUP: supervisor total autonomy support | 0 | 0 | 128 | NR | 2 | NR | 2 | 5 | 3 | United States, Bulgaria | 0 | 0 | 2 |
| Deci, E. L. | 13 | 2001 | POP: US, AUT: total need satisfaction NSUP: top management autonomy support | 0 | 0 | 128 | NR | 2 | NR | 2 | 5 | 3 | United States, Bulgaria | 0 | 0 | 2 |
| Deci, E. L. | 13 | 2001 | POP: US, AUT: total need satisfaction NSUP: total need support | 0 | 0 | 128 | NR | 2 | NR | 2 | 5 | 3 | United States, Bulgaria | 0 | 0 | 2 |
| Demircioglu, M. A. | 14 | 2020 |  | 0 | 0 | 4325 | NR | 2 | NR | 2 | 5.8 | 0 | Australia | 1 | 0 |  |
| Demircioglu, M. A. | 15 | 2018 |  | 0 | 0 | 8705 | NR | 2 | NR | 2 | 5.3 | 0 | Australia | 1 | 0 |  |
| den Broeck, A | 16 | 2012 | S1 | 0 | 0 | 1797 | NR | 2 | NR | 2 | 4.7 | 0 | Belgium | 1 | 0 |  |
| den Broeck, A | 16 | 2012 | S2 | 0 | 0 | 287 | 43.39 (SD=9.10) | 1 | 47% female | 2 | 4.7 | 0 | Belgium | 1 | 0 |  |
| den Broeck, A | 16 | 2012 | S3 | 0 | 0 | 270 | 8.24 (SD=9.01) | 0 | 54% female | 2 | 4.7 | 0 | Belgium | 1 | 0 |  |
| den Broeck, A | 17 | 2015 |  | 0 | 0 | 495 | 39.7 (SD = 10.4) | 0 | 74% female | 2 | 5 | 3 | Belgium | 1 | 0 |  |
| Van den Broeck, A | 18 | 2004 | JP= Manager rating | 0 | 0 | 1760 | 42.6% less than 29 years of age, 35.2% betweenthe ages of 30–39, 13.4% between the ages of 40–49, and7.4% over 50 | 0 | 64.1% female | 2 | 7.8 | 3 | Belgium | 1 | 0 |  |
| Van den Broeck, A | 18 | 2004 | JP= HR rating | 0 | 0 | 1760 | 42.6% less than 29 years of age, 35.2% betweenthe ages of 30–39, 13.4% between the ages of 40–49, and7.4% over 50 | 0 | 64.1% female | 2 | 7.8 | 3 | Belgium | 1 | 0 |  |
| Djordjeviƒá, D. | 19 | 2015 |  | 0 | 0 | 221 | 40.1 (SD=11.4), 77% younger than 50 | 0 | 75% female | 0 | 5.3 | 1 | Serbia | 1 | 1 |  |
| Dysvik, A. | 20 | 2008 |  | 0 | 0 | 343 | 40 (SD=10.22) | 0 | 30% female | 2 | 3.5 | 0 | Norway | 1 | 0 |  |
| Dysvik, A. | 21 | 2010 |  | 0 | 0 | 199 | not reported | 2 | 43.2% female | 2 | 4.7 | 3 | Norway | 1 | 0 |  |
| Dysvik, A. | 22 | 2013 |  | 1 | 0 | 1441 | not reported | 2 | 39.8% female | 2 | 5.8 | 3 | Norway | 1 | 0 |  |
| Dysvik, A. | 23 | 2010 |  | 0 | 0 | 343 | 40 (SD=10.22) | 0 | 30% female | 2 | 4.7 | 0 | Norway | 1 | 0 |  |
| Dysvik, A. | 24 | 2010 |  | 0 | 0 | 114 | 27.29 (SD=1.60) | 0 | 36.8% female | 2 | 5.3 | 3 | Norway | 1 | 0 |  |
| Elias, S. M. | 25 | 2011 |  | 0 | 0 | 671 | 39.47(SD=11.43) | 0 | 52.6% female | 2 | 6.8 | 3 | United States | 0 | 0 |  |
| Eyal, O. | 26 | 2011 |  | 0 | 0 | 122 | 39 (SD=8.80) | 0 | 87.7% female | 0 | 6 | 2 | Israel | 1 | 0 |  |
| Falvo, R | 27 | 2016 |  | 0 | 0 | 120 | 31 and 40 (31.7%), 41 and 50 years (26.7%); 18.3% 30 years,17.5% over 50,(5.8% did not indicate their age) | 2 | 100% male | 1 | 5.5 | 1 | Italy | 0 | 0 |  |
| Fernet, C. | 28 | 2012 |  | 0 | 0 | 356 | 25.9 (SD=6.3) | 1 | 87.8% female | 0 | 5.3 | 2 | Canada | 0 | 0 |  |
| Fernet, C. | 29 | 2012 |  | 0 | 0 | 586 | 41.8 (SD=10.4) | 1 | 76% female | 2 | 6.3 | 2 | Canada | 0 | 0 | 2 |
| Fernet, C. | 29 | 2012 |  | 0 | 0 | 586 | 45.2 (SD=7.02) | 1 | 58% female | 2 | 6.3 | 2 | Canada | 0 | 0 | 2 |
| Fernet, C. | 30 | 2009 | Time 1 | 1 | 0 | 380 | 45.2 (SD=7.02) | 1 | 58% female | 2 | 5.8 | 2 | Canada | 0 | 0 |  |
| Fernet, C. | 30 | 2009 | Time 2 | 1 | 0 | 276 | 41.5 (SD=10.4) | 1 | 80.1% female | 2 | 5.8 | 2 | Canada | 0 | 0 |  |
| Fernet, C. | 31 | 2011 |  | 0 | 0 | 806 | 45.1 (SD=8.28) | 1 | 57% female | 0 | 7 | 2 | Canada | 0 | 0 |  |
| Fernet, C. | 32 | 2020 | Time 1 | 1 | 0 | 660 | 44.3 (SD=9.15) | 0 | 64% female | 0 | 5.5 | 1 | Canada | 0 | 0 |  |
| Fernet, C. | 32 | 2020 | Time 2 | 1 | 0 | 660 | 41.5 (SD=10.4) | 0 | 80.1% female | 0 | 5.5 | 1 | Canada | 0 | 0 |  |
| Fernet, C. | 32 | 2020 | IM Time 1, TU= Affective commitment Time 2 | 1 | 0 | 660 | 26.7 (SD=6.67) | 0 | 87.9% female | 0 | 5.5 | 1 | Canada | 0 | 0 |  |
| Fernet, C. | 32 | 2020 | IM Time 1, TU= Affective commitment Time 3 | 1 | 0 | 660 | 26.7 (SD=6.67) | 0 | 87.9% female | 0 | 5.5 | 1 | Canada | 0 | 0 |  |
| Fernet, C. | 32 | 2020 | IM Time 1, TU= Affective commitment Time 4 | 1 | 0 | 660 | 26.7 (SD=6.67) | 0 | 87.9% female | 0 | 5.5 | 1 | Canada | 0 | 0 |  |
| Fernet, C. | 32 | 2020 | IM Time 2, TU= Affective commitment Time 3 | 1 | 0 | 660 | 26.7 (SD=6.67) | 0 | 87.9% female | 0 | 5.5 | 1 | Canada | 0 | 0 |  |
| Fernet, C. | 32 | 2020 | IM Time 2, TU= Affective commitment Time 4 | 1 | 0 | 660 | 26.7 (SD=6.67) | 0 | 87.9% female | 0 | 5.5 | 1 | Canada | 0 | 0 |  |
| Fernet, C. | 32 | 2020 | IM Time 3, TU= Affective commitment Time 4 | 1 | 0 | 660 | 26.7 (SD=6.67) | 0 | 87.9% female | 0 | 5.5 | 1 | Canada | 0 | 0 |  |
| Fernet, C. | 32 | 2020 | IM Time 1, TU= Intention to leave Time 2 | 1 | 0 | 660 | 26.7 (SD=6.67) | 0 | 87.9% female | 0 | 5.5 | 1 | Canada | 0 | 0 |  |
| Fernet, C. | 32 | 2020 | IM Time 1, TU= Intention to leave Time 3 | 1 | 0 | 660 | 26.7 (SD=6.67) | 0 | 87.9% female | 0 | 5.5 | 1 | Canada | 0 | 0 |  |
| Fernet, C. | 32 | 2020 | IM Time 1, TU= Intention to leave Time 4 | 1 | 0 | 660 | 26.7 (SD=6.67) | 0 | 87.9% female | 0 | 5.5 | 1 | Canada | 0 | 0 |  |
| Fernet, C. | 32 | 2020 | IM Time 2, TU= Intention to leave Time 3 | 1 | 0 | 660 | 26.7 (SD=6.67) | 0 | 87.9% female | 0 | 5.5 | 1 | Canada | 0 | 0 |  |
| Fernet, C. | 33 | 2016 | IM Time 2, TU= Intention to leave Time 4 | 0 | 0 | 589 | 26.7 (SD=6.67) | 0 | 87.9% female | 0 | 6.3 | 2 | Canada | 0 | 0 | 2 |
| Fernet, C. | 34 | 2017 | TU: affective organizational commitment | 0 | 0 | 572 | 26.7 (SD=6.67) | 2 | 87.9% female | 2 | 5.3 | 1 | Canada | 0 | 0 |  |
| Fernet, C. | 34 | 2017 | TU: continuance organizational commitment | 0 | 0 | 572 | 26.36 (SD=3.44) | 2 | 85.7% female | 2 | 5.3 | 1 | Canada | 0 | 0 |  |
| Fernet, C. | 34 | 2017 | TU: organizational turnover intention | 0 | 0 | 572 | NR | 2 | NR | 2 | 5.3 | 1 | Canada | 0 | 0 |  |
| Foulk, T. A | 35 | 2019 |  | 1 | 0 | 79 | 34.31 (SD=4.76) | 0 | 6.3% Female | 1 | 5.3 | 0 | United States | 0 | 0 | 0 |
| Galletta, M. | 36 | 2012 |  | 0 | 0 | 173 | 41.07(SD=6.56, range=25–64) | 1 | 70.4% female | 2 | 4.2 | 1 | Italy | 0 | 0 |  |
| Galletta, M. | 37 | 2016 |  | 0 | 0 | 304 | women 34.7 (SD = 8.11)men 36.5 (SD=8.47) (range 23-56 years) | 0 | 83.9% female | 0 | 6 | 1 | Italy | 0 | 0 |  |
| Galletta, M. | 38 | 2019 | T1: affective commitment | 1 | 0 | 221 | 37.84 (SD = 8.16), range = 23–57 | 0 | 77.2% female | 0 | 8 | 1 | Italy | 0 | 0 |  |
| Galletta, M. | 38 | 2019 | T1: continuance commitment | 1 | 0 | 221 | 37.84 (SD = 8.16), range = 23–58 | 0 | 77.2% female | 0 | 8 | 1 | Italy | 0 | 0 |  |
| Galletta, M. | 38 | 2019 | T2: affective commitment | 1 | 0 | 221 | 37.84 (SD = 8.16), range = 23–59 | 0 | 77.2% female | 0 | 8 | 1 | Italy | 0 | 0 |  |
| Galletta, M. | 38 | 2019 | T2: continuance commitment | 1 | 0 | 221 | 37.84 (SD = 8.16), range = 23–60 | 0 | 77.2% female | 0 | 8 | 1 | Italy | 0 | 0 |  |
| Garcia-Chas, R | 39 | 2016 |  | 0 | 0 | 180 | 35.2 (SD=6.5) | 0 | 26.1% female | 2 | 5.3 | 0 | Spain | 1 | 0 | 1 |
| Gatt, G. | 40 | 2020 |  | 0 | 0 | 127 | 18 and 74 range 35 to 44 years old (35.5%). 54 years old or younger (93.7%). | 2 | 47.2% female | 2 | 7.9 | 3 | New Zealand | 1 | 0 |  |
| Gillet, N. | 41 | 2013 | AUT: need satisfaction, TU: affective commitment | 0 | 0 | 323 | 36.28 (SD= 10.31) | 0 | 94.7% female | 0 | 5.3 | 1 | France | 0 | 0 | 0 |
| Gillet, N. | 42 | 2015 | AUT: need thwarting, TU: affective commitment | 0 | 0 | 129 | 39 (SD=6.56), range 25-57 | 0 | 39.5% female | 2 | 3.2 | 0 | Canada | 0 | 0 | 0 |
| Gillet, N. | 42 | 2015 | AUT: need satisfaction, TU: tu intention | 0 | 0 | 129 | 40 (SD=6.56), range 25-57 | 0 | 39.5% female | 2 | 3.2 | 0 | Canada | 0 | 0 | 0 |
| Gillet, N. | 42 | 2015 | AUT: need thwarting, TU: tu intention | 0 | 0 | 129 | 41 (SD=6.56), range 25-57 | 0 | 39.5% female | 2 | 3.2 | 0 | Canada | 0 | 0 | 0 |
| Gillet, N. | 42 | 2015 | AUT: autonomy satisfaction | 0 | 0 | 129 | 42 (SD=6.56), range 25-57 | 0 | 39.5% female | 2 | 3.2 | 0 | Canada | 0 | 0 |  |
| Gillet, N. | 43 | 2015 | AUT: need satisfaction | 0 | 0 | 461 | 34.69 (SD=10.48) , range=18-69 | 0 | 58.7% female | 2 | 5.3 | 0 | France | 0 | 0 |  |
| Gillet, N. | 43 | 2015 |  | 0 | 0 | 461 | 34.69 (SD=10.48) , range=18-70 | 0 | 58.7% female | 2 | 5.3 | 0 | France | 0 | 0 |  |
| Gillet, N. | 44 | 2017 | Global IM & EM | 0 | 0 | 328 | 43.34 (SD=9.13), range= 18-62 | 1 | 62.5% female | 2 | 5.5 | 0 | France | 0 | 0 | 1 |
| Gillet, N. | 45 | 2012 | Work IM & EM | 0 | 0 | 735 | 35.98 (SD=10.82), range= 18-65 | 0 | 50.7% female | 2 | 5.3 | 3 | France | 0 | 0 | 1 |
| Gillet, N. | 45 | 2012 | WE: Vigour | 0 | 0 | 735 | 35.98 (SD=10.82), range= 18-66 | 0 | 50.7% female | 2 | 5.3 | 3 | France | 0 | 0 | 1 |
| Gillet, N. | 46 | 2019 | WE: dedication | 1 | 0 | 294 | 37.63 (SD=10.02) | 0 | 81.3% female | 0 | 7.5 | 1 | France | 0 | 0 |  |
| Gillet, N. | 46 | 2019 | WE: absorption | 1 | 0 | 294 | 37.63 (SD=10.02) | 0 | 81.3% female | 0 | 7.5 | 1 | France | 0 | 0 |  |
| Gillet, N. | 46 | 2019 | Sample 1 | 1 | 0 | 294 | 37.63 (SD=10.02) | 0 | 81.3% female | 0 | 7.5 | 1 | France | 0 | 0 |  |
| Gillet, N. | 47 | 2020 | Sample 2 | 0 | 0 | 291 | 43.34 (SD=8.76), range= 23-62 | 1 | 82.8% female | 0 | 5.3 | 0 | France | 0 | 0 |  |
| Gillet, N. | 47 | 2020 | Sample 3 | 0 | 0 | 249 | 43.82 (SD=9.47), range= 24-62 | 1 | 65.5% female | 2 | 5.3 | 0 | France | 0 | 0 |  |
| Gillet, N. | 47 | 2020 | Sample 4 | 0 | 0 | 237 | 35.08 (SD=10.55), range=21-64 | 0 | 95.3% female | 0 | 5.3 | 0 | France | 0 | 0 |  |
| Gillet, N. | 47 | 2020 |  | 0 | 0 | 373 | 34.38 (SD=9.17), range=22-63 | 0 | 74.7% female | 2 | 5.3 | 0 | France | 0 | 0 |  |
| Gkorezis, P. | 48 | 2017 |  | 0 | 0 | 124 | 33.93 (SD=9.78) | 0 | 68.3% female | 2 | 7.4 | 3 | Greece | 1 | 1 |  |
| Gostautaite, B. | 49 | 2019 |  | 0 | 0 | 565 | 44.03 (SD=13.70), range 22-80 | 1 | 85% female | 0 | 6.5 | 1 | Lithuania | 1 | 1 |  |
| Greguras, G. J. | 50 | 2009 |  | 0 | 0 | 163 | 35.45 (SD=11.87) | 0 | 58.9% female | 2 | 8 | 0 | Singapore | 1 | 1 |  |
| Gomez‚ÄêBaya, D. | 51 | 2018 |  | 0 | 0 | 2748 | 41.41(average) | 1 | 51.4% female | 2 | 5.5 | 3 | Spain | 1 | 0 |  |
| Gomez‚ÄêBaya, D. | 51 | 2018 |  | 0 | 0 | 1774 | 43.71 (average) | 1 | 81.5% female | 0 | 4.5 | 1 | Bulgaria, Norway | 1 | 2 | 0 |
| Goodboy, A. K. | 52 | 2017 | WE: Vigor | 0 | 0 | 243 | 34.06 (SD = 9.93), range=21-63 | 0 | 75.7% female | 0 | 6.3 | 1 | United States | 0 | 0 |  |
| Goodboy, A. K. | 52 | 2017 | WE: Dedication | 0 | 0 | 243 | 34.06 (SD = 9.93), range=21-64 | 0 | 75.7% female | 0 | 6.3 | 1 | United States | 0 | 0 |  |
| Goodboy, A. K. | 52 | 2017 | WE: Absorption | 0 | 0 | 243 | 34.06 (SD = 9.93), range=21-65 | 0 | 75.7% female | 0 | 6.3 | 1 | United States | 0 | 0 |  |
| Grant, K. | 53 | 2001 | TU: organizational commitment | 0 | 0 | 148 | 34 (median) | 0 | 13% female | 1 | 6.3 | 0 | Australia | 0 | 0 |  |
| Grant, K. | 53 | 2001 | TU: intention to leave | 0 | 0 | 148 | 35 (median) | 0 | 13% female | 1 | 6.3 | 0 | Australia | 0 | 0 |  |
| Graves, L. M. | 54 | 2013 | TU: Affective organizational commitment | 0 | 0 | 283 | 43.3 (SD=12.57 | 1 | 45.9% female | 2 | 5.8 | 2 | United States | 0 | 0 |  |
| Graves, L. M. | 54 | 2013 | TU: Organizational Tenure | 0 | 0 | 283 | 43.3 (SD=12.58 | 1 | 45.9% female | 2 | 5.8 | 2 | United States | 0 | 0 |  |
| Hadi, R. | 55 | 2012 |  | 0 | 0 | 215 | 20-30 40%, 30-40 43.7%, 40-50 8.8%, 50+ 7.4% | 0 | 9.3% female | 1 | 3.2 | 0 | India | 0 | 1 | 2 |
| Haldorai, K. | 56 | 2020 |  | 0 | 0 | 402 | NR | 2 | NR | 2 | 7.9 | 0 | Thailand | 1 | 1 |  |
| Haski-Leventhal, D. | 57 | 2019 |  | 0 | 0 | 911 | 42.83 (SD=9.40) | 1 | NR | 2 | 7.7 | 0 | Australia | 1 | 0 |  |
| Hayati, K. | 58 | 2012 |  | 0 | 0 | 149 | NR | 2 | NR | 2 | 4.7 | 0 | Indonesia | 1 | 1 |  |
| Heyns, M., | 59 | 2017 |  | 0 | 0 | 252 | NR | 2 | 53.3% female | 2 | 4.2 | 0 | South Africa | 1 | 1 |  |
| Klassen, R. M. | 60 | 2012 | REL: Relatedness with Colleagues | 0 | 0 | 409 | 41.57(SD=10.92), range= 20-64 | 1 | 71% F | 2 | 6.3 | 2 | Canada | 0 | 0 | 0 |
| Klassen, R. M. | 60 | 2012 | REL: Relatedness with Students | 0 | 0 | 409 | 41.57(SD=10.92), range= 20-65 | 1 | 71% F | 2 | 6.3 | 2 | Canada | 0 | 0 | 0 |
| Kong, D. T., | 61 | 2015 |  | 0 | 0 | 194 | NR |  | 44.8% female | 2 | 5.8 | 3 | United States | 0 | 0 | 0 |
| Kovjanic, S., | 62 | 2013 |  | 0 | 0 | 190 | 28.36 (SD=8.51) | 0 | 75% female | 0 | 5.8 | 3 | Switzerland | 0 | 0 |  |
| Kovjanic, S., | 63 | 2012 | Study 1 | 0 | 0 | 410 | 36.03 (SD= 11.55) | 0 | 64.9% female | 2 | 6.3 | 3 | Germany, Switzerland | 2 | 0 |  |
| Kovjanic, S., | 64 | 2012 | Study 2 | 1 | 0 | 442 | 33.36 (SD= 11.32) | 0 | 57.5% female | 2 | 6.3 | 3 | Germany, Switzerland | 2 | 2 |  |
| Kuvaas, B. | 65 | 2006 |  | 0 | 0 | 634 | NR | 2 | NR | 2 | 5.3 | 0 | Norway | 1 | 0 | 1 |
| Kuvaas, B. | 66 | 2006 | TU: affective commitment | 0 | 0 | 593 | 44 (average) | 1 | 54.1% female | 2 | 4.7 | 0 | Norway | 1 | 0 |  |
| Kuvaas, B. | 66 | 2006 | TU: Turnover intention | 0 | 0 | 593 | 45 (average) | 1 | 54.1% female | 2 | 4.7 | 0 | Norway | 1 | 0 |  |
| Kuvaas, B. | 67 | 2008 |  | 0 | 0 | 779 | NR | 2 | 70% female | 2 | 5.3 | 3 | Norway | 1 | 0 | 0 |
| Kuvaas, B. | 68 | 2017 | Study 1 | 0 | 0 | 452 | NR | 2 | 57.2% female | 2 | 4.7 | 3 | Norway | 1 | 0 |  |
| Kuvaas, B. | 68 | 2017 | Study 2: TU: Turnover intention | 1 | 0 | 4518 | 48.4 (average) | 2 | 0.572 | 2 | 4.7 | 3 | Norway | 1 | 0 |  |
| Kuvaas, B. | 68 | 2017 | Study 2: TU: Continuance commitment | 1 | 0 | 4518 | 48.4 (average) | 2 | 0.572 | 2 | 4.7 | 3 | Norway | 1 | 0 |  |
| Kuvaas, B. | 68 | 2017 | Study 3 | 0 | 0 | 829 | 44.66 (SD = 9.93 | 1 | 48.1% female | 2 | 4.7 | 3 | Norway | 1 | 0 |  |
| Kuvaas, B. | 69 | 2009 | Study 1 | 0 | 0 | 182 | NR | 2 | NR | 2 | 6.3 | 3 | Norway | 1 | 0 |  |
| Kuvaas, B. | 69 | 2009 | Study 2 | 0 | 0 | 156 | NR | 2 | 49% female | 2 | 6.3 | 0 | Norway | 1 | 0 |  |
| Kuvaas, B. | 69 | 2009 | Study 3 | 0 | 0 | 488 | NR | 2 | 40% female | 2 | 6.3 | 0 | Norway | 1 | 0 |  |
| Kuvaas, B | 70 | 2010 | TU = affective commitment | 0 | 0 | 838 | 45 (SD=10.15) | 1 | 45% female | 2 | 5.3 | 0 | Norway | 1 | 0 |  |
| Kuvaas, B | 70 | 2010 | TU = turnover intention | 0 | 0 | 838 | 46 (SD=10.15) | 1 | 45% female | 2 | 5.3 | 0 | Norway | 1 | 0 |  |
| Lai, L. | 71 | 2011 | TU = affective commitment | 0 | 0 | 881 | 46(SD=11.21) | 1 | 80% female | 0 | 5.8 | 3 | Norway | 1 | 0 |  |
| Lai, L. | 71 | 2011 | TU = normative commitment | 0 | 0 | 881 | 46(SD=11.21) | 1 | 80% female | 0 | 5.8 | 3 | Norway | 1 | 0 |  |
| Lai, L. | 71 | 2011 | TU = continuance commitment | 0 | 0 | 881 | 46(SD=11.21) | 1 | 80% female | 0 | 5.8 | 3 | Norway | 1 | 0 |  |
| Lai, L. | 71 | 2011 | TU = turnover intention | 0 | 0 | 881 | 46(SD=11.21) | 1 | 80% female | 0 | 5.8 | 3 | Norway | 1 | 0 |  |
| Landry, A. T | 72 | 2017 | Study 1 | 0 | 0 | 130 | 25-35 60% 36-45 26.2% 46-55 13.8% | 0 | 61.5% female | 2 | 4.2 | 0 | Greece | 1 | 1 |  |
| Landry, A. T | 72 | 2017 | Study 2 | 1 | 0 | 144 | NR | 2 | 3.5% female | 1 | 4.2 | 0 | Greece | 1 | 1 |  |
| Levesque, M., | 73 | 2004 |  | 0 | 0 | 152 | NR | 2 | 13% female | 1 | 5.3 | 2 | Gabon | 1 | 1 | 0 |
| Lewig, K. A., | 74 | 2003 |  | 0 | 0 | 98 | 32 (SD =10.6),range 18-63 | 0 | 72.4% female | 2 | 5.3 | 0 | Australia | 1 | 0 |  |
| Li, J. | 75 | 2020 |  | 1 | 0 | 245 | 30.84 (SD=6.37) | 0 | 37.1% female | 2 | 5.3 | 0 | China | 0 | 1 |  |
| Li, M. | 76 | 2015 |  | 0 | 0 | 767 | 32.04 (SD = 8.63) | 0 | 64.9% female | 2 | 6.3 | 2 | China | 0 | 1 |  |
| Liu, D. | 77 | 2011 | NSUP: Team leader autonomy support | 1 | 0 | 817 | 27.78 (SD=3.39) | 0 | 48% female | 2 | 6.3 | 3 | United States | 0 | 0 | 0 |
| Liu, D. | 77 | 2011 | NSUP: Peer autonomy support | 1 | 0 | 817 | 27.78 (SD=3.39) | 0 | 48% female | 2 | 6.3 | 3 | United States | 0 | 0 | 0 |
| Liu, H. | 78 | 2018 |  | 0 | 0 | 688 | 18–25 5.5% 26–35 27% 36–45 42.6% 46–55 24.3% 56+ 0.6% | 2 | 19.3% female | 1 | 6.8 | 3 | China | 0 | 1 | 0 |
| Longo, Y., | 79 | 2014 | Study 2- AUT = autonomy satisfaction, CO= competence satisfaction, RE= relatedness satisfaction | 1 | 0 | 356 | 24 (SD=7.51), range= 17-62 | 0 | 74.4% female | 2 | 5.3 | 0 | United Kingdom | 0 | 0 |  |
| Longo, Y., | 79 | 2014 | Study 2- AUT = autonomy frustration, CO= competence frustration, RE= relatedness frustration | 1 | 0 | 356 | 25 (SD=7.51), range= 17-62 | 0 | 74.4% female | 2 | 5.3 | 0 | United Kingdom | 0 | 0 |  |
| Longo, Y., | 79 | 2014 | Study 3- AUT = autonomy satisfaction, CO= competence satisfaction, RE= relatedness satisfaction | 1 | 0 | 243 | 31.59 (SD=9.05), range= 20-67 | 0 | 40% female | 2 | 5.3 | 0 | United Kingdom | 0 | 0 |  |
| Longo, Y., | 79 | 2014 | Study 3- AUT = autonomy frustration, CO= competence frustration, RE= relatedness frustration | 1 | 0 | 243 | 31.59 (SD=9.05), range= 20-68 | 0 | 40% female | 2 | 5.3 | 0 | United States | 0 | 0 |  |
| Lopes, S., | 80 | 2016 | T1 IM, EM, T2 WE: vigor, BU | 1 | 0 | 682 | 31.42 (SD=8.15) | 0 | 57% female | 2 | 5.8 | 3 | Portugal | 1 | 1 |  |
| Lopes, S., | 80 | 2016 | T1 IM, EM, T2 WE: dedication, BU | 1 | 0 | 682 | 31.42 (SD=8.15) | 0 | 61.3% female | 2 | 5.8 | 3 | Portugal | 1 | 1 |  |
| Lopes, S., | 80 | 2016 | T1 IM, EM, T2 WE: absorption, BU | 1 | 0 | 682 | 31.42 (SD=8.15) | 0 | 60.7% female | 2 | 5.8 | 3 | Portugal | 1 | 1 |  |
| Lopes, S., | 80 | 2016 | T2 EM, IM | 1 | 0 | 385 | 30.90 (SD=7.47) | 0 | 60.7% female | 2 | 5.8 | 3 | Portugal | 1 | 1 |  |
| Lopes, S., | 81 | 2013 | WE: Vigor | 0 | 0 | 1325 | 30(SD=7.43), range= 17-62 | 0 | 60.7% female | 2 | 5.8 | 3 | Portugal | 1 | 1 |  |
| Lopes, S., | 81 | 2013 | WE: Dedication | 0 | 0 | 1325 | 30(SD=7.43), range= 17-63 | 0 | 58.6% female | 2 | 5.8 | 3 | Portugal | 1 | 1 |  |
| Lopes, S., | 81 | 2013 | WE: Absorption | 0 | 0 | 1325 | 30(SD=7.43), range= 17-64 | 0 | 58.6% female | 2 | 5.8 | 3 | Portugal | 1 | 1 |  |
| Lopes, S., | 82 | 2018 | WE: Vigor | 0 | 0 | 3938 | 31 (SD= 8.08) | 0 | 53.6% female | 2 | 5.8 | 3 | Portugal | 1 | 1 |  |
| Lopes, S., | 82 | 2018 | WE: Dedication | 0 | 0 | 3938 | 32 (SD= 8.08) | 0 | 53.6% female | 2 | 5.8 | 3 | Portugal | 1 | 1 |  |
| Lopes, S., | 82 | 2018 | WE: Absorption | 0 | 0 | 3938 | 33 (SD= 8.08) | 0 | 53.6% female | 2 | 5.8 | 3 | Portugal | 1 | 1 |  |
| Louka, A. | 83 | 2012 |  | 1 | 1 | 423 | 21-29 27% 30-39 53.2% 40-49 9.2% 50-59 6.1% 60-69 4.5% | 0 | 49.4% female | 2 | 7.4 | 0 | United States | 0 | 0 |  |
| Lu, L. | 84 | 2015 |  | 0 | 0 | 402 | 31.90(SD=7.40) | 0 | 45.2% female | 2 | 4.2 | 3 | China, Taiwan | 2 | 1 |  |
| Lynch Jr., M. F., | 85 | 2005 | NSUP: Perceived autonomy support, JS= intrinsic job satisfaction | 0 | 0 | 186 | 63 (SD=8.69) | 1 | 39.6% female | 2 | 5.3 | 1 | United States | 0 | 0 | 0 |
| Lynch Jr., M. F., | 85 | 2005 | NSUP: Perceived autonomy support, JS= extrinsic job satisfaction | 0 | 0 | 186 | 64 (SD=8.69) | 1 | 39.6% female | 2 | 5.3 | 1 | United States | 0 | 0 | 0 |
| Lynch Jr., M. F., | 85 | 2005 | NSUP: Institutional autonomy support, JS= intrinsic job satisfaction | 0 | 0 | 186 | 65 (SD=8.69) | 1 | 39.6% female | 2 | 5.3 | 1 | United States | 0 | 0 | 1 |
| Lynch Jr., M. F., | 85 | 2005 | NSUP: Institutional autonomy support, JS= extrinsic job satisfaction | 0 | 0 | 186 | 66 (SD=8.69) | 1 | 39.6% female | 2 | 5.3 | 1 | United States | 0 | 0 | 1 |
| Lyu, Y., | 86 | 2019 | TU= affective commitment | 1 | 0 | 266 | 30.41(SD=8.91) | 0 | NR | 2 | 5.8 | 0 | China | 0 | 1 | 1 |
| Lyu, Y., | 86 | 2019 | TU= leave intention | 1 | 0 | 266 | 30.41(SD=8.91) | 0 | NR | 2 | 5.8 | 0 | China | 0 | 1 | 1 |
| Malhotra, N., | 87 | 2020 |  | 0 | 0 | 295 | 58% 31–40 27.5% 21–30 12.5% 41 to 50the remaining were above 50. | 1 | 70.2% female | 2 | 8.9 | 2 | Malaysia | 1 | 1 |  |
| Malinowska, D., | 88 | 2020 |  | 0 | 0 | 1020 | 29.25 (SD= 4.6) | 0 | 69% female | 2 | 6.8 | 0 | Poland | 1 | 0 |  |
| Malinowska, D., | 89 | 2019 |  | 1 | 0 | 907 | 29.42 (SD=4.07) | 0 | 70% female | 2 | 6.8 | 0 | Poland | 1 | 0 | 0 |
| Malinowska, D., | 90 | 2018 |  | 0 | 0 | 318 | 30.8(SD=7.4) | 0 | 74% female | 2 | 4.7 | 0 | Poland | 1 | 0 |  |
| Menges, J | 91 | 2017 |  | 1 | 0 | 97 | 31 (SD = 8.98) | 0 | 100% female | 0 | 6.8 | 3 | Mexico | 1 | 1 |  |
| Merriman, K | 92 | 2017 | JP: IN-role | 0 | 0 | 93 | NR | 2 | NR | 2 | 5.8 | 0 | United States | 0 | 0 | 0 |
| Merriman, K | 92 | 2017 | JP: Extra-role | 0 | 0 | 93 | NR | 2 | NR | 2 | 5.8 | 0 | United States | 0 | 0 | 0 |
| Mishra, M | 93 | 2020 |  | 0 | 0 | 183 | 27.19 (SD=56.78) | 0 | 26.2% female | 2 | 5.8 | 3 | India | 0 | 1 | 0 |
| Mohammad, J | 94 | 2019 |  | 0 | 0 | 282 | Below 25 9.9% 26−35 31.2% 36−45 33.7% 46−55 23.4% 56+ 1.8 % | 2 | 58.2% female | 2 | 6.8 | 0 | Malaysia | 1 | 1 | 1 |
| Moon, T | 95 | 2017 |  | 0 | 0 | 281 | 29.31 (SD = 5.58), range= 21-47 | 0 | 56.9% female | 2 | 7.4 | 0 | South Korea | 1 | 1 |  |
| Moon, T | 96 | 2018 |  | 0 | 0 | 306 | 29 or less 14.7% 30-39 37.9% 40-49 30.4% 50+ 17% | 2 | 26.1% female | 2 | 7.9 | 3 | South Korea | 1 | 1 |  |
| Moran, C | 97 | 2012 |  | 0 | 0 | 226 | mean=31.44(SD=7.83) | 0 | 43.2% female | 2 | 6.3 | 3 | China | 0 | 1 | 0 |
| Mostafa, A | 98 | 2017 | T1 IM & COM, T2 JS | 1 | 0 | 1755 | mean=41 | 1 | 35% female | 2 | 4.7 | 3 | United Kingdom | 0 | 0 |  |
| Mustafa, G | 99 | 2019 |  | 0 | 0 | 100 | mean=31.6 range=23-52 | 0 | 21% female | 1 | 6.3 | 0 | Pakistan | 1 | 1 | 1 |
| Nerstad, C | 100 | 2018 |  | 0 | 0 | 169 | mean=44.18, SD = 10.91 | 1 | 46.7% female | 2 | 6.3 | 0 | Norway | 1 | 0 |  |
| Nie, Y | 101 | 2014 |  | 0 | 0 | 266 | 23-60 | 2 | 70.7% female | 2 | 5.3 | 2 | China | 0 | 1 | 0 |
| Nijhuis, N | 102 | 2012 | WE: vigor | 0 | 0 | 680 | mean=40 (SD=8.92) | 2 | 31.8% female | 2 | 5.8 | 0 | Netherlands | 1 | 0 |  |
| Nijhuis, N | 102 | 2012 | WE: dedication | 0 | 0 | 680 | mean=40 (SD=8.92) | 2 | 31.8% female | 2 | 5.8 | 0 | Netherlands | 1 | 0 |  |
| Nijhuis, N | 102 | 2012 | WE: absorption | 0 | 0 | 680 | mean=40 (SD=8.92) | 2 | 31.8% female | 2 | 5.8 | 0 | Netherlands | 1 | 0 |  |
| Olafsen, A | 103 | 2016 | T1 NS AU, T2 WB, TU | 1 | 0 | 185 | 2=<30,19=30-39,61=40-49,78=50-59, 24=>60 | 2 | 76.2% female | 0 | 6.3 | 1 | Norway | 1 | 0 | 0 |
| Olafsen, A | 103 | 2016 | T1 NS AU, T3 WB, TU | 1 | 0 | 152 | 2=<30,13=30-39,49=40-49,67=50-59,20=>60 | 2 | 76.3% female | 0 | 6.3 | 1 | Norway | 1 | 0 | 0 |
| Olafsen, A | 103 | 2016 | T1 NS AU, T4 WB, TU | 1 | 0 | 115 | 2, 7, 39, 52 | 2 | 76.5% female | 0 | 6.3 | 1 | Norway | 1 | 0 | 0 |
| Olafsen, A | 103 | 2016 | T2 NS AU, T3 WB, TU | 1 | 0 | 152 | 2=<30,13=30-39,49=40-49,67=50-59,20=>60 | 2 | 76.3% female | 0 | 6.3 | 1 | Norway | 1 | 0 | 0 |
| Olafsen, A | 103 | 2016 | T2 NS AU, T4 WB, TU | 1 | 0 | 115 | 2, 7, 39, 52 | 2 | 76.5% female | 0 | 6.3 | 1 | Norway | 1 | 0 | 0 |
| Olafsen, A | 103 | 2016 | T3 NS AU, T4 WB, TU | 1 | 0 | 115 | 2, 7, 39, 52 | 2 | 76.5% female | 0 | 6.3 | 1 | Norway | 1 | 0 | 0 |
| Olafsen, A | 104 | 2016 | T1 AU, T2 BU | 1 | 0 | 185 | 2=<30,19=30-39,61=40-49,78=50-59, 24=>60 | 2 | 76.2% female | 0 | 6.8 | 1 | Norway | 1 | 0 |  |
| Olafsen, A | 104 | 2016 | T1 AU, T3 BU, TU | 1 | 0 | 152 | 2=<30,13=30-39,49=40-49,67=50-59,20=>60 | 2 | 76.3% female | 0 | 6.8 | 1 | Norway | 1 | 0 |  |
| Olafsen, A | 104 | 2016 | T1 AU, T4 BU, TU | 1 | 0 | 115 | 2, 7, 39, 52 | 2 | 76.5% female | 0 | 6.8 | 1 | Norway | 1 | 0 |  |
| Olafsen, A | 104 | 2016 | T2 AU, T3 BU, TU | 1 | 0 | 152 | 2=<30,13=30-39,49=40-49,67=50-59,20=>60 | 2 | 76.3% female | 0 | 6.8 | 1 | Norway | 1 | 0 |  |
| Olafsen, A | 104 | 2016 | T2 AU, T4 BU, TU | 1 | 0 | 115 | 2, 7, 39, 52 | 2 | 76.5% female | 0 | 6.8 | 1 | Norway | 1 | 0 |  |
| Olafsen, A | 104 | 2016 | T3 AU, T4 BU, TU | 1 | 0 | 115 | 2, 7, 39, 52 | 2 | 76.5% female | 0 | 6.8 | 1 | Norway | 1 | 0 |  |
| Olafsen, A | 105 | 2017 | IM= IM, JP= work quality | 0 | 0 | 405 | 47.8%<30 | 2 | 65.7% female | 2 | 5.8 | 3 | Norway | 1 | 0 |  |
| Olafsen, A | 105 | 2017 | IM= Global autonomous motivation, JP= work quality | 0 | 0 | 405 | 47.8%<31 | 2 | 65.7% female | 2 | 5.8 | 3 | Norway | 1 | 0 |  |
| Olafsen, A | 105 | 2017 | IM= IM, JP= global work performance | 0 | 0 | 405 | 47.8%<32 | 2 | 65.7% female | 2 | 5.8 | 3 | Norway | 1 | 0 |  |
| Olafsen, A | 105 | 2017 | IM= Global autonomous motivation, JP= global work performance | 0 | 0 | 405 | 47.8%<33 | 2 | 65.7% female | 2 | 5.8 | 3 | Norway | 1 | 0 |  |
| Omansky, R | 107 | 2016 |  | 0 | 0 | 213 | range=18-36, mean=20.9 (SD=1.9) | 0 | 49% female | 2 | 5.3 | 3 | United States | 0 | 0 |  |
| Onyishi, I | 108 | 2019 |  | 0 | 0 | 233 | range=17-56, mean=39.06 (SD=7.93) | 0 | 69.5% female | 2 | 7.9 | 1 | Nigeria | 1 | 1 |  |
| Park, R | 110 | 2016 |  | 0 | 0 | 601 | NR | 2 | NR | 2 | 5.8 | 0 | South Korea | 1 | 1 |  |
| Park, S | 110 | 2015 |  | 0 | 0 | 790 | mean=49.02 (SD=.49) | 1 | NR | 2 | 6.3 | 0 | United States | 0 | 0 |  |
| Parker, S | 111 | 2010 | WE: Vigor | 0 | 0 | 123 | range=17-60, M=33.97, sd=11.34 | 0 | 80% female | 0 | 4.7 | 1 | Australia | 1 | 0 |  |
| Parker, S | 111 | 2010 | WE: Dedication | 0 | 0 | 123 | range=17-60, M=33.97, sd=11.35 | 0 | 80% female | 0 | 4.7 | 1 | Australia | 1 | 0 |  |
| Parker, S | 111 | 2010 | WE: Absorption | 0 | 0 | 123 | range=17-60, M=33.97, sd=11.36 | 0 | 80% female | 0 | 4.7 | 1 | Australia | 1 | 0 |  |
| Pearce, J | 112 | 1983 |  | 1 | 0 | 106 | NR | 2 | NR | 2 | 5.8 | 3 | United States | 0 | 0 |  |
| Potipiroon, W | 113 | 2017 |  | 0 | 0 | 196 | mean= 44 (SD=8.67) | 1 | 60% female | 0 | 6.3 | 0 | Thailand | 1 | 1 |  |
| Prokesova, L | 114 | 2019 |  | 0 | 0 | 783 | mean= 37 (SD=11.42) | 0 | 65% female | 2 | 6.1 | 0 | Czech Republic | 1 | 0 |  |
| Putra, E | 115 | 2015 | WE: Vigor | 0 | 0 | 143 | mean= 24.39 | 0 | 61.5% female | 2 | 6.8 | 3 | United States | 0 | 0 |  |
| Putra, E | 115 | 2015 | WE: Dedication | 0 | 0 | 143 | mean= 24.40 | 0 | 61.5% female | 2 | 6.8 | 3 | United States | 0 | 0 |  |
| Putra, E | 115 | 2015 | WE: Absorption | 0 | 0 | 143 | mean= 24.41 | 0 | 61.5% female | 2 | 6.8 | 3 | United States | 0 | 0 |  |
| Rahmadani, V | 116 | 2019 | POP: Indonesia, WE: vigor | 0 | 0 | 607 | mean=44.6 (SD=7.7) | 1 | 0% female | 1 | 7.4 | 3 | Indonesia | 1 | 1 |  |
| Rahmadani, V | 116 | 2019 | POP: Indonesia, WE: dedication | 0 | 0 | 607 | mean=44.6 (SD=7.7) | 1 | 0% female | 1 | 7.4 | 3 | Indonesia | 1 | 1 |  |
| Rahmadani, V | 116 | 2019 | POP: Indonesia, WE: Absorption | 0 | 0 | 607 | mean=44.6 (SD=7.7) | 1 | 0% female | 1 | 7.4 | 3 | Indonesia | 1 | 1 |  |
| Rahmadani, V | 116 | 2019 | POP: Russia, WE: vigor | 0 | 0 | 384 | mean=40.4 (SD=11.7) | 2 | 75% female | 0 | 7.4 | 3 | Russia | 0 | 1 |  |
| Rahmadani, V | 116 | 2019 | POP: Russia, WE: dedication | 0 | 0 | 384 | mean=40.4 (SD=11.7) | 2 | 75% female | 0 | 7.4 | 3 | Russia | 0 | 1 |  |
| Rahmadani, V | 116 | 2019 | POP: Russia, WE: Absorption | 0 | 0 | 384 | mean=40.4 (SD=11.7) | 2 | 75% female | 0 | 7.4 | 3 | Russia | 0 | 1 |  |
| Rahmadani, V | 116 | 2020 |  | 1 | 0 | 607 | <30(4.6%), 31-39 (20.1%), 40-49 (43.9%), >50 (31.3%) | 2 | 0% female | 1 | 7.8 | 3 | Indonesia | 1 | 1 |  |
| Ramalu, S | 117 | 2019 |  | 1 | 0 | 152 | 40-49(44.1%), 50-59 (37.5%), | 1 | 22.4% female | 1 | 6.8 | 3 | Malaysia | 1 | 1 |  |
| Randelovic, K | 118 | 2017 |  | 0 | 0 | 147 | mean=32.4 (SD=6.51) | 0 | 19.8% female | 1 | 6.3 | 2 | Serbia | 1 | 1 |  |
| Raven, H | 119 | 2019 |  | 0 | 0 | 80 | m=44.46 | 2 | 61% female | 2 | 5.2 | 2 | Germany | 0 | 0 |  |
| Rawolle, M | 120 | 2016 |  | 0 | 0 | 49 | M=36.14, SD=7.53 | 0 | 38.78% female | 2 | 7.89 | 0 | Germany | 0 | 0 |  |
| Reinardy, S | 121 | 2014 |  | 0 | 0 | 887 | M=41, | 2 | NR | 2 | 8.94 | 0 | United States | 0 | 0 |  |
| Reizer, A | 122 | 2019 |  | 1 | 0 | 116 | mean=31.76, SD=11.02 | 0 | 59% female | 2 | 5 | 3 | Israel | 1 | 0 | 0 |
| Ren, T | 123 | 2017 | TU= affective commitment | 0 | 0 | 222 | 20-30 (68%) | 0 | 75% female | 0 | 5.8 | 0 | China | 0 | 1 | 1 |
| Ren, T | 123 | 2017 | TU= intent to quit | 0 | 0 | 222 | 20-30 (68%) | 0 | 75% female | 0 | 5.8 | 0 | China | 0 | 1 | 1 |
| Renard, M | 124 | 2017 |  | 0 | 0 | 587 | 18-27 12% | 1 | 72% female | 0 | 6.3 | 3 | Australia, Belgium | 2 | 2 |  |
| Robijn, W | 125 | 2020 |  | 0 | 0 | 133 | mean=42.3 (SD=10.2) | 1 | 21.8% female | 1 | 5.8 | 0 | Belgium | 1 | 0 |  |
| Roche, M | 126 | 2019 |  | 1 | 0 | 386 | mean=37.4 (SD=13) | 0 | 42% female | 2 | 5.8 | 0 | New Zealand | 1 | 0 |  |
| Rubino, C | 127 | 2009 | Burnout: Emotional exhaustion | 0 | 0 | 284 | NR | 2 | NR | 2 | 5.8 | 3 | United States | 0 | 0 |  |
| Rubino, C | 127 | 2009 | Burnout: Cynisism | 0 | 0 | 284 | NR | 2 | NR | 2 | 5.8 | 3 | United States | 0 | 0 |  |
| Rubino, C | 127 | 2009 | Burnout: Inefficacy | 0 | 0 | 284 | NR | 2 | NR | 2 | 5.8 | 3 | United States | 0 | 0 |  |
| Sagnak, M | 128 | 2016 |  | 0 | 0 | 850 | 31-40 (46.7%) | 2 | 48,2% female | 2 | 5.26 | 2 | Turkey | 1 | 1 | 0 |
| Sandrin, √â | 129 | 2019 |  | 0 | 0 | 654 | mean=41, SD=8.27 | 2 | 8.6% female | 1 | 6 | 3 | France | 0 | 0 |  |
| Sawang, S | 130 | 2020 | Wave 1 | 1 | 0 | 901 | mean=47 | 1 | 39% female | 2 | 6.7 | 3 | Australia | 1 | 0 |  |
| Sawang, S | 130 | 2020 | Wave 2 | 1 | 0 | 858 | mean=48 | 1 | 39% female | 2 | 6.7 | 3 | Australia | 1 | 0 |  |
| Sawang, S | 130 | 2020 | Wave 3 | 1 | 0 | 805 | mean=49 | 1 | 39% female | 2 | 6.7 | 3 | Australia | 1 | 0 |  |
| Sawang, S | 130 | 2020 | Wave 4 | 1 | 0 | 769 | mean=50 | 1 | 39% female | 2 | 6.7 | 3 | Australia | 1 | 0 |  |
| Sawang, S | 130 | 2020 | Wave 5 | 1 | 0 | 860 | mean=51 | 1 | 39% female | 2 | 6.7 | 3 | Australia | 1 | 0 |  |
| Sawang, S | 130 | 2020 | Wave 6 | 1 | 0 | 840 | mean=52 | 1 | 39% female | 2 | 6.7 | 3 | Australia | 1 | 0 |  |
| Sawang, S | 130 | 2020 | Wave 7 | 1 | 0 | 1112 | mean=53 | 1 | 39% female | 2 | 6.7 | 3 | Australia | 1 | 0 |  |
| Sawang, S | 130 | 2020 | Wave 8 | 1 | 0 | 1029 | mean=54 | 1 | 39% female | 2 | 6.7 | 3 | Australia | 1 | 0 |  |
| Sawang, S | 130 | 2020 | Wave 9 | 1 | 0 | 1018 | mean=55 | 1 | 39% female | 2 | 6.7 | 3 | Australia | 1 | 0 |  |
| Schopman, L | 131 | 2015 |  | 0 | 0 | 171 | mean=40, SD=11.89 | 2 | 80.7% female | 0 | 6.3 | 1 | Netherlands | 1 | 1 |  |
| Schreurs, B | 132 | 2014 |  | 0 | 0 | 307 | mean=38.94 (SD=6.03) | 0 | 33.3% female | 2 | 6.8 | 3 | Belgium, Netherlands | 1 | 2 |  |
| Schultz, P | 133 | 2014 | AUT = Need satisfaction | 0 | 0 | 259 | mean= 35.47 (SD= 9.81) | 0 | 56.8% female | 2 | 6.8 | 0 | United States | 0 | 9 |  |
| Schultz, P | 133 | 2014 | AUT = Need frustration | 0 | 0 | 259 | mean= 35.47 (SD= 9.81) | 0 | 56.8% female | 2 | 6.8 | 0 | United States | 0 | 0 |  |
| Shih, C | 134 | 2019 |  | 1 | 0 | 790 | mean=22 (SD=4.85) | 0 | 35.3% female | 2 | 6.8 | 3 | Taiwan | 1 | 1 |  |
| Shkoler, O | 135 | 2020 | Israeli Workers | 0 | 0 | 77 | mean=35.26 (SD=9.95) | 0 | 36.8% female | 2 | 6.8 | 3 | Israel, Japan | 1 | 2 |  |
| Shkoler, O | 135 | 2020 | Israeli non student employees | 0 | 0 | 165 | mean=35.26 (SD=9.95) | 0 | 36.8% female | 2 | 6.8 | 3 | Israel, Japan | 1 | 2 |  |
| Shkoler, O | 135 | 2020 | Japanese Workers | 0 | 0 | 97 | mean=45.57 (SD=8.93) | 2 | 49.7% female | 2 | 6.8 | 3 | Israel, Japan | 0 | 2 |  |
| Shkoler, O | 135 | 2020 | Japanese non student employees | 0 | 0 | 74 | mean=45.57 (SD=8.93) | 2 | 49.7% female | 2 | 6.8 | 3 | Israel, Japan | 0 | 2 |  |
| Shuck, B | 136 | 2018 |  | 0 | 0 | 365 | NR | 2 | 35% female | 2 | 4.7 | 3 | United States | 0 | 0 |  |
| Skaalvik, E | 137 | 2014 |  | 0 | 0 | 2569 | mean=45 (SD=11.5) | 1 | 72% female | 2 | 5.8 | 2 | Norway | 1 | 0 |  |
| Skiba, T | 138 | 2018 |  | 0 | 1 | 208 | mean=35 (SD=11.30) | 0 | 52% female | 2 | 6.8 | 3 | United States | 0 | 0 |  |
| Skipworth, C | 139 | 2016 |  | 0 | 0 | 100 | NR | 2 | 0% female | 1 | 8 | 3 | United States | 0 | 0 |  |
| Slemp, G | 140 | 2013 | AUT = autonomy, WB= subjective wb | 0 | 0 | 334 | mean=41.94 (SD=11.38) | 1 | 66.8% female | 2 | 5.5 | 2 | Australia | 1 | 0 |  |
| Slemp, G | 140 | 2013 | AUT = autonomy, WB= psychological WB | 0 | 0 | 334 | mean=41.94 (SD=11.38) | 1 | 66.8% female | 2 | 5.5 | 2 | Australia | 1 | 0 |  |
| Slemp, G | 140 | 2013 | AUT = total need satisfaction, WB= subjective wb | 0 | 0 | 334 | mean=41.94 (SD=11.38) | 1 | 66.8% female | 2 | 5.5 | 2 | Australia | 1 | 0 |  |
| Slemp, G | 140 | 2013 | AUT = total need satisfaction, WB= psychological WB | 0 | 0 | 334 | mean=41.94 (SD=11.38) | 1 | 66.8% female | 2 | 5.5 | 2 | Australia | 1 | 0 |  |
| Slemp, G | 141 | 2020 | Sample: Australian | 0 | 0 | 298 | mean=34.10 (SD=11.70) | 0 | 66% female | 2 | 5.8 | 0 | Australia | 1 | 0 |  |
| Slemp, G | 141 | 2020 | Sample: Chinese | 0 | 0 | 228 | 28 | 0 | 33% female | 2 | 5.8 | 3 | Australia | 0 | 0 |  |
| Smith, J | 142 | 2017 | Year 1 | 1 | 0 | 260 | NR | 2 | 42.7% female | 2 | 6.3 | 2 | United States | 0 | 0 |  |
| Smith, J | 142 | 2017 | Year 2 | 1 | 0 | 239 | NR | 2 | 45.2% female | 2 | 6.3 | 2 | United State | 0 | 0 |  |
| Smith, J | 142 | 2017 | Year 3 | 1 | 0 | 252 | NR | 2 | 46% female | 2 | 6.3 | 2 | United States | 0 | 0 |  |
| Steinbauer, R | 143 | 2018 |  | 0 | 0 | 101 | NR | 2 | 33% female | 2 | 5.8 | 0 | United States | 0 | 0 |  |
| Stokowski, S | 144 | 2018 |  | 0 | 0 | 166 | mean=46.58 (SD=11.51) | 1 | 32.5% female | 2 | 7.4 | 2 | United States | 0 | 0 |  |
| Strauss, K | 145 | 2016 |  | 1 | 0 | 254 | mean=40.27 (SD=10.36) | 2 | 32.5% female | 2 | 5.8 | 0 | Canada, United States | 0 | 0 |  |
| Sun, P | 146 | 2016 |  | 0 | 0 | 277 | 36-45 (45.2%) | 2 | 39.4% female | 2 | 6.3 | 0 | Taiwan | 1 | 1 | 0 |
| ten Brummelhuis, L | 147 | 2011 | BU: Exhaustion | 1 | 0 | 352 | mean=39.68 | 0 | 49% female | 2 | 7.4 | 0 | Netherlands | 1 | 0 |  |
| ten Brummelhuis, L | 147 | 2011 | BU: Cynicism | 1 | 0 | 352 | mean=39.69 | 0 | 49% female | 2 | 7.4 | 0 | Netherlands | 1 | 0 |  |
| Thatcher, J | 148 | 2006 |  | 0 | 0 | 228 | mean= 37, sd=8.86 | 0 | 33.3% female | 2 | 5.8 | 0 | United States | 0 | 0 |  |
| Thibault-Landry, A | 149 | 2018 | AUT: Job autonomy, REL: connectedness colleagues | 0 | 0 | 1456 | 37% <35, 51% 35-50, 12%>51 | 2 | 79% female | 0 | 5.8 | 0 | United States | 0 | 0 |  |
| Thibault-Landry, A | 149 | 2018 | AUT: Job autonomy, REL: connectedness leadership | 0 | 0 | 1456 | 37% <35, 51% 35-50, 12%>52 | 2 | 79% female | 0 | 5.8 | 0 | United States | 0 | 0 |  |
| Thibault-Landry, A | 149 | 2018 | AUT: psychological need satisfaction, REL: connectedness colleagues | 0 | 0 | 1456 | 37% <35, 51% 35-50, 12%>53 | 2 | 79% female | 0 | 5.8 | 0 | United States | 0 | 0 |  |
| Thibault-Landry, A | 149 | 2018 | AUT: psychological need satisfaction, REL: connectedness leadership | 0 | 0 | 1456 | 37% <35, 51% 35-50, 12%>54 | 2 | 79% female | 0 | 5.8 | 0 | United States | 0 | 0 |  |
| Trepanier, S | 150 | 2013 |  | 0 | 0 | 1179 | mean=42.7 (SD=10.8) | 1 | 91% female | 0 | 5.8 | 1 | Canada | 0 | 0 |  |
| Trepanier, S | 151 | 2012 | Study 1 | 0 | 0 | 356 | mean=41.8 (SD=10.4) | 1 | 74.9% female | 2 | 4.2 | 2 | Canada | 0 | 0 |  |
| Trepanier, S | 151 | 2012 | Study 2 | 0 | 0 | 277 | mean=44.32 (SD=19.5) | 1 | 62% female | 2 | 5.8 | 2 | Canada | 0 | 0 |  |
| Trepanier, S | 152 | 2014 | AUT T1, BU, WE, TU T2 | 1 | 0 | 508 | 20 | 0 | 90.5% female | 0 | 5.8 | 1 | Canada | 0 | 0 | 0 |
| Trepanier, S | 153 | 2015 | AUT: Need frustration | 0 | 0 | 699 | mean=43.97 (SD=10.51) | 1 | 90.4% female | 0 | 5.8 | 1 | Canada | 0 | 0 |  |
| Trepanier, S | 153 | 2015 | AUT: Need satisfaction | 0 | 0 | 699 | mean=43.97 (SD=10.51) | 1 | 90.4% female | 0 | 5.2 | 1 | Canada | 0 | 0 |  |
| Trepanier, S | 154 | 2020 | T! IM, EM, T2 BU | 1 | 0 | 399 | mean=42.74 (SD=11.40) | 1 | 88.8% female | 0 | 5.2 | 1 | Canada | 0 | 0 |  |
| Tuin, L | 156 | 2020 | AU, CO, REL satisfaction | 0 | 0 | 304 | < 34 (54%), 35-49 (20%), >49 (28%) | 2 | 30% female (90) | 2 | 6.3 | 0 | Netherlands | 1 | 0 |  |
| Tuin, L | 156 | 2020 | AU, CO, REL frustration | 0 | 0 | 304 | < 34 (54%), 35-49 (20%), >49 (28%) | 2 | 30% female (90) | 2 | 6.3 | 0 | Netherlands | 1 | 0 |  |
| Tummers, G | 157 | 2006 |  | 0 | 0 | 184 | mean=35.8 (SD=6.4) | 0 | 64% female | 2 | 6.3 | 1 | Netherlands | 1 | 0 |  |
| Tyagi, P | 158 | 1985 |  | 0 | 0 | 94 | NR | 2 | NR | 2 | 6.3 | 0 | United States | 0 | 0 |  |
| Unanue, W | 159 | 2017 | AUT T1, JS T2 | 1 | 0 | 636 | mean=39.76 (SD=8.61) | 0 | 52.8% female | 2 | 8.4 | 3 | Chile | 1 | 1 |  |
| Unanue, W | 160 | 2017 | Sample: Chile, AUT: Need satisfaction | 0 | 0 | 742 | mean=31.80 (SD=7.57) | 0 | 73% female | 2 | 7.4 | 3 | Chile, Paraguay | 1 | 1 |  |
| Unanue, W | 160 | 2017 | Sample: Chile, AUT: Need frustration | 0 | 0 | 742 | mean=31.80 (SD=7.57) | 0 | 73% female | 2 | 7.4 | 3 | Chile, Paraguay | 1 | 1 |  |
| Unanue, W | 160 | 2017 | Sample: Paraguay, AUT: Need satisfaction | 0 | 0 | 518 | m=28 | 0 | 48% female | 2 | 7.4 | 3 | Chile, Paraguay | 1 | 1 |  |
| Unanue, W | 160 | 2017 | Sample: Paraguay, AUT: Need frustration | 0 | 0 | 518 | m=29 | 0 | 48% female | 2 | 7.4 | 3 | Chile, Paraguay | 1 | 1 |  |
| van Beek, I | 161 | 2011 | WE: Vigor, BU = exhaustion | 0 | 0 | 544 | mean=29.23 (SD=7.48) | 0 | 98.9% female | 0 | 4.7 | 1 | China | 0 | 1 | 0 |
| van Beek, I | 161 | 2011 | WE: Dedication, BU = exhaustion | 0 | 0 | 545 | mean=29.23 (SD=7.48) | 0 | 98.9% female | 0 | 4.7 | 1 | China | 0 | 1 | 0 |
| van Beek, I | 161 | 2011 | WE: Absoroption, BU = exhaustion | 0 | 0 | 546 | mean=29.23 (SD=7.48) | 0 | 98.9% female | 0 | 4.7 | 1 | China | 0 | 1 | 0 |
| van Beek, I | 161 | 2011 | WE: Vigor, BU = cynicism | 0 | 0 | 547 | mean=29.23 (SD=7.48) | 0 | 98.9% female | 0 | 4.7 | 1 | China | 0 | 1 | 0 |
| van Beek, I | 161 | 2011 | WE: Dedication, BU = cynicism | 0 | 0 | 548 | mean=29.23 (SD=7.48) | 0 | 98.9% female | 0 | 4.7 | 1 | China | 0 | 1 |  |
| van Beek, I | 161 | 2011 | WE: Absoroption, BU = cynicism | 0 | 0 | 549 | mean=29.23 (SD=7.48) | 0 | 98.9% female | 0 | 4.7 | 1 | China | 0 | 1 |  |
| van den Berg, P | 162 | 2011 |  | 0 | 0 | 73 | mean=59 (SD=4.1) | 1 | 48% female | 2 | 6.8 | 3 | Netherlands | 1 | 0 |  |
| Van den Broeck, A | 163 | 2011 |  | 0 | 0 | 370 | mean=37.95 (SD=11.19) | 0 | 46% female | 2 | 6.3 | 0 | Belgium | 1 | 0 |  |
| van der Kolk, B | 164 | 2018 |  | 0 | 0 | 142 | 20-30 (4%), 31-40 (12%), 41-50 (30%), >50 (54%) | 1 | 68% female | 2 | 7.4 | 0 | Netherlands | 1 | 0 |  |
| van Dorssen-Boog, P | 165 | 2020 |  | 0 | 0 | 337 | mean= 41.5 (SD=12.8) | 1 | 69% female | 2 | 6.3 | 1 | Netherlands | 1 | 0 |  |
| van Hooff, M | 166 | 2016 | Study 1; AUT = task autonomy | 0 | 0 | 115 | mean= 35.3 (SD=10.2) | 0 | 53% female | 2 | 7.4 | 0 | Netherlands | 1 | 0 |  |
| van Hooff, M | 166 | 2016 | Study 1; AUT = need satisfaction | 0 | 0 | 115 | mean= 35.3 (SD=10.2) | 0 | 53% female | 2 | 7.4 | 0 | Netherlands | 1 | 0 |  |
| van Hooff, M | 166 | 2016 | Study 2 | 0 | 0 | 76 | mean=36.7 (SD=11.0) | 0 | 44% female | 2 | 7.4 | 0 | Netherlands | 1 | 0 |  |
| van Wingerden, J | 167 | 2018 | WE: vigor | 0 | 0 | 1188 | mean=21.8 (SD=1.29) | 0 | 57% female | 2 | 5.8 | 3 | Netherlands | 1 | 0 | 0 |
| van Wingerden, J | 167 | 2018 | WE: dedication | 0 | 0 | 1188 | mean= 42 (SD=10.41) | 1 | 52% female | 2 | 5.8 | 3 | Netherlands | 1 | 0 | 0 |
| van Wingerden, J | 167 | 2018 | WE: absorption | 0 | 0 | 1188 | mean= 42 (SD=10.41) | 1 | 52% female | 2 | 5.8 | 3 | Netherlands | 1 | 0 | 0 |
| Vander Elst, T | 168 | 2012 |  | 0 | 0 | 3185 | mean=39.6 (SD=10.6) | 0 | 62% female | 2 | 6.8 | 3 | Belgium | 1 | 0 |  |
| Vansteenkiste, M | 169 | 2007 |  | 0 | 0 | 855 | mean=40 (SD=11.16) | 2 | 47% female | 2 | 6.3 | 3 | Belgium | 1 | 0 |  |
| Victor, J | 170 | 2017 |  | 0 | 0 | 251 | range= 17-72 | 2 | 574% female | 2 | 6.3 | 3 | South AFrica | 1 | 1 |  |
| Virga, D | 171 | 2013 | WE: vigor | 0 | 0 | 221 | mean=35 | 0 | 82,1% female | 0 | 6.3 | 3 | Romania | 1 | 1 |  |
| Virga, D | 171 | 2013 | WE: dedication | 0 | 0 | 221 | mean=36 | 0 | 82,1% female | 0 | 6.3 | 3 | Romania | 1 | 1 |  |
| Virga, D | 171 | 2013 | WE absorption | 0 | 0 | 221 | mean=37 | 0 | 82,1% female | 0 | 6.3 | 3 | Romania | 1 | 1 |  |
| Vujcic, M | 172 | 2016 |  | 0 | 0 | 153 | mean=43 (SD=10.16) | 1 | 80.3% female | 0 | 7.9 | 2 | Croatia | 1 | 0 |  |
| Walczak, R | 173 | 2014 |  | 0 | 0 | 1021 | m=30.3 (SD= 9.6) | 0 | 55% female | 2 | 5.8 | 3 | Poland | 1 | 0 |  |
| Wallace, J | 174 | 1991 |  | 0 | 0 | 95 | mean=32 | 0 | 80% female | 0 | 6.8 | 0 | NR | 0 | 2 |  |
| Wang, E | 175 | 2019 |  | 0 | 0 | 1212 | mean=49.16 (SD=10.41) | 1 | 84.40% female | 0 | 6.3 | 0 | NR | 0 | 2 |  |
| Wang, N | 176 | 2020 |  | 0 | 0 | 200 | mean=37.82 (SD=12.09) | 0 | 50% female | 2 | 5.8 | 2 | Germany | 0 | 0 |  |
| Wang, Z | 177 | 2018 |  | 1 | 0 | 438 | mean=25.04 (SD=2.73) | 0 | 57% female | 2 | 7.4 | 3 | China | 0 | 1 |  |
| Williams, G | 178 | 2014 |  | 0 | 0 | 287 | NR | 2 | 48.1% female | 2 | 6.3 | 0 | Nordic Countries | 1 | 0 | 0 |
| Wininger, S | 179 | 2013 | REL: student | 0 | 0 | 126 | NR | 2 | 54% female | 2 | 4.2 | 2 | United States | 0 | 0 |  |
| Wininger, S | 179 | 2013 | REL: colleague | 0 | 0 | 126 | NR | 2 | 54% female | 2 | 4.2 | 2 | United States | 0 | 0 |  |
| Winter-Collins, A | 180 | 2016 | JS: Total satisfaction | 0 | 0 | 107 | mean=31.2 (SD=9.2)mean=31.2 (SD=9.2) mean=29.8 (SD=8.5) mean=29.5 (SD=10.6) mean= 32.2 (SD=8.4) mean=38.9 (SD=15.8) | 0 | NR | 2 | 4.7 | 1 | United States | 0 | 0 |  |
| Winter-Collins, A | 180 | 2016 | JS: extrinsic rewards | 0 | 0 | 107 | mean=31.2 (SD=9.2)mean=31.2 (SD=9.2) mean=29.8 (SD=8.5) mean=29.5 (SD=10.6) mean= 32.2 (SD=8.4) mean=38.9 (SD=15.8) | 0 | NR | 2 | 4.7 | 1 | United States | 0 | 0 |  |
| Winter-Collins, A | 180 | 2016 | JS: Schedule | 0 | 0 | 107 | mean=31.2 (SD=9.2)mean=31.2 (SD=9.2) mean=29.8 (SD=8.5) mean=29.5 (SD=10.6) mean= 32.2 (SD=8.4) mean=38.9 (SD=15.8) | 0 | NR | 2 | 4.7 | 1 | United States | 0 | 0 |  |
| Winter-Collins, A | 180 | 2016 | JS: balance | 0 | 0 | 107 | mean=31.2 (SD=9.2)mean=31.2 (SD=9.2) mean=29.8 (SD=8.5) mean=29.5 (SD=10.6) mean= 32.2 (SD=8.4) mean=38.9 (SD=15.8) | 0 | NR | 2 | 4.7 | 1 | United States | 0 | 0 |  |
| Winter-Collins, A | 180 | 2016 | JS: coworkers interaction | 0 | 0 | 107 | mean=31.2 (SD=9.2)mean=31.2 (SD=9.2) mean=29.8 (SD=8.5) mean=29.5 (SD=10.6) mean= 32.2 (SD=8.4) mean=38.9 (SD=15.8) | 0 | NR | 2 | 4.7 | 1 | United States | 0 | 0 |  |
| Winter-Collins, A | 180 | 2016 | JS: interaction opportunities | 0 | 0 | 107 | mean=31.2 (SD=9.2)mean=31.2 (SD=9.2) mean=29.8 (SD=8.5) mean=29.5 (SD=10.6) mean= 32.2 (SD=8.4) mean=38.9 (SD=15.8) | 0 | NR | 2 | 4.7 | 1 | United States | 0 | 0 |  |
| Winter-Collins, A | 180 | 2016 | JS: professional opportunities | 0 | 0 | 107 | mean=31.2 (SD=9.2)mean=31.2 (SD=9.2) mean=29.8 (SD=8.5) mean=29.5 (SD=10.6) mean= 32.2 (SD=8.4) mean=38.9 (SD=15.8) | 0 | NR | 2 | 4.7 | 1 | United States | 0 | 0 |  |
| Winter-Collins, A | 180 | 2016 | JS: Praise | 0 | 0 | 107 | mean=31.2 (SD=9.2)mean=31.2 (SD=9.2) mean=29.8 (SD=8.5) mean=29.5 (SD=10.6) mean= 32.2 (SD=8.4) mean=38.9 (SD=15.8) | 0 | NR | 2 | 4.7 | 1 | United States | 0 | 0 |  |
| Winter-Collins, A | 180 | 2016 | JS: control | 0 | 0 | 107 | mean=31.2 (SD=9.2)mean=31.2 (SD=9.2) mean=29.8 (SD=8.5) mean=29.5 (SD=10.6) mean= 32.2 (SD=8.4) mean=38.9 (SD=15.8) | 0 | NR | 2 | 4.7 | 1 | United States | 0 | 0 |  |
| Xie, T | 181 | 2016 | WE: Overall | 0 | 0 | 204 | NR | 2 | NR | 2 | 6.8 | 0 | China | 0 | 1 |  |
| Xie, T | 181 | 2016 | WE: Cognitive | 0 | 0 | 204 | NR | 2 | NR | 2 | 6.8 | 0 | China | 0 | 1 |  |
| Xie, T | 181 | 2016 | WE: emotional | 0 | 0 | 204 | NR | 2 | NR | 2 | 6.8 | 0 | China | 0 | 1 |  |
| Xie, T | 181 | 2016 | WE: Physical | 0 | 0 | 204 | NR | 2 | NR | 2 | 6.8 | 0 | China | 0 | 1 |  |
| Yan, L | 182 | 2019 |  | 0 | 0 | 211 | mean=22.49 (SD=3.59) | 0 | 43.6%female | 2 | 5.8 | 2 | Japan | 0 | 1 |  |
| Yang, J | 183 | 2020 |  | 0 | 0 | 260 | <25 (21.5%), 26-35 (73.8%), 36-45 (3.8%), >45 (0.8%) | 0 | 62.7% female | 2 | 5.8 | 3 | China | 0 | 1 |  |
| Yoon, D | 184 | 2020 |  | 1 | 0 | 93 | mean=29.74 (SD=8.11) | 0 | 61% female | 2 | 7.9 | 3 | South Korea | 1 | 1 |  |
| Yoon, H | 185 | 2015 | WE: Overall | 0 | 0 | 271 | mean=39.93 (SD=8.92) | 0 | 47% female | 2 | 7.4 | 0 | South Korea | 1 | 1 |  |
| Yoon, H | 185 | 2015 | WE: Cognitive | 0 | 0 | 271 | mean=39.93 (SD=8.92) | 0 | 47% female | 2 | 7.4 | 0 | South Korea | 1 | 1 |  |
| Yoon, H | 185 | 2015 | WE: Emotional | 0 | 0 | 271 | mean=39.93 (SD=8.92) | 0 | 47% female | 2 | 7.4 | 0 | South Korea | 1 | 1 |  |
| Yoon, H | 185 | 2015 | WE: Physical | 0 | 0 | 271 | mean=39.93 (SD=8.92) | 0 | 47% female | 2 | 7.4 | 0 | South Korea | 1 | 1 |  |
| Young, G | 186 | 2012 | JP: Time 1 | 1 | 0 | 171 | NR | 2 | NR | 2 | 7.4 | 1 | United States | 0 | 0 |  |
| Young, G | 186 | 2012 | JP: Time 2 | 1 | 0 | 171 | NR | 2 | NR | 2 | 7.4 | 1 | United States | 0 | 0 |  |
| Young, G | 186 | 2012 | JP: Time 3 | 1 | 0 | 171 | NR | 2 | NR | 2 | 7.4 | 1 | United States | 0 | 0 |  |
| Young, G | 186 | 2012 | JP: Time 4 | 1 | 0 | 171 | NR | 2 | NR | 2 | 7.4 | 1 | United States | 0 | 0 |  |
| Young, G | 186 | 2012 | JP: Time 5 | 1 | 0 | 171 | NR | 2 | NR | 2 | 7.4 | 1 | United States | 0 | 0 |  |
| Young, G | 186 | 2012 | JP: Time 6 | 1 | 0 | 171 | NR | 2 | NR | 2 | 7.4 | 1 | United States | 0 | 0 |  |
| Zeijen, M | 187 | 2020 |  | 1 | 0 | 111 | mean=40.02, (SD=11.42) | 2 | 34% female | 2 | 5.8 | 3 | Netherlands | 1 | 0 |  |
| Zhang, J | 188 | 2016 | Study 1 | 1 | 1 | 415 | NR | 2 | 33.2% female | 2 | 5.2 | 0 | China | 0 | 1 |  |
| Zhang, J | 188 | 2016 | Study 2 | 1 | 1 | 139 | NR | 2 | 54% female | 2 | 5.2 | 0 | China | 0 | 1 |  |
| Zhao, X | 189 | 2016 |  | 0 | 0 | 401 | mean= 25.75, SD=6.45 | 0 | 54.3% female | 2 | 7.4 | 3 | China | 0 | 1 | 0 |
| Zhou, Q | 190 | 2019 |  | 1 | 0 | 648 | 26-35 (52.6%) | 2 | 52.6% female | 2 | 8.9 | 3 | China | 0 | 1 |  |

*Note.* ^a^Studies including multiple within-study (W-S) measures (meas.) or included multiple measures of an effect across time points; ^b^Sex distribution expressed as percentage of females in the sample; ^c^Age covariate – studies classified as younger (coded o), younger (1), or balanced (2) age samples; ^d^Sex covariate – studies classified as having predominantly female (0), predominantly male (1), or balanced (2) sex samples; ^e^Work type moderator variable classified workers in primarily for-profit (0), public service (1), or mixed for-profit and public (2) settings; ^f^Employee type moderator variable classified as studies with corporate employees (0), healthcare workers (1), teachers (2), or other (3) as the primary worker type in the sample; ^g^GDP code classified as studies on samples from the top 10 GDP countries (0) or countries outside the top 10 GDP (1); ^h^Cultural orientation code classified as individualist (0) or collectivist (1) according to sample country; ^i^Leader autonomy support (LAS) proximity, coded as proximal (0) or distal (1) support; Emp. code = Employee type moderator variable; LAS code = Leader autonomy support moderator variable; WB = Well being; JP = Job performance; NSUP = Need support; AUT = Autonomy need satisfaction; REL = Relatedness need satisfaction; COM = Competence need satisfaction; POP = Population sampled; TU = Employee turnover; IM = Intrinsic motivation; ER = External regulation; BU = Burnout; CS = Cross-sectional study design; LS = Longitudinal study design; EXP = Experimental study design; NR = Data not reported.

Supplement G

*Quality Assessment Checklist for Survey Studies in Psychology (Q-SSP) and Guide*

| Study: | | | | | |
| --- | --- | --- | --- | --- | --- |
| The Q-SSP is meant to be scored with the use of its guide; please, refer to the guide below. | | | | | |
| **Research**  **domain** | **Quality item** | **Yes** | **No** | **Not stated clearly** | **N/A** |
| Introduction  (Rationale) | 1. Was the problem or phenomenon under investigation defined, described, and justified? |  |  |  |  |
| Introduction  (Rationale) | 2. Was the population under investigation defined, described, and justified? |  |  |  |  |
| Introduction  (Rationale) | 3. Were specific research questions and/or hypotheses stated? |  |  |  |  |
| Introduction  (Variables) | 4. Were operational definitions of all study variables provided? |  |  |  |  |
| Participants  (Sampling) | 5. Were participant inclusion criteria stated? |  |  |  |  |
| Participants  (Sampling) | 6. Was the participant recruitment strategy described? |  |  |  |  |
| Participants  (Sampling) | 7. Was a justification/ rationale for the sample size provided? |  |  |  |  |
| Data  (Collection) | 8. Was the attrition rate provided?  (applies to cross-sectional and prospective studies) |  |  |  |  |
| Data  (Analyses) | 9. Was a method of treating attrition provided?  (applies to cross-sectional and prospective studies) |  |  |  |  |
| Data  (Analyses) | 10. Were the data analysis techniques justified (i.e., was the link between hypotheses/ aims / research questions and data analyses explained)? |  |  |  |  |
| Data  (Measures) | 11. Were the measures provided in the report (or in a supplement) in full? |  |  |  |  |
| Data  (Measures) | 12. Was evidence provided for the validity of all the measures (or instrument) used? |  |  |  |  |
| Data  (Collection) | 13. Was information provided about the person(s) who collected the data (e.g., training, expertise, other demographic characteristics)? |  |  |  |  |
| Data  (Collection) | 14. Was information provided about the context (e.g., place) of data collection? |  |  |  |  |
| Data  (Collection) | 15. Was information provided about the duration (or start and end date) of data collection? |  |  |  |  |
| Data  (Results) | 16. Was the study sample described in terms of key demographic characteristics? |  |  |  |  |
| Data  (Discussion) | 17. Was discussion of findings confined to the population from which the sample was drawn? |  |  |  |  |
| Ethics | 18. Were participants asked to provide (informed) consent or assent? |  |  |  |  |
| ­Ethics | 19. Were participants debriefed at the end of data collection? |  |  |  |  |
| Ethics | 20. Were funding sources or conflicts of interest disclosed? |  |  |  |  |
| **SCORING (optional; see guide below)**  **Overall Quality Score (%):**  Compute an overall study quality score expressed as a percentage by dividing  YES (Y) scores by the Total (T) number of **APPLICABLE** items and multiplying  by 100. If a report fails to attain a Y score for 5 of the items, then it may be classed as of questionable quality.  Specifically:  When (T) = 20, then a score of Y/T ≥ 75% may be considered acceptable quality.  When (T) = 19, then a score of Y/T ≥ 73% may be considered acceptable quality.  When (T) = 18, then a score of Y/T ≥ 72% may be considered acceptable quality.  When (T) = 17, then a score of Y/T ≥ 70% may be considered acceptable quality.  If Y/T <75% or < 73% or < 72%, or < 70% (depending on number of applicable items),  then study is of questionable quality.  **Domain Quality Scores**  Express domain quality scores as a simple ratio of the (Y) items, divided by the (T) applicable items.  **(4 items) Introduction (Rationale/Variables) score: /4**  **(3 items) Participants (Sampling/Recruitment) score: /3**  **(10 items) Data (Collection/Analyses/Measures/Results/Discussion) score: /10**  **(3 items) Ethics score: /3**    IN A DATA FILE, ASSIGN **1** FOR YES SCORES; **0** FOR NO OR NOT STATED CLCLEARLY; AND **2** = FOR NOT APPLICABLE. | |  |  |  |  |

Supplement H

**Table H1**

*Summary of Article Quality Assessed by the Quality Assessment Checklist for Survey Studies in Psychology (Q-SSP) by Category and Item*

| Category and Item | Category Midpoint | Category  Mean | Proportion of articles meeting quality criteria (%) |
| --- | --- | --- | --- |
| Introduction | 4 | 2.92 |  |
| 1. Was the problem or phenomenon under investigation defined, described, and justified? |  |  | 98.7 |
| 2. Was the population under investigation defined, described, and justified? |  |  | 70.8 |
| 3. Were specific research questions or hypotheses stated? |  |  | 98.7 |
| 4. Were operational definitions of all study variables provided? |  |  | 95.5 |
| Participants | 1 | 1.24 |  |
| 5. Were participant inclusion criteria stated? |  |  | 53.9 |
| 6. Was the participant recruitment strategy described? |  |  | 90.9 |
| 7. Was a justification/ rationale for the sample size provided? |  |  | 9.7 |
| Data | 5 | 4.38 |  |
| 8. Was the attrition rate provided?  (applies to cross-sectional and prospective studies) |  |  | 36.6 |
| 9. Was a method of treating attrition provided?  (applies to cross-sectional and prospective studies) |  |  | 32.0 |
| 10. Were the data analysis techniques justified (i.e., was the link between hypotheses/ aims / research questions and data analyses explained)? |  |  | 88.9 |
| 11. Were the measures provided in the report (or in a supplement) in full? |  |  | 43.1 |
| 12. Was evidence provided for the validity of all the measures (or instrument) used? |  |  | 85.0 |
| 13. Was information provided about the person(s) who collected the data (e.g., training, expertise, other demographic characteristics)? |  |  | 15.7 |
| 14. Was information provided about the context (e.g., place) of data collection? |  |  | 80.4 |
| 15. Was information provided about the duration (or start and end date) of data collection? |  |  | 14.4 |
| 16. Was the study sample described in terms of key demographic characteristics? |  |  | 70.6 |
| 17. Was discussion of findings confined to the population from which the sample was drawn? |  |  | 82.4 |
| Ethics | 0.5 | 0.66 |  |
| 18. Were participants asked to provide (informed) consent or assent? |  |  | 25.5 |
| 19. Were participants debriefed at the end of data collection? |  |  | 5.2 |
| 20. Were funding sources or conflicts of interest disclosed? |  |  | 51.6 |
|  |  |  |  |

Supplement I

*Moderators of Correlations*

In addition to testing the effects of moderators on effects in our truncated model, we also conducted a supplementary analysis testing the effects of our moderators on each zero-order averaged bias corrected correlation from the multilevel meta-analysis. These are the averaged correlations among study constructs that comprised the correlation matrix ultimately used as input for our model tests. This analysis serves as a supplement to the analysis reported in the main article. The moderator analyses for the model test supersedes this analysis because the latter focused on unique effects (i.e., accounts for the effects of the other constructs) rather than univariate analysis represented by the zero-order correlations which does not correct for such attenuation. To conduct this supplementary analysis, we employed a series of meta-regression analyses in which we regressed each correlation on the set of moderator variables expressed as a contrast code – all moderators were categorical with the exception of study quality, a continuous moderator.

Our moderator predictions for the correlation analysis were identical to those for the moderation analysis for the model tests. Specifically, for our work type moderator, we expected larger averaged correlations between controlled motivation and adaptive workplace outcomes in studies on for-profit work environments. Regarding employee type, we hypothesized larger correlations between autonomous motivation and adaptive workplace outcomes among studies targeting teachers and healthcare workers. For sample country of origin GDP, we hypothesized larger correlations between autonomous motivation and workplace outcomes, and smaller correlations between controlled motivation and workplace outcomes, in employee samples of studies whose country of origin was outside of the top 10 GDP group. With respect to cultural orientation, we expected no differences in the size of the averaged correlations consistent with previous cross-cultural research in other contexts (Chirkov et al., 2003; Sheldon et al., 2004). Finally, we hypothesized larger correlations between psychological need support and need satisfaction, and between need support and autonomous forms of motivation, in studies where leaders were more proximal to their employees. We also included demographic (e.g., sample average age, predominant sample sex) and methodological (e.g., study design, study quality) constructs as covariates in our meta-regression analyses.

**Results**

Our meta-regression analyses indicated that correlations between autonomous forms of motivation and maladaptive workplace outcomes were larger in healthcare workers compared to corporate employees, while there were no effects on adaptive outcomes and autonomous motivation. However, the relationship between controlled motivation and adaptive outcomes was larger in teachers and other mixed-work employees compared to corporate employees. Next, we observed larger correlations between basic psychological need satisfaction and autonomous forms of motivation in samples from countries outside of the top 10 GDP ranks compared to the top 10 countries. But the correlations between need satisfaction and various workplace outcomes including turnover, job performance, and work engagement were all smaller in samples from countries outside of the top 10 countries.

In addition, our analyses indicated that correlations between competence and relatedness need satisfaction and intrinsic motivation were larger in studies originating in countries outside of the top 10 ranking by GDP. Further, correlations between extrinsic motivation and job satisfaction and work engagement were larger in studies comprising teachers relative to those comprising corporate employees and those comprising other mixed-work employees relative to those comprising corporate employees, respectively. Finally, correlations between intrinsic motivation and burnout were larger in studies on samples comprising healthcare workers compared to those comprising corporate employees. Due to missing cells in the matrix of correlations at each level of the moderator variable, we were not able to test proximity of leader support or cultural orientation in our meta-regression moderator analyses.

**Discussion**

Our goal was to assess whether patterns of moderator effects on the averaged zero-order correlations from our multilevel meta-analysis were consistent with theory, that is, could we identify differences in correlations between self-determination theory constructs and motivational and adaptive and maladaptive outcomes (e.g., correlations between perceived need support and need satisfaction constructs, between need support and autonomous forms of motivation, or between autonomous forms of motivation and adaptive outcomes)? We observed relatively few consistent effects of the work and employee type moderators on averaged correlations save a few isolated effects, none of which were indicative of a characteristic pattern. By contrast, we observed consistent moderating effects of sample country GDP on correlations between need satisfaction and motivation and need satisfaction and work outcomes. Specifically, the correlations between competence and relatedness need satisfaction and autonomous motivation were larger in studies originating from countries ranked outside the top 10 by GDP than those from countries ranked inside the top 10, while correlations between need satisfaction constructs and turnover, job performance, and work engagement were smaller in studies originating from countries ranked outside the top 10 by GDP. This finding was consistent with our hypotheses, our findings for these effects in our truncated model, and other studies and suggest that even though individuals from countries outside of the top 10 GDP may be more likely to experience autonomous motivation when needs are satisfied, autonomous motivation is less pertinent when it comes to work outcomes. This may be because employees are more focused on external motives such as pay given the far greater need to provide for themselves rather than others as they cannot rely on governmental services and resources (Ryan et al., 1999).

**References**

Chirkov, V. I., Ryan, R. M., Kim, Y., & Kaplan, U. (2003). Differentiating autonomy from individualism and independence: A self-determination theory perspective on internalization of cultural orientations and well-being. *Journal of Personality and Social Psychology, 84*, 97-110. https://doi.org/10.1037/0022-3514.84.1.97

Ryan, R. M., Chirkov, V. I., Little, T. D., Sheldon, K. M., Timoshina, E., & Deci, E. L. (1999). The American dream in Russia: Extrinsic aspirations and well-being in two cultures. *Personality and Social Psychology Bulletin, 25*, 1509-1524. https://doi.org/10.1177/01461672992510007

Sheldon, K. M., Elliot, A. J., Ryan, R. M., Chirkov, V., Kim, Y., Wu, C., Demir, M., & Sun, Z. (2004). Self-concordance and subjective well-being in four cultures. *Journal of Cross-Cultural Psychology, 35*, 209-233. <https://doi.org/10.1177/0022022103262245>

Supplement J

**Table J1**

*Results of Multilevel Meta-Analysis Models of Zero-Order Correlations Among Study Constructs Including and Excluding Moderators*

| Model | *k* | *r^b,c^* | 95% confidence interval of *r* | | σ^2^ within | σ^2^ between | *Q* (residual heterogeneity) | AIC | *I*^2^ | var σ^2^ | |  | Moderator tests | | |
| --- | --- | --- | --- | --- | --- | --- | --- | --- | --- | --- | --- | --- | --- | --- | --- |
|  |  |  | LL | UL |  |  |  |  |  | within | between |  | *Q* | df | LRT |
| IM-EM | 70 | 0.126^***^ | 0.056 | 0.197 | .014 | .053 | 2124.340^***^ | 4.014 | 97.31 | 20.23 | 77.08 |  | ‒ | ‒ | ‒ |
|  |  | -0.221 | -0.687 | 0.245 | .008 | .058 | 1494.493^***^ | 10.580 | 97.01 | 11.74 | 85.27 |  | 9.320 | 7 | 7.434 |
|  |  | -0.312 | -0.766 | 0.142 | .008 | .043 | 1235.667^***^ | 4.569 | 96.15 | 15.49 | 80.65 |  | 22.561^*^ | 10 | 19.444^*^ |
| IM-AUT | 38 | 0.394^***^ | 0.339 | 0.448 | .018 | .004 | 320.332^***^ | -24.641 | 88.33 | 71.82 | 16.51 |  | ‒ | ‒ | ‒ |
|  |  | 0.308^*^ | 0.012 | 0.604 | .015 | <.001 | 237.266^***^ | -21.834 | 82.32 | 81.36 | <0.01 |  | 15.977^*^ | 8 | 13.196 |
|  |  | 0.145 | -0.251 | 0.538 | .014 | <.001 | 230.629^***^ | -18.522 | 81.07 | 81.07 | <0.01 |  | 19.538 | 11 | 15.881 |
| IM-COM | 18 | 0.407^***^ | 0.322 | 0.493 | .011 | .011 | 143.986^***^ | -10.085 | 88.05 | 43.93 | 44.12 |  | ‒ | ‒ | ‒ |
|  |  | -0.796^**^ | -0.336 | -0.256 | .008 | <.001 | 53.655^***^ | -8.338 | 69.15 | 69.15 | <0.01 |  | 22.040^**^ | 7 | 12.253 |
|  |  | -1.406^***^ | -2.066 | -0.746 | .003 | <.001 | 33.265^***^ | -11.390 | 46.72 | 46.72 | <0.01 |  | 53.672^***^ | 10 | 21.305^*^ |
| IM-REL | 20 | 0.358^***^ | 0.301 | 0.414 | .013 | <.001 | 128.930^***^ | -19.801 | 84.10 | 81.28 | 2.82 |  | ‒ | ‒ | ‒ |
|  |  | -0.101 | -0.511 | 0.309 | .008 | <.001 | 78.089^***^ | -12.648 | 74.85 | 74.85 | <0.01 |  | 8.520 | 7 | 6.847 |
|  |  | -0.491^*^ | -0.915 | -0.066 | .005 | <.001 | 51.439^***^ | -16.299 | 61.18 | 61.18 | <0.01 |  | 23.548^**^ | 9 | 14.498 |
| IM-NSUP | 29 | 0.357^***^ | 0.312 | 0.401 | .009 | .002 | 132.813^***^ | -34.487 | 80.59 | 66.68 | 13.91 |  | ‒ | ‒ | ‒ |
|  |  | -0.063 | -0.354 | 0.229 | .006 | <.001 | 82.935^***^ | -30.083 | 76.01 | 76.01 | <0.01 |  | 14.595^*^ | 7 | ‒ |
|  |  | <0.001 | -0.344 | 0.344 | .005 | <.001 | 77.605^***^ | -26.344 | 77.19 | 77.19 | <0.01 |  | 17.844 | 10 | ‒ |
| IM-IG^a^ | 3 | 0.598^***^ | 0.553 | 0.643 | <.001 | <.001 | 1.161 | -5.867 | 0.00 | <0.01 | <0.01 |  | ‒ | ‒ | ‒ |
| IM-IJ | 19 | 0.202^***^ | 0.100 | 0.304 | .003 | .032 | 253.369^***^ | -7.376 | 93.01 | 8.62 | 84.39 |  | ‒ | ‒ | ‒ |
|  |  | 0.244 | -0.557 | 1.046 | .004 | .024 | 172.306^***^ | 1.499 | 90.65 | 11.65 | 78.99 |  | 3.487 | 6 | 3.126 |
|  |  | 0.048 | -0.941 | 1.037 | .004 | .018 | 125.498^***^ | 6.178 | 87.90 | 15.29 | 72.61 |  | 8.270 | 10 | 6.446 |
| IM-ID | 24 | 0.545^***^ | 0.446 | 0.644 | .003 | .040 | 725.101^***^ | -8.826 | 94.66 | 7.00 | 87.66 |  | ‒ | ‒ | ‒ |
|  |  | 0.241 | -0.460 | 0.943 | .003 | .031 | 430.288^***^ | 1.018 | 92.93 | 8.47 | 84.46 |  | 4.746 | 7 | 4.156 |
|  |  | 0.130 | -0.576 | 0.837 | .004 | .022 | 226.096^***^ | 2.895 | 90.61 | 12.74 | 77.86 |  | 11.475 | 10 | 8.280 |
| IM-JP | 48 | 0.290^***^ | 0.246 | 0.333 | .004 | .010 | 321.211^***^ | -53.432 | 84.66 | 25.42 | 59.23 |  | ‒ | ‒ | ‒ |
|  |  | 0.406^**^ | 0.135 | 0.678 | .005 | .010 | 246.544^***^ | -41.906 | 85.89 | 23.71 | 62.18 |  | 4.557 | 7 | ‒ |
|  |  | 0.387^**^ | 0.109 | 0.664 | .004 | .009 | 235.313^***^ | -38.337 | 85.94 | 25.32 | 60.63 |  | 7.268 | 10 | ‒ |
| IM-JS | 30 | 0.396^***^ | 0.317 | 0.476 | .027 | .014 | 499.914^***^ | -2.442 | 93.92 | 62.62 | 31.30 |  | ‒ | ‒ | ‒ |
|  |  | 0.297 | -0.253 | 0.847 | .026 | .002 | 345.588^***^ | 3.669 | 89.75 | 83.48 | 6.27 |  | 11.979 | 8 | 9.889 |
|  |  | 0.432 | -0.101 | 0.964 | .023 | <.001 | 270.618^***^ | 5.151 | 87.68 | 87.68 | <0.01 |  | 19.057 | 11 | 14.407 |
| IM-TU | 71 | -0.289^***^ | -0.344 | -0.235 | .025 | .010 | 1090.490^***^ | -26.577 | 95.13 | 67.86 | 27.27 |  | ‒ | ‒ | ‒ |
|  |  | -0.323 | -0.696 | 0.049 | .024 | .011 | 1067.314^***^ | -12.563 | 95.64 | 66.10 | 29.54 |  | 1.261 | 8 | ‒ |
|  |  | -0.255 | -0.558 | 0.048 | .027 | <.001 | 852.427^***^ | -17.797 | 94.65 | 94.65 | <0.01 |  | 14.202 | 11 | ‒ |
| IM-BU | 35 | -0.305^***^ | -0.361 | -0.249 | .003 | .016 | 418.368^***^ | -34.517 | 92.87 | 14.95 | 77.92 |  | ‒ | ‒ | ‒ |
|  |  | -0.257 | -0.689 | 0.174 | <.001 | .016 | 287.698^***^ | -30.885 | 89.98 | <0.01 | 89.98 |  | 17.028^*^ | 7 | 10.368 |
|  |  | -0.552^*^ | -1.037 | -0.067 | <.001 | .013 | 262.080^***^ | -29.778 | 87.47 | <0.01 | 87.47 |  | 22.839^*^ | 10 | 15.261 |
| IM-WE | 63 | 0.459^***^ | 0.400 | 0.516 | .002 | .029 | 957.575^***^ | -61.601 | 94.89 | 5.71 | 89.18 |  | ‒ | ‒ | ‒ |
|  |  | 0.586^**^ | 0.196 | 0.976 | .002 | .023 | 708.110^***^ | -56.157 | 93.47 | 6.02 | 87.46 |  | 11.537 | 8 | 10.554 |
|  |  | 0.573^*^ | 0.097 | 1.050 | .002 | .020 | 586.429^***^ | -53.924 | 92.66 | 6.82 | 85.84 |  | 16.658 | 11 | 14.323 |
| IM-WB | 10 | 0.451^***^ | 0.329 | 0.572 | <.001 | .028 | 137.180^***^ | -6.037 | 92.68 | <0.01 | 92.68 |  | ‒ | ‒ | ‒ |
|  |  | -0.189 | -0.862 | 0.485 | <.001 | .012 | 36.822^***^ | -1.285 | 81.40 | <0.01 | 81.40 |  | 8.275 | 5 | 5.249 |
|  |  | -0.103 | -0.871 | 0.665 | <.001 | .011 | 36.327^***^ | 2.280 | 84.52 | <0.01 | 84.52 |  | 9.063 | 7 | 5.683 |
| EM-AUT | 21 | -0.038 | -0.090 | 0.014 | .001 | .008 | 83.100^***^ | -26.944 | 74.83 | 6.87 | 67.96 |  | ‒ | ‒ | ‒ |
|  |  | 0.171^***^ | -0.109 | 0.451 | <.001 | .003 | 37.937^***^ | -25.961 | 46.80 | <0.01 | 46.80 |  | 18.577^**^ | 7 | 13.017 |
|  |  | 0.282 | -0.098 | 0.662 | <.001 | .002 | 35.955^***^ | -20.795 | 44.57 | <0.01 | 44.57 |  | 20.340^*^ | 10 | 13.851 |
| EM-COM^a^ | 7 | -0.034 | -0.163 | 0.095 | .026 | <.001 | 45.181^***^ | 1.530 | 86.26 | 86.26 | <0.01 |  | ‒ | ‒ | ‒ |
|  |  | 0.661^***^ | 0.403 | 0.919 | <.001 | <.001 | 0.963 | -8.279 | 0.00 | <0.01 | <0.01 |  | 44.218^***^ | 5 | 19.809^**^ |
| EM-REL^a^ | 9 | -0.093^*^ | -0.175 | -0.011 | .013 | <.001 | 63.756^***^ | -5.890 | 85.55 | 83.54 | 2.01 |  | ‒ | ‒ | ‒ |
| EM-NSUP | 13 | 0.034 | -0.071 | 0.140 | .006 | .024 | 197.569^***^ | -3.430 | 93.35 | 18.49 | 74.86 |  | ‒ | ‒ | ‒ |
|  |  | 0.382 | -0.136 | 0.899 | .004 | .002 | 48.398^***^ | -7.913 | 73.41 | 46.88 | 26.53 |  | 35.666^***^ | 6 | 16.483^*^ |
|  |  | 0.722 | -0.113 | 1.557 | .001 | <.001 | 18.242^***^ | -13.763 | 35.75 | 35.75 | <0.01 |  | 117.859^***^ | 9 | 28.334^***^ |
| EM-IG^a^ | 3 | 0.029 | -0.054 | 0.113 | .002 | .002 | 7.737^*^ | -0.906 | 59.71 | 29.86 | 29.86 |  | ‒ | ‒ | ‒ |
| EM-IJ | 19 | 0.285^***^ | 0.205 | 0.364 | .001 | .019 | 150.206^***^ | -15.807 | 88.10 | 4.01 | 84.09 |  | ‒ | ‒ | ‒ |
|  |  | -0.366 | -0.765 | 0.033 | <.001 | .005 | 42.798^***^ | -20.430 | 61.59 | <0.01 | 61.59 |  | 31.368^***^ | 7 | 18.623^**^ |
|  |  | -0.387 | -0.780 | 0.006 | .003 | <.001 | 36.515^***^ | -15.710 | 47.61 | 47.61 | <0.01 |  | 56.666^***^ | 10 | 19.903^*^ |
| EM-ID | 21 | 0.130^**^ | 0.039 | 0.221 | .042 | <.001 | 288.140^***^ | 0.990 | 94.45 | 94.45 | <0.01 |  | ‒ | ‒ | ‒ |
|  |  | 0.608 | -0.098 | 1.313 | .004 | .031 | 223.204^***^ | 4.215 | 93.07 | 11.18 | 81.89 |  | 18.464^**^ | 6 | 8.776 |
|  |  | 0.513 | -0.099 | 1.124 | .005 | .020 | 173.717^***^ | 1.750 | 90.70 | 17.65 | 73.04 |  | 23.408^**^ | 7 | 13.240 |
| EM-JP | 22 | 0.063 | -0.026 | 0.152 | .003 | .024 | 181.002^***^ | -12.769 | 91.75 | 10.65 | 81.10 |  | ‒ | ‒ | ‒ |
|  |  | -0.420^*^ | -0.803 | -0.036 | .009 | .006 | 116.896^***^ | -5.209 | 84.68 | 51.43 | 33.25 |  | 10.735 | 7 | 6.440 |
|  |  | -0.516^**^ | -0.858 | -0.173 | .013 | <.001 | 97.737^***^ | -3.183 | 82.07 | 82.07 | <0.01 |  | 19.319^*^ | 9 | 8.414 |
| EM-JS | 15 | 0.011 | -0.107 | 0.129 | .024 | .020 | 361.392^***^ | 1.679 | 95.39 | 52.09 | 43.30 |  | ‒ | ‒ | ‒ |
|  |  | -0.283 | -0.833 | 0.266 | .011 | <.001 | 78.016^***^ | -1.155 | 83.10 | 83.10 | <0.01 |  | 34.956^***^ | 7 | 16.834^*^ |
|  |  | -0.088 | -0.450 | 0.275 | .003 | <.001 | 34.064^***^ | -10.738 | 56.03 | 56.03 | <0.01 |  | 120.725^***^ | 10 | 32.418^***^ |
| EM-TU | 23 | -0.008 | -0.090 | 0.075 | .005 | .015 | 261.714^***^ | -23.662 | 93.43 | 24.90 | 68.53 |  | ‒ | ‒ | ‒ |
|  |  | 0.492^*^ | 0.012 | 0.971 | .005 | <.001 | 75.884^***^ | -27.841 | 77.46 | 77.46 | <0.01 |  | 42.949^***^ | 6 | 16.179^*^ |
|  |  | 0.710 | -0.176 | 1.596 | .004 | <.001 | 69.549^***^ | -24.221 | 75.86 | 75.86 | <0.01 |  | 50.142^***^ | 9 | 18.558^*^ |
| EM-BU^a^ | 21 | 0.124^***^ | 0.074 | 0.174 | .004 | .005 | 193.949^***^ | -30.632 | 88.54 | 39.39 | 49.16 |  | ‒ | ‒ | ‒ |
|  |  | -0.150 | -0.514 | 0.215 | <.001 | .005 | 103.807^***^ | -31.774 | 79.28 | 0.42 | 78.86 |  | 22.416^***^ | 6 | 13.143^*^ |
| EM-WE | 39 | 0.050 | -0.051 | 0.150 | <.001 | .056 | 721.946^***^ | -42.815 | 97.72 | 0.25 | 97.48 |  | ‒ | ‒ | ‒ |
|  |  | 0.072 | -0.632 | 0.777 | <.001 | .048 | 445.911^***^ | -33.149 | 97.27 | 0.33 | 96.94 |  | 2.625 | 6 | 2.335 |
|  |  | -0.077 | -0.657 | 0.503 | <.001 | .030 | 317.509^***^ | -38.839 | 95.71 | 0.47 | 95.24 |  | 16.667^*^ | 8 | 12.024 |
| EM-WB^a^ | 8 | -0.076 | -0.204 | 0.053 | .009 | .017 | 106.168^***^ | -0.712 | 93.47 | 33.06 | 60.41 |  | ‒ | ‒ | ‒ |
|  |  | 2.020 | -0.083 | 4.123 | .001 | <.001 | 10.869^**^ | -8.765 | 23.65 | 23.65 | <0.01 |  | 73.948^***^ | 5 | 18.053^**^ |
| AUT-COM | 42 | 0.519^***^ | 0.464 | 0.575 | .015 | .011 | 708.168^***^ | -23.515 | 94.57 | 54.63 | 39.94 |  | ‒ | ‒ | ‒ |
|  |  | 0.469^*^ | 0.025 | 0.912 | .015 | .008 | 502.284^***^ | -12.615 | 91.80 | 60.73 | 31.07 |  | 5.604 | 8 | 5.100 |
|  |  | 0.456 | -0.003 | 0.915 | .015 | .006 | 459.978^***^ | -10.143 | 90.69 | 65.79 | 24.90 |  | 9.971 | 11 | 8.628 |
| AUT-REL | 52 | 0.485^***^ | 0.440 | 0.530 | .015 | .006 | 716.655^***^ | -42.357 | 93.70 | 66.02 | 27.68 |  | ‒ | ‒ | ‒ |
|  |  | 0.608^***^ | 0.285 | 0.932 | .013 | .003 | 572.492^***^ | -36.492 | 90.65 | 75.75 | 14.90 |  | 11.620 | 8 | 10.135 |
|  |  | 0.679^***^ | 0.321 | 1.036 | .013 | .002 | 515.439^***^ | -32.529 | 89.77 | 77.14 | 12.63 |  | 14.269 | 11 | 12.172 |
| AUT-NSUP | 29 | 0.464^***^ | 0.405 | 0.524 | .023 | <.001 | 400.863^***^ | -16.983 | 89.97 | 89.97 | <0.01 |  | ‒ | ‒ | ‒ |
|  |  | 0.925^***^ | 0.569 | 0.282 | .014 | <.001 | 183.040^***^ | -15.090 | 82.76 | 82.76 | <0.01 |  | 14.941^*^ | 7 | 12.107 |
|  |  | 1.359^***^ | 0.795 | 1.924 | .012 | <.001 | 158.995^***^ | -13.493 | 78.57 | 78.57 | <0.01 |  | 22.135^*^ | 10 | 16.509 |
| AUT-JP | 23 | 0.270^***^ | 0.212 | 0.328 | .016 | <.001 | 120.514^***^ | -17.095 | 84.46 | 84.46 | <0.01 |  | ‒ | ‒ | ‒ |
|  |  | 0.320 | -0.163 | 0.802 | .009 | <.001 | 73.140^***^ | -12.444 | 74.56 | 74.56 | <0.01 |  | 11.513 | 7 | 9.349 |
|  |  | 0.528^*^ | 0.089 | 0.967 | .004 | <.001 | 51.769^***^ | -15.695 | 56.91 | 56.91 | <0.01 |  | 29.281^**^ | 10 | 18.601^*^ |
| AUT-JS | 47 | 0.413^***^ | 0.354 | 0.471 | .004 | .022 | 604.282^***^ | -44.046 | 95.44 | 16.14 | 79.31 |  | ‒ | ‒ | ‒ |
|  |  | 0.148 | -0.086 | 0.383 | .004 | .007 | 301.806^***^ | -52.870 | 88.86 | 31.43 | 57.43 |  | 35.581^***^ | 8 | 24.824^**^ |
|  |  | 0.064 | -0.193 | 0.321 | .005 | .005 | 279.481^***^ | -50.016 | 86.54 | 44.10 | 42.44 |  | 45.443^***^ | 11 | 27.970^**^ |
| AUT-TU | 37 | -0.370^***^ | -0.435 | -0.305 | .023 | .007 | 467.884^***^ | -16.683 | 91.27 | 71.21 | 20.06 |  | ‒ | ‒ | ‒ |
|  |  | -0.653^***^ | -0.932 | -0.374 | .019 | <.001 | 273.255^***^ | -13.236 | 86.39 | 86.39 | <0.01 |  | 16.471^*^ | 8 | 12.553 |
|  |  | -0.527^**^ | -0.811 | -0.242 | .016 | <.001 | 230.228^***^ | -13.342 | 83.67 | 83.67 | <0.01 |  | 26.339^**^ | 11 | 18.659 |
| AUT-BU | 36 | -0.429^***^ | -0.496 | -0.362 | .009 | .011 | 441.961^***^ | -33.678 | 89.87 | 40.90 | 48.96 |  | ‒ | ‒ | ‒ |
|  |  | 0.114 | -0.372 | 0.600 | .010 | .003 | 260.154^***^ | -26.562 | 82.98 | 61.91 | 21.07 |  | 9.041 | 7 | 6.884 |
|  |  | -0.071 | -0.672 | 0.529 | .011 | .003 | 194.397^***^ | -23.240 | 78.71 | 76.63 | 2.08 |  | 14.852 | 10 | 9.561 |
| AUT-WE | 67 | 0.422^***^ | 0.374 | 0.471 | .007 | .014 | 702.252^***^ | -63.152 | 90.62 | 30.77 | 59.85 |  | ‒ | ‒ | ‒ |
|  |  | 0.516^**^ | 0.204 | 0.828 | .007 | .015 | 633.602^***^ | -51.290 | 90.11 | 27.68 | 62.43 |  | 2.211 | 7 | 2.138 |
|  |  | 0.574^**^ | 0.220 | 0.927 | .006 | .014 | 523.044^***^ | -46.707 | 89.16 | 27.59 | 61.57 |  | 3.704 | 10 | 3.555 |
| AUT-WB | 20 | 0.451^***^ | 0.374 | 0.528 | <.001 | .011 | 186.305^***^ | -36.920 | 84.05 | 1.20 | 82.85 |  | ‒ | ‒ | ‒ |
|  |  | 0.244 | -0.375 | 0.862 | <.001 | .002 | 36.034^**^ | -38.945 | 43.50 | <0.01 | 43.50 |  | 25.319^**^ | 5 | 12.025^*^ |
|  |  | -0.306 | -0.929 | 0.317 | <.001 | <.001 | 8.728 | -49.367 | 0.00 | <0.01 | <0.01 |  | 177.577^***^ | 8 | 28.447^**^ |
| COM-REL | 37 | 0.464^***^ | 0.420 | 0.509 | .016 | <.001 | 367.138^***^ | -33.765 | 92.16 | 92.16 | <0.01 |  | ‒ | ‒ | ‒ |
|  |  | 0.687^***^ | 0.352 | 1.022 | .009 | <.001 | 236.319^***^ | -34.339 | 82.80 | 82.20 | <0.01 |  | 21.016^**^ | 8 | 16.574^*^ |
|  |  | 0.772^**^ | 0.288 | 1.255 | .008 | <.001 | 208.559^***^ | -32.286 | 79.97 | 79.97 | <0.01 |  | 27.220^**^ | 11 | 20.521^*^ |
| COM-NSUP^a^ | 12 | 0.437^***^ | 0.305 | 0.568 | .016 | .014 | 172.446^***^ | -2.150 | 92.53 | 50.46 | 42.07 |  | ‒ | ‒ | ‒ |
| COM-ID^a^ | 3 | 0.473^***^ | 0.397 | 0.549 | .001 | .001 | 5.093 | -1.786 | 41.39 | 20.69 | 20.70 |  | ‒ | ‒ | ‒ |
| COM-JP | 9 | 0.414^***^ | 0.323 | 0.504 | <.001 | .012 | 42.336^***^ | -7.302 | 80.33 | 1.97 | 78.36 |  | ‒ | ‒ | ‒ |
|  |  | 0.726^***^ | 0.354 | 1.098 | <.001 | <.001 | 1.878 | -14.710 | 0.00 | <0.01 | <0.01 |  | 40.46^***^ | 6 | 19.408^**^ |
|  |  | 0.693^***^ | 0.311 | 1.075 | <.001 | <.001 | 1.286 | -13.301 | 0.00 | <0.01 | <0.01 |  | 41.050^***^ | 7 | 20.000^**^ |
| COM-JS | 18 | 0.439^***^ | 0.357 | 0.521 | .011 | .014 | 941.265^***^ | -8.687 | 95.80 | 41.23 | 54.59 |  | ‒ | ‒ | ‒ |
|  |  | 0.390 | -0.036 | 0.817 | .009 | <.001 | 102.555^***^ | -7.816 | 80.51 | 80.51 | <0.01 |  | 22.692^**^ | 7 | 13.129 |
|  |  | 0.415 | -0.179 | 1.009 | .009 | <.001 | 96.126^***^ | -2.051 | 79.81 | 79.81 | <0.01 |  | 23.168^*^ | 10 | 13.364 |
| COM-TU^a^ | 10 | -0.322^***^ | -0.412 | -0.232 | .017 | <.001 | 62.232^***^ | -4.131 | 84.40 | 84.40 | <0.01 |  | ‒ | ‒ | ‒ |
|  |  | -4.567^***^ | -6.934 | -2.200 | .001 | <.001 | 14.336^***^ | -1.750 | 26.99 | 26.99 | <0.01 |  | 31.288^***^ | 8 | 13.618 |
| COM-BU^a^ | 9 | -0.375^***^ | -0.448 | -0.302 | .009 | <.001 | 48.799^***^ | -6.985 | 82.26 | 82.26 | <0.01 |  | ‒ | ‒ | ‒ |
|  |  | 0.636 | -0.626 | 1.898 | <.001 | <.001 | 12.517^**^ | -7.951 | 0.00 | <0.01 | <0.01 |  | 36.281^***^ | 5 | 10.966 |
| COM-WE | 35 | 0.480^***^ | 0.398 | 0.563 | .007 | .023 | 451.157^***^ | -28.100 | 93.87 | 21.76 | 72.11 |  | ‒ | ‒ | ‒ |
|  |  | 0.430 | -0.369 | 1.229 | .005 | .021 | 334.004^***^ | -21.405 | 92.00 | 16.07 | 75.93 |  | 8.425 | 7 | 7.305 |
|  |  | 0.382 | -0.397 | 1.161 | .005 | .019 | 314.404^***^ | -17.401 | 91.24 | 17.75 | 73.49 |  | 10.796 | 10 | 9.300 |
| COM-WB^a^ | 6 | 0.498^***^ | 0.386 | 0.610 | .018 | <.001 | 59.881^***^ | -0.469 | 93.41 | 93.41 | <0.01 |  | ‒ | ‒ | ‒ |
| REL-NSUP^a^ | 19 | 0.412^***^ | 0.315 | 0.508 | .010 | .016 | 375.469^***^ | -11.736 | 92.87 | 35.14 | 57.72 |  | ‒ | ‒ | ‒ |
|  |  | 0.270 | -0.491 | 1.030 | .012 | <.001 | 121.247^***^ | -8.396 | 81.97 | 81.97 | <0.01 |  | 15.547^*^ | 6 | 8.660 |
| REL-IJ^a^ | 3 | 0.026 | -0.071 | 0.123 | .005 | <.001 | 11.039^**^ | -0.236 | 72.77 | 72.77 | <0.01 |  | ‒ | ‒ | ‒ |
| REL-ID^a^ | 5 | 0.325^***^ | 0.242 | 0.408 | .007 | <.001 | 19.922^***^ | -3.469 | 73.98 | 73.98 | <0.01 |  | ‒ | ‒ | ‒ |
| REL-JP^a^ | 7 | 0.308^***^ | 0.198 | 0.419 | .002 | .014 | 43.668 | -2.306 | 87.00 | 10.76 | 76.24 |  | ‒ | ‒ | ‒ |
|  |  | 0.792^***^ | 0.334 | 1.249 | <.001 | <.001 | 1.628 | -10.842 | 0.00 | <0.01 | <0.01 |  | 42.040^***^ | 5 | 18.536^**^ |
| REL-JS | 30 | 0.384^***^ | 0.334 | 0.433 | .007 | .004 | 157.045^***^ | -34.765 | 84.80 | 55.92 | 28.88 |  | ‒ | ‒ | ‒ |
|  |  | 0.376^**^ | 0.107 | 0.645 | .007 | <.001 | 99.795 | -28.239 | 64.77 | 64.77 | <0.01 |  | 10.142 | 7 | 7.475 |
|  |  | 0.588^**^ | 0.179 | 0.996 | .006 | <.001 | 82.582^***^ | -25.054 | 56.20 | 56.20 | <0.01 |  | 14.008 | 10 | 10.289 |
| REL-TU | 16 | -0.398^***^ | -0.517 | -0.279 | .019 | .026 | 358.763^***^ | 1.382 | 96.08 | 41.35 | 54.74 |  | ‒ | ‒ | ‒ |
|  |  | -1.145^**^ | -2.014 | -0.276 | .008 | <.001 | 71.004^***^ | -4.873 | 81.18 | 81.18 | <0.01 |  | 53.514^***^ | 8 | 22.255^**^ |
|  |  | -0.440 | -3.119 | 2.240 | .005 | <.001 | 54.471^***^ | -5.833 | 76.19 | 76.19 | <0.01 |  | 80.656^***^ | 10 | 27.215^**^ |
| REL-BU | 19 | -0.266^***^ | -0.361 | -0.172 | .009 | .020 | 315.646^***^ | -8.240 | 93.77 | 28.61 | 65.16 |  | ‒ | ‒ | ‒ |
|  |  | -0.026 | -0.719 | 0.666 | .012 | .006 | 111.204^***^ | .030 | 86.90 | 58.82 | 28.08 |  | 9.516 | 7 | 5.730 |
|  |  | 0.170 | -0.701 | 1.041 | .013 | <.001 | 89.778^***^ | 2.137 | 83.46 | 83.46 | <0.01 |  | 22.424^*^ | 10 | 9.623 |
| REL-WE | 41 | 0.398^***^ | 0.328 | 0.468 | .005 | .020 | 382.900^***^ | -40.663 | 92.62 | 19.02 | 73.60 |  | ‒ | ‒ | ‒ |
|  |  | 0.103 | -0.280 | 0.486 | .005 | .008 | 212.817^***^ | -40.499 | 85.26 | 31.88 | 53.38 |  | 18.992^**^ | 7 | 13.835 |
|  |  | 0.015 | -0.398 | 0.427 | .005 | .006 | 198.943^***^ | -35.907 | 83.74 | 36.57 | 47.17 |  | 22.456^*^ | 10 | 15.244 |
| REL-WB^a^ | 8 | 0.403^***^ | 0.357 | 0.450 | .003 | <.001 | 22.584^**^ | -14.024 | 66.92 | 66.92 | <0.01 |  | ‒ | ‒ | ‒ |
| NSUP-IJ^a^ | 6 | 0.155^*^ | 0.005 | 0.306 | .016 | .016 | 68.184^***^ | 3.066 | 93.38 | 46.69 | 46.69 |  | ‒ | ‒ | ‒ |
|  |  | -0.470 | -1.125 | 0.186 | <.001 | <.001 | <0.001 | -9.014 | - | - | - |  | 68.184^***^ | 5 | 22.080^***^ |
| NSUP-ID^a^ | 6 | 0.295^***^ | 0.175 | 0.416 | .010 | .010 | 42.576^***^ | .514 | 89.61 | 44.81 | 44.81 |  | ‒ | ‒ | ‒ |
|  |  | -0.895^**^ | -1.550 | -0.240 | <.001 | <.001 | <0.001 | -9.014 | - | - | - |  | 42.576^***^ | 5 | 19.528^**^ |
| NSUP-JP | 18 | 0.234^***^ | 0.156 | 0.312 | .009 | .012 | 133.303^***^ | -9.726 | 89.32 | 39.29 | 50.03 |  | ‒ | ‒ | ‒ |
|  |  | 0.252 | -0.514 | 1.018 | .010 | .004 | 99.208^***^ | -2.651 | 89.96 | 63.82 | 26.14 |  | 7.401 | 6 | ‒ |
|  |  | 0.302 | -0.456 | 1.060 | .009 | .003 | 89.767^***^ | -0.641 | 90.90 | 66.77 | 24.13 |  | 10.122 | 8 | ‒ |
| NSUP-JS | 20 | 0.388^***^ | 0.307 | 0.469 | .030 | <.001 | 165.336^***^ | -4.731 | 90.25 | 90.25 | <0.01 |  | ‒ | ‒ | ‒ |
|  |  | 0.386 | -0.103 | 0.876 | .013 | <.001 | 82.103^***^ | -4.261 | 76.81 | 76.81 | <0.01 |  | 19.377^**^ | 7 | 13.529 |
|  |  | 0.501 | -0.023 | 1.025 | .011 | <.001 | 71.531^***^ | -0.020 | 72.18 | 72.18 | <0.01 |  | 23.297^**^ | 10 | 15.289 |
| NSUP-TU | 23 | -0.329^***^ | -0.410 | -0.247 | .033 | .002 | 257.653^***^ | -3.273 | 92.62 | 87.88 | 4.74 |  | ‒ | ‒ | ‒ |
|  |  | -0.315 | -0.842 | 0.212 | .028 | <.001 | 194.752^***^ | 7.090 | 94.19 | 93.07 | 1.12 |  | 3.171 | 7 | ‒ |
|  |  | -0.309 | -0.950 | 0.333 | .023 | <.001 | 159.096^***^ | 8.630 | 94.24 | 94.24 | <0.01 |  | 8.778 | 10 | ‒ |
| NSUP-BU | 19 | -0.333^***^ | -0.399 | -0.267 | <.001 | .010 | 88.714^***^ | -29.963 | 76.89 | 1.04 | 75.85 |  | ‒ | ‒ | ‒ |
|  |  | 0.338 | -0.086 | 0.761 | <.001 | .001 | 24.842^**^ | -28.209 | 29.03 | 2.76 | 26.27 |  | 28.802^**^ | 7 | 12.246 |
|  |  | 8.833 | -1.177 | 18.843 | <.001 | <.001 | 7.786 | -37.961 | 0.00 | <0.01 | <0.01 |  | 80.928^***^ | 10 | 27.998^**^ |
| NSUP-WE | 25 | 0.387^***^ | 0.312 | 0.462 | .004 | .013 | 274.017^***^ | -28.869 | 88.68 | 20.87 | 67.81 |  | ‒ | ‒ | ‒ |
|  |  | 0.609 | -0.032 | 1.250 | .004 | .007 | 143.919^***^ | -21.962 | 82.47 | 33.02 | 49.45 |  | 6.743 | 6 | 5.093 |
|  |  | 0.655^*^ | 0.095 | 1.215 | .005 | <.001 | 73.118^***^ | -27.569 | 66.10 | 66.10 | <0.01 |  | 49.618^***^ | 9 | 16.701 |
| NSUP-WB^a^ | 15 | 0.434^***^ | 0.392 | 0.477 | .003 | <.001 | 27.880^*^ | -25.697 | 48.95 | 48.95 | <0.01 |  | ‒ | ‒ | ‒ |
|  |  | -1.567 | -4.488 | 1.355 | .002 | <.001 | 22.737^**^ | -16.581 | 29.62 | 29.62 | <0.01 |  | 3.014 | 6 | 2.884 |
|  |  | -2.284 | -5.391 | 0.823 | .002 | <.001 | 20.800^**^ | -15.928 | 25.62 | 25.62 | <0.01 |  | 4.549 | 7 | 4.231 |
| IG-ID^a^ | 3 | 0.517^***^ | 0.473 | 0.562 | <.001 | <.001 | 1.495 | -5.533 | 0.00 | <0.01 | <0.01 |  | ‒ | ‒ | ‒ |
| IG-WE^a^ | 3 | 0.290^***^ | 0.235 | 0.345 | .002 | <.001 | 9.518^**^ | -5.583 | 68.48 | 68.48 | <0.01 |  | ‒ | ‒ | ‒ |
| IJ-ID | 19 | 0.319^***^ | 0.222 | 0.415 | .005 | .027 | 281.244^***^ | -8.143 | 92.65 | 13.98 | 78.67 |  | ‒ | ‒ | ‒ |
|  |  | 0.088 | -0.757 | 0.933 | .005 | .019 | 189.481^***^ | 1.583 | 89.58 | 18.93 | 70.65 |  | 4.972 | 7 | 4.275 |
|  |  | -0.538 | -1.237 | 0.161 | .011 | <.001 | 92.471^***^ | -0.707 | 78.99 | 78.99 | <0.01 |  | 42.342^***^ | 10 | 12.565 |
| IJ-JP^a^ | 9 | 0.062 | -0.078 | 0.201 | <.001 | .023 | 62.867^***^ | -7.748 | 87.66 | <0.01 | 87.66 |  | ‒ | ‒ | ‒ |
|  |  | -1.754^***^ | -2.629 | -0.880 | <.001 | <.001 | 0.519 | -15.963 | 0.00 | <0.01 | <0.01 |  | 62.348^***^ | 6 | 20.215^**^ |
| IJ-JS^a^ | 4 | 0.138 | -0.056 | 0.332 | .018 | .018 | 46.129^***^ | 4.380 | 91.60 | 45.80 | 45.80 |  | ‒ | ‒ | ‒ |
|  |  | 0.035 | -0.169 | 0.239 | <.001 | <.001 | 0.00 | -3.440 | - | - | - |  | 46.129^***^ | 3 | 13.820^*^ |
| IJ-TU^a^ | 6 | -0.015 | -0.123 | 0.083 | .002 | .004 | 19.237^**^ | -5.743 | 68.11 | 25.05 | 43.06 |  | ‒ | ‒ | ‒ |
| IJ-BU^a^ | 5 | -0.148^***^ | -0.204 | -0.092 | .003 | <.001 | 12.833^*^ | -7.369 | 60.72 | 60.72 | <0.01 |  | ‒ | ‒ | ‒ |
|  |  | -0.145^***^ | -0.210 | -0.079 | <.001 | <.001 | 6.293^*^ | -6.811 | 19.55 | 19.55 | <0.01 |  | 5.152’ | 2 | 3.442 |
| IJ-WE^a^ | 10 | 0.048 | -0.091 | 0.186 | .002 | .026 | 192.788^***^ | -6.513 | 93.66 | 8.12 | 85.54 |  | ‒ | ‒ | ‒ |
|  |  | 0.345^***^ | 0.291 | 0.399 | <.001 | <.001 | 10.587^*^ | -16.999 | 21.24 | 21.24 | <0.01 |  | 145.986^***^ | 5 | 20.486^**^ |
| IJ-WB^a^ | 7 | 0.077 | -0.044 | 0.198 | <.001 | .017 | 100.048^***^ | -5.779 | 90.17 | <0.01 | 90.17 |  | ‒ | ‒ | ‒ |
|  |  | -0.370^***^ | -0.475 | -0.265 | <.001 | <.001 | 1.697 | -15.009 | 0.00 | <0.01 | <0.01 |  | 98.350^***^ | 4 | 17.230^**^ |
| ID-JP | 11 | 0.187^***^ | 0.077 | 0.296 | .003 | .015 | 88.253^***^ | -9.617 | 85.11 | 13.33 | 71.78 |  | ‒ | ‒ | ‒ |
|  |  | -0.453 | -2.317 | 1.412 | .001 | .010 | 54.881^***^ | -2.221 | 79.27 | 8.81 | 70.46 |  | 5.479 | 6 | 4.604 |
|  |  | 1.492^**^ | 0.463 | 2.541 | <.001 | <.001 | 5.917 | -16.883 | 0.00 | <0.01 | <0.01 |  | 82.336^***^ | 7 | 21.266^**^ |
| ID-JS^a^ | 5 | 0.488^***^ | 0.338 | 0.638 | .013 | .013 | 44.968^***^ | 2.545 | 88.70 | 44.35 | 44.35 |  | ‒ | ‒ | ‒ |
|  |  | 1.504^***^ | 0.889 | 2.119 | <.001 | <.001 | 0.00 | -5.237 | - | - | - |  | 44.968^***^ | 4 | 15.782^**^ |
| ID-TU^a^ | 8 | -0.255^**^ | -0.449 | -0.061 | .021 | .024 | 130.375^***^ | 3.063 | 93.42 | 42.97 | 50.45 |  | ‒ | ‒ | ‒ |
| ID-BU^a^ | 6 | -0.320^***^ | -0.505 | -0.136 | .007 | .029 | 144.235^***^ | 1.605 | 96.33 | 18.50 | 77.83 |  | ‒ | ‒ | ‒ |
|  |  | -0.306^***^ | -0.376 | -0.236 | .001 | <.001 | 9.886^**^ | -6.770 | 30.25 | 30.25 | <0.01 |  | 90.841^***^ | 3 | 14.375^**^ |
| ID-WE | 15 | 0.471^***^ | 0.380 | 0.562 | .001 | .016 | 170.608^***^ | -19.313 | 91.97 | 6.83 | 85.14 |  | ‒ | ‒ | ‒ |
|  |  | 1.103^***^ | 0.499 | 1.707 | .001 | .007 | 65.927^***^ | -13.843 | 85.78 | 13.40 | 72.38 |  | 9.020 | 6 | 6.530 |
|  |  | 1.150^***^ | 0.669 | 1.630 | .001 | .002 | 37.485^***^ | -17.684 | 72.55 | 27.27 | 45.28 |  | 27.975^***^ | 7 | 12.371 |
| ID-WB^a^ | 8 | 0.475^***^ | 0.375 | 0.574 | <.001 | .013 | 52.655^***^ | -7.255 | 87.05 | <0.01 | 87.05 |  | ‒ | ‒ | ‒ |
|  |  | -4.878^**^ | -7.810 | -1.947 | <.001 | <.001 | 2.089 | -16.415 | 0.00 | <0.01 | <0.01 |  | 50.566^***^ | 5 | 19.160^**^ |
| JP-JS^a^ | 7 | 0.420^***^ | 0.331 | 0.510 | .005 | .005 | 18.592^**^ | -3.176 | 64.93 | 32.46 | 32.46 |  | ‒ | ‒ | ‒ |
|  |  | 0.302^*^ | 0.027 | 0.577 | .001 | .001 | 9.526^**^ | 1.518 | 17.98 | 8.99 | 8.99 |  | 7.597 | 5 | 5.307 |
| JP-TU | 18 | -0.170^***^ | -0.238 | -0.102 | .018 | <.001 | 99.119^***^ | -10.231 | 86.48 | 86.48 | <0.01 |  | ‒ | ‒ | ‒ |
|  |  | -0.162 | -0.609 | 0.285 | .013 | <.001 | 73.236^***^ | .798 | 90.04 | 90.04 | <0.01 |  | 4.887 | 7 | ‒ |
|  |  | -0.096 | -0.759 | 0.566 | .012 | <.001 | 72.356^***^ | 3.252 | 78.69 | 78.69 | <0.01 |  | 7.453 | 10 | 6.517 |
| JP-BU^a^ | 6 | -0.274^***^ | -0.416 | -0.132 | .001 | .022 | 63.848^***^ | -0.053 | 86.85 | 2.72 | 84.12 |  | ‒ | ‒ | ‒ |
|  |  | 0.092 | -0.152 | 0.336 | <.001 | <.001 | 1.583 | -6.819 | 0.00 | <0.01 | <0.01 |  | 62.265^***^ | 4 | 14.766^**^ |
| JP-WE | 17 | 0.437^***^ | 0.320 | 0.554 | .003 | .030 | 306.962^***^ | -10.847 | 94.75 | 9.29 | 85.46 |  | ‒ | ‒ | ‒ |
|  |  | 0.117 | -1.393 | 1.626 | .003 | .024 | 274.757^***^ | -1.208 | 92.72 | 10.17 | 82.55 |  | 2.597 | 6 | 2.362 |
|  |  | -1.269 | -3.083 | 0.545 | .003 | .015 | 98.808^***^ | 1.392 | 90.25 | 15.87 | 74.39 |  | 8.173 | 9 | 5.761 |
| JP-WB^a^ | 3 | 0.343^***^ | 0.183 | 0.503 | .013 | <.001 | 9.621^**^ | 2.833 | 66.76 | 66.76 | <0.01 |  | ‒ | ‒ | ‒ |
| JS-TU | 24 | -0.415^***^ | -0.507 | -0.323 | .046 | .002 | 358.397^***^ | 3.364 | 94.31 | 90.40 | 3.91 |  | ‒ | ‒ | ‒ |
|  |  | 0.325 | -0.272 | 0.923 | .032 | <.001 | 222.218^***^ | 10.313 | 91.16 | 91.16 | <0.01 |  | 11.062 | 8 | 9.051 |
|  |  | 0.229 | -0.369 | 0.826 | .027 | <.001 | 187.038^***^ | 13.137 | 89.45 | 89.45 | <0.01 |  | 16.131 | 11 | 12.227 |
| JS-BU^a^ | 10 | -0.463^***^ | -0.523 | -0.404 | .006 | <.001 | 62.893^***^ | -13.523 | 76.68 | 76.68 | <0.01 |  | ‒ | ‒ | ‒ |
| JS-WE | 10 | 0.589^***^ | 0.528 | 0.650 | .007 | <.001 | 49.172^***^ | -12.234 | 80.02 | 80.02 | <0.01 |  | ‒ | ‒ | ‒ |
|  |  | 0.446^**^ | 0.130 | 0.761 | .002 | <.001 | 19.122^**^ | -7.772 | 41.45 | 41.45 | <0.01 |  | 9.186 | 5 | 5.537 |
|  |  | 0.828 | -1.838 | 3.494 | <.001 | <.001 | 11.789^**^ | -10.245 | 0.00 | <0.01 | <0.01 |  | 37.383^***^ | 7 | 12.011 |
| JS-WB^a^ | 9 | 0.528^***^ | 0.411 | 0.645 | .029 | <.001 | 116.550^***^ | .779 | 94.72 | 94.72 | <0.01 |  | ‒ | ‒ | ‒ |
| TU-BU | 16 | 0.447^***^ | 0.355 | 0.538 | .003 | .021 | 137.002^***^ | -10.303 | 94.94 | 10.47 | 84.48 |  | ‒ | ‒ | ‒ |
|  |  | 0.890 | 0.140 | 1.639 | .003 | .010 | 84.781^***^ | -5.546 | 90.98 | 19.37 | 71.61 |  | 8.964 | 6 | 7.243 |
|  |  | 1.267^***^ | 0.820 | 1.714 | .001 | <.001 | 31.904^***^ | -27.369 | 47.97 | 47.97 | <0.01 |  | 74.806^***^ | 9 | 35.066^***^ |
| TU-WE | 12 | -0.207^*^ | -0.381 | -0.032 | .090 | <.001 | 218.902^***^ | 11.987 | 96.31 | 96.31 | <0.01 |  | ‒ | ‒ | ‒ |
|  |  | -1.213^**^ | -2.036 | -0.391 | .020 | <.001 | 67.360 | 9.602 | 81.22 | 81.22 | <0.01 |  | 33.217^***^ | 7 | 16.385^*^ |
|  |  | -1.261^***^ | -1.985 | -0.537 | .014 | <.001 | 53.446^***^ | 7.906 | 76.55 | 76.55 | <0.01 |  | 47.348^***^ | 8 | 20.081^*^ |
| TU-WB^a^ | 4 | -0.462^***^ | -0.628 | -0.295 | .026 | <.001 | 35.517^***^ | 3.261 | 90.66 | 90.66 | <0.01 |  | ‒ | ‒ | ‒ |
| BU-WB^a^ | 3 | -0.646^***^ | -0.739 | -0.553 | <.001 | <.001 | 0.422 | -4.991 | 0.00 | <0.01 | <0.01 |  | ‒ | ‒ | ‒ |
| BU-WE | 31 | -0.399^***^ | -0.508 | -0.290 | .008 | .045 | 617.149^***^ | -14.396 | 98.21 | 14.07 | 84.15 |  | ‒ | ‒ | ‒ |
|  |  | -0.439 | -1.250 | 0.372 | .004 | .047 | 450.843^***^ | -10.626 | 98.08 | 7.41 | 90.67 |  | 10.464 | 6 | 8.230 |
|  |  | 0.475 | -0.379 | 1.329 | .004 | .023 | 339.169^***^ | -14.692 | 96.63 | 14.23 | 82.40 |  | 24.135^**^ | 9 | 18.296^*^ |
| WB-WE^a^ | 3 | 0.463^***^ | 0.418 | 0.507 | <.001 | <.001 | 0.178 | -7.445 | 0.00 | <0.01 | <0.01 |  | ‒ | ‒ | ‒ |
|  |  | 0.450^***^ | 0.303 | 0.597 | <.001 | <.001 | <0.001 | -3.622 | ‒ | ‒ | ‒ |  | .178 | 2 | 0.178 |

*Note.* Values printed on top line are results of the standard multilevel meta-analytic model, values printed on second line are results of the meta-regression model in which the study effect size was regressed on the set of covariates (age, sex, study quality, study design), and values printed on the third line are results of the meta-regression model in which the study effect size was regressed on the covariates and moderator variables. Associations for the following relations were omitted due to small samples: AUT-IG, AUT-ID, AUT-IJ, COM-IG, COM-IJ, REL-IG, NSUP-IG, IG-IJ, IG-JP, IG-JS, IG-BU, IG-TU, and IG-WB. ^a^There were insufficient studies available in subgroups to compute meta-regression analyses for covariate and moderator variables for this correlation. ^b^Coefficients reported on the second and third lines for each effect size are the intercept from the respective meta-regression model and are interpreted as the averaged effect size estimate when the moderators are at their reference value. ^c^Degrees of freedom = 3 in each analysis. *k* = Number of studies; *r* = Averaged sample-weighted correlation; UL = Upper limit of the 95% confidence interval of *r*; LL = Lower limit of the 95% confidence interval of *r*; σ^2^ = Variance component from the multilevel model, an estimate of the “true” variability in the effect size; *Q* = Cochrane’s *Q* statistic; AIC = Akaike’s Information Criterion; *I*^2^ = Higgins and Thompson’s ([2002](#_ENREF_83)) *I*^2^ statistic; var σ^2^ = Percentage of overall variance attributable to within and between study variance components; LRT = Likelihood ratio test testing for differences across models including and excluding moderators; IM = Global or aggregated autonomous or intrinsic motivation; EM = Global or aggregated extrinsic or controlled motivation or external regulation; AUT = Autonomy need support; COM = Competence need support; REL = Relatedness need support; NSUP = Need Support; IG = Integrated regulation; ID = Identified regulation; IJ = Introjected regulation; JP = Job performance; JS = Job satisfaction; TU = Turnover; BU = Burnout; WE = Work engagement; WB = Well-being.

^*^*p* < .05 ^**^*p* < .01 ^***^*p* < .001

Supplement K

**Table K1**

*Fit Statistics for Multilevel Multivariate Meta-Analytic Models for the Full Sample and Moderator Groups*

| Model | *N* | *k* | χ^2^ | Model fit | |  | Fit indexes | | | | | |  | Model selection criteria | |
| --- | --- | --- | --- | --- | --- | --- | --- | --- | --- | --- | --- | --- | --- | --- | --- |
|  |  |  |  | df | *p* |  | CFI | TLI | SRMR | RMSEA | RMSEA  95% CI | |  | AIC | BIC |
|  |  |  |  |  |  |  |  |  |  |  | LL | UL |  |  |  |
| Full sample MASEM model | 93552 | 185 | 136.690 | 28 | <.001 |  | 0.981 | 0.937 | .066 | .006 | .006 | .008 |  | 80.690 | -183.806 |
|  | 93552 | 185 | 96.271 | 28 | <.001 |  | 0.989 | 0.965 | .055 | .005 | .004 | .006 |  | 40.271 | -224.224 |
| Truncated MASEM model | 93552 | 185 | 105.682 | 4 | <.001 |  | 0.973 | 0.900 | .063 | .017 | .014 | .019 |  | 97.682 | 59.897 |
|  | 93552 | 185 | 68.909 | 4 | <.001 |  | 0.985 | 0.945 | .057 | .013 | .011 | .016 |  | 60.909 | 23.124 |
| Moderator: Country GDP |  |  |  |  |  |  |  |  |  |  |  |  |  |  |  |
| Top 10 GDP | 33053 | 89 | 69.400 | 4 | <.001 |  | 0.967 | 0.877 | .066 | .022 | .018 | .027 |  | 61.400 | 27.776 |
|  | 33053 | 89 | 54.693 | 4 | <.001 |  | 0.977 | 0.913 | .062 | .020 | .015 | .024 |  | 46.693 | 13.070 |
| < Top 10 GDP | 60756 | 96 | 27.219 | 4 | <.001 |  | 0.986 | 0.949 | .054 | .010 | .007 | .013 |  | 19.219 | -16.839 |
|  | 60756 | 96 | 27.690 | 4 | <.001 |  | 0.986 | 0.947 | .053 | .009 | .007 | .014 |  | 19.690 | -16.369 |
| Moderator: Work type |  |  |  |  |  |  |  |  |  |  |  |  |  |  |  |
| Public service | 38452 | 61 | 38.353 | 4 | <.001 |  | 0.979 | 0.921 | .058 | .015 | .011 | .019 |  | 30.353 | -3.876 |
|  | 38452 | 61 | 49.946 | 4 | <.001 |  | 0.970 | 0.886 | .068 | .017 | .013 | .022 |  | 41.946 | 7.717 |
| For-profit | 45966 | 103 | 70.827 | 4 | <.001 |  | 0.967 | 0.878 | .087 | .019 | .015 | .023 |  | 62.827 | 27.885 |
|  | 45966 | 103 | 37.681 | 4 | <.001 |  | 0.988 | 0.954 | .079 | .014 | .010 | .018 |  | 29.681 | -5.262 |
| Excl. mixed work samples^a^ | 84236 | 163 | 97.931 | 4 | <.001 |  | 0.973 | 0.898 | .062 | .017 | .014 | .020 |  | 89.931 | 52.566 |
|  | 84236 | 163 | 35.419 | 4 | <.001 |  | 0.994 | 0.956 | .046 | .010 | .007 | .013 |  | 27.419 | -9.947 |
| Moderator: Employee type |  |  |  |  |  |  |  |  |  |  |  |  |  |  |  |
| Corporate employee | 35187 | 66 | 18.643 | 4 | <.001 |  | 0.985 | 0.944 | .080 | .010 | .006 | .015 |  | 10.643 | -23.231 |
|  | 35187 | 66 | 11.179 | 4 | .025 |  | 0.995 | 0.980 | .063 | .007 | .002 | .012 |  | 3.179 | -30.695 |
| Healthcare worker | 11054 | 29 | 17.237 | 4 | .002 |  | 0.987 | 0.951 | .044 | .017 | .010 | .026 |  | 9.237 | -20.006 |
|  | 11054 | 29 | 35.335 | 4 | <.001 |  | 0.958 | 0.842 | .089 | .027 | .019 | .035 |  | 27.335 | -1.907 |
| Teacher | 10908 | 26 | 25.604 | 4 | <.001 |  | 0.983 | 0.938 | .057 | .022 | .015 | .031 |  | 17.604 | -11.585 |
|  | 10908 | 26 | 25.218 | 4 | <.001 |  | 0.982 | 0.932 | .058 | .022 | .014 | .031 |  | 17.218 | -11.971 |
| Other/mixed | 39631 | 26 | 54.991 | 4 | <.001 |  | 0.958 | 0.842 | .085 | .018 | .014 | .022 |  | 46.991 | 12.641 |
|  | 39631 | 26 | 75.929 | 4 | <.001 |  | 0.934 | 0.753 | .095 | .021 | .017 | .026 |  | 67.929 | 33.579 |
| Moderator: Cultural orientation |  |  |  |  |  |  |  |  |  |  |  |  |  |  |  |
| Individualist | 69335 | 126 | 72.897 | 4 | <.001 |  | 0.980 | 0.923 | .057 | .016 | .013 | .019 |  | 64.897 | 28.310 |
|  | 69335 | 126 | 40.552 | 4 | <.001 |  | 0.991 | 0.967 | .050 | .012 | .008 | .015 |  | 32.552 | -4.035 |
| Collectivist | 22290 | 54 | 31.777 | 4 | <.001 |  | 0.961 | 0.853 | .077 | .018 | .012 | .024 |  | 23.777 | -8.271 |
|  | 22290 | 54 | 29.660 | 4 | <.001 |  | 0.965 | 0.868 | .076 | .017 | .012 | .023 |  | 21.660 | -10.388 |
| Moderator: Proximity of leader autonomy support |  |  |  |  |  |  |  |  |  |  |  |  |  |  |  |
| Proximal | 16575 | 36 | 55.901 | 4 | <.001 |  | 0.965 | 0.869 | .061 | .028 | .022 | .035 |  | 47.901 | 16.995 |
|  | 16757 | 36 | 67.269 | 4 | <.001 |  | 0.945 | 0.793 | .070 | .031 | .025 | .037 |  | 59.269 | 28.362 |
| Distal or indeterminate | 4451 | 13 | 15.028 | 4 | .005 |  | 0.975 | 0.906 | .052 | .025 | .012 | .039 |  | 7.028 | -18.576 |
|  | 4451 | 13 | 13.578 | 4 | .009 |  | 0.976 | 0.910 | .050 | .023 | .010 | .037 |  | 5.578 | -20.026 |

*Note*. Values printed on upper line are for models unadjusted for covariates, values printed on lower line are for models adjusted for the following covariates: age, sex, and study quality. ^a^Sensitivity analysis testing whether conclusions on parameter estimated should be altered when excluding studies with samples of employed in public service and for-profit organizations. *N* = Total sample size across studies contributing to model; *k* = Number of studies contributing to estimated model; χ^2^ = Model goodness-of-fit chi-square relative to independence (totally free) model; df = Degrees of freedom; CFI = Comparative fit index; TLI = Tucker-Lewis index; SRMR = Standardised root mean square residual; RMSEA = Root mean square error of approximation; RMSEA 95% CI = 95% confidence intervals of RMSEA; LL = Lower limit of the RMSEA 95% confidence interval; UL = Upper limit of the RMSEA 95% confidence interval; AIC = Akaike’s information criterion; BIC = Bayesian information criterion; MASEM = Meta-analytic structural equation modelling.

**Table K2**

*Variability Estimates and Heterogeneity Statistics for Multilevel Multivariate Meta-Analytic Models for the Full Sample and Moderator Groups*

| Model^a^ | L2 σ^2^ | L3 σ^2^ | *Q*^a^ | df | *I*^2^ | L2 var | L3 var |
| --- | --- | --- | --- | --- | --- | --- | --- |
|  |  |  |  |  |  |  |  |
| Full model | .009 | .021 | 23022.971^***^ | 1688 | 93.83 | 27.85 | 65.98 |
|  | .009 | .021 | 23052.120^***^ | 1688 | 93.88 | 28.14 | 65.73 |
| Truncated model | .009 | .025 | 22591.200^***^ | 1521 | 94.51 | 24.52 | 69.99 |
|  | .009 | .025 | 22745.964^***^ | 1521 | 94.57 | 25.15 | 69.42 |
| Moderator analyses |  |  |  |  |  |  |  |
| Moderator: Country GDP | . |  |  |  |  |  |  |
| Top 10 GDP | .009 | .027 | 10951.091^***^ | 834 | 93.21 | 24.02 | 69.18 |
|  | .009 | .027 | 11168.337^***^ | 834 | 93.17 | 23.40 | 69.77 |
| <Top 10 GDP | .010 | .021 | 10816.004^***^ | 649 | 95.44 | 29.77 | 65.68 |
|  | .010 | .021 | 10854.105^***^ | 649 | 95.54 | 31.35 | 64.18 |
| Moderator: Work type |  |  |  |  |  |  |  |
| Public service | .002 | .026 | 7905.161^***^ | 460 | 94.28 | 5.48 | 88.81 |
|  | .003 | .026 | 8225.519^***^ | 460 | 94.52 | 10.18 | 84.34 |
| For-profit | .012 | .023 | 12417.579^***^ | 907 | 94.56 | 32.49 | 62.07 |
|  | .012 | .023 | 12425.775^***^ | 907 | 94.59 | 32.90 | 61.69 |
| Excluding mixed work samples^b^ | .009 | .025 | 21065.215^***^ | 1382 | 94.69 | 24.18 | 70.51 |
|  | .010 | .025 | 21607.268^***^ | 1382 | 94.83 | 26.03 | 68.80 |
| Moderator: Employee type |  |  |  |  |  |  |  |
| Corporate employee | .009 | .027 | 7185.874^***^ | 481 | 93.60 | 22.80 | 70.80 |
|  | .011 | .027 | 7416.021^***^ | 481 | 93.92 | 27.36 | 66.56 |
| Healthcare worker | .003 | .019 | 3000.443^***^ | 271 | 90.01 | 11.93 | 78.08 |
|  | .012 | .019 | 3543.823^***^ | 271 | 92.62 | 36.65 | 55.98 |
| Teacher | .001 | .021 | 2264.891^***^ | 239 | 90.18 | 3.46 | 86.71 |
|  | .002 | .022 | 2440.119^***^ | 239 | 91.01 | 8.23 | 82.78 |
| Other | .013 | .022 | 8400.306^***^ | 485 | 96.06 | 36.34 | 59.71 |
|  | .014 | .022 | 8439.213^***^ | 485 | 96.11 | 37.07 | 59.04 |
| Moderator: Cultural orientation |  |  |  |  |  |  |  |
| Individualist | .005 | .023 | 15848.131^***^ | 1085 | 93.38 | 16.15 | 77.23 |
|  | .005 | .023 | 15686.903^***^ | 1085 | 93.42 | 16.34 | 77.08 |
| Collectivist | .009 | .027 | 5086.666^***^ | 377 | 94.70 | 23.62 | 71.09 |
|  | .010 | .026 | 5215.495^***^ | 377 | 94.78 | 25.29 | 69.50 |
| Moderator: Proximity of leader autonomy support |  |  |  |  |  |  |  |
| Proximal | .002 | .024 | 5564.119^***^ | 408 | 92.34 | 6.87 | 85.47 |
|  | .013 | .023 | 6193.371^***^ | 408 | 94.34 | 33.29 | 61.05 |
| Distal or indeterminate | .007 | .021 | 1917.327^***^ | 172 | 91.72 | 22.04 | 69.68 |
|  | .018 | .021 | 2551.892^***^ | 172 | 93.90 | 42.80 | 51.09 |

*Note*. ^a^Values printed on the upper line are unadjusted for covariates and values printed on the lower line are adjusted for the following covariates: age, sex, study quality, and study design. ^a^Sensitivity analysis testing whether conclusions on parameter estimated should be altered when excluding studies with samples of employed in public service and for-profit organizations. L2 = Level 2 variance component of multilevel model (variance between effect sizes within studies); L3 = Level 3 variance component of the multilevel meta-analytic model (variance between studies); σ^2^ = Estimate of ‘true’ variability in the effect; *Q* = Cochran’s *Q* test; df = Degrees of freedom for *Q*; *I*^2^ = Higgins and Thompson’s ([2002](#_ENREF_83)) *I*^2^ statistic; L2 var. = Percentage of total variability attributable to variability between effect sizes within studies (level 2); L3 var. = Percentage of total variability attributable to variability between studies (level 3).^***^*p* < .001 ^**^*p* < .01 ^*^*p* < .05

**Table K3**

*Results of Multilevel Multivariate Meta-Analysis of Relations Between Constructs from Self-Determination Theory and Workplace Outcomes for the Full Model Unadjusted and Adjusted for Covariates*

| Effect | Unadjusted model | | | |  | Covariate adjusted model^a^ | | | |
| --- | --- | --- | --- | --- | --- | --- | --- | --- | --- |
|  | *r* | SE | 95% CI | |  | *r* | SE | 95% CI | |
|  |  |  | LL | UL |  |  |  | LL | UL |
| IM-EM | .122^***^ | .021 | .081 | .162 |  | .255^***^ | .021 | .215 | .296 |
| IM-AU | .401^***^ | .027 | .347 | .455 |  | .534^***^ | .028 | .480 | .588 |
| IM-COM | .397^***^ | .040 | .320 | .475 |  | .531^***^ | .040 | .454 | .609 |
| IM-REL | .363^***^ | .037 | .292 | .435 |  | .494^***^ | .037 | .422 | .566 |
| IM-NSUP | .377^***^ | .031 | .317 | .438 |  | .511^***^ | .031 | .450 | .572 |
| IM-IJ | .237^***^ | .037 | .165 | .308 |  | .367^***^ | .037 | .295 | .439 |
| IM-ID | .513^***^ | .032 | .450 | .576 |  | .644^***^ | .032 | .580 | .707 |
| IM-JP | .277^***^ | .025 | .229 | .326 |  | .405^***^ | .025 | .356 | .453 |
| IM-JS | .395^***^ | .031 | .335 | .455 |  | .531^***^ | .031 | .470 | .591 |
| IM-TU | -.280^***^ | .023 | -.325 | -.235 |  | -.140^***^ | .023 | -.185 | -.094 |
| IM-BU | -.301^***^ | .029 | -.357 | -.245 |  | -.165^***^ | .029 | -.221 | -.109 |
| IM-WE | .458^***^ | .022 | .414 | .501 |  | .591^***^ | .022 | .547 | .635 |
| IM-WB | .479^***^ | .048 | .386 | .572 |  | .612^***^ | .048 | .518 | .705 |
| EM-AU | -.028 | .036 | -.100 | .043 |  | .107^**^ | .037 | .035 | .178 |
| EM-COM | .021 | .063 | -.103 | .144 |  | .157^*^ | .063 | .033 | .280 |
| EM-REL | -.047 | .053 | -.151 | .056 |  | .087 | .053 | -.017 | .190 |
| EM-NSUP | .0682 | .045 | -.019 | .155 |  | .204^***^ | .045 | .117 | .291 |
| EM-IJ | .310^***^ | .038 | .235 | .385 |  | .440^***^ | .038 | .365 | .515 |
| EM-ID | .162^***^ | .036 | .091 | .233 |  | .293^***^ | .036 | .221 | .364 |
| EM-JP | .037 | .037 | -.036 | .109 |  | .165^***^ | .037 | .093 | .238 |
| EM-JS | .021 | .043 | -.062 | .105 |  | .155^***^ | .043 | .072 | .239 |
| EM-TU | .035 | .036 | -.035 | .105 |  | .173^***^ | .036 | .103 | .243 |
| EM-BU | .129^***^ | .036 | .059 | .199 |  | .268^***^ | .036 | .198 | .338 |
| EM-WE | .085^**^ | .028 | .029 | .140 |  | .219^***^ | .028 | .163 | .274 |
| EM-WB | -.027 | .056 | -.135 | .082 |  | .107 | .056 | -.002 | .216 |
| AU-COM | .515^***^ | .027 | .463 | .567 |  | .647^***^ | .027 | .595 | .699 |
| AU-REL | .482^***^ | .024 | .434 | .529 |  | .615^***^ | .024 | .567 | .662 |
| AU-NSUP | .498^***^ | .033 | .434 | .562 |  | .634^***^ | .033 | .570 | .698 |
| AU-IJ | .022 | .074 | -.122 | .166 |  | .155^*^ | .074 | .011 | .300 |
| AU-ID | .344^***^ | .058 | .231 | .457 |  | .477^***^ | .058 | .364 | .590 |
| AU-JP | .248^***^ | .038 | .174 | .323 |  | .377^***^ | .038 | .302 | .452 |
| AU-JS | .413^***^ | .027 | .360 | .467 |  | .546^***^ | .027 | .493 | .600 |
| AU-TU | -.370^***^ | .030 | -.427 | -.312 |  | -.236^***^ | .030 | -.293 | -.178 |
| AU-BU | -.393^***^ | .030 | -.452 | -.334 |  | -.256^***^ | .030 | -.316 | -.197 |
| AU-WE | .404^***^ | .022 | .360 | .448 |  | .536^***^ | .022 | .493 | .580 |
| AU-WB | .447^***^ | .040 | .369 | .524 |  | .581^***^ | .040 | .503 | .659 |
| COM-REL | .458^***^ | .028 | .404 | .513 |  | .591^***^ | .028 | .536 | .646 |
| COM-NSUP | .448^***^ | .049 | .352 | .544 |  | .584^***^ | .049 | .489. | .680 |
| COM-IJ | .004 | .153 | -.295 | .303 |  | .138 | .153 | -.161 | .438 |
| COM-ID | .447^***^ | .090 | .271 | .623 |  | .579^***^ | .090 | .402 | .755 |
| COM-JP | .364^***^ | .055 | .257 | .472 |  | .494^***^ | .055 | .386 | .602 |
| COM-JS | .453^***^ | .039 | .377 | .529 |  | .587^***^ | .039 | .511 | .664 |
| COM-TU | -.317^***^ | .053 | -.420 | -.214 |  | -.190^***^ | .053 | -.293 | -.087 |
| COM-BU | -.330^***^ | .056 | -.438 | -.221 |  | -.194^***^ | .056 | -.303 | -.085 |
| COM-WE | .442^***^ | .030 | .384 | .500 |  | .575^***^ | .030 | .517 | .633 |
| COM-WB | .527^***^ | .064 | .401 | .653 |  | .659^***^ | .064 | .533 | .785 |
| REL-NSUP | .393^***^ | .038 | .319 | .468 |  | .528^***^ | .038 | .453 | .603 |
| REL-IJ | .076 | .089 | -.098 | .250 |  | .211^*^ | .089 | .037 | .385 |
| REL-ID | .323^***^ | .070 | .187 | .460 |  | .457^***^ | .070 | .320 | .593 |
| REL-JP | .221^***^ | .061 | .101 | .340 |  | .349^***^ | .061 | .229 | .469 |
| REL-JS | .406^***^ | .035 | .338 | .474 |  | .540^***^ | .035 | .471 | .608 |
| REL-TU | -.388^***^ | .041 | -.299 | -.149 |  | -.256^***^ | .041 | -.336 | -.176 |
| REL-BU | -.224^***^ | .038 | -.299 | -.149 |  | -.090^*^ | .038 | -.165 | -.015 |
| REL-WE | .376^***^ | .027 | .322 | .430 |  | .510^***^ | .027 | .456 | .563 |
| REL-WB | .441^***^ | .056 | .331 | .551 |  | .574^***^ | .056 | .463 | .684 |
| NS-IJ | .197^**^ | .064 | .071 | .323 |  | .330^***^ | .064 | .204 | .457 |
| NS-ID | .340^***^ | .064 | .214 | .466 |  | .473^***^ | .064 | .347 | .599 |
| NS-JP | .231^***^ | .043 | .146 | .316 |  | .366^***^ | .044 | .280 | .451 |
| NS-JS | .413^***^ | .039 | .338 | .489 |  | .550^***^ | .039 | .475 | .626 |
| NS-TU | -.319^***^ | .036 | -.389 | -.248 |  | -.180^***^ | .036 | -.251 | -.109 |
| NS-BU | -.295^***^ | .040 | -.372 | -.217 |  | -.158^***^ | .040 | -.236 | -.080 |
| NS-WE | .369^***^ | .035 | .300 | .438 |  | .503^***^ | .035 | .434 | .572 |
| NS-WB | .467^***^ | .045 | .379 | .555 |  | .603^***^ | .045 | .515 | .691 |
| IJ-ID | .301^***^ | .039 | .224 | .377 |  | .430^***^ | .039 | .354 | .507 |
| IJ-JP | .039 | .058 | -.076 | .153 |  | .164^**^ | .059 | .050 | .279 |
| IJ-JS | .199^*^ | .081 | .041 | .357 |  | .332^***^ | .081 | .173 | .490 |
| IJ-TU | .096 | .061 | -.023 | .215 |  | .229^***^ | .061 | .110 | .348 |
| IJ-BU | -.045 | .069 | -.181 | .090 |  | .088 | .070 | -.048 | .224 |
| IJ-WE | .118^*^ | .054 | .013 | .223 |  | .250^***^ | .054 | .145 | .355 |
| IJ-WB | .124^*^ | .060 | .007 | .240 |  | .257^***^ | .060 | .140 | .374 |
| ID-JP | .183^***^ | .050 | .085 | .280 |  | .309^***^ | .050 | .212 | .407 |
| ID-JS | .525^***^ | .072 | .384 | .666 |  | .657^***^ | .072 | .516 | .799 |
| ID-TU | -.120^*^ | .057 | -.232 | -.008 |  | .012 | .057 | -.101 | .124 |
| ID-BU | -.325^***^ | .063 | -.449 | -.201 |  | -.191^**^ | .063 | -.315 | -.067 |
| ID-WE | .474^***^ | .042 | .392 | .556 |  | .606^***^ | .042 | .524 | .688 |
| ID-WB | .516^***^ | .056 | .406 | .625 |  | .649^***^ | .056 | .539 | .759 |
| JP-JS | .415^***^ | .065 | .287 | .545 |  | .548^***^ | .066 | .419 | .676 |
| JP-TU | -.149^***^ | .037 | -.222 | -.076 |  | -.018 | .037 | -.091 | .055 |
| JP-BU | -.292^***^ | .070 | -.430 | -.154 |  | -.155^*^ | .071 | -.293 | -.017 |
| JP-WE | .393^***^ | .041 | .314 | .473 |  | .522^***^ | .041 | .442 | .602 |
| JP-WB | .290^**^ | .105 | .085 | .495 |  | .415^***^ | .105 | .209 | .620 |
| JS-TU | -.437^***^ | .035 | -.505 | -.369 |  | -.302^***^ | .035 | -.370 | -.234 |
| JS-BU | -.486^***^ | .053 | -.590 | -.383 |  | -.354 | .053 | -.457 | -.250 |
| JS-WE | .546^***^ | .052 | .444 | .648 |  | .682^***^ | .052 | .580 | .784 |
| JS-WB | .557^***^ | .054 | .451 | .662 |  | .689^***^ | .054 | .584 | .795 |
| TU-BU | .472^***^ | .042 | .390 | .553 |  | .610^***^ | .042 | .528 | .692 |
| TU-WE | -.240^***^ | .049 | -.335 | -.144 |  | -.109^*^ | .049 | -.205 | -.013 |
| TU-WB | -.461^***^ | .079 | -.616 | -.306 |  | -.328^***^ | .079 | -.483 | -.172 |
| BU-WB | -.372^***^ | .030 | -.432 | -.313 |  | -.237^***^ | .030 | -.296 | -.177 |
| BU-WE | -.630^***^ | .102 | -.831 | -.430 |  | -.491^***^ | .102 | -.691 | -.290 |
| WB-WE | .507^***^ | .089 | .332 | .681 |  | .640^***^ | .089 | .465 | .816 |

*Note*. ^a^Effect sizes adjusted for the following covariates: age, gender, study quality, and study design. *r^+^* = Zero-order correlation corrected for sampling error; 95% CI = 95% confidence interval of *r^+^*; LL = Lower limit of 95% confidence interval; UL = Upper limit of 95% confidence interval; SE = Standard error; IM = Intrinsic motivation; EM = Extrinsic motivation or external regulation; AUT = Autonomy need satisfaction; COM = Competence need satisfaction; REL = Relatedness need satisfaction; NS = Need Support; ID = Identified regulation; IJ = Introjected regulation; JP = Job performance; JS = Job satisfaction; TU = Turnover; BU = Burnout; WE = Work engagement; WB = Well-being.

^***^*p* < .001 ^**^*p* < .01 ^*^*p* < .05

Supplement L

**Table L1**

*Standardized Parameter Estimates for Direct and Indirect Effects of Self-Determination Theory Constructs on Workplace Outcomes from the Multilevel Meta-Analytic Structural Equation Model of the Full Model Unadjusted and Adjusted for Covariates*

| Effect | Unadjusted model | | |  | Model adjusted for covariates | | |
| --- | --- | --- | --- | --- | --- | --- | --- |
|  | β | 95% CI | |  | β | 95% CI | |
|  |  | LL | UL |  |  | LL | UL |
| Direct effects |  |  |  |  |  |  |  |
| NSUP→AUT | .547^***^ | .487 | .607 |  | .665^***^ | .603 | .726 |
| NSUP→COM | .595^***^ | .523 | .667 |  | .716^***^ | .643 | .789 |
| NSUP→REL | .445^***^ | .373 | .517 |  | .566^***^ | .491 | .641 |
| AUT→IM | .219^***^ | .128 | .310 |  | .248^***^ | .113 | .383 |
| AUT→ID | .196^***^ | .088 | .303 |  | .168 | -.003 | .340 |
| AUT→IJ | -.114 | -.269 | .042 |  | -.065 | -.333 | .204 |
| AUT→ER | -.070 | -.201 | .061 |  | -.099 | -.276 | .077 |
| COM→IM | .272^***^ | .170 | .374 |  | .323^***^ | .180 | .465 |
| COM→ID | .472^***^ | .361 | .583 |  | .524^***^ | .347 | .700 |
| COM→IJ | .130 | -.049 | .309 |  | .275 | -.024 | .575 |
| COM→ER | .123 | -.045 | .291 |  | .254^*^ | .050 | .458 |
| REL→IM | .179^***^ | .077 | .281 |  | .182^*^ | .039 | .325 |
| REL→ID | .161^**^ | .050 | .272 |  | .167 | -.006 | .340 |
| REL→IJ | -.062 | -.223 | .099 |  | -.016 | -.287 | .255 |
| REL→ER | -.017 | -.171 | .137 |  | .076 | -.112 | .264 |
| IM→WE | .328^***^ | .247 | .410 |  | .374^***^ | .261 | .487 |
| IM→JP | .238^***^ | .154 | .323 |  | .300^***^ | .175 | .418 |
| IM→JS | .189^**^ | .070 | .308 |  | .193^*^ | .027 | .359 |
| IM→WB | .346^***^ | .203 | .488 |  | .379^***^ | .187 | .571 |
| IM→TU | -.285^***^ | -.391 | -.180 |  | -.259^***^ | -.398 | -.119 |
| IM→BU | -.206^***^ | -.312 | -.010 |  | -.155^*^ | -.297 | -.014 |
| ID→WE | .482^***^ | .394 | .571 |  | .569^***^ | .446 | .692 |
| ID→JP | .258^***^ | .152 | .364 |  | .358^***^ | .216 | .499 |
| ID→JS | .638^***^ | .513 | .763 |  | .774^***^ | .604 | .944 |
| ID→WB | .583^***^ | .442 | .723 |  | .696^***^ | .502 | .891 |
| ID→TU | -.329^***^ | -.453 | -.204 |  | -.210^**^ | -.371 | -.050 |
| ID→BU | -.407^***^ | -.528 | -.286 |  | -.272^***^ | -.426 | -.118 |
| IJ→WE | -.252^***^ | -.370 | -.133 |  | -.308^***^ | -.469 | -.147 |
| IJ→JP | -.202^**^ | -.331 | -.073 |  | -.271^**^ | -.444 | -.098 |
| IJ→JS | -.199^*^ | -.370 | -.029 |  | -.248^*^ | -.479 | -.016 |
| IJ→WB | -.263^***^ | -.414 | -.112 |  | -.319^**^ | -.524 | -.114 |
| IJ→TU | .485^***^ | .347 | .623 |  | .515^***^ | .347 | .683 |
| IJ→BU | .226^**^ | .072 | .379 |  | .167 | -.030 | .364 |
| ER→WE | .045 | -.044 | .133 |  | .106 | -.008 | .220 |
| ER→JP | .040 | -.056 | .136 |  | .127^*^ | .002 | .253 |
| ER→JS | -.048 | -.174 | .077 |  | .002 | -.162 | .166 |
| ER→WB | -.076 | -.224 | .071 |  | -.024 | -.207 | .158 |
| ER→TU | -.010 | -.121 | .100 |  | .068 | -.056 | .193 |
| ER→BU | .173^**^ | .069 | .278` |  | .327^***^ | .203 | .450 |
| Indirect effects |  |  |  |  |  |  |  |
| NSUP→NSAT→AM→JP | .205^***^ | .165 | .244 |  | .355^***^ | .298 | .413 |
| NSUP→NSAT→AM→JS | .361^***^ | .319 | .404 |  | .546^***^ | .486 | .606 |
| NSUP→NSAT→AM→TU | -.254^***^ | -.295 | -.213 |  | -.251^***^ | -.309 | -.194 |
| NSUP→NSAT→AM→BU | -.261^***^ | -.299 | -.224 |  | -.236^***^ | -.286 | -.185 |
| NSUP→NSAT→AM→WE | .340^***^ | .302 | .379 |  | .517^***^ | .463 | .571 |
| NSUP→NSAT→AM→WB | .392^***^ | .340 | .445 |  | .594^***^ | .522 | .666 |
| NSUP→NSAT→CM→JP | .004 | -.010 | .018 |  | -.019 | -.048 | .010 |
| NSUP→NSAT→CM→JS | .001 | -.013 | .016 |  | -.036^*^ | -.067 | -.004 |
| NSUP→NSAT→CM→TU | -.006 | -.039 | .026 |  | .086^**^ | .034 | .137 |
| NSUP→NSAT→CM→BU | .002 | -.017 | .020 |  | .076^***^ | .043 | .110 |
| NSUP→NSAT→CM→WE | .004 | -.013 | .021 |  | -.028 | -.060 | .004 |
| NSUP→NSAT→CM→WB | .002 | -.018 | .020 |  | -.050^*^ | -.086 | -.014 |
| Sums of indirect effects |  |  |  |  |  |  |  |
| NSUP→NSAT→AM/CM→JP^a^ | .208^***^ | .170 | .247 |  | .337^***^ | .292 | .381 |
| NSUP→NSAT→AM/CM→JS^b^ | .363^***^ | .324 | .401 |  | .511^***^ | .467 | .554 |
| NSUP→NSAT→AM/CM→TU^c^ | -.261^***^ | -.296 | -.225 |  | -.166^***^ | -.205 | -.127 |
| NSUP→NSAT→AM/CM→BU^d^ | -.259^***^ | -.294 | -.225 |  | -.159^***^ | -.197 | -.121 |
| NSUP→NSAT→AM/CM→WE^e^ | .345^***^ | .311 | .378 |  | .489^***^ | .451 | .528 |
| NSUP→NSAT→AM/CM→WB^f^ | .394^***^ | .346 | .441 |  | .544^***^ | .491 | .597 |
| Correlations |  |  |  |  |  |  |  |
| IM↔ER | .116^***^ | .061 | .171 |  | .131^***^ | .071 | .190 |
| IM↔IJ | .340^***^ | .249 | .431 |  | .323^***^ | .224 | .422 |
| IM↔ID | .088^*^ | .011 | .165 |  | .104^*^ | .022 | .187 |
| ER↔IJ | .316^***^ | .232 | .400 |  | .413^***^ | .321 | .505 |
| ER↔ID | .151^**^ | .050 | .251 |  | .127^*^ | .028 | .226 |
| IJ↔ID | .458^***^ | .359 | .557 |  | .408^***^ | .286 | .530 |
| AU↔COM | .184^***^ | .118 | .250 |  | .166^***^ | .093 | .239 |
| AU↔REL | .247^***^ | .183 | .310 |  | .245^***^ | .172 | .318 |
| COM↔REL | .193^***^ | .121 | .264 |  | .185^***^ | .104 | .265 |
| JP↔JS | .182^**^ | .047 | .317 |  | .169^*^ | .029 | .309 |
| JP↔TU | .040 | -.046 | .127 |  | .117^*^ | .027 | .206 |
| JP↔BU | -.121 | -.263 | .022 |  | -.051 | -.195 | .093 |
| JP↔WE | .170^***^ | .085 | .255 |  | .152^***^ | .062 | .242 |
| JP↔WB | .026 | -.183 | .234 |  | .000 | -.211 | .212 |
| JS↔TU | -.168^***^ | -.262 | -.074 |  | -.146^**^ | -.249 | -.043 |
| JS↔BU | -.170^**^ | -.288 | -.052 |  | -.155^*^ | -.276 | -.035 |
| JS↔WE | .153^**^ | .041 | .266 |  | .125^*^ | .006 | .244 |
| JS↔WB | .101 | -.020 | .222 |  | .062 | -.067 | .192 |
| TU↔BU | .271^***^ | .168 | .375 |  | .473^***^ | .368 | .580 |
| TU↔WE | .039 | -.068 | .146 |  | .060 | -.051 | .170 |
| TU↔WB | -.133 | -.299 | .033 |  | -.110 | -.278 | .058 |
| BU↔WE | -.086^*^ | -.156 | -.016 |  | -.070 | -.141 | .000 |
| BU↔WB | -.278^**^ | -.483 | -.071 |  | -.264^*^ | -.471 | -.057 |
| WE↔WB | .076 | -.105 | .257 |  | .044 | -.141 | .229 |

*Note*. Adjusted model parameters are adjusted for the following covariates: age, sex, study quality, and study design. ^a^Sum of indirect effects of need support on job performance through all self-determination theory constructs; ^b^Sum of indirect effects of need support on job satisfaction through all self-determination theory constructs; ^c^Sum of indirect effects of need support on turnover through all self-determination theory constructs; ^d^Sum of indirect effects of need support on burnout through all self-determination theory constructs; ^e^Sum of indirect effects of need support on work engagement through all self-determination theory constructs; ^f^Sum of indirect effects of need support on well-being through all self-determination theory constructs. β = Standardized path coefficient; 95% CI = 95% confidence interval of parameter estimate; LL = Lower limit of 95% CI; UL = Upper limit of 95% CI; IM = Intrinsic motivation; ER = External regulation; AUT = Autonomy need satisfaction; COM = Competence need satisfaction; REL = Relatedness need satisfaction; NSUP = Need support; ID = Identified regulation; IJ = Introjected regulation; JP = Job performance; JS = Job satisfaction; TU = Turnover; BU = Burnout; WE = Work engagement; WB = Well-Being; NSAT = All need satisfaction constructs; AM = Autonomous forms of motivation (IM, ID); CM = Controlled forms of motivation (IJ, ER).

^***^*p* < .001 ^**^*p* < .01 ^*^*p* < .05

**Table L2**

*Standardized Parameter Estimates for the Direct and Indirect Effects of Self-Determination Theory Constructs on Workplace Outcomes from the Multilevel Meta-Analytic Structural Equation Model of the Truncated Model Unadjusted and Adjusted for Covariates*

| Effect | Model unadjusted for covariates | | |  | Model adjusted for covariates | | |
| --- | --- | --- | --- | --- | --- | --- | --- |
|  | β | 95% CI | |  | β | 95% CI | |
|  |  | LL | UL |  |  | LL | UL |
| Direct effects |  |  |  |  |  |  |  |
| NSUP→NSAT | .601^***^ | .557 | .646 |  | .720^***^ | .675 | .765 |
| NSUP→AD | .310^***^ | .276 | .344 |  | .439^***^ | .396 | .482 |
| NSUP→MAL | -.301^***^ | -.355 | -.247 |  | -.161^***^ | -.222 | -.101 |
| NSAT→AM | .444^***^ | .406 | .482 |  | .575^***^ | .538 | .612 |
| NSAT→CM | .014 | -.036 | .064 |  | .170^***^ | .121 | .219 |
| AM→AD | .310^***^ | .276 | .344 |  | .355^***^ | .312 | .398 |
| AM→MAL | -.234^***^ | -.276 | -.192 |  | -.162^***^ | -.216 | -.108 |
| CM→AD | .002 | -.034 | .037 |  | .038 | -.002 | .078 |
| CM→MAL | .112^***^ | .059 | .166 |  | .282^***^ | .226 | .339 |
| Indirect effects |  |  |  |  |  |  |  |
| NSUP→NSAT→AM→AD | .083^***^ | .071 | .094 |  | .147^***^ | .127 | .166 |
| NSUP→NSAT→CM→AD | .000 | -.000 | .000 |  | .005^*^ | .000 | .009 |
| NSUP→NSAT→AM→MAL | -.062^***^ | -.074 | -.051 |  | -.067^***^ | -.090 | -.044 |
| NSUP→NSAT→CM→MAL | -.001 | -.003 | .004 |  | .035^***^ | .021 | .048 |
| Sums of indirect effects |  |  |  |  |  |  |  |
| NSUP→NSAT→CM/AM→AD^a^ | .083^***^ | .071 | .094 |  | .152^***^ | .133 | .171 |
| NSUP→NSAT→CM/AM→MAL^b^ | -.062^***^ | -.073 | -.049 |  | -.032^**^ | -.056 | -.009 |
| Total Effects |  |  |  |  |  |  |  |
| NSUP→AD^c^ | .468^***^ | .434 | .502 |  | .591^***^ | .558 | .623 |
| NSUP→MAL^d^ | -.363^***^ | -.411 | -.315 |  | -.194^***^ | -.239 | -.149 |
| Correlations |  |  |  |  |  |  |  |
| AM↔CM | .162^***^ | .123 | .201 |  | .210^***^ | .168 | .251 |
| AD↔MAL | -.114^***^ | -.154 | -.073 |  | -.075^***^ | -.115 | -.034 |

*Note*. Adjusted model parameters are adjusted for the following covariates: age, sex, study quality, and study design. ^a^Sum of indirect effects of need support on adaptive workplace outcomes through all self-determination theory constructs; ^b^Sum of indirect effects of need support on maladaptive workplace outcomes through all self-determination theory constructs; ^c^Total effect of need support on adaptive workplace outcomes; ^d^Total effect of need support on maladaptive workplace outcomes. β = Standardized path coefficient; 95% CI = 95% confidence interval of parameter estimate; LL = Lower limit of 95% CI; UL = Upper limit of 95% CI; AM= Autonomous motivation; CM = Controlled motivation; NSUP = Need support; AD = Adaptive workplace outcomes collapsed across job performance, job satisfaction, work engagement, and well-being outcome variables; MAL = Maladaptive workplace outcomes collapsed across turnover and burnout outcomes; NSAT = Need satisfaction collapsed across autonomy, competence, and relatedness constructs.

^***^*p* < .001 ^**^*p* < .01 ^*^*p* < .05

Supplement M

**Table M1**

*Standardized Parameter Estimates for the Direct and Indirect Effects of Self-Determination Theory Constructs on Workplace Outcomes from the Multilevel Meta-Analytic Structural Equation Model of the Truncated Model for Corporate Employee and Healthcare Employee Type Moderator Groups with Comparisons*

| Effect |  | Comparison group: Employee type, corporate | | |  | Comparison group: Employee type, healthcare | | | Model comparisons | | | | |
| --- | --- | --- | --- | --- | --- | --- | --- | --- | --- | --- | --- | --- | --- |
|  |  | β | 95% CI | |  | β | 95% CI | | β_diff_ | 95% CI | | *t* | *p* |
|  |  |  | LL | UL |  |  | LL | UL |  | LL | UL |  |  |
| Direct effects |  |  |  |  |  |  |  |  |  |  |  |  |  |
| NSAT→AM |  | .627^***^ | .562 | .691 |  | .253^***^ | .169 | .336 | .374 | .268 | .479 | 6.951 | <.001 |
| NSAT→CM |  | .239^***^ | .152 | .325 |  | -.094 | -.192 | .004 | .333 | .203 | .464 | 5.002 | <.001 |
| NSUP→NSAT |  | .901^***^ | .773 | 1.030 |  | .504^***^ | .424 | .584 | .397 | .246 | .548 | 5.139 | <.001 |
| AM→AD |  | .387^***^ | .296 | .479 |  | .303^***^ | .226 | .380 | .084 | -.036 | .204 | 1.378 | .168 |
| CM→AD |  | .023 | -.048 | .095 |  | -.073 | -.170 | .025 | .096 | -.025 | .217 | 1.558 | .119 |
| NSUP→AD |  | .387^***^ | .293 | .481 |  | .306^***^ | .233 | .379 | .081 | -.038 | .200 | 1.336 | .182 |
| AM→MAL |  | -.079 | -.208 | .050 |  | -.371^***^ | -.459 | -.282 | .291 | .135 | .448 | 3.649 | <.001 |
| CM→MAL |  | .436^***^ | .320 | .553 |  | -.156^**^ | -.267 | -.046 | .593 | .432 | .753 | 7.236 | <.001 |
| NSUP→MAL |  | -.120 | -.254 | .014 |  | -.408^***^ | -.524 | -.292 | .288 | .111 | .465 | 3.184 | .001 |
|  |  |  |  |  |  |  |  |  |  |  |  |  |  |
| Indirect effects |  |  |  |  |  |  |  |  |  |  |  |  |  |
| NSUP→NSAT→AM→AD |  | .219^***^ | .160 | .277 |  | .039^***^ | .021 | .056 | .180 | .119 | .241 | 5.775 | <.001 |
| NSUP→NSAT→CM→AD |  | .005 | -.010 | .020 |  | .003 | -.003 | .010 | .002 | -.014 | .018 | 0.194 | .846 |
| NSUP→NSAT→AM→MAL |  | -.045 | -.117 | .028 |  | -.047^***^ | -.064 | -.030 | .002 | -.072 | .077 | 0.063 | .950 |
| NSUP→NSAT→CM→MAL |  | .094^***^ | .046 | .142 |  | .007 | -.003 | .018 | .086 | .037 | .136 | 3.437 | .001 |
|  |  |  |  |  |  |  |  |  |  |  |  |  |  |
| Sums of indirect effects |  |  |  |  |  |  |  |  |  |  |  |  |  |
| NSUP→NSAT→AM/CM→AD^a^ |  | .224^***^ | .169 | .279 |  | .042^***^ | .025 | .059 | .182 | .124 | .240 | 6.161 | <.001 |
| NSUP→NSAT→AM/CM→MAL^b^ |  | .049 | -.034 | .132 |  | -.040^***^ | -.060 | -.019 | .089 | .003 | .175 | 2.025 | .043 |
|  |  |  |  |  |  |  |  |  |  |  |  |  |  |
| Total Effects |  |  |  |  |  |  |  |  |  |  |  |  |  |
| NSUP→AD^c^ |  | .611^***^ | .554 | .668 |  | .348^***^ | .227 | .419 | .263 | .172 | .354 | 5.678 | <.001 |
| NSUP→MAL^d^ |  | -.071 | -.149 | .006 |  | -.448^***^ | -.554 | -.342 | .377 | .245 | .508 | 5.622 | <.001 |
|  |  |  |  |  |  |  |  |  |  |  |  |  |  |
| Correlations |  |  |  |  |  |  |  |  |  |  |  |  |  |
| AM↔CM |  | .233^***^ | .162 | .305 |  | .076 | -.032 | .184 | .157 | .027 | .286 | 2.375 | .018 |
| AD↔MAL |  | -.016 | -.090 | .059 |  | -.303^***^ | -.406 | -.201 | .288 | .161 | .414 | 4.464 | <.001 |

*Note*. Model parameters are adjusted for the following covariates: age, sex, study quality, and study design. ^a^Sum of indirect effects of need support on adaptive workplace outcomes through need support and all motivational forms; ^b^Sum of indirect effects of need support on maladaptive workplace outcomes through need support and all motivational forms; ^c^Total effect of need support on adaptive workplace outcomes; ^d^Total effect of need support on maladaptive workplace outcomes. β = Standardized parameter estimate; 95% CI = 95% confidence interval; LL = Lower limit of 95% CI; UL = Upper limit of 95% CI; β_diff_ = Difference in standardized path coefficient; *t* = Test of difference in standardized path coefficient based on confidence interval about the mean difference (Schenker & Gentleman, 2001); AM= Autonomous motivation; CM = Controlled motivation; NSUP = Need support; AD = Adaptive workplace outcomes collapsed across job performance, job satisfaction, work engagement, and well-being outcome variables; MAL = Maladaptive workplace outcomes collapsed across turnover and burnout outcomes; NSAT = Need satisfaction collapsed across autonomy, competence, and relatedness constructs.

^***^*p* < .001 ^**^*p* < .01 ^*^*p* < .05

**Table M2**

*Standardized Parameter Estimates for the Direct and Indirect Effects of Self-Determination Theory Constructs on Workplace Outcomes from the Multilevel Meta-Analytic Structural Equation Model of the Truncated Model for Corporate Employee and Healthcare Employee Type Moderator Groups with Comparisons*

| Effect |  | Comparison group: Employee type, corporate | | |  | Comparison group: Employee type, teachers | | | Model comparisons | | | | |
| --- | --- | --- | --- | --- | --- | --- | --- | --- | --- | --- | --- | --- | --- |
|  |  | β | 95% CI | |  | β | 95% CI | | β_diff_ | 95% CI | | *t* | *p* |
|  |  |  | LL | UL |  |  | LL | UL |  | LL | UL |  |  |
| Direct effects |  |  |  |  |  |  |  |  |  |  |  |  |  |
| NSAT→AM |  | .627^***^ | .562 | .691 |  | .425^***^ | .354 | .495 | .202 | .106 | .297 | 0.202 | .106 |
| NSAT→CM |  | .239^***^ | .152 | .325 |  | -.093^*^ | -.171 | -.015 | .332 | .215 | .448 | 0.332 | .215 |
| NSUP→NSAT |  | .901^***^ | .773 | 1.030 |  | .484^***^ | .404 | .564 | .417 | .266 | .569 | 0.417 | .266 |
| AM→AD |  | .387^***^ | .296 | .479 |  | .398^***^ | .317 | .479 | -.011 | -.134 | .113 | -0.011 | -.134 |
| CM→AD |  | .023 | -.048 | .095 |  | -.098^*^ | -.192 | -.003 | .121 | .003 | .239 | 0.121 | .003 |
| NSUP→AD |  | .387^***^ | .293 | .481 |  | .412^***^ | .312 | .512 | -.025 | -.162 | .113 | -0.025 | -.162 |
| AM→MAL |  | -.079 | -.208 | .050 |  | -.375^***^ | -.466 | -.283 | .296 | .137 | .454 | 0.296 | .137 |
| CM→MAL |  | .436^***^ | .320 | .553 |  | .136^*^ | .021 | .251 | .300 | .136 | .464 | 0.300 | .136 |
| NSUP→MAL |  | -.120 | -.254 | .014 |  | -.353^***^ | -.454 | -.253 | .233 | .066 | .400 | 0.233 | .066 |
|  |  |  |  |  |  |  |  |  |  |  |  |  |  |
| Indirect effects |  |  |  |  |  |  |  |  |  |  |  |  |  |
| NSUP→NSAT→AM→AD |  | .219^***^ | .160 | .277 |  | .082^***^ | .060 | .104 | .137 | .074 | .199 | 4.286 | <.001 |
| NSUP→NSAT→CM→AD |  | .005 | -.010 | .020 |  | .004 | -.001 | .010 | .001 | -.015 | .017 | 0.130 | .897 |
| NSUP→NSAT→AM→MAL |  | -.045 | -.117 | .028 |  | -.077^***^ | -.098 | -.056 | .032 | -.043 | .108 | 0.836 | .403 |
| NSUP→NSAT→CM→MAL |  | .094^***^ | .046 | .142 |  | -.006 | -.013 | .001 | .100 | -.001 | .200 | 4.019 | <.001 |
|  |  |  |  |  |  |  |  |  |  |  |  |  |  |
| Sums of indirect effects |  |  |  |  |  |  |  |  |  |  |  |  |  |
| NSUP→NSAT→AM/CM→AD^a^ |  | .224^***^ | .169 | .279 |  | .086^***^ | .064 | .108 | .138 | .079 | .197 | 4.543 | <.001 |
| NSUP→NSAT→AM/CM→MAL^b^ |  | .049 | -.034 | .132 |  | -.083^***^ | -.105 | -.061 | .132 | .046 | .218 | 2.997 | .003 |
|  |  |  |  |  |  |  |  |  |  |  |  |  |  |
| Total Effects |  |  |  |  |  |  |  |  |  |  |  |  |  |
| NSUP→AD^c^ |  | .611^***^ | .554 | .668 |  | .498^***^ | .410 | .587 | .113 | .008 | .218 | 2.104 | .035 |
| NSUP→MAL^d^ |  | -.071 | -.149 | .006 |  | -.436^***^ | -.525 | -.348 | .365 | .287 | .443 | 6.077 | <.001 |
|  |  |  |  |  |  |  |  |  |  |  |  |  |  |
| Correlations |  |  |  |  |  |  |  |  |  |  |  |  |  |
| AM↔CM |  | .233^***^ | .162 | .305 |  | .057 | <.001 | .052 | .176 | .059 | .293 | 3.306 | .001 |
| AD↔MAL |  | -.016 | -.090 | .059 |  | -.081 | -.180 | .019 | .065 | -.059 | .190 | 1.034 | .301 |

*Note*. Model parameters are adjusted for the following covariates: age, sex, study quality, and study design. ^a^Sum of indirect effects of need support on adaptive workplace outcomes through need support and all motivational forms; ^b^Sum of indirect effects of need support on maladaptive workplace outcomes through need support and all motivational forms; ^c^Total effect of need support on adaptive workplace outcomes; ^d^Total effect of need support on maladaptive workplace outcomes. β = Standardized parameter estimate; 95% CI = 95% confidence interval; LL = Lower limit of 95% CI; UL = Upper limit of 95% CI; β_diff_ = Difference in standardized path coefficient; *t* = Test of difference in standardized path coefficient based on confidence interval about the mean difference (Schenker & Gentleman, 2001); AM= Autonomous motivation; CM = Controlled motivation; NSUP = Need support; AD = Adaptive workplace outcomes collapsed across job performance, job satisfaction, work engagement, and well-being outcome variables; MAL = Maladaptive workplace outcomes collapsed across turnover and burnout outcomes; NSAT = Need satisfaction collapsed across autonomy, competence, and relatedness constructs.

^***^*p* < .001 ^**^*p* < .01 ^*^*p* < .05

**Table M3**

*Standardized Parameter Estimates for the Direct and Indirect Effects of Self-Determination Theory Constructs on Workplace Outcomes from the Multilevel Meta-Analytic Structural Equation Model of the Truncated Model for Corporate Employee and Mixed-Work or ‘Other’ Employee Type Moderator Groups with Comparisons*

| Effect |  | Comparison group: Employee type, corporate | | |  | Comparison group: Employee type, other | | | Model comparisons | | | | |
| --- | --- | --- | --- | --- | --- | --- | --- | --- | --- | --- | --- | --- | --- |
|  |  | β | 95% CI | |  | β | 95% CI | | β_diff_^a^ | 95% CI | | *t*^b^ | *p* |
|  |  |  | LL | UL |  |  | LL | UL |  | LL | UL |  |  |
| Direct effects |  |  |  |  |  |  |  |  |  |  |  |  |  |
| NSAT→AM |  | .627^***^ | .562 | .691 |  | .367^***^ | .291 | .442 | .260 | .160 | .359 | 5.129 | .000 |
| NSAT→CM |  | .239^***^ | .152 | .325 |  | .080 | -.075 | .236 | .159 | -.019 | .336 | 1.751 | .080 |
| NSUP→NSAT |  | .901^***^ | .773 | 1.030 |  | .493^***^ | .419 | .567 | .408 | .260 | .557 | 5.394 | .000 |
| AM→AD |  | .387^***^ | .296 | .479 |  | .197^***^ | .147 | .247 | .190 | .085 | .295 | 3.572 | .000 |
| CM→AD |  | .023 | -.048 | .095 |  | -.024 | -.083 | .034 | .047 | -.045 | .140 | 1.008 | .313 |
| NSUP→AD |  | .387^***^ | .293 | .481 |  | .289^***^ | .221 | .356 | .098 | -.017 | .214 | 1.658 | .097 |
| AM→MAL |  | -.079 | -.208 | .050 |  | -.357^***^ | -.429 | -.286 | .278 | .130 | .426 | 3.689 | .000 |
| CM→MAL |  | .436^***^ | .320 | .553 |  | -.058 | -.158 | .041 | .494 | .341 | .648 | 6.320 | .000 |
| NSUP→MAL |  | -.120 | -.254 | .014 |  | -.501^***^ | -.608 | -.393 | .381 | .209 | .553 | 4.343 | .000 |
|  |  |  |  |  |  |  |  |  |  |  |  |  |  |
| Indirect effects |  |  |  |  |  |  |  |  |  |  |  |  |  |
| NSUP→NSAT→AM→AD |  | .219^***^ | .160 | .277 |  | .036^***^ | .023 | .048 | .183 | .123 | .243 | 5.983 | .000 |
| NSUP→NSAT→CM→AD |  | .005 | -.010 | .020 |  | -.001 | -.004 | .002 | .006 | -.009 | .021 | 0.789 | .430 |
| NSUP→NSAT→AM→MAL |  | -.045 | -.117 | .028 |  | -.065^***^ | .008 | -.081 | .020 | -.054 | .094 | 0.533 | .594 |
| NSUP→NSAT→CM→MAL |  | .094^***^ | .046 | .142 |  | -.002 | -.007 | .003 | .096 | .047 | .144 | 3.877 | .000 |
|  |  |  |  |  |  |  |  |  |  |  |  |  |  |
| Sums of indirect effects |  |  |  |  |  |  |  |  |  |  |  |  |  |
| NSUP→NSAT→AM/CM→AD^a^ |  | .224^***^ | .169 | .279 |  | .035^***^ | .022 | .047 | .189 | .132 | .246 | 6.534 | .000 |
| NSUP→NSAT→AM/CM→MAL^b^ |  | .049 | -.034 | .132 |  | -.067^***^ | -.084 | -.050 | .116 | .031 | .201 | 2.669 | .008 |
|  |  |  |  |  |  |  |  |  |  |  |  |  |  |
| Total Effects |  |  |  |  |  |  |  |  |  |  |  |  |  |
| NSUP→AD^c^ |  | .611^***^ | .554 | .668 |  | .323^***^ | .259 | .388 | .288 | .202 | .374 | 6.558 | .000 |
| NSUP→MAL^d^ |  | -.071 | -.149 | .006 |  | -.568^***^ | -.667 | -.468 | .497 | .370 | .623 | 7.719 | .000 |
|  |  |  |  |  |  |  |  |  |  |  |  |  |  |
| Correlations |  |  |  |  |  |  |  |  |  |  |  |  |  |
| AM↔CM |  | .233^***^ | .162 | .305 |  | .050 | -.032 | .131 | .183 | .075 | .291 | 3.308 | .001 |
| AD↔MAL |  | -.016 | -.090 | .059 |  | -.216 | .037 | -.289 | .200 | .097 | .304 | 3.788 | .000 |

*Note*. Model parameters are adjusted for the following covariates: age, sex, study quality, and study design. ^a^Sum of indirect effects of need support on adaptive workplace outcomes through need support and all motivational forms; ^b^Sum of indirect effects of need support on maladaptive workplace outcomes through need support and all motivational forms; ^c^Total effect of need support on adaptive workplace outcomes; ^d^Total effect of need support on maladaptive workplace outcomes. β = Standardized parameter estimate; 95% CI = 95% confidence interval; LL = Lower limit of 95% CI; UL = Upper limit of 95% CI; β_diff_ = Difference in standardized path coefficient; *t* = Test of difference in standardized path coefficient based on confidence interval about the mean difference (Schenker & Gentleman, 2001); AM= Autonomous motivation; CM = Controlled motivation; NSUP = Need support; AD = Adaptive workplace outcomes collapsed across job performance, job satisfaction, work engagement, and well-being outcome variables; MAL = Maladaptive workplace outcomes collapsed across turnover and burnout outcomes; NSAT = Need satisfaction collapsed across autonomy, competence, and relatedness constructs.

^***^*p* < .001 ^**^*p* < .01 ^*^*p* < .05

**Table M4**

*Standardized Parameter Estimates for the Direct and Indirect Effects of Self-Determination Theory Constructs on Workplace Outcomes from the Multilevel Meta-Analytic Structural Equation Model of the Truncated Model for Healthcare Employee and Teacher Employee type Moderator Groups with Comparisons*

| Effect |  | Comparison group: Employee type, healthcare | | |  | Comparison group: Employee type, teachers | | | Model comparisons | | | | |
| --- | --- | --- | --- | --- | --- | --- | --- | --- | --- | --- | --- | --- | --- |
|  |  | β | 95% CI | |  | β | 95% CI | | β_diff_^a^ | 95% CI | | *t*^b^ | *p* |
|  |  |  | LL | UL |  |  | LL | UL |  | LL | UL |  |  |
| Direct effects |  |  |  |  |  |  |  |  |  |  |  |  |  |
| NSAT→AM |  | .253^***^ | .169 | .336 |  | .425^***^ | .354 | .495 | -.172 | -.256 | -.089 | -3.093 | .002 |
| NSAT→CM |  | -.094 | -.192 | .004 |  | -.093^*^ | -.171 | -.015 | -.001 | -.099 | .097 | -0.023 | .982 |
| NSUP→NSAT |  | .504^***^ | .424 | .584 |  | .484^***^ | .404 | .564 | .020 | -.086 | .127 | 0.352 | .725 |
| AM→AD |  | .303^***^ | .226 | .380 |  | .398^***^ | .317 | .479 | -.095 | -.205 | .015 | -1.661 | .097 |
| CM→AD |  | -.073 | -.170 | .025 |  | -.098^*^ | -.192 | -.003 | .025 | -.101 | .152 | 0.367 | .714 |
| NSUP→AD |  | .306^***^ | .233 | .379 |  | .412^***^ | .312 | .512 | -.106 | -.216 | .004 | -1.681 | .093 |
| AM→MAL |  | -.371^***^ | -.459 | -.282 |  | -.375^***^ | -.466 | -.283 | .004 | -.125 | .133 | 0.067 | .946 |
| CM→MAL |  | -.156^**^ | -.267 | -.046 |  | .136^*^ | .021 | .251 | -.292 | -.441 | -.144 | -3.596 | .000 |
| NSUP→MAL |  | -.408^***^ | -.524 | -.292 |  | -.353^***^ | -.454 | -.253 | -.055 | -.203 | .093 | -0.704 | .481 |
|  |  |  |  |  |  |  |  |  |  |  |  |  |  |
| Indirect effects |  |  |  |  |  |  |  |  |  |  |  |  |  |
| NSUP→NSAT→AM→AD |  | .039^***^ | .021 | .056 |  | .082^***^ | .060 | .104 | -.043 | -.066 | -.021 | -3.017 | .003 |
| NSUP→NSAT→CM→AD |  | .003 | -.003 | .010 |  | .004 | -.001 | .010 | -.001 | -.048 | .047 | -0.128 | .898 |
| NSUP→NSAT→AM→MAL |  | -.047^***^ | -.064 | -.030 |  | -.077^***^ | -.098 | -.056 | .030 | .002 | .057 | 2.156 | .031 |
| NSUP→NSAT→CM→MAL |  | .007 | -.003 | .018 |  | -.006 | -.013 | .001 | .013 | -.075 | .102 | 2.122 | .034 |
|  |  |  |  |  |  |  |  |  |  |  |  |  |  |
| Sums of indirect effects |  |  |  |  |  |  |  |  |  |  |  |  |  |
| NSUP→NSAT→AM/CM→AD^a^ |  | .042^***^ | .025 | .059 |  | .086^***^ | .064 | .108 | -.044 | -.071 | -.016 | -3.092 | .002 |
| NSUP→NSAT→AM/CM→MAL^b^ |  | -.040^***^ | -.060 | -.019 |  | -.083^***^ | -.105 | -.061 | .043 | .014 | .073 | 2.828 | .005 |
|  |  |  |  |  |  |  |  |  |  |  |  |  |  |
| Total Effects |  |  |  |  |  |  |  |  |  |  |  |  |  |
| NSUP→AD^c^ |  | .348^***^ | .227 | .419 |  | .498^***^ | .410 | .587 | -.150 | -.221 | -.079 | -2.596 | .009 |
| NSUP→MAL^d^ |  | -.448^***^ | -.554 | -.342 |  | -.436^***^ | -.525 | -.348 | -.012 | -.118 | .094 | -0.169 | .866 |
|  |  |  |  |  |  |  |  |  |  |  |  |  |  |
| Correlations |  |  |  |  |  |  |  |  |  |  |  |  |  |
| AM↔CM |  | .076 | -.032 | .184 |  | .057 | .000 | .052 | .019 | -.139 | .177 | 0.285 | .775 |
| AD↔MAL |  | -.303^***^ | -.406 | -.201 |  | -.081 | -.180 | .019 | -.222 | -.365 | -.079 | -3.054 | .002 |

*Note*. Model parameters are adjusted for the following covariates: age, sex, study quality, and study design. ^a^Sum of indirect effects of need support on adaptive workplace outcomes through need support and all motivational forms; ^b^Sum of indirect effects of need support on maladaptive workplace outcomes through need support and all motivational forms; ^c^Total effect of need support on adaptive workplace outcomes; ^d^Total effect of need support on maladaptive workplace outcomes. β = Standardized parameter estimate; 95% CI = 95% confidence interval; LL = Lower limit of 95% CI; UL = Upper limit of 95% CI; β_diff_ = Difference in standardized path coefficient; *t* = Test of difference in standardized path coefficient based on confidence interval about the mean difference (Schenker & Gentleman, 2001); AM= Autonomous motivation; CM = Controlled motivation; NSUP = Need support; AD = Adaptive workplace outcomes collapsed across job performance, job satisfaction, work engagement, and well-being outcome variables; MAL = Maladaptive workplace outcomes collapsed across turnover and burnout outcomes; NSAT = Need satisfaction collapsed across autonomy, competence, and relatedness constructs.

^***^*p* < .001 ^**^*p* < .01 ^*^*p* < .05

**Table M5**

*Standardized Parameter Estimates for the Direct and Indirect Effects of Self-Determination Theory Constructs on Workplace Outcomes from the Multilevel Meta-Analytic Structural Equation Model of the Truncated Model for Healthcare Employee and ‘Mixed-Work’ or Other Employee Type Moderator Groups with Comparisons*

| Effect |  | Comparison group: Employee type, healthcare | | |  | Comparison group: Employee type, others | | | Model comparisons | | | | |
| --- | --- | --- | --- | --- | --- | --- | --- | --- | --- | --- | --- | --- | --- |
|  |  | β | 95% CI | |  | β | 95% CI | | β_diff_^a^ | 95% CI | | *t*^b^ | *p* |
|  |  |  | LL | UL |  |  | LL | UL |  | LL | UL |  |  |
| Direct effects |  |  |  |  |  |  |  |  |  |  |  |  |  |
| NSAT→AM |  | .253^***^ | .169 | .336 |  | .367^***^ | .291 | .442 | -.114 | -.227 | -.001 | -1.992 | .046 |
| NSAT→CM |  | -.094 | -.192 | .004 |  | .080 | -.075 | .236 | -.174 | -.358 | .009 | -1.862 | .063 |
| NSUP→NSAT |  | .504^***^ | .424 | .584 |  | .493^***^ | .419 | .567 | .011 | -.098 | .121 | 0.203 | .839 |
| AM→AD |  | .303^***^ | .226 | .380 |  | .197^***^ | .147 | .247 | .106 | .014 | .199 | 2.262 | .024 |
| CM→AD |  | -.073 | -.170 | .025 |  | -.024 | -.083 | .034 | -.049 | -.162 | .065 | -0.838 | .402 |
| NSUP→AD |  | .306^***^ | .233 | .379 |  | .289^***^ | .221 | .356 | .017 | -.082 | .116 | 0.335 | .738 |
| AM→MAL |  | -.371^***^ | -.459 | -.282 |  | -.357^***^ | -.429 | -.286 | -.014 | -.128 | .101 | -0.235 | .814 |
| CM→MAL |  | -.156^**^ | -.267 | -.046 |  | -.058 | -.158 | .041 | -.098 | -.247 | .050 | -1.299 | .194 |
| NSUP→MAL |  | -.408^***^ | -.524 | -.292 |  | -.501^***^ | -.608 | -.393 | .093 | -.065 | .251 | 1.151 | .250 |
|  |  |  |  |  |  |  |  |  |  |  |  |  |  |
| Indirect effects |  |  |  |  |  |  |  |  |  |  |  |  |  |
| NSUP→NSAT→AM→AD |  | .039^***^ | .021 | .056 |  | .036^***^ | .023 | .048 | .003 | -.019 | .024 | 0.238 | .812 |
| NSUP→NSAT→CM→AD |  | .003 | -.003 | .010 |  | -.001 | -.004 | .002 | .004 | -.003 | .012 | 1.261 | .207 |
| NSUP→NSAT→AM→MAL |  | -.047^***^ | -.064 | -.030 |  | -.065^***^ | .008 | -.081 | .018 | -.005 | .041 | 1.489 | .136 |
| NSUP→NSAT→CM→MAL |  | .007 | -.003 | .018 |  | -.002 | .003 | -.007 | .009 | -.002 | .021 | 1.622 | .105 |
|  |  |  |  |  |  |  |  |  |  |  |  |  |  |
| Sums of indirect effects |  |  |  |  |  |  |  |  |  |  |  |  |  |
| NSUP→NSAT→AM/CM→AD^a^ |  | .042^***^ | .025 | .059 |  | .035^***^ | .022 | .047 | .007 | -.015 | .029 | 0.657 | .511 |
| NSUP→NSAT→AM/CM→MAL^b^ |  | -.040^***^ | -.060 | -.019 |  | -.067^***^ | -.084 | -.050 | .027 | .000 | .054 | 2.014 | .044 |
|  |  |  |  |  |  |  |  |  |  |  |  |  |  |
| Total Effects |  |  |  |  |  |  |  |  |  |  |  |  |  |
| NSUP→AD^c^ |  | .348^***^ | .227 | .419 |  | .323^***^ | .259 | .388 | .025 | -.071 | .121 | 0.513 | .608 |
| NSUP→MAL^d^ |  | -.448^***^ | -.554 | -.342 |  | -.568^***^ | -.667 | -.468 | .120 | -.026 | .266 | 1.619 | .105 |
|  |  |  |  |  |  |  |  |  |  |  |  |  |  |
| Correlations |  |  |  |  |  |  |  |  |  |  |  |  |  |
| AM↔CM |  | .076 | -.032 | .184 |  | .050 | -.032 | .131 | .026 | -.108 | .161 | 0.380 | .704 |
| AD↔MAL |  | -.303^***^ | -.406 | -.201 |  | -.216 | .037 | -.289 | -.087 | -.213 | .038 | -1.366 | .172 |

*Note*. Model parameters are adjusted for the following covariates: age, sex, study quality, and study design. ^a^Sum of indirect effects of need support on adaptive workplace outcomes through need support and all motivational forms; ^b^Sum of indirect effects of need support on maladaptive workplace outcomes through need support and all motivational forms; ^c^Total effect of need support on adaptive workplace outcomes; ^d^Total effect of need support on maladaptive workplace outcomes. β = Standardized parameter estimate; 95% CI = 95% confidence interval; LL = Lower limit of 95% CI; UL = Upper limit of 95% CI; β_diff_ = Difference in standardized path coefficient; *t* = Test of difference in standardized path coefficient based on confidence interval about the mean difference (Schenker & Gentleman, 2001); AM= Autonomous motivation; CM = Controlled motivation; NSUP = Need support; AD = Adaptive workplace outcomes collapsed across job performance, job satisfaction, work engagement, and well-being outcome variables; MAL = Maladaptive workplace outcomes collapsed across turnover and burnout outcomes; NSAT = Need satisfaction collapsed across autonomy, competence, and relatedness constructs.

^***^*p* < .001 ^**^*p* < .01 ^*^*p* < .05

**Table M6**

*Standardized Parameter Estimates for the Direct and Indirect Effects of Self-Determination Theory Constructs on Workplace Outcomes from the Multilevel Meta-Analytic Structural Equation Model of the Truncated Model for Comparing Teacher and Mixed-Work ‘Other’ Employee Type Moderator Groups with Comparisons*

| Effect |  | Comparison group: Employee type, teachers | | |  | Comparison group: Employee type, others | | | Model comparisons | | | | |
| --- | --- | --- | --- | --- | --- | --- | --- | --- | --- | --- | --- | --- | --- |
|  |  | β | 95% CI | |  | β | 95% CI | | β_diff_^a^ | 95% CI | | *t*^b^ | *p* |
|  |  |  | LL | UL |  |  | LL | UL |  | LL | UL |  |  |
| Direct effects |  |  |  |  |  |  |  |  |  |  |  |  |  |
| NSAT→AM |  | .425^***^ | .354 | .495 |  | .367^***^ | .291 | .442 | .058 | -.046 | .162 | 1.101 | .271 |
| NSAT→CM |  | -.093^*^ | -.171 | -.015 |  | .080 | -.075 | .236 | -.173 | -.347 | .001 | -1.952 | .051 |
| NSUP→NSAT |  | .484^***^ | .404 | .564 |  | .493^***^ | .419 | .567 | -.009 | -.119 | .101 | -0.162 | .871 |
| AM→AD |  | .398^***^ | .317 | .479 |  | .197^***^ | .147 | .247 | .201 | .104 | .298 | 4.139 | .000 |
| CM→AD |  | -.098^*^ | -.192 | -.003 |  | -.024 | -.083 | .034 | -.074 | -.185 | .037 | -1.305 | .192 |
| NSUP→AD |  | .412^***^ | .312 | .512 |  | .289^***^ | .221 | .356 | .123 | .003 | .243 | 1.998 | .046 |
| AM→MAL |  | -.375^***^ | -.466 | -.283 |  | -.357^***^ | -.429 | -.286 | -.018 | -.135 | .099 | -0.304 | .761 |
| CM→MAL |  | .136^*^ | .021 | .251 |  | -.058 | -.158 | .041 | .194 | .041 | .347 | 2.500 | .012 |
| NSUP→MAL |  | -.353^***^ | -.454 | -.253 |  | -.501^***^ | -.608 | -.393 | .148 | .001 | .295 | 1.971 | .049 |
|  |  |  |  |  |  |  |  |  |  |  |  |  |  |
| Indirect effects |  |  |  |  |  |  |  |  |  |  |  |  |  |
| NSUP→NSAT→AM→AD |  | .082^***^ | .060 | .104 |  | .036^***^ | .023 | .048 | .046 | .021 | .071 | 3.563 | .000 |
| NSUP→NSAT→CM→AD |  | .004 | -.001 | .010 |  | -.001 | -.004 | .002 | .005 | -.002 | .012 | 1.564 | .118 |
| NSUP→NSAT→AM→MAL |  | -.077^***^ | -.098 | -.056 |  | -.065^***^ | .008 | -.081 | -.012 | -.039 | .015 | -0.891 | .373 |
| NSUP→NSAT→CM→MAL |  | -.006 | -.013 | .001 |  | -.002 | .003 | -.007 | -.004 | -.012 | .004 | -0.911 | .362 |
|  |  |  |  |  |  |  |  |  |  |  |  |  |  |
| Sums of indirect effects |  |  |  |  |  |  |  |  |  |  |  |  |  |
| NSUP→NSAT→AM/CM→AD^a^ |  | .086^***^ | .064 | .108 |  | .035^***^ | .022 | .047 | .051 | .025 | .077 | 3.950 | .000 |
| NSUP→NSAT→AM/CM→MAL^b^ |  | -.083^***^ | -.105 | -.061 |  | -.067^***^ | -.084 | -.050 | -.016 | -.044 | .012 | -1.128 | .259 |
|  |  |  |  |  |  |  |  |  |  |  |  |  |  |
| Total Effects |  |  |  |  |  |  |  |  |  |  |  |  |  |
| NSUP→AD^c^ |  | .498^***^ | .410 | .587 |  | .323^***^ | .259 | .388 | .175 | .066 | .284 | 3.132 | .002 |
| NSUP→MAL^d^ |  | -.436^***^ | -.525 | -.348 |  | -.568^***^ | -.667 | -.468 | .132 | -.001 | .265 | 1.943 | .052 |
|  |  |  |  |  |  |  |  |  |  |  |  |  |  |
| Correlations |  |  |  |  |  |  |  |  |  |  |  |  |  |
| AM↔CM |  | .057 | .000 | .052 |  | .050 | -.032 | .131 | .007 | -.115 | .129 | 0.123 | .902 |
| AD↔MAL |  | -.081 | -.180 | .019 |  | -.216 | .037 | -.289 | .135 | .012 | .258 | 2.149 | .032 |

*Note*. Model parameters are adjusted for the following covariates: age, sex, study quality, and study design. ^a^Sum of indirect effects of need support on adaptive workplace outcomes through need support and all motivational forms; ^b^Sum of indirect effects of need support on maladaptive workplace outcomes through need support and all motivational forms; ^c^Total effect of need support on adaptive workplace outcomes; ^d^Total effect of need support on maladaptive workplace outcomes. β = Standardized parameter estimate; 95% CI = 95% confidence interval; LL = Lower limit of 95% CI; UL = Upper limit of 95% CI; β_diff_ = Difference in standardized path coefficient; *t* = Test of difference in standardized path coefficient based on confidence interval about the mean difference (Schenker & Gentleman, 2001); AM= Autonomous motivation; CM = Controlled motivation; NSUP = Need support; AD = Adaptive workplace outcomes collapsed across job performance, job satisfaction, work engagement, and well-being outcome variables; MAL = Maladaptive workplace outcomes collapsed across turnover and burnout outcomes; NSAT = Need satisfaction collapsed across autonomy, competence, and relatedness constructs.

^***^*p* < .001 ^**^*p* < .01 ^*^*p* < .05

**Table M7**

*Standardized Parameter Estimates for the Direct and Indirect Effects of Self-Determination Theory Constructs on Workplace Outcomes from the Multilevel Meta-Analytic Structural Equation Model of the Truncated Model for Study Samples from Top-10 GDP Countries and Studies on Samples from Countries Not in the Top-10 GDP with Comparisons*

| Effect |  | Comparison group: GDP, top 10 | | |  | Comparison group: GDP, other | | | Model comparisons | | | | |
| --- | --- | --- | --- | --- | --- | --- | --- | --- | --- | --- | --- | --- | --- |
|  |  | β | 95% CI | |  | β | 95% CI | | β_diff_^a^ | 95% CI | | *t*^b^ | *p* |
|  |  |  | LL | UL |  |  | LL | UL |  | LL | UL |  |  |
| Direct effects |  |  |  |  |  |  |  |  |  |  |  |  |  |
| NSAT→AM |  | .533^***^ | .484 | .582 |  | .459^***^ | .398 | .520 | .074 | -.004 | .152 | 1.854 | .064 |
| NSAT→CM |  | .154^***^ | .091 | .218 |  | -.026 | -.011 | .055 | .180 | .077 | .283 | 3.415 | .001 |
| NSUP→NSAT |  | .692^***^ | .634 | .750 |  | .562^***^ | .491 | .633 | .130 | .038 | .222 | 2.779 | .005 |
| AM→AD |  | .368^***^ | .310 | .427 |  | .293^***^ | .247 | .338 | .075 | .001 | .149 | 1.984 | .047 |
| CM→AD |  | .016 | -.039 | .072 |  | .011 | -.038 | .060 | .005 | -.069 | .079 | 0.132 | .895 |
| NSUP→AD |  | .426^***^ | .369 | .484 |  | .361^***^ | .303 | .418 | .065 | -.015 | .145 | 1.567 | .117 |
| AM→MAL |  | -.174^***^ | -.240 | -.109 |  | -.239^***^ | -.303 | -.175 | .065 | -.026 | .156 | 1.391 | .164 |
| CM→MAL |  | .232^***^ | .161 | .303 |  | .137^**^ | .049 | .225 | .095 | -.018 | .208 | 1.647 | .100 |
| NSUP→MAL |  | -.171^***^ | -.242 | -.099 |  | -.323^***^ | -.417 | -.229 | .152 | .033 | .271 | 2.523 | .012 |
|  |  |  |  |  |  |  |  |  |  |  |  |  |  |
| Indirect effects |  |  |  |  |  |  |  |  |  |  |  |  |  |
| NSUP→NSAT→AM→AD |  | .136^***^ | .110 | .161 |  | .075^***^ | .060 | .091 | .061 | .031 | .091 | 4.007 | .000 |
| NSUP→NSAT→CM→AD |  | .002 | -.004 | .008 |  | .000 | -.001 | .001 | .002 | -.004 | .008 | 0.016 | .987 |
| NSUP→NSAT→AM→MAL |  | -.064^***^ | -.089 | -.040 |  | -.062^***^ | -.079 | -.044 | -.002 | -.033 | .029 | -0.131 | .895 |
| NSUP→NSAT→CM→MAL |  | .025^***^ | .010 | .039 |  | -.002 | -.008 | .004 | .027 | .012 | .042 | 3.372 | .001 |
|  |  |  |  |  |  |  |  |  |  |  |  |  |  |
| Sums of indirect effects |  |  |  |  |  |  |  |  |  |  |  |  |  |
| NSUP→NSAT→AM/CM→AD^a^ |  | .138^***^ | .113 | .162 |  | .075^***^ | .059 | .091 | .063 | .033 | .093 | 2.165 | .030 |
| NSUP→NSAT→AM/CM→MAL^b^ |  | -.040^***^ | -.065 | -.014 |  | -.064^***^ | -.082 | -.045 | .024 | -.007 | .055 | 1.493 | .135 |
|  |  |  |  |  |  |  |  |  |  |  |  |  |  |
| Total Effects |  |  |  |  |  |  |  |  |  |  |  |  |  |
| NSUP→AD^c^ |  | .564^***^ | .519 | .610 |  | .436^***^ | .384 | .488 | .128 | .060 | .196 | 1.284 | .199 |
| NSUP→MAL^d^ |  | -.210^***^ | -.268 | -.152 |  | -.387^***^ | -.471 | -.303 | .177 | .074 | .280 | 3.399 | .001 |
|  |  |  |  |  |  |  |  |  |  |  |  |  |  |
| Correlations |  |  |  |  |  |  |  |  |  |  |  |  |  |
| AM↔CM |  | .206^***^ | .152 | .259 |  | .153^***^ | .093 | .213 | .053 | -.028 | .134 | 1.292 | .196 |
| AD↔MAL |  | -.092^**^ | -.154 | -.030 |  | -.121^***^ | -.175 | -.066 | .029 | -.054 | .112 | 0.689 | .491 |

*Note*. Model parameters are adjusted for the following covariates: age, sex, study quality, and study design. ^a^Sum of indirect effects of need support on adaptive workplace outcomes through need support and all motivational forms; ^b^Sum of indirect effects of need support on maladaptive workplace outcomes through need support and all motivational forms; ^c^Total effect of need support on adaptive workplace outcomes; ^d^Total effect of need support on maladaptive workplace outcomes. β = Standardized parameter estimate; 95% CI = 95% confidence interval; LL = Lower limit of 95% CI; UL = Upper limit of 95% CI; β_diff_ = Difference in standardized path coefficient; *t* = Test of difference in standardized path coefficient based on confidence interval about the mean difference (Schenker & Gentleman, 2001); AM= Autonomous motivation; CM = Controlled motivation; NSUP = Need support; AD = Adaptive workplace outcomes collapsed across job performance, job satisfaction, work engagement, and well-being outcome variables; MAL = Maladaptive workplace outcomes collapsed across turnover and burnout outcomes; NSAT = Need satisfaction collapsed across autonomy, competence, and relatedness constructs.

^***^*p* < .001 ^**^*p* < .01 ^*^*p* < .05

**Table M8**

*Standardized Parameter Estimates for the Direct and Indirect Effects of Self-Determination Theory Constructs on Workplace Outcomes from the Multilevel Meta-Analytic Structural Equation Model of the Truncated Model for Studies on Employees Engaged in Public Service Work Type and Studies on Employees Engaged in For-Profit Work Type with Comparisons*

| Effect |  | Comparison group: Work type, public service | | |  | Comparison group: Work type, for-profit | | | Model comparisons | | | | |
| --- | --- | --- | --- | --- | --- | --- | --- | --- | --- | --- | --- | --- | --- |
|  |  | β | 95% CI | |  | β | 95% CI | | β_diff_^a^ | 95% CI | | *t*^b^ | *p* |
|  |  |  | LL | UL |  |  | LL | UL |  | LL | UL |  |  |
| Direct effects |  |  |  |  |  |  |  |  |  |  |  |  |  |
| NSAT→AM |  | .392^***^ | .319 | .466 |  | .670^***^ | .624 | .717 | -.278 | -.364 | -.192 | -6.279 | <.001 |
| NSAT→CM |  | -.085^***^ | -.156 | -.014 |  | .315^***^ | .237 | .393 | -.400 | -.505 | -.295 | -7.434 | <.001 |
| NSUP→NSAT |  | .505^***^ | .435 | .576 |  | .826^***^ | .766 | .886 | -.321 | -.413 | -.228 | -6.803 | <.001 |
| AM→AD |  | .364^***^ | .297 | .430 |  | .349^***^ | .282 | .416 | .015 | -.079 | .109 | 0.312 | .755 |
| CM→AD |  | -.084^***^ | -.158 | -.010 |  | .056 | -.003 | .114 | -.140 | -.234 | -.045 | -2.893 | .004 |
| NSUP→AD |  | .347^***^ | .270 | .424 |  | .452^***^ | .387 | .516 | -.105 | -.205 | -.005 | -2.045 | .041 |
| AM→MAL |  | -.320^***^ | -.381 | -.258 |  | -.124^*^ | -.222 | -.026 | -.196 | -.311 | -.081 | -3.318 | .001 |
| CM→MAL |  | .013 | -.066 | .091 |  | .463^***^ | .371 | .555 | -.450 | -.571 | -.329 | -7.295 | <.001 |
| NSUP→MAL |  | -.421^***^ | -.510 | -.033 |  | -.126^*^ | -.233 | -.020 | -.295 | -.434 | -.155 | -2.213 | .027 |
|  |  |  |  |  |  |  |  |  |  |  |  |  |  |
| Indirect effects |  |  |  |  |  |  |  |  |  |  |  |  |  |
| NSUP→NSAT→AM→AD |  | .072^***^ | .054 | .090 |  | .193^***^ | .155 | .231 | -.121 | -.163 | -.079 | -5.639 | <.001 |
| NSUP→NSAT→CM→AD |  | .004 | -.001 | .008 |  | .014^*^ | .001 | .028 | -.011 | -.025 | .004 | -1.457 | .145 |
| NSUP→NSAT→AM→MAL |  | -.063^***^ | -.079 | -.048 |  | -.069^*^ | -.123 | -.014 | .005 | -.051 | .061 | 0.183 | .855 |
| NSUP→NSAT→CM→MAL |  | -.001 | -.004 | .003 |  | .120^***^ | .077 | .164 | -.121 | -.165 | -.077 | -5.416 | <.001 |
|  |  |  |  |  |  |  |  |  |  |  |  |  |  |
| Sums of indirect effects |  |  |  |  |  |  |  |  |  |  |  |  |  |
| NSUP→NSAT→AM/CM→AD^a^ |  | .076^***^ | .057 | .094 |  | .208^***^ | .172 | .243 | -.132 | -.172 | -.092 | -6.410 | <.001 |
| NSUP→NSAT→AM/CM→MAL^b^ |  | -.064^***^ | -.080 | -.048 |  | .052 | -.015 | .118 | -.116 | -.184 | -.047 | -3.306 | .001 |
|  |  |  |  |  |  |  |  |  |  |  |  |  |  |
| Total Effects |  |  |  |  |  |  |  |  |  |  |  |  |  |
| NSUP→AD^c^ |  | .423^***^ | .356 | .490 |  | .659^***^ | .618 | .701 | -.236 | -.315 | -.158 | -5.911 | <.001 |
| NSUP→MAL^d^ |  | -.484^***^ | -.564 | -.405 |  | -.074^*^ | -.133 | -.016 | -.410 | -.509 | -.311 | -8.106 | <.001 |
|  |  |  |  |  |  |  |  |  |  |  |  |  |  |
| Correlations |  |  |  |  |  |  |  |  |  |  |  |  |  |
| AM↔CM |  | .194^***^ | .123 | .266 |  | .195^***^ | .131 | .259 | -.001 | -.098 | .096 | -0.019 | .985 |
| AD↔MAL |  | -.141^***^ | -.219 | -.064 |  | -.061^*^ | -.114 | -.007 | -.080 | -.173 | .013 | -1.671 | .095 |

*Note*. Model parameters are adjusted for the following covariates: age, sex, study quality, and study design. ^a^Sum of indirect effects of need support on adaptive workplace outcomes through need support and all motivational forms; ^b^Sum of indirect effects of need support on maladaptive workplace outcomes through need support and all motivational forms; ^c^Total effect of need support on adaptive workplace outcomes; ^d^Total effect of need support on maladaptive workplace outcomes. β = Standardized parameter estimate; 95% CI = 95% confidence interval; LL = Lower limit of 95% CI; UL = Upper limit of 95% CI; β_diff_ = Difference in standardized path coefficient; *t* = Test of difference in standardized path coefficient based on confidence interval about the mean difference (Schenker & Gentleman, 2001); AM= Autonomous motivation; CM = Controlled motivation; NSUP = Need support; AD = Adaptive workplace outcomes collapsed across job performance, job satisfaction, work engagement, and well-being outcome variables; MAL = Maladaptive workplace outcomes collapsed across turnover and burnout outcomes; NSAT = Need satisfaction collapsed across autonomy, competence, and relatedness constructs.

^***^*p* < .001 ^**^*p* < .01 ^*^*p* < .05

**Table M9**

*Standardized Parameter Estimates for the Direct and Indirect Effects of Self-Determination Theory Constructs on Workplace Outcomes from the Multilevel Meta-Analytic Structural Equation Model of the Truncated Model in the Full Sample of Studies and on Employees Engaged in ‘Mixed’ Public Service Work Types and For-Profit Work type with Comparisons (Sensitivity Analysis)*

| Effect | Full Sample | | |  | Comparison group: Work type, for-profit and mixed samples | | | Model comparisons | | | | |
| --- | --- | --- | --- | --- | --- | --- | --- | --- | --- | --- | --- | --- |
|  | β | 95% CI | |  | β | 95% CI | | β_diff_^a^ | 95% CI | | *t*^b^ | *p* |
|  |  | LL | UL |  |  | LL | UL |  | LL | UL |  |  |
| Direct effects |  |  |  |  |  |  |  |  |  |  |  |  |
| NSAT→AM | .575^***^ | .538 | .612 |  | .692^***^ | .653 | .731 | -.117 | -.171 | -.063 | -4.239 | .000 |
| NSAT→CM | .170^***^ | .121 | .219 |  | .308^***^ | .257 | .360 | -.138 | -.209 | -.067 | -3.813 | .000 |
| NSUP→NSAT | .720^***^ | .675 | .765 |  | .824^***^ | .777 | .870 | -.104 | -.168 | -.039 | -3.142 | .002 |
| AM→AD | .355^***^ | .312 | .398 |  | .378^***^ | .314 | .442 | -.023 | -.100 | .054 | -0.589 | .556 |
| CM→AD | .038 | -.002 | .078 |  | .042 | -.008 | .093 | -.004 | -.069 | .061 | -0.123 | .902 |
| NSUP→AD | - | - | - |  | .477^***^ | .419 | .534 | -.038 | -.109 | .034 | -1.028 | .304 |
| AM→MAL | -.162^***^ | -.216 | -.108 |  | -.169^***^ | -.252 | -.085 | .007 | -.093 | .107 | 0.129 | .898 |
| CM→MAL | .282^***^ | .226 | .339 |  | .444^***^ | .375 | .512 | -.161 | -.250 | -.073 | -3.561 | .000 |
| NSUP→MAL | - | - | - |  | -.068 | -.151 | .015 | -.094 | -.196 | .009 | -1.793 | .073 |
|  |  |  |  |  |  |  |  |  |  |  |  |  |
| Indirect effects |  |  |  |  |  |  |  |  |  |  |  |  |
| NSUP→NSAT→AM→AD | .147^***^ | .127 | .166 |  | .215^***^ | .179 | .252 | -.068 | -.110 | -.027 | -3.231 | .001 |
| NSUP→NSAT→CM→AD | .005^*^ | .000 | .009 |  | .011 | -.002 | .023 | -.006 | -.019 | .007 | -0.899 | .369 |
| NSUP→NSAT→AM→MAL | -.067^***^ | -.090 | -.044 |  | -.096^***^ | -.145 | -.047 | .029 | -.025 | .083 | 1.060 | .289 |
| NSUP→NSAT→CM→MAL | .035^***^ | .021 | .048 |  | .113^***^ | .083 | .142 | -.078 | -.110 | -.046 | -4.752 | .000 |
|  |  |  |  |  |  |  |  |  |  |  |  |  |
| Sums of indirect effects |  |  |  |  |  |  |  |  |  |  |  |  |
| NSUP→NSAT→AM/CM→AD^a^ | .152^***^ | .133 | .171 |  | .226^***^ | .193 | .259 | -.075 | -.112 | -.037 | -3.867 | .000 |
| NSUP→NSAT→AM/CM→MAL^b^ | -.032^**^ | -.056 | -.009 |  | .017 | -.033 | .066 | -.049 | -.104 | .006 | -1.746 | .081 |
|  |  |  |  |  |  |  |  |  |  |  |  |  |
| Total Effects |  |  |  |  |  |  |  |  |  |  |  |  |
| NSUP→AD^c^ | .591^***^ | .558 | .623 |  | .703^***^ | .669 | .736 | -.112 | -.159 | -.065 | -4.705 | .000 |
| NSUP→MAL^d^ | -.194^***^ | -.239 | -.149 |  | -.051^*^ | -.096 | -.006 | -.143 | -.207 | -.079 | -4.369 | .000 |
|  |  |  |  |  |  |  |  |  |  |  |  |  |
| Correlations |  |  |  |  |  |  |  |  |  |  |  |  |
| AM↔CM | .210^***^ | .168 | .251 |  | .230^***^ | .181 | .278 | -.020 | -.084 | .044 | -0.623 | .534 |
| AD↔MAL | -.075^***^ | -.115 | -.034 |  | -.060^**^ | -.105 | -.016 | -.014 | -.074 | .046 | -0.469 | .639 |

*Note*. Model parameters are adjusted for the following covariates: age, sex, study quality, and study design. ^a^Sum of indirect effects of need support on adaptive workplace outcomes through need support and all motivational forms; ^b^Sum of indirect effects of need support on maladaptive workplace outcomes through need support and all motivational forms; ^c^Total effect of need support on adaptive workplace outcomes; ^d^Total effect of need support on maladaptive workplace outcomes. β = Standardized parameter estimate; 95% CI = 95% confidence interval; LL = Lower limit of 95% CI; UL = Upper limit of 95% CI; β_diff_ = Difference in standardized path coefficient; *t* = Test of difference in standardized path coefficient based on confidence interval about the mean difference (Schenker & Gentleman, 2001); AM= Autonomous motivation; CM = Controlled motivation; NSUP = Need support; AD = Adaptive workplace outcomes collapsed across job performance, job satisfaction, work engagement, and well-being outcome variables; MAL = Maladaptive workplace outcomes collapsed across turnover and burnout outcomes; NSAT = Need satisfaction collapsed across autonomy, competence, and relatedness constructs.

^***^*p* < .001 ^**^*p* < .01 ^*^*p* < .05

**Table M10**

*Standardized Parameter Estimates for the Direct and Indirect Effects of Self-Determination Theory Constructs on Workplace Outcomes from the Multilevel Meta-Analytic Structural Equation Model of the Truncated Model in Study Samples from Countries Classified as Individualist in Cultural Orientation and Study Samples from Countries Classified as Collectivist in Cultural Orientation with Comparisons*

| Effect | Comparison group: Individualist | | |  | Comparison group: Collectivist | | | Model comparisons | | | | |
| --- | --- | --- | --- | --- | --- | --- | --- | --- | --- | --- | --- | --- |
|  | β | 95% CI | |  | β | 95% CI | | β_diff_^a^ | 95% CI | | *t*^b^ | *p* |
|  |  | LL | UL |  |  | LL | UL |  | LL | UL |  |  |
| Direct effects |  |  |  |  |  |  |  |  |  |  |  |  |
| NSAT→AM | .605^***^ | .564 | .646 |  | .429^***^ | .352 | .505 | .176 | .089 | .263 | 3.974 | .000 |
| NSAT→CM | .136^***^ | .081 | .191 |  | .138^**^ | .044 | .233 | -.002 | -.111 | .107 | -0.036 | .971 |
| NSUP→NSAT | .721^***^ | .674 | .769 |  | .605^***^ | .496 | .713 | .116 | -.002 | .234 | 1.920 | .055 |
| AM→AD | .373^***^ | .320 | .425 |  | .298^***^ | .236 | .360 | .075 | -.007 | .157 | 1.809 | .070 |
| CM→AD | .031 | -.016 | .078 |  | .009 | -.060 | .077 | .022 | -.061 | .105 | 0.519 | .604 |
| NSUP→AD | .457^***^ | .406 | .509 |  | .336^***^ | .260 | .412 | .121 | .029 | .213 | 2.583 | .010 |
| AM→MAL | -.141^***^ | -.203 | -.079 |  | -.237^***^ | -.329 | -.144 | .096 | -.015 | .207 | 1.690 | .091 |
| CM→MAL | .291^***^ | .232 | .350 |  | .125 | -.003 | .254 | .166 | .026 | .306 | 2.301 | .021 |
| NSUP→MAL | -.169^***^ | -.238 | -.100 |  | -.142^*^ | -.252 | -.031 | -.027 | -.156 | .102 | -0.406 | .685 |
|  |  |  |  |  |  |  |  |  |  |  |  |  |
| Indirect effects |  |  |  |  |  |  |  |  |  |  |  |  |
| NSUP→NSAT→AM→AD | .163^***^ | .139 | .187 |  | .077^***^ | .054 | .101 | .086 | .053 | .119 | 5.018 | .000 |
| NSUP→NSAT→CM→AD | .003 | -.001 | .008 |  | .001 | -.005 | .006 | .002 | -.005 | .009 | 0.552 | .581 |
| NSUP→NSAT→AM→MAL | -.062^***^ | -.089 | -.034 |  | -.061^***^ | .014 | -.088 | -.001 | -.040 | .038 | -0.010 | .992 |
| NSUP→NSAT→CM→MAL | .029^***^ | .014 | .043 |  | .010 | .007 | -.004 | .019 | .000 | .038 | 0.638 | .523 |
|  |  |  |  |  |  |  |  |  |  |  |  |  |
| Sums of indirect effects |  |  |  |  |  |  |  |  |  |  |  |  |
| NSUP→NSAT→AM/CM→AD^a^ | .166^***^ | .142 | .189 |  | .078^***^ | .054 | .102 | .088 | .055 | .121 | 2.952 | .003 |
| NSUP→NSAT→AM/CM→MAL^b^ | -.033^*^ | -.062 | -.004 |  | -.051^***^ | -.076 | -.025 | .018 | -.019 | .055 | 1.144 | .253 |
|  |  |  |  |  |  |  |  |  |  |  |  |  |
| Total Effects |  |  |  |  |  |  |  |  |  |  |  |  |
| NSUP→AD^c^ | .623^***^ | .587 | .659 |  | .414^***^ | .347 | .481 | .209 | .134 | .284 | 1.892 | .059 |
| NSUP→MAL^d^ | -.202^***^ | -.252 | -.153 |  | -.193^***^ | -.291 | -.094 | -.009 | -.119 | .101 | -0.351 | .726 |
|  |  |  |  |  |  |  |  |  |  |  |  |  |
| Correlations |  |  |  |  |  |  |  |  |  |  |  |  |
| AM↔CM | .219^***^ | .179 | .267 |  | .157^***^ | .084 | .230 | .062 | -0.026 | .150 | 1.387 | .166 |
| AD↔MAL | -.065^**^ | -.113 | -.017 |  | -.162^***^ | -.235 | -.090 | .097 | .009 | .185 | 2.187 | .029 |

*Note*. Model parameters are adjusted for the following covariates: age, sex, study quality, and study design. ^a^Sum of indirect effects of need support on adaptive workplace outcomes through need support and all motivational forms; ^b^Sum of indirect effects of need support on maladaptive workplace outcomes through need support and all motivational forms; ^c^Total effect of need support on adaptive workplace outcomes; ^d^Total effect of need support on maladaptive workplace outcomes. β = Standardized parameter estimate; 95% CI = 95% confidence interval; LL = Lower limit of 95% CI; UL = Upper limit of 95% CI; β_diff_ = Difference in standardized path coefficient; *t* = Test of difference in standardized path coefficient based on confidence interval about the mean difference (Schenker & Gentleman, 2001); AM= Autonomous motivation; CM = Controlled motivation; NSUP = Need support; AD = Adaptive workplace outcomes collapsed across job performance, job satisfaction, work engagement, and well-being outcome variables; MAL = Maladaptive workplace outcomes collapsed across turnover and burnout outcomes; NSAT = Need satisfaction collapsed across autonomy, competence, and relatedness constructs.

^***^*p* < .001 ^**^*p* < .01 ^*^*p* < .05

**Table M11**

*Standardized Parameter Estimates for the Direct and Indirect Effects of Self-Determination Theory Constructs on Workplace Outcomes from the Multilevel Meta-Analytic Structural Equation Model of the Truncated Model in Studies on Organizations with Leaders Offering Proximal Autonomy Support and Studies on Organizations with Leaders Offering Indeterminate Autonomy Support*

| Effect | Comparison group: Leadership Autonomy Support Proximity: Proximal | | |  | Comparison group: Leadership Autonomy Support Proximity: Indeterminate | | | Model Comparisons | | | | |
| --- | --- | --- | --- | --- | --- | --- | --- | --- | --- | --- | --- | --- |
|  | β | 95% CI | |  | β | 95% CI | | β_diff_ | 95% CI | | *t*^b^ | *p* |
|  |  | LL | UL |  |  | LL | UL |  | LL | UL |  |  |
| Direct effects |  |  |  |  |  |  |  |  |  |  |  |  |
| NSAT→AM | .366^***^ | .299 | .432 |  | .476^***^ | .363 | .589 | -.110 | -1.249 | 1.029 | -1.644 | .100 |
| NSAT→CM | -.172^***^ | -.257 | -.087 |  | .059 | -.081 | .199 | -.231 | -.395 | -.067 | -2.764 | .006 |
| NSUP→NSAT | .411^***^ | .343 | .478 |  | .489^***^ | .385 | .592 | -.078 | -.202 | .046 | -1.237 | .216 |
| AM→AD | .309^***^ | .248 | .371 |  | .323^***^ | .214 | .431 | -.014 | -.139 | .111 | -0.220 | .826 |
| CM→AD | -.140^***^ | -.213 | -.067 |  | -.005 | -.137 | .127 | -.135 | -.285 | .015 | -1.754 | .079 |
| NSUP→AD | .246^***^ | .184 | .309 |  | .246^***^ | .152 | .340 | .000 | -.113 | .113 | <0.001 | >.999 |
| AM→MAL | -.500^***^ | -.606 | -.394 |  | -.307^***^ | -.415 | -.199 | -.193 | -.344 | -.042 | -2.500 | .012 |
| CM→MAL | -.006 | -.132 | .121 |  | .176^**^ | .026 | .326 | -.182 | -.380 | .016 | -1.818 | .069 |
| NSUP→MAL | -.387^***^ | -.474 | .300 |  | -.238^***^ | -.360 | -.116 | -.149 | -.298 | .000 | -0.720 | .472 |
|  |  |  |  |  |  |  |  |  |  |  |  |  |
| Indirect effects |  |  |  |  |  |  |  |  |  |  |  |  |
| NSUP→NSAT→AM→AD | .046^***^ | .030 | .063 |  | .075^***^ | .034 | .116 | -.029 | -.074 | .016 | -0.608 | .543 |
| NSUP→NSAT→CM→AD | .010^*^ | .002 | .018 |  | .000 | -.004 | .004 | .010 | .001 | .019 | 2.191 | .028 |
| NSUP→NSAT→AM→MAL | -.075^***^ | -.094 | -.056 |  | -.072^***^ | -.099 | -.044 | -.003 | -.035 | .029 | -0.015 | .988 |
| NSUP→NSAT→CM→MAL | .000 | -.009 | .009 |  | .005 | -.010 | .020 | -.005 | -.121 | .111 | -0.195 | .846 |
|  |  |  |  |  |  |  |  |  |  |  |  |  |
| Sums of indirect effects |  |  |  |  |  |  |  |  |  |  |  |  |
| NSUP→NSAT→AM/CM→AD^a^ | .056^***^ | .041 | .072 |  | .075^***^ | .034 | .116 | -.019 | -.051 | .013 | -1.614 | .107 |
| NSUP→NSAT→AM/CM→MAL^b^ | -.075^***^ | -.096 | -.054 |  | -.066^***^ | -.092 | -.041 | -.009 | -.042 | .024 | -0.532 | .593 |
|  |  |  |  |  |  |  |  |  |  |  |  |  |
| Total Effects |  |  |  |  |  |  |  |  |  |  |  |  |
| NSUP→AD^c^ | .303^***^ | .240 | .366 |  | .321^***^ | .216 | .425 | -.018 | -.129 | .093 | -0.289 | .772 |
| NSUP→MAL^d^ | -.461^***^ | -.539 | -.384 |  | -.305^***^ | .216 | .425 | -.156 | -.296 | -.016 | -2.185 | .029 |
|  |  |  |  |  |  |  |  |  |  |  |  |  |
| Correlations |  |  |  |  |  |  |  |  |  |  |  |  |
| AM↔CM | .103^*^ | .023 | .184 |  | .143^*^ | .030 | .256 | -.040 | -.179 | .099 | -0.565 | .572 |
| AD↔MAL | -.257^***^ | -.349 | -.165 |  | -.032 | -.189 | .125 | -.225 | -.407 | -.043 | -2.423 | .015 |

*Note*. Model parameters are adjusted for the following covariates: age, sex, study quality, and study design. ^a^Sum of indirect effects of need support on adaptive workplace outcomes through need support and all motivational forms; ^b^Sum of indirect effects of need support on maladaptive workplace outcomes through need support and all motivational forms; ^c^Total effect of need support on adaptive workplace outcomes; ^d^Total effect of need support on maladaptive workplace outcomes. β = Standardized parameter estimate; 95% CI = 95% confidence interval; LL = Lower limit of 95% CI; UL = Upper limit of 95% CI; β_diff_ = Difference in standardized path coefficient; *t* = Test of difference in standardized path coefficient based on confidence interval about the mean difference (Schenker & Gentleman, 2001); AM= Autonomous motivation; CM = Controlled motivation; NSUP = Need support; AD = Adaptive workplace outcomes collapsed across job performance, job satisfaction, work engagement, and well-being outcome variables; MAL = Maladaptive workplace outcomes collapsed across turnover and burnout outcomes; NSAT = Need satisfaction collapsed across autonomy, competence, and relatedness constructs.

^***^*p* < .001 ^**^*p* < .01 ^*^*p* < .05

Supplement N

**Table N1**

*Publication Bias Statistics for Meta-Analysis of Relations Among Constructs from Self-Determination Theory and Workplace Outcomes*

| Effect | τ | Trim and fill | | | |  | Regression tests | | |  | *p*-curve^b^ | |  | *p*-uniform* | | | | |  | 3PSM | | | |
| --- | --- | --- | --- | --- | --- | --- | --- | --- | --- | --- | --- | --- | --- | --- | --- | --- | --- | --- | --- | --- | --- | --- | --- |
|  |  | *r*^+^ | 95% CI | | *k*0 |  | *z* | *r*^+^_PET_ | *r*^+^_PEESE_ |  | *z* (right skewness) | *z*^a^ |  | *r*^+^ | 95% CI | | τ^2^ | χ^2^*_p_*_-uni_ |  | *r*^+^ | 95% CI | | χ^2^_3PSM_^a^ |
|  |  |  | LB | UB |  |  |  |  |  |  |  |  |  |  | LB | UB |  |  |  |  | LB | UB |  |
| IM-ER | 0.037 | .131^***^ | .057 | .204 | 0 |  | 0.938 | 0.045 | 0.079 |  | -31.107^***^ | 27.139 |  | .327^***^ | 0.165 | 0.493 | .129 | 4.055 |  | .220^***^ | .101 | .339 | 3.461 |
| IM-AUT | -0.024 | .406^***^ | .343 | .469 | 0 |  | -0.199 | 0.424^***^ | 0.419^***^ |  | -32.410^***^ | 30.241 |  | .442^**^ | 0.279 | 0.538 | .046 | 0.028 |  | .414^***^ | .346 | .482 | 0.208 |
| IM-COM | -0.152 | .486^***^ | .400 | .572 | 3 |  | -1.106 | 0.610^***^ | 0.536^***^ |  | -20.826^***^ | 19.155 |  | .391 | - | 0.605 | .088 | 0.784 |  | .441^***^ | .355 | .528 | 0.729 |
| IM-REL | 0.033 | .384^***^ | .313 | .456 | 0 |  | 0.179 | 0.353 | 0.376^***^ |  | -24.099^***^ | 22.457 |  | .392 | -0.009 | 0.500 | .032 | 0.233 |  | .385^***^ | .317 | .434 | 0.625 |
| IM-NSUP | 0.033 | .316^***^ | .263 | .369 | 6 |  | 0.516 | 0.322^***^ | 0.342^***^ |  | -29.406^***^ | 27.044 |  | .393^***^ | 0.322 | 0.451 | .014 | 0.212 |  | .366^***^ | .317 | .416 | 0.172 |
| IM-IG | 0.333 | .591^***^ | .549 | .633 | 1 |  | 0.218 | 0.583^***^ | 0.588^***^ |  | -13.433^***^ | 14.074 |  | .691^***^ | 0.630 | 0.759 | .000 | 0.973 |  | - | - | - | - |
| IM-IJ | 0.033 | .233^***^ | .120 | .347 | 1 |  | -0.581 | 0.334 | 0.302^**^ |  | -18.634^***^ | 17.877 |  | .336^**^ | 0.167 | 0.486 | .032 | 2.164 |  | .307^***^ | .170 | .444 | 2.486 |
| IM-ID | 0.059 | .564^***^ | .457 | .672 | 0 |  | -0.253 | 0.615^**^ | 0.590^***^ |  | -31.212 | 32.145 |  | .713^***^ | 0.419 | 0.887 | .112 | 0.078 |  | .589^***^ | .490 | .687 | 1.120 |
| IM-JP | -0.027 | .339^***^ | .292 | .385 | 6 |  | -0.102 | 0.309^***^ | 0.311^***^ |  | -26.009^***^ | 22.525 |  | .174 | -0.371 | 0.315 | .048 | 5.081 |  | .222^**^ | .082 | .361 | 7.308^**^ |
| IM-JS | -0.102 | .402^***^ | .316 | .488 | 0 |  | -0.187 | 0.430^**^ | 0.435^***^ |  | -28.365^***^ | 27.068 |  | .448^**^ | 0.209 | 0.580 | .073 | 0.008 |  | .409^***^ | .299 | .519 | 0.033 |
| IM-TU | -0.031 | -.322^***^ | -.387 | -.257 | 0 |  | -0.063 | -0.316^**^ | -0.323^***^ |  | -33.273^***^ | 30.468 |  | -.314^*^ | -0.417 | -0.120 | .080 | 0.863 |  | -.308^***^ | -.384 | -.233 | 1.577 |
| IM-BU | 0.128 | -.320^***^ | -.381 | -.258 | 0 |  | 0.704 | -0.363^***^ | -0.323^***^ |  | -27.485^***^ | 25.691 |  | -.334^***^ | -0.415 | -0.229 | .038 | 0.086 |  | -.317^***^ | -.380 | -.254 | 0.271 |
| IM-WE | 0.056 | .494^***^ | .426 | .561 | 0 |  | 1.164 | 0.396^***^ | 0.443^***^ |  | -39.990^***^ | 39.304 |  | .477 | -0.030 | 0.630 | .132 | 2.297 |  | .458^***^ | .403 | .513 | 4.037 |
| IM-WB | -0.071 | .469^***^ | .331 | .607 | 0 |  | -0.358 | 0.574 | 0.528^**^ |  | -18.860^***^ | 17.763 |  | .436 | - | 0.729 | .121 | 0.402 |  | .448^***^ | .338 | .557 | 0.557 |
| ER-AUT | 0.243 | -.075^*^ | -.149 | -.001 | 3 |  | 1.553 | -0.173 | -0.102 |  | - | - |  | -.060 | -0.117 | 0.041 | .006 | - |  | .015 | -.138 | .167 | 0.906 |
| ER-COM | 0.200 | -.010 | -.186 | .166 | 0 |  | 0.138 | -0.079 | -0.010 |  | -3.529^***^ | 2.116 |  | .023 | -0.223 | 0.716 | .033 | 0.130 |  | .013 | -.267 | .293 | 0.048 |
| ER-REL | -0.200 | -.076 | -.180 | .030 | 0 |  | 0.356 | -0.177 | -0.134 |  | -4.569^***^ | 3.071 |  | .026 | -0.168 | - | .030 | 0.631 |  | .019 | -.397 | .436 | 1.712 |
| ER-NSUP | 0.018 | .036 | -.079 | .150 | 0 |  | -0.091 | 0.053 | 0.035 |  | -9.406^***^ | 7.589 |  | .035 | -0.143 | 0.270 | .034 | 0.038 |  | .031 | -.145 | .207 | 0.005 |
| ER-IG | 1.000 | -.040 | -.160 | .080 | 2 |  | 2.734^**^ | -0.162^**^ | -0.079^*^ |  | - | - |  | -.022 | -0.086 | 0.181 | .000 | 1.802 |  | - | - | - | - |
| ER-IJ | -0.099 | .296^***^ | .210 | .382 | 0 |  | -0.013 | 0.298 | 0.276^**^ |  | -20.201^***^ | 18.185 |  | .322^*^ | 0.105 | 0.432 | .026 | 0.074 |  | .306^***^ | .201 | .412 | 0.078 |
| ER-ID | -0.017 | .110 | -.002 | .221 | 0 |  | -0.645 | 0.239 | 0.204 |  | -12.734^***^ | 10.074 |  | .063 | -0.152 | 0.257 | .053 | 0.307 |  | .053 | -.128 | .234 | 0.613 |
| ER-JP | 0.010 | .064 | -.033 | .162 | 0 |  | -0.018 | 0.067 | 0.070 |  | -7.791^***^ | 5.473 |  | .048 | -0.103 | 0.226 | .030 | 0.180 |  | .036 | -.109 | .180 | 0.232 |
| ER-JS | -0.091 | .090 | -.046 | .226 | 3 |  | -1.212 | 0.243 | 0.105 |  | -12.500^***^ | 11.327 |  | .237 | -0.030 | 0.767 | .073 | 2.702 |  | .205 | -.117 | .528 | 3.838^*^ |
| ER-TU | -0.294 | -.008 | -.106 | .089 | 0 |  | -2.719 | 0.342^*^ | 0.198^*^ |  | -7.452^***^ | 5.723 |  | -.012 | -0.151 | 0.204 | .024 | 0.023 |  | -.016 | -.155 | .124 | 0.020 |
| ER-BU | 0.295 | .109^***^ | .052 | .165 | 2 |  | 2.571^*^ | -0.022 | 0.062 |  | -11.874^***^ | 9.182 |  | .155^**^ | 0.069 | 0.226 | .006 | 1.016 |  | .128^**^ | .048 | .209 | 0.001 |
| ER-WE | 0.186 | .063 | -.054 | .180 | 0 |  | 2.943^**^ | -0.272^*^ | -0.117 |  | - | - |  | - | - | - | - | - |  | .171 | -.036 | .377 | 1.819 |
| ER-WB | -0.200 | -.083 | -.230 | .065 | 0 |  | -1.130 | 0.289 | 0.107 |  | -7.762^***^ | 5.767 |  | -.091 | -0.275 | 0.443 | .028 | 0.086 |  | -.090 | -.278 | .097 | 0.013 |
| AUT-COM | -0.091 | .582^***^ | .521 | .643 | 6 |  | -0.413 | 0.565^***^ | 0.564^***^ |  | -41.141 | 41.073 |  | .581^*^ | 0.289 | 0.712 | .111 | 0.956 |  | .530^***^ | .481 | .579 | 0.685 |
| AUT-REL | -0.047 | .530^***^ | .480 | .581 | 4 |  | -0.011 | 0.505^***^ | 0.513^***^ |  | -44.931^***^ | 44.198 |  | .561^***^ | 0.444 | 0.644 | .058 | 0.366 |  | .501^***^ | .459 | .542 | 0.751 |
| AUT-NSUP | 0.077 | .467^***^ | .367 | .566 | 0 |  | 0.193 | 0.438^**^ | 0.431^***^ |  | -25.526 | 25.127 |  | .471 | -1.202 | 0.654 | .089 | 0.503 |  | .448^***^ | .367 | .528 | 0.967 |
| AUT-IJ | <0.001 | -.006 | -.184 | .172 | 0 |  | -0.549 | 0.168 | 0.098 |  | -1.637^*^ | 0.420 |  | -.029 | -0.253 | 0.449 | .016 | 0.380 |  | -.028 | -.229 | .174 | 0.083 |
| AUT-ID | -0.238 | .367^***^ | .259 | .474 | 0 |  | -0.313 | 0.436 | 0.404^**^ |  | -14.712^***^ | 13.109 |  | .363 | - | 0.511 | .027 | 0.142 |  | .346^***^ | .265 | .427 | 0.723 |
| AUT-JP | -0.048 | .288^***^ | .218 | .359 | 0 |  | -0.374 | 0.329^**^ | 0.309^***^ |  | -18.785^***^ | 16.502 |  | .337^***^ | 0.230 | 0.419 | .014 | 0.880 |  | .322^***^ | .256 | .388 | 2.196 |
| AUT-JS | <0.001 | .349^***^ | .285 | .412 | 10 |  | 0.453 | 0.394^***^ | 0.418^***^ |  | -39.455^***^ | 38.075 |  | .46^***^ | 0.328 | 0.543 | .043 | 0.077 |  | .422^***^ | .353 | .491 | 0.075 |
| AUT-TU | 0.024 | -.389^***^ | -.464 | -.313 | 0 |  | 0.091 | -0.399^**^ | -0.383^***^ |  | -26.258^***^ | 24.258 |  | -.423^***^ | -0.519 | -0.312 | .046 | 0.012 |  | -.387^***^ | -.463 | -.311 | 0.121 |
| AUT-BU | 0.111 | -.476^***^ | -.551 | -.400 | 0 |  | 0.064 | -0.481^***^ | -0.478^***^ |  | -27.603^***^ | 26.371 |  | -.538^***^ | -0.649 | -0.419 | .045 | <0.001 |  | - | - | - | - |
| AUT-WE | -0.149 | .463^***^ | .413 | .514 | 0 |  | -1.211 | 0.544^***^ | 0.505^***^ |  | -39.459^***^ | 38.035 |  | .512^***^ | 0.403 | 0.590 | .045 | 0.077 |  | .468^***^ | .418 | .519 | 0.312 |
| AUT-WB | -0.286 | .495^***^ | .398 | .592 | 1 |  | 0.956 | 0.376^*^ | 0.459^***^ |  | -21.072 | 21.587 |  | .586^**^ | 0.402 | 0.725 | .029 | 0.022 |  | .515^***^ | .431 | .599 | 0.039 |
| COM-REL | -0.057 | .481^***^ | .434 | .528 | 0 |  | -0.207 | 0.493^***^ | 0.491^***^ |  | -39.104^***^ | 38.784 |  | .533^***^ | 0.455 | 0.599 | .030 | 0.038 |  | .479^***^ | .439 | .519 | 0.431 |
| COM-NSUP | -0.333 | .467^***^ | .287 | .647 | 0 |  | -0.822 | 0.628^**^ | 0.555^***^ |  | -13.401^***^ | 13.439 |  | .444 | - | 0.752 | .101 | 0.241 |  | .417^***^ | .283 | .551 | 0.826 |
| COM-ID | 1.000 | .475^***^ | .383 | .567 | 0 |  | 1.590 | -0.028 | 0.235 |  | -13.433^***^ | 14.074 |  | .520^*^ | 0.378 | 0.660 | .004 | 0.194 |  | .473^***^ | .396 | .550 | <0.001 |
| COM-JP | -0.143 | .432^***^ | .328 | .536 | 0 |  | -0.638 | 0.545^**^ | 0.516^***^ |  | -16.427^***^ | 16.390 |  | .461 | -0.504 | 0.607 | .026 | 0.030 |  | .428^***^ | .346 | .509 | 0.192 |
| COM-JS | 0.219 | .455^***^ | .367 | .542 | 0 |  | 0.599 | 0.396^**^ | 0.414^***^ |  | -28.355^***^ | 27.174 |  | .481^*^ | 0.059 | 0.617 | .057 | 0.280 |  | .442^***^ | .371 | .513 | 0.815 |
| COM-TU | 0.111 | -.368^***^ | -.467 | -.269 | 2 |  | 1.074 | -0.475^**^ | -0.412^***^ |  | -15.952^***^ | 14.946 |  | -.341^**^ | -0.450 | -0.209 | .019 | 0.051 |  | -.324^***^ | -.413 | -.235 | 0.008 |
| COM-BU | -0.200 | -.347^***^ | -.454 | -.239 | 1 |  | -1.075 | -0.272^*^ | -0.317^***^ |  | -15.477^***^ | 14.590 |  | -.403^*^ | -0.553 | -0.243 | .018 | 0.177 |  | -.370^***^ | -.465 | -.275 | 0.001 |
| COM-WE | -0.118 | .551^***^ | .467 | .637 | 1 |  | -0.502 | 0.600^***^ | 0.577^***^ |  | -29.358^***^ | 29.523 |  | .602^*^ | 0.260 | 0.750 | .079 | 0.151 |  | .529^***^ | .460 | .599 | 0.218 |
| COM-WB | 0.333 | .561^***^ | .448 | .675 | 0 |  | 0.713 | 0.455^**^ | 0.516^***^ |  | -15.511^***^ | 15.886 |  | .653^*^ | 0.323 | 0.864 | .024 | 0.145 |  | .559^***^ | .465 | .653 | <0.001 |
| REL-NSUP | -0.422 | .496^***^ | .398 | .595 | 2 |  | -2.495^*^ | 0.792^***^ | 0.596^***^ |  | -22.447^***^ | 21.302 |  | .477 | -1.243 | 0.655 | .064 | 0.161 |  | .451^***^ | .369 | .532 | 0.225 |
| REL-ID | <0.001 | .323^***^ | .207 | .439 | 0 |  | -0.294 | 0.485 | 0.414 |  | -11.595^***^ | 10.652 |  | .330 | - | 0.484 | .013 | 0.048 |  | .318^***^ | .231 | .404 | 0.148 |
| REL-JP | 0.067 | .316^***^ | .194 | .438 | 0 |  | -0.458 | 0.421 | 0.402 |  | -15.204^***^ | 13.962 |  | .368^*^ | 0.102 | 0.512 | .016 | 0.495 |  | .354^***^ | .259 | .450 | 1.499 |
| REL-JS | -0.177 | .396^***^ | .344 | .448 | 3 |  | 0.546 | 0.386^***^ | 0.399^***^ |  | -27.269^***^ | 25.152 |  | .448^***^ | 0.373 | 0.514 | .013 | 0.071 |  | .414^***^ | .370 | .458 | 0.510 |
| REL-TU | -0.152 | -.420^***^ | -.554 | -.285 | 0 |  | -0.074 | -0.406^*^ | -0.421^***^ |  | -21.044^***^ | 20.364 |  | -.449 | -0.656 | 0.343 | .107 | 0.127 |  | - | - | - | - |
| REL-BU | 0.026 | -.211^***^ | -.312 | -.109 | 4 |  | -0.852 | -0.193 | -0.227^**^ |  | -18.316^***^ | 16.176 |  | -.335^***^ | -0.427 | -0.242 | .018 | 1.965 |  | -.316^***^ | -.394 | -.237 | 3.581 |
| REL-WE | -0.063 | .442^***^ | .369 | .515 | 0 |  | -0.204 | 0.466^**^ | 0.445^***^ |  | -28.764^***^ | 28.331 |  | .459^*^ | 0.106 | 0.577 | .055 | 0.390 |  | .429^***^ | .368 | .490 | 1.054 |
| REL-WB | -0.333 | .465^***^ | .423 | .507 | 1 |  | -0.540 | 0.493^***^ | 0.484^***^ |  | -14.109^***^ | 13.948 |  | .492^**^ | 0.425 | 0.553 | .000 | 1.052 |  | - | - | - | - |
| NS-IJ | 0.467 | .156 | -.009 | .321 | 0 |  | 2.337^*^ | -0.474 | -0.192 |  | -9.175^***^ | 7.399 |  | .279^*^ | 0.013 | 0.544 | .028 | 1.239 |  | .244^*^ | .021 | .467 | 0.932 |
| NS-ID | 1.000 | .297^***^ | .164 | .429 | 0 |  | 2.846^**^ | -0.241 | 0.001 |  | -12.603^***^ | 11.225 |  | -.047 | - | 0.429 | .111 | 1.204 |  | .145^*^ | .043 | .259 | 2.235 |
| NS-JP | 0.276 | .240^***^ | .162 | .317 | 0 |  | 2.191^*^ | 0.049 | 0.140^*^ |  | -14.35^***^ | 11.84 |  | .184 | -0.173 | 0.327 | .033 | 0.836 |  | .169^*^ | .002 | .336 | 2.012 |
| NS-JS | 0.105 | .365^***^ | .260 | .469 | 2 |  | 0.218 | 0.358 | 0.375^**^ |  | -23.448^***^ | 22.581 |  | .418 | -0.037 | 0.570 | .059 | 0.089 |  | .382^***^ | .230 | .534 | 0.166 |
| NS-TU | -0.100 | -.347^***^ | -.429 | -.264 | 1 |  | -0.247 | -0.328^*^ | -0.349^***^ |  | -24.802^***^ | 23.886 |  | -.387^**^ | -0.487 | -0.271 | .033 | 0.021 |  | -.359^***^ | -.441 | -.278 | 0.063 |
| NS-BU | -0.018 | -.387^***^ | -.462 | -.311 | 2 |  | 0.428 | -0.409^**^ | -0.383^***^ |  | -19.264^***^ | 17.020 |  | -.376^***^ | -0.463 | -0.286 | .015 | 0.009 |  | -.355^***^ | -.424 | -.286 | <0.001 |
| NS-WE | 0.030 | .441^***^ | .363 | .520 | 1 |  | -0.721 | 0.531^**^ | 0.463^***^ |  | -23.958^***^ | 22.717 |  | .455^*^ | 0.194 | 0.567 | .031 | 0.063 |  | .422^***^ | .357 | .487 | 0.284 |
| NS-WB | 0.286 | .434^***^ | .388 | .480 | 3 |  | 2.377^*^ | 0.365^***^ | 0.409^***^ |  | -21.377^***^ | 21.281 |  | .506^***^ | 0.444 | 0.585 | .004 | 0.243 |  | - | - | - | - |
| IG-ID | 0.333 | .500^***^ | .461 | .539 | 2 |  | 0.933 | 0.463^***^ | 0.494^***^ |  | -13.433^***^ | 14.074 |  | .574^**^ | 0.518 | 0.665 | .000 | 0.170 |  | - | - | - | - |
| IJ-ID | 0.011 | .332^***^ | .229 | .435 | 0 |  | -0.509 | 0.435^*^ | 0.403^**^ |  | -22.138^***^ | 20.892 |  | .363^*^ | 0.061 | 0.506 | .046 | 0.011 |  | .352^***^ | .232 | .473 | 0.191 |
| IJ-JP | 0.600 | .065 | -.099 | .229 | 0 |  | 1.031 | -0.428 | -0.216 |  | -2.491^**^ | 0.942 |  | -.077 | -0.353 | 0.157 | .017 | 2.040 |  | -.081 | -.283 | .120 | 2.745 |
| IJ-JS | 0.333 | .138 | -.085 | .362 | 0 |  | 0.222 | -0.007 | 0.099 |  | - | - |  | .202 | -0.236 | 0.632 | .041 | 0.252 |  | .410^*^ | .054 | .767 | 4.241^*^ |
| IJ-TU | 1.000 | -.031 | -.148 | .086 | 0 |  | 2.605^**^ | -0.763^**^ | -0.405^**^ |  | - | - |  | .016 | -0.157 | - | .011 | 0.191 |  | - | - | - | - |
| IJ-BU | 0.333 | -.152^***^ | -.239 | -.064 | 0 |  | -0.136 | 0.004 | -0.405^**^ |  | - | - |  | -.154 | -0.258 | 0.096 | .003 | 0.176 |  | -.152^***^ | -.225 | -.079 | 0.003 |
| IJ-WE | -0.067 | .096 | -.075 | .267 | 1 |  | -0.983 | 0.285 | 0.168 |  | -4.800^***^ | 3.994 |  | .133 | -0.154 | 0.552 | .042 | 0.357 |  | .115 | -.159 | .398 | 0.336 |
| IJ-WB | 0.200 | .167^*^ | .018 | .317 | 2 |  | -0.271 | 0.189 | 0.132 |  | - | - |  | .326^*^ | 0.064 | 0.845 | .025 | 3.236 |  | .312^*^ | .009 | .616 | 4.801^*^ |
| ID-JP | 0.333 | .202^**^ | .070 | .335 | 0 |  | 0.024 | 0.192 | 0.214 |  | -6.658^***^ | 5.598 |  | .272^*^ | <0.001 | 0.444 | .017 | 0.661 |  | .242^**^ | .078 | .405 | 0.326 |
| ID-JS | -0.200 | .488^***^ | .320 | .656 | 0 |  | -0.502 | 0.742 | 0.626^*^ |  | -14.108^***^ | 13.658 |  | .528 | - | 0.796 | .062 | 0.055 |  | .477^***^ | .348 | .606 | 0.198 |
| ID-TU | 0.667 | -.301^**^ | -.505 | -.096 | 0 |  | 1.266 | -1.422 | -0.882 |  | -12.66^***^ | 11.47 |  | -.309 | -0.565 | - | .039 | 0.059 |  | -.292^**^ | -.494 | -.091 | 0.116 |
| ID-BU | <0.001 | -.347^**^ | -.595 | -.098 | 0 |  | 0.017 | -0.364 | -0.356 |  | -13.433^***^ | 13.864 |  | -.319 | -0.708 | - | .100 | 0.109 |  | -.316^*^ | -.621 | -.011 | 0.318 |
| ID-WE | -0.167 | .505^***^ | .415 | .595 | 0 |  | -1.069 | 0.647^***^ | 0.581^***^ |  | -20.735 | 20.952 |  | .566^**^ | 0.400 | 0.687 | .026 | 0.068 |  | .505^***^ | .431 | .578 | 0.046 |
| ID-WB | -0.067 | .502^***^ | .370 | .634 | 0 |  | -0.416 | 0.640 | 0.585^**^ |  | -17.732^***^ | 17.429 |  | .563 | -0.984 | 0.773 | .048 | 0.030 |  | .500^***^ | .393 | .607 | 0.051 |
| JP-JS | 0.333 | .422^***^ | .324 | .520 | 0 |  | 0.936 | 0.179 | 0.301^*^ |  | -16.390^***^ | 14.051 |  | .443 | - | 0.604 | .030 | 0.077 |  | .415^***^ | .334 | .495 | 0.260 |
| JP-TU | -0.231 | -.172^***^ | -.262 | -.083 | 0 |  | -1.319 | -0.008 | -0.088 |  | -7.903^***^ | 6.132 |  | -.085 | -0.276 | - | .070 | 0.921 |  | -.145^*^ | -.274 | -.020 | 1.207 |
| JP-BU | <0.001 | -.335^***^ | -.511 | -.160 | 1 |  | 0.369 | -0.396 | -0.362^*^ |  | -8.308^***^ | 7.108 |  | -.280 | -0.547 | - | .060 | 0.054 |  | -.276^**^ | -.471 | -.081 | 0.185 |
| JP-WE | 0.067 | .471^***^ | .338 | .604 | 0 |  | 1.140 | 0.266 | 0.379^**^ |  | -21.635^***^ | 20.872 |  | .306 | - | 0.710 | .212 | 1.117 |  | .431^***^ | .328 | .535 | 1.202 |
| JS-TU | -0.111 | -.407^***^ | -.494 | -.319 | 3 |  | -1.160 | -0.281 | -0.350^***^ |  | -29.110^***^ | 28.822 |  | -.507^***^ | -0.620 | -0.381 | .053 | 0.009 |  | -.449^***^ | -.533 | -.365 | 0.041 |
| JS-BU | 0.238 | -.524^***^ | -.559 | -.488 | 1 |  | 0.988 | -0.551^***^ | -0.532^***^ |  | -18.008^***^ | 17.190 |  | -.566^***^ | -0.630 | -0.483 | .003 | 0.494 |  | - | - | - | - |
| JS-WE | -0.600 | .713^***^ | .624 | .801 | 3 |  | -2.328^*^ | 0.783^***^ | 0.719^***^ |  | -18.997^***^ | 19.692 |  | .772^**^ | 0.627 | 0.903 | .018 | 0.018 |  | .653^***^ | .599 | .707 | <0.001 |
| JS-WB | 0.067 | .572^***^ | .416 | .728 | 0 |  | 0.247 | 0.504 | 0.545^**^ |  | -17.534^***^ | 17.855 |  | .673 | - | 0.959 | .090 | 0.034 |  | .569^***^ | .442 | .695 | 0.070 |
| TU-BU | <0.001 | .469^***^ | .366 | .572 | 0 |  | -1.095 | 0.594^***^ | 0.562^***^ |  | -23.816^***^ | 24.162 |  | .552^**^ | 0.307 | 0.697 | .047 | 0.215 |  | .497^***^ | .412 | .582 | 1.792 |
| TU-WE | 0.382 | -.222^*^ | -.407 | -.037 | 0 |  | 2.288^*^ | -0.867^**^ | -0.597^**^ |  | -18.733^***^ | 18.319 |  | -.138 | -0.435 | 1.012 | .140 | 0.277 |  | -.163 | -.505 | .180 | 0.279 |
| BU-WE | 0.294 | -.432^***^ | -.553 | -.312 | 0 |  | 2.465^*^ | -0.689^***^ | -0.560^***^ |  | -28.120^***^ | 27.974 |  | -.533^**^ | -0.677 | -0.344 | .074 | 0.710 |  | -.470^***^ | -.570 | -.371 | 2.230 |
| WB-WE | -0.333 | .463^***^ | .418 | .507 | 0 |  | 0.032 | 0.459^***^ | 0.460^***^ |  | -13.433^***^ | 14.074 |  | .501^***^ | 0.454 | 0.548 | .000 | <0.001 |  | - | - | - | - |

*Note*. ^a^Test statistic non-significant (*p* > .05) in all cases. ^b^Power estimate (1-β) is >99% in all cases. τ = Kendall’s τ from Begg and Mazumdar’s (1994) rank correlation test; Trim and fill = Duval and Tweedie’s (2000) trim and fill analysis; *r*^+^ = Corrected meta-analytic effect size estimate from publication bias test; 95% CI = 95% confidence interval of corrected effect size estimate; *k*0 = Estimated number of ‘missing’ studies on the right-hand/left-hand side of the funnel plot from trim and fill analysis; Regression tests = Publication bias tests based on regression of study effect size on precision estimate; *z* = Funnel plot asymmetry test statistic from Sterne, Egger, and Davey Smith’s (2001) regression test; PET = Stanley and Doucouliagos’ (2014) precision effect test; PEESE = Stanley and Doucouliagos’ (2014) precision effect estimate with standard error; *p*-curve = Simonsohn, Nelson, and Simmons’ (2014) *p*-curve analysis; *z* (right skewness) = Test statistic for *p*-curve right skewness; *z* (flatness) = Test statistic for degree of *p*-curve ‘flatness’; *p*-uniform* = van Aert and van Assen’s (2018) *p*-uniform* analysis; τ^2^ = Estimate of ‘true’ variance in population from *p*-uniform* analysis; χ^2^*_p_*_-uni.*_ = Likelihood ratio test of publication bias from *p*-uniform* analysis; 3PSM = Vevea and Hedges’ (2005) three-parameter selection method analysis; χ^2^_3PSM_ = Likelihood ratio test of publication bias from 3PSM analysis; IM = Intrinsic motivation; ER = External regulation; AUT = Autonomy need satisfaction; COM = Competence need satisfaction; REL = Relatedness need satisfaction; NSAT = Need support; ID = Identified regulation; IJ = Introjected regulation; JP = Job Performance; JS = Job Satisfaction; TU = Turnover; BU = Burnout; WE = Work Engagement; WB = Well-Being.

^***^ *p* < .001 ^**^ *p* < .01 ^*^ *p* < .05
